# Revisiting the reference genomes of human pathogenic *Cryptosporidium* species: reannotation of *C. parvum* Iowa and a new *C. hominis* reference

**DOI:** 10.1038/srep16324

**Published:** 2015-11-09

**Authors:** Juan P. Isaza, Ana Luz Galván, Victor Polanco, Bernice Huang, Andrey V. Matveyev, Myrna G. Serrano, Patricio Manque, Gregory A. Buck, Juan F. Alzate

**Affiliations:** 1 Grupo de Parasitología, Facultad de Medicina, Universidad de Antioquia Carrera 53 No. 61-30, Medellin, Antioquia 05001, Colombia; 2 Centro Nacional de Secuenciación Genómica-CNSG, Universidad de Antioquia Carrera 53 No. 61-30, Medellin, Antioquia 05001, Colombia; 3 Universidad Mayor de Chile-Centro de Genómica y Bioinformatica Camino La piramide 5750 Huechuraba, Santiago de Chile, 8580000, Chile; 4 Virginia Commonwealth University – Center for the Study of Biological Complexity 1101 E. Marshall St., Virginia 23298-0678, US.

## Abstract

*Cryptosporidium parvum* and *C. hominis* are the most relevant species of this genus for human health. Both cause a self-limiting diarrhea in immunocompetent individuals, but cause potentially life-threatening disease in the immunocompromised. Despite the importance of these pathogens, only one reference genome of each has been analyzed and published. These two reference genomes were sequenced using automated capillary sequencing; as of yet, no next generation sequencing technology has been applied to improve their assemblies and annotations. For *C. hominis*, the main challenge that prevents a larger number of genomes to be sequenced is its resistance to axenic culture. In the present study, we employed next generation technology to analyse the genomic DNA and RNA to generate a new reference genome sequence of a *C. hominis* strain isolated directly from human stool and a new genome annotation of the *C. parvum* Iowa reference genome.

*Cryptosporidium spp.* are apicomplexan parasites of vertebrate organisms. Currently, 26 species with a broad host range including vertebrates from fish to mammals, are recognized by the scientific community^1^. Although human infections have been reported with more than 15 different species of this genus, *C. parvum* and *C. hominis* are of primary concern for human health. *C. parvum* infects cattle, sheep, goats and humans, as primary hosts, but *C. hominis* infects only humans and primates^1^,^2^. Originally, isolates from humans were classified into two genotypes of *C. parvum*. Type I the human genotype, or Type II the cattle genotype. Which exhibited clear differences in host range, host cell invasion, and genetics^3^,^4^,^5^,^6^. These genotypes are now considered discrete species. Both parasites are transmitted by fecal, food, and waterborne routes, and the course of infection depends largely on the immunological state of the host. A self-limiting watery diarrhea is common in immunocompetent humans, but immunocompromised individuals, including people with AIDS, cryptosporidiosis represents a life-threatening intestinal disease for which there are limited safe and effective treatment options available^7^.

The genome sequences of *C. parvum* Iowa strain and *C. hominis* TU502 strain, as reported a decade ago, both include approximately 9.1 Mbp, have GC contents of ~30%, and are reported to have introns in an estimated 5 to 20% of their genes^8^,^9^. Comparison of these genomes showed that chromosomes of these parasites are completely syntenic and exhibit only 3 to 5% sequence divergence at the nucleotide level. No significant insertions, deletions or rearrangements were detected and most differences in chromosome length are due to the remaining gaps within the current genome assembly^4^.

Additional *Crypstosporidium* species, including *C. muris* RN66, *C. hominis* UKH1, *C. baileyi* TAMU-09Q1 and *C. meleagridis* UKMEL1, have recently been sequenced, and a recent version of *C. hominis* TU502 genome was released as “*C. hominis* TU502 new”. This genomic data is available in public databases including GenBank and CryptoDB (http://cryptodb.org)^10^. Of these, the *C. parvum* Iowa strain sequence is the most complete, with a nearly complete gap free chromosome assembly and a comprehensive gene prediction and annotation. The *C. hominis* TU502 and *C. muris* RN66 genomes remain in multiple contigs with extensive gene prediction and annotation. The genome sequences of the remaining species are represented by multiple contigs with open reading frames (ORFs) identified. Empirical analysis of the expressed RNAs from different stages of *C. parvum* using 5′-EST and full-length cDNA libraries sequencing have improved predictions of the coding sequences of the genomes^11^,^12^. However, ambiguities in the gene models of all of these parasites; *i.e.*, transcription start and stop sites, translation start stop sites, identification of introns and intron-exon boundaries, clearly still require significant improvements. Moreover, there is still a need for optimization of the assemblies of these genomes, additional analyses supporting genome annotations, and identification and characterization of differences among these genomes that can explain the differing phenotypes; *i.e.*, host range, infectivity, pathogenesis, etc., among *Cryptosporidium* species.

It is also noteworthy that *C. hominis* TU502, although originally isolated from human stool, was propagated extensively in experimentally infected animals before its genome was isolated and sequenced^9^. Other protozoan pathogens; *e.g. Trypanosoma cruzi* and *Toxoplasma gondii*, have been shown to have altered activities, some of which may be due to genetic alterations, after long term maintenance under laboratory conditions^13^,^14^. Although the effect of serial passages in animal models on genetic composition of *Cryptosporidium spp.* is not yet known, it is likely if not certain that passage in a restrictive host; *i.e.*, piglets for the human pathogen *C. hominis*, would select for some genetic modifications. Thus, analysis of a more recent isolate from a human sample would be appropriate.

Herein, we describe analysis of a new *C. hominis* reference genome from a parasite that was directly isolated from an infected human, and a significant improvement of the *C. parvum* Iowa genome annotation available on the CryptoDB database, based on full-length cDNA clone sequences generated by capillary sequencing and RNA-Seq data generated by high throughput NGS.

## Results

### An improved gene annotation (IGA) of the *C. parvum* Iowa strain genome

The original genome annotation (OGA), *C. parvum* Iowa release 6, available in the CryptoDB database has 3,805 protein coding genes distributed among eight chromosomes. Inevitable gene calling errors result in the lack of a correct translation start or stop codons, or correct splice junctions on some of these genes. To correct these errors to permit more efficient and accurate comparative genome analysis, we compiled additional sequence information to build more accurate gene models. Thus, we used a MAKER software pipeline with 8,065 and 9,496 sequences from full length cDNA libraries generated from oocyst and sporozoite mRNA, respectively, to optimize our gene calls. The average sizes of these cDNA sequences were 929 nt for the sporozoite library and 705 nt for the oocyst library. Subsequently, RNA-seq transcriptome data was obtained from a 48 hours time-course *in vitro* infection of cultured HCT-8 cells. The resultant reads were pooled and treated as single dataset. Approximately 4% of these reads (5,540,140) mapped to the *C. parvum* reference genome; 5,299,229 reads mapped to the predicted genes. The remaining reads, largely from the HCT-8 cell transcripts, were ignored in this study. A median of 247 reads were mapped to each *C. parvum* gene, and 91% of the genes were supported by at least 10 reads. Only 89 genes were not represented by any reads in this RNA-seq dataset. This dataset was employed to validate and improve the gene models of *C. parvum*; *i.e.*, to identify reading frames, intron and exon boundaries.

**Figure 1:**
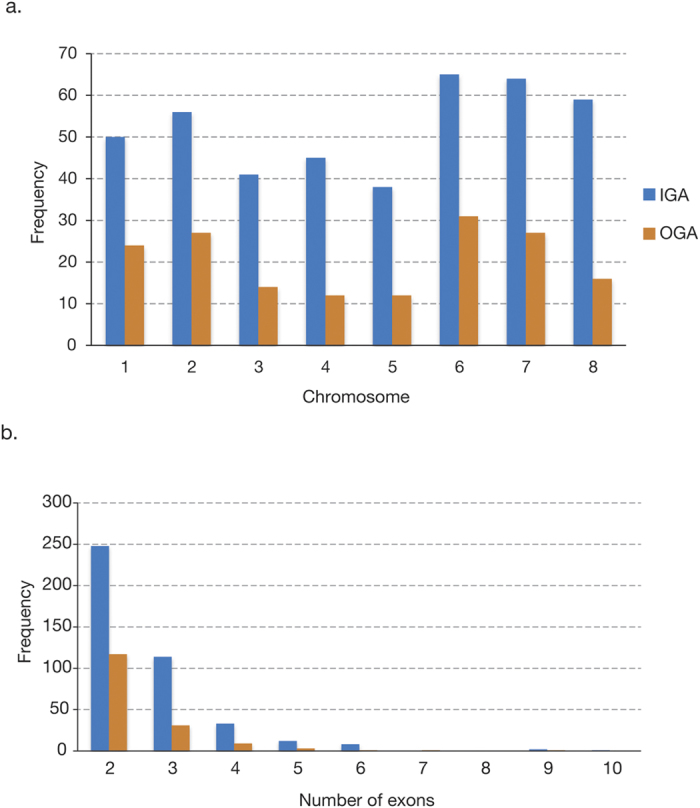
Frequency of genes with introns across chromosomes and number of exons per gene. Bar charts showing (**a**) Distribution of genes with introns in *C. parvum* Iowa chromosomes and **b**) exons distribution among genes of *C. parvum* Iowa genome (intronless genes were excluded). IGA (Blue) stands for Improved Genome Annotation (this work) and OGA (Orange) stand for Original Genome Annotation.

Our improved genome annotation (IGA) identified differences in the number of genes as well as the intron content of the coding genome of *C. parvum* Iowa (Table 1). IGA identified 3,865 protein-coding genes, 74 of which were not identified in the OGA. As expected most of the genes; *i.e.*, ~90% or 3,446, were demonstrated to lack introns, but whereas OGA reported that only 4.3% of the genes contain introns, while the IGA clearly identified introns in ~10.8% of them. Moreover, fourteen coding regions of the OGA were exons belonging to flanking genes in the new annotation (Supplementary Table S1). Thus, our analysis identified 511 additional exons and 451 additional introns not observed in previous annotations. Overall, genes with introns have between 2 and 10 exons, with a mean of 2.6 exons per gene (Fig. 1b).

To characterize the newly predicted genes, we carried out a BLASTP search against the non-redundant (NR) NCBI protein database. Only 54 genes had at least one hit under the established e-value threshold. Twenty-six of the best hits were with *C. muris* genes, 21 were with *C. hominis* genes, and only two best hits were with genes annotated in species outside the Phylum Apicomplexa. Of the BLASTP hits, 49 were annotated as hypothetical proteins. The remaining five were annotated as *ribonuclease P/MRP protein subunit POP5, hydrogen-transporting ATP synthase, ci-meta2, REX1 DNA repair domain-containing protein* and *uracil-DNA glycosylase family protein.*

To evaluate the overall improvement in gene models in the new annotation, we took both datasets OGA and IGA and ran HMM searches against PFAM-A and SCOP databases. In the IGA dataset there is a slight increase in the number of unique PFAM domains, while SCOP domains remain unchanged. In the PFAM analysis, 1,939 of 1,969 domains are shared between both datasets, 7 domains were lost and 30 domains were gained in the IGA dataset (see Supplementary table S2). However, assessment of the sequential order in which domains are linked in the proteins; *i.e.*, the protein domain architecture (PDA), identified 143 new PDAs. We also found 502 genes in IGA with at least one EC code, 2 more than were reported in the OGA, but no difference was observed in the EC codes assigned to each dataset. A total of 2,030 genes in the IGA were assigned to 967 different GO terms, although there was no statistically significant difference in the terms assigned to both datasets (data not shown).

### Genetic characterization of a Colombian *C. hominis* isolate

**Figure 2:**
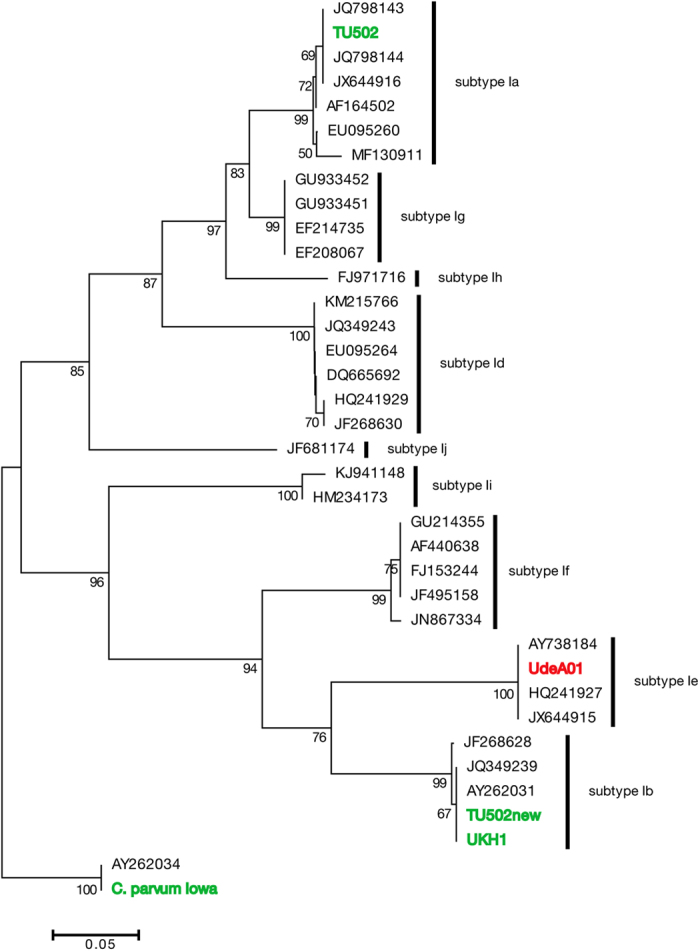
Phylogenetic position of *C. hominis* UdeA01 based on partial sequence of GP60 gene. Phylogenetic tree based on partial sequence of GP60 gene from different isolates of *C. hominis* and *C. parvum*. The tree was constructed by Neighbour-Joining method and 1, 000 bootstrap replicates. Bootstrap values above 50 are shown. The tree was rooted with the GP60 sequence of *C. parvum* Iowa.

To avoid possible impacts of selection pressure during animal passage or cell culture, we directly enriched and analyzed parasite oocysts from the stool of an infected HIV positive woman. After microscopic confirmation of the *Cryptosporidium* infection, the oocysts were enriched by sodium chloride differential flotation. To genotype the isolate, a phylogenetic analysis was carried out with the nucleotide sequence of the highly polymorphic genes HSP70 and GP60. A neighbour-joining phylogenetic analysis using the partial HSP70 nucleotide sequence confirmed that this was a *C. hominis* isolate (data not shown). The inferred tree topology using the gene GP60 indicated that this *C. hominis* isolate, termed UdeA01, belongs to the subtype family Ie and the assigned subtype was IeA11G3T3. Our phylogenetic reconstruction showed that *C. hominis* TU502 and “*C. hominis* TU502 new” are quite divergent and positioned on two different subtype families, Ia and Ib respectively. In contrast, *C. hominis UKH1* and “*C. hominis* TU502 new” comprise a clade inside subtype family Ib (Fig. 2).

### *Cryptosporidium hominis* UdeA01 genome assembly and annotation

Approximately 12 × 10^6^ oocysts were enriched by differential flotation of *C. hominis* oocysts in a saturated solution NaCl. Semi-quantitative PCR analysis using the GP60 and 16S genes as targets showed that the *C. hominis* genome was enriched relative to bacterial DNA after the NaCl flotation experiment, (data not shown). After DNA extraction, whole genome shotgun sequencing was performed in the Roche 454 FLX(+) and the Illumina MiSeq platforms.

A total of 2,041,315 454 FLX(+) reads and 18,190,842 Illumina reads were obtained after sequencing, of which 276,886 (13%) and 2,132,921 (12%) respectively, were assembled using the *C. hominis* UdeA01 contigs. *De novo* assembly of these reads yielded 112,561 contigs (>100 bases), with an N50 of 5,764 bases. BLASTN searches of these contigs against the *C. parvum* reference genome identified 124 contigs longer than 500 bases that belonged to *C. hominis* UdeA01 isolate. This contig set summed a total of 9.05 million bases and its basic metric parameters can be found in Table 2.

Assuming the complete genome synteny reported between *C. hominis* TU502 and *C. parvum* Iowa, we built the 8 chromosome scaffolds of the parasite genome using ABACAS, and performed an automatic gap filling procedure using the IMAGE pipeline, thereby reducing the number of gaps in the chromosomes to 86. During the contig scaffolding process we observed two structural incongruences between *C. hominis* UdeA01 and *C. parvum* Iowa chromosomes. The first involves an inverted region in chromosome 2. In this region, the *C. parvum* reference has two genes, cgd2_10 and cgd2_470. In our chromosome assembly, the inverted region merged both gene models into a single gene. The second incongruence, located in chromosome 5, starts at the intergenic region after gene cgd5_3100 and expands to the telomere. This region presents several gaps and one stretch with ambiguous bases in the *C. parvum* reference. Although this region was assembled in several contigs in *C. hominis* UdeA01, two contigs were assembled that clearly show two conflicting junction points. The first conflict is that genes cdg5_3100 to cdg5_4500 are neighbors in the *C. hominis* UdeA01 *de novo* contig. Similarly, cdg5_3110 and cdg5_3610 were assembled contiguously without any base ambiguities. These incongruences were confirmed by PCR using primers targeting both ends of the contigs involved in the conflictive junctions. Mazurie and coworkers reported similar observations when they compared *C. parvum* Iowa and *C. hominis* TU502 reference chromosomes^4^.

In chromosome 5, genes from cgd5_4510 to cgd5_4610 in *C. parvum* Iowa were not assigned to the assembled chromosomes of *C. hominis* UdeA01. However, most of these genes (cgd5_4510, cgd5_4520, cgd5_4540, cgd5_4560, cgd5_4570, cgd5_4600 and cgd5_4610) were found on 4 unassigned contigs that totalling approximately 25 kb.

**Figure 3:**
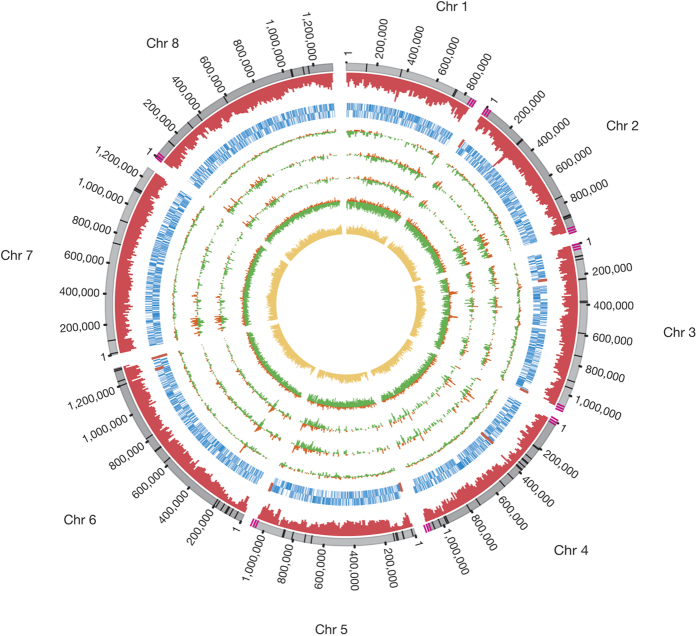
Circular representation of *C. hominis* UdeA01 genome. Graphical representation of *C. hominis* UdeA01 chromosomes. Circles from outer to inner represents: Chromosomes (grey); histogram of read coverage in windows size of 10 kb (red); forward CDSs (blue); reverse CDSs (blue); C. hominis TU502 – C. hominis UdeA01 intergenic region SNPs density (orange) and coding region SNPs density (green); “*C. hominis* TU502 new” – *C. hominis* UdeA01 intergenic region SNPs density (orange) and coding region SNPs density (green); *C. hominis* UKH1 – *C. hominis* UdeA01 intergenic region SNPs density (orange) and coding region SNPs density (green); *C. parvum* Iowa – *C. hominis* UdeA01 intergenic region SNPs density (orange) and coding region SNPs density (green); GC content in window size of 10 kb (yellow). Black bars in chromosome circle represents the remaining gaps. Three pink bars represent the telomeric region where it was reached. Red bars over CDS circles represent the location of eleven genes of *C. hominis* UdeA01 absent in *C. parvum* Iowa genome.

The resulting genome of *C. hominis* UdeA01 is 9.05 Mb in length, presents a mean coverage of 68X, contains 86 gaps and exhibits a GC content of 30% (Fig. 3).

Oocysts isolated directly from an infected human may offer the possibility to analyse the presence of a different clone mixture of *Cryptosporidium* parasites. For this purpose, the sequence variation within the Illumina reads data set was measured. Over the whole genome, excluding Copy Number Variation (CNVs) or Indel regions, 726 high quality SNPs were detected. This suggests that a possible mixture of genotypes is present in this Colombian patient, however the number is far below previous studies where two isolates of *C. parvum* were analyzed and around 12.000 SNPs were detected^6^. Interestingly, in those positions with an alternative allele, the proportion of reads that support the reference and the alternative were uneven, with a media ratio close to 1:3 in favor of the reference (Supplementary Figure S3). Detailed analysis of the SNPs positions showed that the presented genome reference of *C. hominis* UdeA01 is composed in the vast majority of the dominant genotype, because only 74 nucleotide positions exhibit a higher read depth of a base that is not that of the reference sequence.

The obtained data do not support the hypothesis that the volunteer sample harboured a mixture of multiple parasite strains. Rather, the results fit better the possibility that at most two very similar clones were present, but one is predominant and is represented in the genome reference of *C. hominis* UdeA01.

The eight chromosomes have a total of 3,819 coding sequences, fewer than the CDS sequences predicted for *C. parvum* Iowa. This difference could be due to the exclusion of CDS sequences that contain gaps and genes contained in the unassigned contigs. A total of 3,809 CDS were automatically transferred from our upgraded version of *C. parvum* Iowa annotation (IGA) to the *C. hominis* UdeA01 chromosomes and manually curated using ARTEMIS. Additionally, 11 protein-coding sequences not present in *C. parvum* were identified in *C. hominis* UdeA01 chromosomes. These CDSs are located mainly in chromosome regions where our contigs exceed *C. parvum* Iowa contig lengths. Nine of these CDS encode for hypothetical proteins, one is 40S ribosomal protein S11 and the last one is a DinB/family X-type DNA polymerase, a new locus of cgd4_3920 from *C. parvum* Iowa. For a more detailed description of these genes, see Table 3.

Excluding coding regions with internal gaps, a total of five protein-coding genes from *C. parvum* Iowa were absent in the *C. hominis* UdeA01 genome sequence. Two telomeric insulin-like peptidases (cgd6_5510 and cgd6_5520) were not found in the assembled chromosomes or unassigned contigs of *C. hominis* UdeA01. Although the reported localization of these genes is at the 3′ end of chromosome 6 in *C. parvum*, none of these genes were found in the available contigs of *C. hominis* UKH1, *C. hominis* TU502 or *“C. hominis* TU502 new”. However, in this chromosomal region we identified 2 ORFs with high similarity to cgd2_3560 and cgd2_3570.

The genes cgd8_680 and cgd8_690 were also absent in the analyzed *C. hominis* genomes. In this region, our assembly shows neither gaps nor ambiguities, whereas the *C. parvum* Iowa reference presented a large gap of 10.000 bases. These results need to be interpreted with care because the, cgd8_680 nucleotide sequence aligned with the 3′ end of the cgd8_660, while cgd8_690 aligned with the 5′ region. The *C. parvum* Iowa genome assembly should be verified in this region.

Finally, genes cgd6_5480 and cgd6_5490 correspond to a tandem duplication event in *C. parvum* Iowa, whereas in *C. hominis* genomes the former gene is absent.

### Comparative genome analysis between *C. hominis* strains and *C. parvum* Iowa

Single nucleotide polymorphisms (SNPs) were extracted from collinear blocks built with the MUMMER software package. In this analysis, we included the public available genomes of *C. hominis* TU502, “*C. hominis* TU502 new”, *C. hominis* UKH1 and *C. parvum* Iowa. A total of 37,504 SNPs were predicted in protein-coding regions, while only 7,807 were observed in non-coding regions, representing approximately 0.4% and 0.1% of the genomes. Comparative analysis between “*C. hominis* TU502 new” and *C. hominis* UKH1 at single nucleotide polymorphisms level suggest that they are closely related, which is also confirmed by a phylogenetic analysis of the GP60 coding sequence (Fig. 2). One hundred fifty-two SNPs including coding and non-coding regions were found between these strains, in contrast to the 1,857 SNPs we observed between the clinical isolates *C. hominis* UdeA01 and *C. hominis* UKH1.

Similar to the previously reported percentage of non-synonymous SNPs (nsSNPs) between *C. parvum* Iowa and *C. parvum* TU114 (Anthroponotic strain)^6^, we found that the percentage of nsSNPs was homogenous across genomes, ranging from 30% to 34%, with an average genome-wide frequency of 31% of non-synonymous substitutions between each *C. hominis* strains compared with *C. parvum* Iowa. On the other hand, the percentage of non-synonymous substitutions between *C. hominis* strains is higher, ranging from 62% to 65%, excluding the comparison between “*C. hominis* TU502 new” and *C. hominis* UKH1, which showed a frequency of 48% of non-synonymous SNPs.

**Figure 4:**
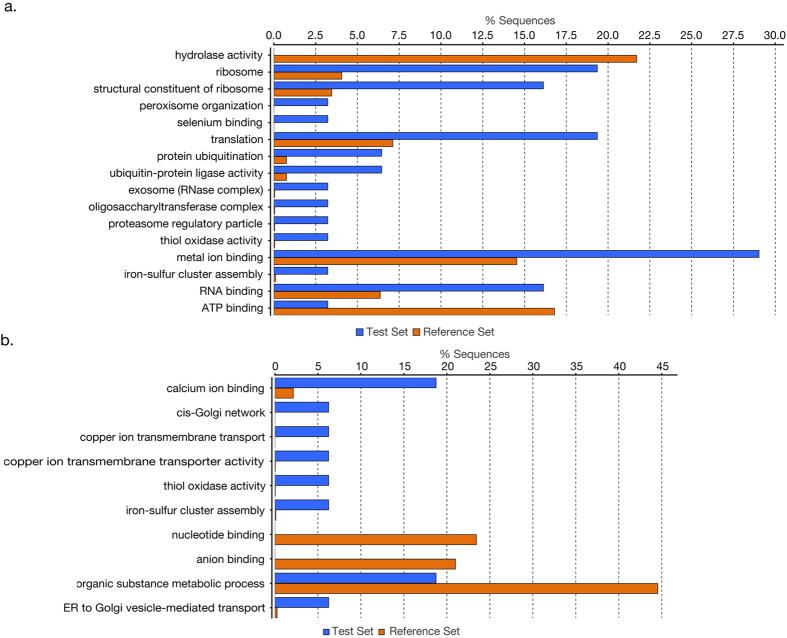
Gene Ontology enrichment analysis of highly divergent genes between *C. hominis* isolates. Gene Ontology enrichment analysis for (**a**) 77 highly divergent genes between *C. hominis* TU502 and *C. hominis* UdeA01 (test) whole putative *C. hominis* UdeA01 proteome (reference). (**b**) 37 highly divergent genes between *C. hominis* UdeA01 and *C. hominis* UKH1 (test) whole putative *C. hominis* UdeA01 proteome (reference). GO terms found over/under represented by a two-tailed Fisher Exact test with a p-value below 0.05.

One of the limitations of this comparative analysis is the lack of a confident annotation in the available reference genomes of *C. hominis*, leaving neither the possibility for orthologous gene comparisons or the ability to calculate dN/dS ratio to identify proteins under selection pressure. Thus, to detect highly divergent genes between *C. hominis* strains, we calculated the SNPs density (SNPs/kilobase) in the protein-coding genes based on the localization of *C. hominis* UdeA01 CDSs. We established a threshold value of 2.1 SNPs/kb to classify genes with a high density of SNPs. To calculate this cutoff, two standard deviations were summed to the median value of the SNPs/kb distribution in protein-coding regions. This threshold is 6.7 fold and 9.7 fold of the whole genome frequency of SNPs/kb in *C. hominis* TU502 and *C. hominis* UKH1, respectively. Using this approach, we identified 77 highly divergent genes between *C. hominis* TU502 and *C. hominis* UdeA01. A two-tailed Fisher exact test of the associated Gene Ontology terms to these genes showed an over representation of the terms: Ribosome (p-value 0.0016), structural constituent of ribosome (p-value 0.0046), translation (p-value 0.022), and metal ion binding (p-value 0.037); while the terms hydrolase activity (p-value 0.0012) and ATP binding (p-value 0.048) were under represented (Fig. 4a). Similarly, we found 37 genes with an SNP density higher than 2.1 SNPs/kb between *C. hominis* UdeA01 and *C. hominis* UKH1. In these analyses, the more over represented term was calcium ion binding (p-value 0.005), while the terms nucleotide binding (p-value 0.032), anion binding (p-value 0.032), and organic substance metabolic process (p-value 0.044) were under represented (Fig. 4b).

**Figure 5:**
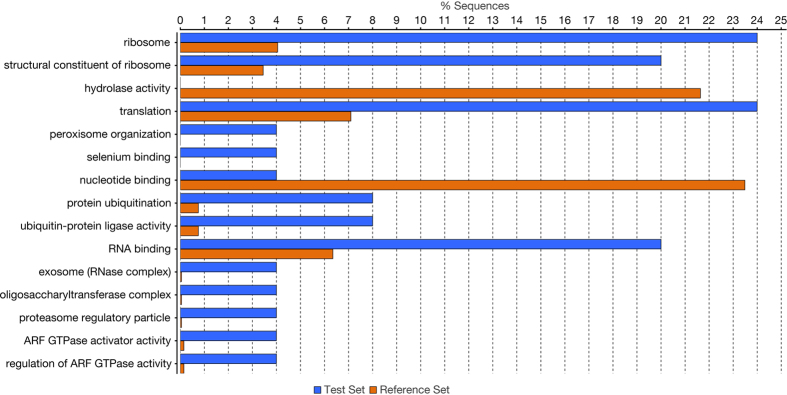
Gene Ontology (GO) terms overrepresented in the highly divergent genes with specific SNPs of *C. hominis* TU502. Gene Ontology enrichment analysis for highly divergent genes with specific SNPs of *C. hominis* TU502 (test) whole putative *C. hominis* UdeA01 proteome (reference). GO terms found over/under represented by a two-tailed Fisher Exact test with a p-value below 0.05

We therefore wanted to determine polymorphic sites that might be involved in a possible adaptation process of *C. hominis* TU502 to its laboratory host. Thus, we selected SNP positions where the human-derived isolates shared the same nucleotide, but that position varied from its counterpart in the animal propagated isolate. In 1,090 protein-coding genes we identified 1,748 specific SNPs positions of *C. hominis* TU502. Among these, only 64 genes were classified with high density SNPs. The enriched GO terms associated to these genes were Ribosome (p-value 0.00048), structural constituent of ribosome (p-value 0.0017), translation (p-value 0.0076) and RNA binding (p-value 0.02) (Fig. 5).

For a comparative analyses of the protein-coding genes with the zoonotic *Cryptosporidium parvum* Iowa strain, we used the improved genome annotation reported here to perform an orthologous gene comparison over 3,770 amino acid sequences against *C. hominis* UdeA01 putative proteome. Four hundred and seven of these proteins have an identity lower than 95%, and 219 are 100% identical proteins; the remaining (83%) putative proteins have an identity between 95 and 99%. The changes in the coding sequences are mainly due to nucleotide indel or substitution events that induce frameshifts and result in truncated or longer proteins. However, some proteins in *C. hominis* UdeA01 are longer than those in *C. parvum* because this latter has some low quality regions within the assembly, resulting in split open reading frames. Exploring the annotation of the 113 more divergent proteins with an identity below 90% (Supplementary Table S4), we found that 49 (43%) have transmembrane domains predicted by TMHMM, whereas in the overall proteome transmembrane domain prediction we identified 818 (21%) proteins, which is statistically significant (Pearson’s Chi-squared, p-value = 6.45e–05).

In order to identify proteins under positive, negative or neutral pressure selection, the dN/dS ratio was calculated over the orthologous protein dataset (Supplementary Table S5). We found that 22 proteins with a dN/dS ratio greater than 1.1, nineteen of these proteins are annotated as hypothetical proteins and the INTERPROSCAN results did not show any clue of their possible function. The remaining proteins are cgd1_2880 (13 kDa membrane protein subunit), cgd4_1230 (60S ribosomal protein L28) and cgd5_1260 (DHHC-type zinc finger domain-containing protein). Five of the proteins with positive pressure selection (23%) have transmembrane domains predicted by TMHMM, although there is no statistical significance (Pearson’s Chi-squared, p-value = 1) compared to the whole putative proteome proportion of 21% of transmembrane proteins.

To identify larger sequence polymorphisms (LSPs), flanking sequences of collinear blocks were extracted and pairwise aligned using MUSCLE. With this analysis, we identified in the *C. hominis* UdeA01 genome 99 insertions and 122 deletions larger than 30 nucleotides (Table 4). A total of 107 genes have at least one indel event within the CDS and 62 genes have an indel event in the nearest 1,000 nt upstream of the predicted start codon.

The largest insertion in *C. hominis* UdeA01 was a 4,452 nucleotides region located in the chromosome 4 between the genes cgd4_1530 and cgd4_1540. This region corresponds to a gap in the current version of the *C. parvum* Iowa genome and contains three ORFs with sizes in nucleotides of 1,989, 507 and 342. A BLASTP search showed hits with DinB/family X-type DNA polymerase (cgd4_3920) of *C. parvum*, 40S ribosomal protein S11 of *C. muris* and a hypothetical protein of *Neospora caninum*, respectively. A deletion of 1,248 bp was identified in chromosome 7 within the cgd7_2870 gene, which encodes an uncharacterized low complexity protein. This deletion is located in the repetitive region of this gene. Another deletion of 522 nucleotides was identified, in the coding region of the gene cgd5_1940, which encodes a hypothetical protein in chromosome 5.

## Discussion

The *Cryptosporidium hominis* TU502 genome available on CryptoDB database was obtained from oocysts initially isolated from human feces and then propagated extensively in calves and gnotobiotic piglets before being sequenced. *C. parvum* Iowa, a zoonotic strain, oocysts were isolated directly from infected calves for sequencing^8^,^9^. In addition to these genomes, two new genomes of *C. hominis* have been released in the 7^th^ version of CryptoDB database without any annotation. This release includes a new sequencing project of *C. hominis* TU502 named as “*C. hominis* TU502 new” and a *C. hominis* strain isolated from a human clinical sample from United Kingdom, UKH1. Thus, we elected to sequence a human-derived isolate of *C. hominis* without animal propagation, with the aim of performing a comparative genomic analysis that might provide a better understanding of the genomic adaption to their natural host.

Despite the fact that *C. parvum* Iowa and *C. hominis* TU502 are 95–97% identical in nucleotide sequence, incongruences in the annotated gene models are obvious in cryptoDB database. *C. parvum* Iowa has 3,805 annotated protein-coding genes, while *C. hominis* TU502 has 3,886. Additionally some gene models are still incomplete; lacking the correct number of exons and some genes do not start from an ATG. To improve the structural and functional annotation of *C. parvum* Iowa genome and permit further comparative analysis with *C. hominis* UdeA01 genome reported here, two full-length cDNA libraries derived from oocysts and sporozoites of *C. parvum* Iowa strain and transcriptome data from RNA-seq analyses were generated to improve the gene models deposited in cryptoDB version 6.

A slight increase in the number of protein-coding genes was found during the re-annotation process, *i.e.*, 74 new genes were identified with evidence supported for the cDNA libraries or RNA-seq. Ten percent of the annotated gene models had at least one intron, double the number of intron-bearing genes previously reported. Regardless of the number of new genes and exons discovered here, most of these (66%) were hypothetical proteins, so few new biological functions were revealed. This result can be explained for the high proportion of hypothetical proteins in related apicomplexan genomes; *i.e*, 51% in *Toxoplasma gondii*, 51% in *C. muris* and 61% in *C. hominis.* Nevertheless the IGA dataset includes a more comprehensive and accurate gene model collection. In a previous work using 1,066 full-length cDNA clones of *C. parvum* HNJ-1, six hundred and eighty sequences were found to be unique, among these only 562 were assigned to annotated gene models of *C. parvum* Iowa. Twenty six percent had at least one discordance with the reported gene; with 85% and 18% of the inconsistencies were located at the 5′ and the 3′ end respectively^12^. These issues are not only a concern in *Cryptosporidium* species. Reference genomes of other apicomplexan parasites, including *Plasmodium falciparum* and *T. gondii* also contain inconsistencies between the annotated gene models and transcriptome evidence. Wakaguri and coworkers, using 5′ end partial cDNA libraries of six apicomplexans (*Plasmodium falciparum*, *Plasmodium vivax*, *Plasmodium yoelii*, *Plasmodium berghei*, *C. parvum* and *T. gondii*) found that more frequent discrepancies were at gene level, which means that gene models contained at least one mis-annotated exon. For *Plasmodium* species the frequency of this error ranges from 9% to 21% of the annotated genes. *Toxoplasma gondii* showed the higher rate of mis-annotated exons with 24%; while in *C. parvum* only 5% of genes had this error^11^, which is very close to the 5.7% found with our analyses. This low level of error in the annotated gene models of *C. parvum* is consistent with the compactness of its genome and the small percentage of predicted genes with introns^8^.

A challenging aspect in *Cryptosporidium* research is the lack of an *in vitro* culture system. As a consequence, oocysts are propagated in suitable laboratory animals, possibly complicating the identification of genetic differences and their relationship to phenotypic characteristics. The *C. hominis* UdeA01 genome sequence presented here, is the result of a hybrid sequencing strategy that involved whole genome shotgun in the 454 FLX(+) and Miseq 2 × 250 platforms. Following a metagenome-like approach we were able to sequence, assemble and annotate a human isolate of *C. hominis*. This isolate represents the first reference genome for the American continent and also the first of the subtype family Ie available.

This family of *C. hominis* is reported to be less frequent and associated mainly with children from developing countries like Bangladesh, India, Uganda and Peru^15^. Clinical manifestations of human cryptosporidiosis varied among *C. hominis* subtype families, infections with the subtype family Id are associated with more diverse and severe clinical manifestation, while infections with the subtype family Ie are associated with diarrhea episodes^16^,^17^,^18^.

To date, there is no evidence of *C. parvum* and *C. hominis* recombination in nature. This could be attributed to a reproductive isolation between these species^5^, precluding any suitable genetic linkage analysis to determine chromosomal regions involved in host specificity. Alternative strategies have been used for this purpose, including the non-synonymous versus synonymous substitutions ratio calculation. Previous comparative analysis between *C. parvum* Iowa and *C. hominis* TU502 showed that proteins with highest dN/dS ratios were surface-associated proteins, including proteins with signal peptides or transmembrane domains^4^,^19^. Following a similar methodology we evaluated the evolutionary pressure between orthologous genes of *C. parvum* and *C. hominis* UdeA01. We identified 22 genes with a dN/dS ratio greater than 1.1; among these, the 23% encode for proteins with transmembrane domains. Although this result was not statically significant, it allowed us to notice a tendency to an increased genetic divergence in genes encoding surface-associated proteins. This observation was later confirmed by the predicted cellular localization of the highly divergent genes (genes with an identity lower than 90%), in which we showed that 49 (43%) contain transmembrane domains. These results are consistent with early works on others apicomplexan parasites like *Plasmodium* and *Theileria*, in which genes encoding merozoite surface proteins yielded the highest dN/dS ratios^20^,^21^,^22^. Some proteins with the orthologous identity were previously characterized as putative surface proteins, which may play a role in the host-parasite interaction. These include mainly mucins (cgd2_430, cgd4_3550, cgd8_700, cgd2_420, cgd6_5400, cgd6_5410), one secreted protein (cgd1_470) and one Kazal-like serine protease inhibitor (cgd8_3550). In addition, two proteins associated with the invasion organelles microneme and dense granules were identified as fast evolving proteins, cgd7_4020 (known also as GP900) and cgd7_4500, respectively^23^. Interestingly, none of the above mentioned genes were detected as highly divergent by the implemented methodology of SNPs density in the comparative analysis between *C. hominis* UdeA01 and *C. hominis* TU502 or *C. hominis* UKH1. This observation suggests that this protein repertoire may play a role as determinant for host specificity.

Previous reports on protozoan parasites have shown that serial passages in laboratory animals or cell cultures can alter the parasite growing rate and result in loss of virulence. Thus, the *Theileria annulata* Hisar S45 cell line gradually loses virulence in cattle after continuous propagation *in vitro*^24^, the *Toxoplasma gondii* RH strain showed decreased pathogenicity after a single passage in rats^13^. And *in vitro* passages of *Leishmania infantum* and *Trypanosoma cruzi* resulted in loss of virulence, in the former by an inadequate capacity to differentiate into amastigote forms and the latter as a consequence of a reduced capacity to undergo metacyclogenesis^14^,^25^. In contrast, Chan and coworkers showed that adaptation and continuous propagation of *Plasmodium vivax* on monkeys does not induce systematic changes in the parasite genome^26^. *C. hominis* TU502 was originally isolated from a child with cryptosporidiosis in Uganda. This isolate was consecutively propagated on gnotobiotic piglets and sometimes in calves. Later it was isolated from a person of the laboratory who got accidentally infected^27^. Thus, we selected *C. hominis* TU502 specific SNPs for detailed analysis. Among these, we found that genes with highest density of SNPs are involved in ribosome assembly and translation processes, but this pattern was not observed in the comparative analyses between the human direct derived isolates. Although these results suggest that an adaptation process could be taking place in the parasite, it is important to highlight that the isolates compared here represent different subtype families. *C. hominis* TU502 belongs to the subtype Ia while *C. hominis* UdeA01 and *C. hominis* UKH1 are Ie and Ib, respectively. In fact, molecular epidemiology studies based on GP60 nucleotide sequence have shown that some subtypes are more virulent^16^,^17^,^18^. Thus, to gain knowledge about any adaptation process of *C. hominis* to its laboratory host is required to sequence and analyse the genome of the same strain before and after the animal propagation.

## Methods

### Isolates

*Cryptosporidium parvum* oocysts (Iowa strain) were bought from the University of Arizona, Sterling Parasitology Laboratory. *C. hominis* oocysts (UdeA01 strain) were isolated from the stool of a female HIV patient residing in Colombian in 2013.

### *Cryptosporidium parvum* genome reannotation

Two normalized full cDNA libraries were constructed from oocysts and sporozoites using SMARTer RACE cDNA Amplification kit following manufacturer’s instructions (Clontech laboratories Inc.). High-level normalization of the cDNA libraries was achieved using Trimmer cDNA Normalization Kit (Evrogen) followed by cloning in pGEM-T Easy Vector System (Promega) and sequenced using capillary sequencing. Additionally, a 48 hours time-course infection transcriptome by RNA-seq dataset was generated from HCT-8 infected cells with freshly excysted oocysts of *C. parvum* Iowa strain. *C. parvum* oocysts were added to uninfected HCT-8 cells at a MOI of 1:3.75 to establish a synchronous infection. To allow for adherence, *C. parvum* oocysts were incubated with 1 × 10^6^ HCT-8 cells in 1 ml for 1h at 37 °C. Cells were washed 6x with phosphate-buffered saline (PBS) to remove unbound oocysts, and incubated at 37 °C in 5% CO_2_. Infected cells were collected at 1, 2, 4, 6, 12, 24 and 48 h post infection. RNA was extracted using RNAqueous kit, following manufactuer’s instructions (Life Technologies). Poly-A enriched RNA was sequenced on the HiSeq2000 platform with 2 × 100 paired-end libraries (Illumina Inc.) at the Center for the Study of Biological Complexity (Virginia Commonwealth University). A total of 129,665,844 read pairs were generated across all time points.

Data generated from full-length cDNA libraries were used to build gene models. The dataset included 8,065 sequences obtained from oocysts and 9,496 sequences belonging to sporozoites. Vector and low quality regions were removed from reads using the Sequence cleaner tool and UniVec database from NCBI (retrieved on July 30, 2014)^28^. Filtered reads were mapped to the *C. parvum* Iowa genome (release 6, available at http://cryptodb.org) using the MAKER genome annotation pipeline^29^, which combines *in silico* prediction with protein homology and transcriptome data. The pipeline included: i) initial identification and masking of repeat elements using repeatMasker; ii) EST dataset (cDNA libraries) mapping with the program EXONERATE^30^; iii) *Ab initio* gene prediction with the program AUGUSTUS^31^ with Toxoplasma as reference species; and iv) GENEMARK-ES^32^ unsupervised gene prediction.

RNA-seq reads were aligned to the *C. parvum* Iowa genome using TopHat, a splice junction mapper built upon the short read aligner Bowtie^33^. The alignments were merged and treated as a single dataset used to assembly transcripts with CUFFLINKS^34^. The overall number of RNA-seq read pairs that mapped the *C. parvum* genome were 5,540,140, around 4%, and 5,299,229 reads were mapped to the gene models predicted on the IGA. The full-cDNA libraries and RNA-seq gene models were manually inspected using APOLLO^35^ and ARTEMIS^36^.

The *C. parvum* OGA was used as a reference (CryptoDB, release 6) for the *C. parvum* IGA. In order to include a new gene model, it must comply with support of the TopHat read mapping and CUFFLINKS or MAKER prediction. Those gene models that lack expression evidence of intron and exon discrepancy were left unaltered in the IGA version.

### *Cryptosporidium hominis* UdeA01 sequencing

*Cryptosporidium hominis* UdeA01 oocysts were purified from a fecal sample collected from an HIV Colombian female patient by flotation in a saturated NaCl solution^37^. The sample was stored at 4 °C with an antibiotic cocktail until DNA was extracted. There was no animal model for parasite propagation and oocyst production in this study. DNA was extracted using DNeasy blood and tissue kit (Qiagen) with a modification in the lysis step. Briefly, oocysts were resuspended in ATL buffer and then subjected to 10 freeze-thaw cycles (each for two minutes) in liquid nitrogen and water bath (90 °C).

454 FLX + (Roche, Basel, Switzerland) sequencing was performed at the National Center for Genomic Sequencing, Universidad de Antioquia. Illumina sequencing was performed at the Center for Genomics and Bioinformatics, Universidad Mayor de Chile. All genomic libraries were generated according to the manufacturer’s protocols.

### *Cryptosporidium hominis* UdeA01 genome assembly and annotation

Low quality reads were removed using PRINSEQ^38^, based on the following parameters: minimum read length of 50 bases; remove exact/reverse complementary duplicated reads; trim reads at the 5′-end to 10th base and trim reads by quality score from the 3′-end with a threshold score lower of 30. For *de novo* genome assembly, NEWBLER^39^ version 2.9 was used with default parameters. Due to the mixture of different microorganisms within the enriched *C. hominis* UdeA01 purified DNA, a metagenomic read dataset was obtained. For this reason the *Cryptosporidium* assembled contigs were selected using a BLASTN^40^ with a threshold e-value of 1e-20 and *Cryptosporidium parvum* Iowa genome and *C. hominis* TU502 genomic contigs as subjects. These contigs were assigned with ABACAS^41^ to pseudo-chromosomes using as reference *C. parvum* Iowa chromosomes. Furthermore some gaps were closed by 12 iterations of IMAGE^42^ with a k-mer of 91 for each chromosome, and inconsistencies were confirmed by PCR and capillarity sequencing. Single nucleotide variations (SNV or SNPs) were called using the program NGSEP^43^ with the module FINDVARIANTS and following settings: -minQuality = 70; -maxBaseQS = 30; -maxAlnsPerStartPos = 2. For this analysis all the illumina reads were mapped to the *C. hominis* UdeA01 assembled chromosomes using bowtie 2 with default settings.

RATT^44^ was used to automatically transfer the upgraded *C. parvum* Iowa genome annotation to the *C. hominis* UdeA01 pseudo-chromosomes, followed by manual inspection of the coding regions in ARTEMIS^36^.

### Functional annotation

Protein sequences were searched against the Structural Classification Of Proteins (SCOP) database^45^ with an e-value threshold at superfamily level of 1e-3. Additional functional annotations were assigned using PFAM-A version 27^46^ Hidden Marcov Models (HMM) to scan the predicted proteins using the HMMER package^47^ with an e-value threshold of 1e-3.

BLAST2GO^48^ version 2.8 was used to assign Gene Ontology (GO) terms to the annotated proteins. Briefly, a BLASTP search to the NR database was carried out, the accession numbers were mapped to the Gene Onotology database and only those with an e-value lower than 1e-6 were kept. For the statistical analysis of enriched GO terms a two-tailed Fisher Exact test was used.

### Comparative genomic

*Cryptosporidium hominis* TU502, “*C. hominis* TU502 new” and *C. hominis* UKH1 genome sequences represented by contigs were retrieved from cryptoDB database release 8, which is available at http://cryptodb.org/common/downloads/release-8.0/.

Large sequence polymorphisms (LSPs) larger than 30 nucleotides were identified from collinear blocks built by MAUVEALIGNER^49^ between *C. parvum* Iowa and *C. hominis* UdeA01 genomes. Flanking sequences of collinear blocks were extracted from each genome and then aligned to each other using MUSCLE^50^. Identification of single nucleotide polymorphisms (SNPs) was performed using the MUMMER^51^ show-snps tool, ignoring ambiguous mapped regions. Only SNPs located 30 nucleotides away from the nearest mismatch were reported in order to avoid SNPs over low complexity regions or located at doubtfully aligned regions.

MUTATION HUNTER wrapper was implemented to identify genes under positive, negative or neutral selective pressure^4^. Briefly, pairs of orthologous genes between *C. parvum Iowa* (IGA) and *C. hominis* UdeA01 were identified using INPARANOID version 4.1^52^, then aligned using FASTA36^53^ and finally dN/dS ratios were calculated using PAML^54^.

## Additional Information

**Accession codes:** Raw reads, assembly and annotation of *C. hominis* UdeA01 have been deposited in the European Nucleotide Archive (ENA) under the Bioproject ID PRJEB10000. RNA-seq raw data of *C. parvum* have been deposited in NCBI as Bioproject ID PRJNA291984.

**How to cite this article**: Isaza, J. P. *et al.* Revisiting the reference genomes of human pathogenic *Cryptosporidium species*: reannotation of *C. parvum* Iowa and a new *C. hominis* reference. *Sci. Rep.* **5**, 16324; doi: 10.1038/srep16324 (2015).

## Supplementary Material

Supplementary Information

## Figures and Tables

**Table 1:** Summary of general statistics for the genome re-annotation of *C. parvum* Iowa and the genome annotation of *C. hominis* UdeA01.

	*C. parvum* Iowa		*C. hominis* UdeA01
	OGA	IGA	
Number of genes	3,805	3,865	3,819
Mean gene length (bp) excluding introns	1,793	1,783	1,784
Percent coding	75	75.7	75.4
Genes with introns (%)	4.3	10.8	10.9
**exons**			
Number	4,042	4,553	4,503
Mean length (bp)	1,687	1,514	1,514
Mean number per gene*	2.5	2.6	2.6
G + C content %	32	32	32
**introns**			
Number	237	688	684
Mean length (bp)	84	99	100
G + C content %	22	22	22
**Intergenic regions**			
G + C content %	25.2	25	25.2
Mean length (bp)	589	552	563
tRNAs	45	45	45
**Annotaion**			
Genes with EC code	500	502	498
Number of unique EC code	207	207	206
Genes with GO terms	2,025	2,030	2,026
Number unique GO terms	958	967	968
Number of unique PFAM domains	1,946	1,969	1,982
Number of unique SCOP superfamilies	1,667	1,667	1,677

^*^Excluding intronless genes.

**Table 2:** Summary of genome assembly of *C. hominis* UdeA01.

Parameter	*De novo* assembly	Blast selected contigs
Total contigs	112,561	262
Contigs longer than 500 nt	54,337	124
Average contig size (nt)	2,279	72,994
Largest contig (nt)	787,208	579,377
N50	5,764	153,969
Total assembled bases (Mb)	141	9.05
coverage	—	68X

**Table 3:** Functional annotation of genes detected on *C. hominis* UdeA01 genome sequence which were absent in *C. parvum* Iowa.

Gene	Blastp	Pfam-A	Panther	SCOP	GO	E.C	Gene3d	SignalP-TM	SignalP-noTM	TMHMM
uda2_newUdeA_01	hypothetical protein	n.a	n.a	n.a	n.a	n.a	no	no	no	no
uda3_newUdeA_01	hypothetical protein	n.a	PTHR23213	n.a	n.a	n.a	n.a	no	yes	no
uda3_newUdeA_02	hypothetical protein	n.a	n.a	n.a	n.a	n.a	n.a	no	yes	no
uda4_newUdeA_01	DinB/family X-type DNA polymerase	PF00817	PTHR11076:SF11	SSF100879	GO:0003887	EC:2.7.7.7	G3DSA:1.10.150.20	no	no	no
uda4_newUdeA_02	40S ribosomal protein S11	PF00366.15	PTHR10744	SSF50249	GO:0005840	n.a	G3DSA:2.40.50.140	no	no	no
uda4_newUdeA_03	hypothetical protein	n.a	n.a	n.a	n.a	n.a	n.a	no	no	yes
uda5_newUdeA_02	hypothetical protein	n.a	n.a	n.a	n.a	n.a	n.a	no	yes	no
uda5_newUdeA_03	hypothetical protein	n.a	n.a	n.a	n.a	n.a	n.a	no	no	no
uda6_newUdeA_01	conserved hypothetical transmembrane protein	n.a	n.a	n.a	GO:0016021	n.a	n.a	no	no	yes
uda6_newUdeA_02	hypothetical protein	n.a	n.a	n.a	GO:0050660	n.a	G3DSA:3.20.20.70	no	no	no
uda6_newUdeA_03	hypothetical protein	PF13367.1	n.a	n.a	n.a	n.a	n.a	no	no	yes

**Table 4:** Summary of indel events detected between *C. parvum* Iowa and *C. hominis* UdeA01.

Chromosome	Insertions	Deletions	Largest insertion (nt)	Shortest insertion (nt)	Largest deletion (nt)	Shortest deletion (nt)
1	14	13	86	33	90	30
2	7	12	143	35	318	30
3	9	12	67	33	66	30
4	17	15	4,452	30	190	30
5	11	15	158	30	522	30
6	20	20	242	30	69	30
7	9	11	184	32	1,248	30
8	12	24	114	30	249	30
**total**	99	122	—	—	—	—
