# Supplementary material for: Revisiting the reference genomes of human pathogenic Cryptosporidium species: reannotation of C. parvum Iowa and a new C. hominis reference
Source: Sci Rep. 2015 Nov 9;5:16324. doi: 10.1038/srep16324 (PMC4637869; doi:10.1038/srep16324)
Supplement: Supplementary Information [file srep16324-s1.pdf]

Revisiting the reference genomes of human pathogenic *Cryptosporidium* species: reannotation of *C. parvum* Iowa and a new *C. hominis* reference.

Juan Pablo Isaza [1-2], Ana Luz Galvan [1], Victor Polanco[3], Bernice Huang [4], Andrey V. Matveyev [4], Myrna G. Serrano [4], Patricio Manque[3], Gregory A. Buck [4], Juan Fernando Alzate [1-2] *

1 Grupo de Parasitología, Universidad de Antioquia

2 Centro Nacional de Secuenciación Genómica - CNSG, Universidad de Antioquia

3 Universidad Mayor de Chile - Centro de Genómica y Bioinformatica

4 Virginia Commonwealth University – Center for the Study of Biological Complexity

*jfernando.alzate@udea.edu.co

Supplementary Table S1

Fourteen coding regions of original genome annotation that were actually exons belonging to flanking genes in the improved genome annotation

gene	cryptoDB annotation
cgd1_1440	hypothetical protein
cgd3_2290	possible domain AAA, ATpase family
cgd3_3540	hypothetical protein
cgd4_1290	small hypothetical protein, possible conserved
cgd4_4250	hypothetical protein
cgd4_4430	OTU like cysteine protease
cgd5_2970	hypothetical protein
cgd5_2980	hypothetical protein
cgd6_3320	hypothetical protein
cgd6_5020	protein with WD40 repeats
cgd7_1880	60S ribosomal protein L44
cgd7_510	RNA polymerase III subunit C11
cgd8_3800	hypothetical protein
cgd8_5320	Ser/Thr protein kinase

Supplementary Table S2

New PFAM-A domains detected in the IGA

PFAM accesion	PFAM description	gene
PF00163.14	Ribosomal protein S4/S9 N-terminal domain	cgd8_1840
PF00780.17	CNH domain	cgd7_170
PF01900.14	Rpp14/Pop5 family	cgd3_new_06 - cgd4_new_01
PF02453.12	Reticulon	cgd7_new_10
PF02845.11	CUE domain	cgd4_2360
PF03174.8	Chitobiase/beta-hexosaminidase C-terminal domain	cgd7_new_15
PF05160.8	DSS1/SEM1 family	cgd1_new_04
PF05486.7	Signal recognition particle 9 kDa protein (SRP9)	cgd7_new_03
PF05493.8	ATP synthase subunit H	cgd4_new_06
PF05603.7	Protein of unknown function (DUF775)	cgd6_340
PF06012.7	Domain of Unknown Function (DUF908)	cgd3_1180
PF06331.7	Transcription factor TFIIH complex subunit Tfb5	cgd6_new_06
PF06698.6	Protein of unknown function (DUF1192)	cgd2_1350
PF07890.7	Rrp15p	cgd3_new_08
PF07926.7	TPR/MLP1/MLP2-like protein	chd7_4630
PF08315.7	cwf18 pre-mRNA splicing factor	cgd7_new_05
PF08923.5	Mitogen-activated protein kinase kinase 1 interacting	cgd7_new_02
PF09420.5	Ribosome biogenesis protein Nop16	cgd1_new_01
PF09446.5	VMA21-like domain	cgd8_new_02
PF09805.4	Nucleolar protein 12 (25kDa)	cgd8_new_12
PF10247.4	Reactive mitochondrial oxygen species modulator 1	cgd8_2360
PF10253.4	Mitotic checkpoint regulator, MAD2B-interacting	cgd2_4310
PF10273.4	Pre-rRNA-processing protein TSR2	cgd6_new_04
PF10372.4	Bacterial membrane-spanning protein N-terminus	cgd7_530
PF10392.4	Golgi transport complex subunit 5	cgd3_1200
PF12542.3	Pre-mRNA splicing factor	cgd5_4020
PF12773.2	Double zinc ribbon	cgd7_505
PF13865.1	C-terminal duplication domain of Friend of PRMT1	cgd5_new_06
PF14517.1	Tachylectin	cgd8_3690
PF15490.1	Telomere-capping, CST complex subunit	cgd8_new_03

Supplementary Figure S3. Boxplot of reads percentage supporting intra isolate single nucleotide variants.

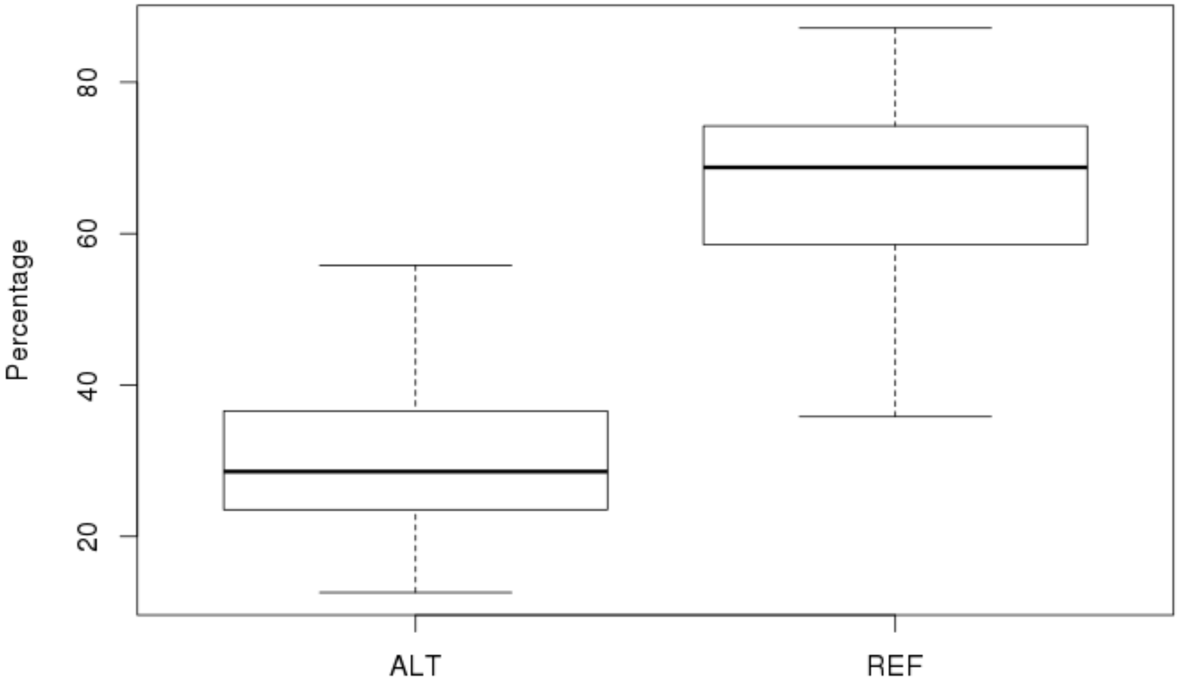

NGSTools package was implemented to discover intra isolate SNV using the module FindVariants. A total of 726 SNVs were found, which represent 0.008% of genome positions. REF refers to the allele reported in the genome (consensus from genome assembly) and ALT refers to the alternative allele reported by NGSTools. The median of reads percentage supporting REF and ALT alleles were 69%(median reads coverage=39) and 28% (median read coverage= 15.5) respectively.

Supplementary Table S4

Highly divergent proteins (identity lower than 90%) between *C. hominis* UdeA01 and *C. parvum Iowa.*

C. hominis UdeA01	*C. parvum Iowa*	identity
CHUDEA1_110	cgd1_110	0.867
CHUDEA1_120	cgd1_120	0.772
CHUDEA1_130	cgd1_130	0.808
CHUDEA1_2880	cgd1_2880	0.881
CHUDEA1_3850	cgd1_3850	0.786
CHUDEA1_470	cgd1_470	0.745
CHUDEA1_620	cgd1_620	0.886
CHUDEA1_680	cgd1_680	0.836
CHUDEA2_2550	cgd2_2550	0.879
CHUDEA2_2570	cgd2_2570	0.883
CHUDEA2_2900	cgd2_2900	0.877
CHUDEA2_3520	cgd2_3520	0.832
CHUDEA2_3530	cgd2_3530	0.786
CHUDEA2_390	cgd2_390	0.813
CHUDEA2_400	cgd2_400	0.825
CHUDEA2_4020	cgd2_4020	0.898
CHUDEA2_410	cgd2_410	0.829
CHUDEA2_420	cgd2_420	0.572
CHUDEA2_430	cgd2_430	0.709
CHUDEA2_4370	cgd2_4370	0.78
CHUDEA2_440	cgd2_440	0.785
CHUDEA2_450	cgd2_450	0.79
CHUDEA2_newUdeA_01	cgd5_4570	0.266
CHUDEA3_10	cgd3_10	0.833
CHUDEA3_1100	cgd3_1100	0.826
CHUDEA3_1150	cgd3_1150	0.702
CHUDEA3_1160	cgd3_1160	0.848
CHUDEA3_1690	cgd3_1690	0.867
CHUDEA3_1710	cgd3_1710	0.857
CHUDEA3_1730	cgd3_1730	0.879
CHUDEA3_1740	cgd3_1740	0.85
CHUDEA3_1750	cgd3_1750	0.887
CHUDEA3_1760	cgd3_1760	0.806
CHUDEA3_1770	cgd3_1770	0.755
CHUDEA3_1780	cgd3_1780	0.829
CHUDEA3_190	cgd3_190	0.779
CHUDEA3_4260	cgd3_4260	0.875
CHUDEA3_4270	cgd3_4270	0.895

CHUDEA3_820	cgd3_820	0.887
CHUDEA4_10	cgd4_10	0.869
CHUDEA4_1300	cgd4_1300	0.681
CHUDEA4_2510	cgd4_2510	0.895
CHUDEA4_31	cgd4_31	0.849
CHUDEA4_32	cgd4_32	0.899
CHUDEA4_3520	cgd4_3520	0.889
CHUDEA4_3550	cgd4_3550	0.881
CHUDEA4_3630	cgd4_3630	0.68
CHUDEA4_3640	cgd4_3640	0.88
CHUDEA4_3650	cgd4_3650	0.83
CHUDEA4_3670	cgd4_3670	0.736
CHUDEA4_3680	cgd4_3680	0.878
CHUDEA4_3690	cgd4_3690	0.717
CHUDEA4_3970	cgd4_3970	0.85
CHUDEA4_4470	cgd4_4470	0.884
CHUDEA4_4480	cgd4_4480	0.89
CHUDEA4_4500	cgd4_4500	0.769
CHUDEA4_770	cgd4_770	0.874
CHUDEA4_920	cgd4_920	0.747
CHUDEA4_990	cgd4_990	0.892
CHUDEA5_10	cgd5_10	0.87
CHUDEA5_1940	cgd5_1940	0.872
CHUDEA5_20	cgd5_20	0.889
CHUDEA5_2180	cgd5_2180	0.679
CHUDEA5_3000	cgd5_3000	0.82
CHUDEA5_30	cgd5_40	0.723
CHUDEA5_3490	cgd5_3490	0.899
CHUDEA5_40	cgd5_40	0.723
CHUDEA5_450	cgd5_450	0.89
CHUDEA5_50	cgd5_50	0.82
CHUDEA5_newUdeA_01	cgd6_5470	0.78
CHUDEA5_new_01	cgd5_new_01	0.833
CHUDEA6_1080	cgd6_1080	0.669
CHUDEA6_10	cgd6_10	0.471
CHUDEA6_3050	cgd6_3050	0.854
CHUDEA6_3930	cgd6_3930	0.819
CHUDEA6_40	cgd6_40	0.716
CHUDEA6_4230	cgd6_4230	0.89
CHUDEA6_4740	cgd6_4740	0.841
CHUDEA6_4980	cgd6_4980	0.881
CHUDEA6_5270	cgd6_5270	0.887
CHUDEA6_5400	cgd6_5400	0.765
CHUDEA6_5410	cgd6_5410	0.856
CHUDEA6_5430	cgd6_5430	0.794

CHUDEA6_5470	cgd6_5470	0.78
CHUDEA6_5490	cgd5_4600	0.767
CHUDEA6_5490	cgd5_4610	0.695
CHUDEA6_60	cgd6_60	0.881
CHUDEA7_10	cgd7_10	0.845
CHUDEA7_1210	cgd7_1210	0.888
CHUDEA7_1280	cgd7_1280	0.767
CHUDEA7_1370	cgd7_1370	0.891
CHUDEA7_1870	cgd7_1870	0.872
CHUDEA7_2120	cgd7_2120	0.885
CHUDEA7_4020	cgd7_4020	0.81
CHUDEA7_4430	cgd7_4430	0.833
CHUDEA7_4500	cgd7_4500	0.823
CHUDEA7_5510	cgd7_5510	0.891
CHUDEA8_1220	cgd8_1220	0.819
CHUDEA8_20	cgd8_20	0.874
CHUDEA8_2160	cgd8_2160	0.845
CHUDEA8_30	cgd8_30	0.885
CHUDEA8_3120	cgd8_3120	0.886
CHUDEA8_3550	cgd8_3550	0.761
CHUDEA8_40	cgd8_40	0.791
CHUDEA8_4190	cgd8_4180	0.71
CHUDEA8_520	cgd8_520	0.82
CHUDEA8_5290	cgd8_5290	0.894
CHUDEA8_5370	cgd8_5370	0.646
CHUDEA8_5380	cgd8_5380	0.754
CHUDEA8_5390	cgd8_5390	0.884
CHUDEA8_60	cgd8_60	0.791
CHUDEA8_660	cgd8_660	0.856
CHUDEA8_700	cgd8_700	0.869

Supplementary table S5

dN/dS analysis between orthologous genes of *C. parvum* Iowa and *C. hominis* UdeA01

C. hominis UdeA01	*C. parvum Iowa*	identity	dS	dN	dN/dS
CHUDEA1_1000	cgd1_1000	0.98	0.0486	0.0086	0.176954733
CHUDEA1_100	cgd1_100	0.972	0.0649	0.0125	0.192604006
CHUDEA1_1010	cgd1_1010	0.983	0.0655	0.0071	0.108396947
CHUDEA1_1020	cgd1_1020	0.969	0.029	0.0132	0.455172414
CHUDEA1_1030	cgd1_1030	0.97	0.0847	0.0172	0.203069658
CHUDEA1_1040	cgd1_1040	0.985	0.0806	0.0049	0.060794045
CHUDEA1_1050	cgd1_1050	0.972	0.0495	0.013	0.262626263
CHUDEA1_1060	cgd1_1060	0.979	0.0577	0.0088	0.152512998
CHUDEA1_1070	cgd1_1070	0.988	0.0598	0.0052	0.086956522
CHUDEA1_1080	cgd1_1080	0.996	0.1033	0.0015	0.014520813
CHUDEA1_1090	cgd1_1090	0.971	0.052	0.0127	0.244230769
CHUDEA1_10	cgd1_10	0.97	0.0555	0.0136	0.245045045
CHUDEA1_1100	cgd1_1100	0.998	0.0558	0.0008	0.014336918
CHUDEA1_110	cgd1_110	0.867	0.0992	0.0615	0.619959677
CHUDEA1_1110	cgd1_1110	0.971	0.0409	0.0115	0.281173594
CHUDEA1_1120	cgd1_1120	0.965	0.0543	0.0163	0.300184162
CHUDEA1_1130	cgd1_1130	0.987	0.0647	0.0053	0.081916538
CHUDEA1_1140	cgd1_1140	0.985	0.0724	0.0066	0.091160221
CHUDEA1_1150	cgd1_1150	0.937	0.0861	0.0283	0.328687573
CHUDEA1_1160	cgd1_1160	0.96	0.0285	0.0193	0.677192982
CHUDEA1_1170	cgd1_1170	0.995	0.0415	0.0022	0.053012048
CHUDEA1_1180	cgd1_1180	0.985	0.0623	0.0074	0.118780096
CHUDEA1_1190	cgd1_1190	0.976	0	0.0111	1000
CHUDEA1_1200	cgd1_1200	0.987	0.1106	0.0055	0.049728752
CHUDEA1_120	cgd1_120	0.772	0.1169	0.1163	0.994867408
CHUDEA1_1210	cgd1_1210	0.979	0.0921	0.0092	0.099891422
CHUDEA1_1220	cgd1_1220	0.983	0.0707	0.0078	0.110325318
CHUDEA1_1230	cgd1_1230	0.979	0.0561	0.0092	0.16399287
CHUDEA1_1240	cgd1_1240	0.993	0.0493	0.0029	0.058823529
CHUDEA1_1250	cgd1_1250	0.975	0.0642	0.0121	0.18847352
CHUDEA1_1260	cgd1_1260	0.965	0.0464	0.011	0.237068966
CHUDEA1_1270	cgd1_1270	0.997	0.0599	0.0012	0.020033389
CHUDEA1_1280	cgd1_1280	0.996	0.0481	0.0019	0.03950104
CHUDEA1_1290	cgd1_1290	0.989	0.0437	0.0051	0.116704805
CHUDEA1_1300	cgd1_1300	0.969	0.0365	0.0141	0.38630137
CHUDEA1_130	cgd1_130	0.808	0.1265	0.084	0.664031621
CHUDEA1_1310	cgd1_1310	0.99	0.0497	0.0041	0.08249497
CHUDEA1_1320	cgd1_1320	1	0.0964	0	0
CHUDEA1_1330	cgd1_1330	0.989	0.043	0.0059	0.137209302
CHUDEA1_1340	cgd1_1340	0.98	0.0599	0.0097	0.161936561

CHUDEA1_1350	cgd1_1350	0.981	0.0502	0.0097	0.193227092
CHUDEA1_1360	cgd1_1360	0.955	0.0683	0.0226	0.330893119
CHUDEA1_1370	cgd1_1370	0.989	0.0947	0.005	0.05279831
CHUDEA1_1380	cgd1_1380	0.98	0.0405	0.009	0.222222222
CHUDEA1_1390	cgd1_1390	0.963	0.0708	0.0166	0.234463277
CHUDEA1_1400	cgd1_1400	0.976	0.0475	0.0115	0.242105263
CHUDEA1_1410	cgd1_1410	0.988	0.0598	0.0052	0.086956522
CHUDEA1_1420	cgd1_1420	0.988	0.0628	0.0053	0.084394904
CHUDEA1_1430	cgd1_1430	0.946	0.0717	0.0246	0.343096234
CHUDEA1_1450	cgd1_1450	0.949	0.0541	0.0237	0.438077634
CHUDEA1_1460	cgd1_1460	0.957	0.0592	0.0196	0.331081081
CHUDEA1_1470	cgd1_1470	0.98	0.0744	0.0087	0.116935484
CHUDEA1_1480	cgd1_1480	0.965	0.0486	0.0159	0.327160494
CHUDEA1_1490	cgd1_1490	0.98	0.0601	0.0097	0.161397671
CHUDEA1_1500	cgd1_1500	0.976	0.0548	0.0115	0.209854015
CHUDEA1_150	cgd1_150	0.926	0.0658	0.0306	0.465045593
CHUDEA1_1510	cgd1_1510	0.989	0.0436	0.0047	0.107798165
CHUDEA1_1530	cgd1_1530	0.975	0.0404	0.0113	0.27970297
CHUDEA1_1540	cgd1_1540	0.99	0.0584	0.0051	0.087328767
CHUDEA1_1550	cgd1_1550	0.991	0.0528	0.0042	0.079545455
CHUDEA1_1560	cgd1_1560	0.987	0.0761	0.0056	0.073587385
CHUDEA1_1570	cgd1_1570	0.955	0.0616	0.0121	0.196428571
CHUDEA1_1580	cgd1_1580	0.986	0.0603	0.0065	0.107794362
CHUDEA1_1590	cgd1_1590	0.99	0.0517	0.0046	0.088974855
CHUDEA1_1600	cgd1_1600	0.983	0.0479	0.0076	0.158663883
CHUDEA1_160	cgd1_160	0.928	0.0588	0.026	0.442176871
CHUDEA1_1610	cgd1_1610	0.963	0.041	0.0161	0.392682927
CHUDEA1_1620	cgd1_1620	0.961	0.0442	0.0175	0.395927602
CHUDEA1_1630	cgd1_1630	0.921	0.0599	0.0362	0.604340568
CHUDEA1_1640	cgd1_1640	0.964	0.0668	0.0146	0.218562874
CHUDEA1_1650	cgd1_1650	0.992	0.0495	0.0032	0.064646465
CHUDEA1_1660	cgd1_1660	1	0.0373	0	0
CHUDEA1_1670	cgd1_1670	0.965	0.075	0.0147	0.196
CHUDEA1_1680	cgd1_1680	0.972	0.0614	0.0109	0.17752443
CHUDEA1_1690	cgd1_1690	0.967	0.0466	0.0149	0.319742489
CHUDEA1_1700	cgd1_1700	0.981	0.0515	0.0082	0.159223301
CHUDEA1_170	cgd1_170	0.993	0.0508	0.0031	0.061023622
CHUDEA1_1710	cgd1_1710	0.981	0.0554	0.0084	0.151624549
CHUDEA1_1720	cgd1_1720	0.97	0.0523	0.0139	0.265774379
CHUDEA1_1730	cgd1_1730	0.93	0.064	0.0374	0.584375
CHUDEA1_1740	cgd1_1740	0.95	0.0304	0.0261	0.858552632
CHUDEA1_1750	cgd1_1750	0.965	0.0393	0.0159	0.404580153
CHUDEA1_1760	cgd1_1760	0.971	0.0416	0.0114	0.274038462
CHUDEA1_1770	cgd1_1770	0.976	0.0938	0.0115	0.122601279
CHUDEA1_1780	cgd1_1780	0.97	0.0548	0.0139	0.253649635

CHUDEA1_1790	cgd1_1790	0.964	0.0506	0.0155	0.306324111
CHUDEA1_1800	cgd1_1800	0.978	0.0642	0.0089	0.138629283
CHUDEA1_180	cgd1_180	0.962	0.0486	0.0109	0.224279835
CHUDEA1_1810	cgd1_1810	0.926	0.0946	0.031	0.32769556
CHUDEA1_1820	cgd1_1820	0.984	0.0571	0.007	0.122591944
CHUDEA1_1830	cgd1_1830	0.985	0.0425	0.0064	0.150588235
CHUDEA1_1840	cgd1_1840	0.962	0.0435	0.0175	0.402298851
CHUDEA1_1850	cgd1_1850	0.966	0.0647	0.0145	0.224111283
CHUDEA1_1860	cgd1_1860	0.981	0.0492	0.0083	0.168699187
CHUDEA1_1870	cgd1_1870	0.975	0.0697	0.0097	0.139167862
CHUDEA1_1880	cgd1_1880	0.956	0.0564	0.0212	0.375886525
CHUDEA1_1890	cgd1_1890	0.991	0.0417	0.004	0.095923261
CHUDEA1_1900	cgd1_1900	0.996	0.0557	0.0018	0.032315978
CHUDEA1_190	cgd1_190	0.98	0.0874	0.0086	0.098398169
CHUDEA1_1910	cgd1_1910	0.973	0.0592	0.0122	0.206081081
CHUDEA1_1920	cgd1_1920	0.963	0.059	0.0164	0.277966102
CHUDEA1_1930	cgd1_1930	0.982	0.0674	0.0077	0.114243323
CHUDEA1_1940	cgd1_1940	0.968	0.0458	0.0145	0.316593886
CHUDEA1_1950	cgd1_1950	0.995	0.0592	0.0021	0.035472973
CHUDEA1_1960	cgd1_1960	0.985	0.0613	0.0065	0.106035889
CHUDEA1_1970	cgd1_1970	1	0.0643	0	0
CHUDEA1_1980	cgd1_1980	0.968	0.0632	0.0148	0.234177215
CHUDEA1_1990	cgd1_1990	0.976	0.0669	0.0099	0.147982063
CHUDEA1_2000	cgd1_2000	0.944	0.0485	0.0274	0.564948454
CHUDEA1_200	cgd1_200	0.989	0.0535	0.0046	0.085981308
CHUDEA1_2010	cgd1_2010	0.972	0.0496	0.0118	0.237903226
CHUDEA1_2020	cgd1_2020	0.99	0.0713	0.006	0.084151473
CHUDEA1_2030	cgd1_2030	0.985	0.0544	0.007	0.128676471
CHUDEA1_2040	cgd1_2040	0.998	0.0931	0.0009	0.009667025
CHUDEA1_2050	cgd1_2050	0.957	0.052	0.0193	0.371153846
CHUDEA1_2060	cgd1_2060	0.989	0.0261	0.0046	0.176245211
CHUDEA1_2070	cgd1_2070	0.98	0.0576	0.009	0.15625
CHUDEA1_2080	cgd1_2080	0.961	0.0423	0.0185	0.437352246
CHUDEA1_2090	cgd1_2090	0.965	0.0471	0.0159	0.337579618
CHUDEA1_20	cgd1_20	0.97	0.0433	0.0141	0.325635104
CHUDEA1_2100	cgd1_2100	0.968	0.0506	0.0151	0.298418972
CHUDEA1_210	cgd1_210	0.952	0.0448	0.0215	0.479910714
CHUDEA1_2110	cgd1_2110	0.965	0.0619	0.0158	0.255250404
CHUDEA1_2120	cgd1_2120	0.957	0.0505	0.0202	0.4
CHUDEA1_2130	cgd1_2130	0.992	0.0765	0.0035	0.045751634
CHUDEA1_2140	cgd1_2140	0.911	0.0824	0.0399	0.484223301
CHUDEA1_2150	cgd1_2150	0.99	0.0688	0.0045	0.065406977
CHUDEA1_2160	cgd1_2160	0.991	0.079	0.004	0.050632911
CHUDEA1_2170	cgd1_2170	0.973	0.0388	0.0118	0.304123711
CHUDEA1_2180	cgd1_2180	0.943	0.0288	0.0279	0.96875

CHUDEA1_2190	cgd1_2190	0.985	0.06	0.0069	0.115
CHUDEA1_2200	cgd1_2200	0.952	0.0486	0.0207	0.425925926
CHUDEA1_220	cgd1_220	0.969	0.0462	0.0137	0.296536797
CHUDEA1_2210	cgd1_2210	0.958	0.1559	0.0179	0.114817191
CHUDEA1_2220	cgd1_2220	0.972	0.0648	0.0132	0.203703704
CHUDEA1_2230	cgd1_2230	0.985	0.0919	0.0065	0.070729053
CHUDEA1_2240	cgd1_2240	0.951	0.0692	0.0214	0.309248555
CHUDEA1_2250	cgd1_2250	1	0.0574	0	0
CHUDEA1_2260	cgd1_2260	1	0.0491	0	0
CHUDEA1_2270	cgd1_2270	1	0.0119	0	0
CHUDEA1_2280	cgd1_2280	0.935	0.0649	0.0287	0.442218798
CHUDEA1_2290	cgd1_2290	0.986	0.0518	0.0047	0.090733591
CHUDEA1_2300	cgd1_2300	0.956	0.0582	0.0198	0.340206186
CHUDEA1_230	cgd1_230	0.991	0.0426	0.0036	0.084507042
CHUDEA1_2310	cgd1_2310	0.981	0.0675	0.009	0.133333333
CHUDEA1_2320	cgd1_2320	0.984	0.0607	0.0076	0.125205931
CHUDEA1_2330	cgd1_2330	0.982	0.0703	0.0073	0.103840683
CHUDEA1_2340	cgd1_2340	0.989	0.0494	0.0051	0.103238866
CHUDEA1_2350	cgd1_2350	0.95	0.0542	0.0219	0.404059041
CHUDEA1_2360	cgd1_2360	0.997	0.0337	0.0014	0.041543027
CHUDEA1_2370	cgd1_2370	0.972	0.037	0.0127	0.343243243
CHUDEA1_2380	cgd1_2380	0.966	0.0741	0.016	0.215924426
CHUDEA1_2390	cgd1_2390	0.977	0.0252	0.0102	0.404761905
CHUDEA1_2400	cgd1_2400	0.914	0.0835	0.0387	0.463473054
CHUDEA1_240	cgd1_240	0.977	0.1071	0.0099	0.092436975
CHUDEA1_2410	cgd1_2410	0.969	0.0334	0.0148	0.443113772
CHUDEA1_2420	cgd1_2420	0.969	0.0509	0.0135	0.265225933
CHUDEA1_2430	cgd1_2430	0.99	0.0791	0.0042	0.053097345
CHUDEA1_2440	cgd1_2440	0.963	0.0538	0.0167	0.310408922
CHUDEA1_2450	cgd1_2450	0.983	0.0621	0.0073	0.117552335
CHUDEA1_2460	cgd1_2460	0.979	0.0777	0.0093	0.11969112
CHUDEA1_2470	cgd1_2470	0.991	0.0868	0.0042	0.048387097
CHUDEA1_2480	cgd1_2480	0.972	0.0627	0.0115	0.183413078
CHUDEA1_2490	cgd1_2490	0.988	0.0824	0.0052	0.063106796
CHUDEA1_2500	cgd1_2500	0.967	0.0474	0.0153	0.32278481
CHUDEA1_250	cgd1_250	0.99	0.0812	0.0043	0.052955665
CHUDEA1_2510	cgd1_2510	0.986	0.0648	0.0064	0.098765432
CHUDEA1_2520	cgd1_2520	0.953	0.1003	0.0191	0.190428714
CHUDEA1_2530	cgd1_2530	0.976	0.0512	0.0104	0.203125
CHUDEA1_2540	cgd1_2540	0.977	0.0514	0.0099	0.192607004
CHUDEA1_2550	cgd1_2550	0.978	0.0522	0.0097	0.185823755
CHUDEA1_2560	cgd1_2560	0.984	0.0587	0.0071	0.120954003
CHUDEA1_2570	cgd1_2570	0.978	0.0627	0.0096	0.153110048
CHUDEA1_2580	cgd1_2580	0.984	0.0504	0.007	0.138888889
CHUDEA1_2590	cgd1_2590	0.984	0.0822	0.0086	0.104622871

CHUDEA1_2600	cgd1_2600	0.973	0.0599	0.0118	0.196994992
CHUDEA1_260	cgd1_260	0.954	0.0461	0.0198	0.429501085
CHUDEA1_2610	cgd1_2610	0.952	0.0469	0.0224	0.47761194
CHUDEA1_2620	cgd1_2620	0.998	0.0741	0.0009	0.012145749
CHUDEA1_2630	cgd1_2630	0.976	0.055	0.0105	0.190909091
CHUDEA1_2640	cgd1_2640	0.969	0.0957	0.0137	0.143155695
CHUDEA1_2650	cgd1_2650	0.986	0.0446	0.006	0.134529148
CHUDEA1_2660	cgd1_2660	1	0.0687	0	0
CHUDEA1_2670	cgd1_2670	0.984	0.0718	0.0071	0.098885794
CHUDEA1_2680	cgd1_2680	0.99	0.0728	0.0045	0.061813187
CHUDEA1_2690	cgd1_2690	0.99	0.0436	0.0046	0.105504587
CHUDEA1_2700	cgd1_2700	0.988	0.066	0.0052	0.078787879
CHUDEA1_270	cgd1_270	0.982	0.0374	0.008	0.213903743
CHUDEA1_2710	cgd1_2710	0.992	0.0518	0.0035	0.067567568
CHUDEA1_2720	cgd1_2720	0.987	0.0462	0.0055	0.119047619
CHUDEA1_2730	cgd1_2730	0.994	0.0342	0.0026	0.076023392
CHUDEA1_2740	cgd1_2740	0.983	0.0667	0.0079	0.11844078
CHUDEA1_2750	cgd1_2750	0.973	0.0643	0.0113	0.175738725
CHUDEA1_2760	cgd1_2760	0.997	0.0508	0.0012	0.023622047
CHUDEA1_2770	cgd1_2770	0.995	0.0728	0.0024	0.032967033
CHUDEA1_2780	cgd1_2780	0.976	0.0981	0.0107	0.109072375
CHUDEA1_2790	cgd1_2790	0.972	0.0882	0.0122	0.138321995
CHUDEA1_2800	cgd1_2800	0.977	0.0454	0.0105	0.231277533
CHUDEA1_280	cgd1_280	0.981	0.0415	0.0085	0.204819277
CHUDEA1_2810	cgd1_2810	0.981	0.0741	0.0081	0.109311741
CHUDEA1_2830	cgd1_2830	0.994	0.073	0.0014	0.019178082
CHUDEA1_2840	cgd1_2840	0.979	0.0666	0.0093	0.13963964
CHUDEA1_2850	cgd1_2850	0.969	0.0861	0.0139	0.161440186
CHUDEA1_2860	cgd1_2860	0.986	0.0493	0.0043	0.087221095
CHUDEA1_2870	cgd1_2870	0.962	0.0674	0.0175	0.259643917
CHUDEA1_2880	cgd1_2880	0.881	0.0168	0.0595	3.541666667
CHUDEA1_2890	cgd1_2890	0.973	0.0551	0.012	0.217785844
CHUDEA1_2900	cgd1_2900	0.978	0.0732	0.0103	0.140710383
CHUDEA1_290	cgd1_290	0.994	0.065	0.0027	0.041538462
CHUDEA1_2910	cgd1_2910	0.971	0.0517	0.012	0.232108317
CHUDEA1_2920	cgd1_2920	0.995	0.0245	0.002	0.081632653
CHUDEA1_2930	cgd1_2930	0.985	0.0648	0.0063	0.097222222
CHUDEA1_2940	cgd1_2940	0.973	0.0387	0.0115	0.297157623
CHUDEA1_2950	cgd1_2950	0.981	0.0579	0.0084	0.14507772
CHUDEA1_2960	cgd1_2960	0.993	0.0436	0.0031	0.071100917
CHUDEA1_2970	cgd1_2970	0.995	0.0919	0.0011	0.011969532
CHUDEA1_2980	cgd1_2980	0.983	0.0566	0.007	0.123674912
CHUDEA1_2990	cgd1_2990	0.967	0.027	0.0141	0.522222222
CHUDEA1_3000	cgd1_3000	0.993	0.0244	0.0032	0.131147541
CHUDEA1_300	cgd1_300	1	0.0667	0	0

CHUDEA1_3010	cgd1_3010	0.996	0.0653	0.0016	0.024502297
CHUDEA1_3020	cgd1_3020	0.989	0.0418	0.005	0.119617225
CHUDEA1_3030	cgd1_3030	0.99	0.05	0.0044	0.088
CHUDEA1_3040	cgd1_3040	0.984	0.0649	0.0072	0.110939908
CHUDEA1_3050	cgd1_3050	0.983	0.0545	0.0075	0.137614679
CHUDEA1_3060	cgd1_3060	0.946	0.0483	0.0103	0.213250518
CHUDEA1_3070	cgd1_3070	0.959	0.056	0.015	0.267857143
CHUDEA1_3080	cgd1_3080	0.979	0.0594	0.0085	0.143097643
CHUDEA1_3090	cgd1_3090	0.956	0.0634	0.0165	0.260252366
CHUDEA1_30	cgd1_30	0.982	0.0492	0.0079	0.160569106
CHUDEA1_3100	cgd1_3100	0.977	0.0463	0.0101	0.218142549
CHUDEA1_310	cgd1_310	0.991	0.0519	0.0036	0.069364162
CHUDEA1_3110	cgd1_3110	0.975	0.0417	0.0117	0.28057554
CHUDEA1_3120	cgd1_3120	0.983	0.0464	0.0074	0.159482759
CHUDEA1_3130	cgd1_3130	0.984	0.0346	0.0073	0.210982659
CHUDEA1_3140	cgd1_3140	0.98	0.1123	0.0082	0.0730187
CHUDEA1_3150	cgd1_3150	0.988	0.0709	0.0049	0.069111425
CHUDEA1_3160	cgd1_3160	0.947	0.068	0.0162	0.238235294
CHUDEA1_3170	cgd1_3170	0.949	0.0658	0.0121	0.183890578
CHUDEA1_3180	cgd1_3180	0.969	0.0342	0.0136	0.397660819
CHUDEA1_3190	cgd1_3190	0.973	0.0884	0.0109	0.123303167
CHUDEA1_3200	cgd1_3200	0.976	0.0508	0.0098	0.192913386
CHUDEA1_320	cgd1_320	0.983	0.0464	0.0071	0.153017241
CHUDEA1_3210	cgd1_3210	0.983	0.0674	0.0057	0.084569733
CHUDEA1_3220	cgd1_3220	0.957	0.0486	0.0146	0.300411523
CHUDEA1_3230	cgd1_3230	0.924	0.0813	0.0185	0.227552276
CHUDEA1_3240	cgd1_3240	0.974	0.0544	0.0085	0.15625
CHUDEA1_3250	cgd1_3250	0.938	0.0949	0.0157	0.165437302
CHUDEA1_3260	cgd1_3260	0.945	0.0728	0.0189	0.259615385
CHUDEA1_3270	cgd1_3270	0.977	0.0795	0.0025	0.031446541
CHUDEA1_3280	cgd1_3280	0.929	0.0672	0.0176	0.261904762
CHUDEA1_3290	cgd1_3290	0.903	0.0908	0.0282	0.310572687
CHUDEA1_3300	cgd1_3300	0.997	0.1107	0.0013	0.011743451
CHUDEA1_330	cgd1_330	0.996	0.0638	0.0025	0.039184953
CHUDEA1_3310	cgd1_3310	0.991	0.0349	0.0034	0.097421203
CHUDEA1_3320	cgd1_3320	0.968	0.0402	0.0126	0.313432836
CHUDEA1_3330	cgd1_3330	0.979	0.0766	0.0089	0.11618799
CHUDEA1_3340	cgd1_3340	0.993	0.0534	0.0026	0.048689139
CHUDEA1_3350	cgd1_3350	0.988	0.1356	0.0045	0.033185841
CHUDEA1_3360	cgd1_3360	0.985	0.0557	0.006	0.107719928
CHUDEA1_3370	cgd1_3370	0.948	0.1021	0.006	0.058765916
CHUDEA1_3380	cgd1_3380	0.993	0.0622	0.0031	0.049839228
CHUDEA1_3390	cgd1_3390	0.993	0.0555	0.0009	0.016216216
CHUDEA1_3400	cgd1_3400	0.966	0.0806	0.0083	0.102977667
CHUDEA1_340	cgd1_340	0.995	0.0368	0.0023	0.0625

CHUDEA1_3410	cgd1_3410	1	0.0499	0	0
CHUDEA1_3420	cgd1_3420	0.973	0.0583	0.0076	0.130360206
CHUDEA1_3430	cgd1_3430	0.924	0.027	0.0406	1.503703704
CHUDEA1_3440	cgd1_3440	0.995	0.0565	0.0019	0.033628319
CHUDEA1_3450	cgd1_3450	0.944	0.1168	0.0184	0.157534247
CHUDEA1_3460	cgd1_3460	0.986	0.1197	0.0052	0.043441938
CHUDEA1_3470	cgd1_3470	0.984	0.1012	0.0064	0.063241107
CHUDEA1_3480	cgd1_3480	0.982	0.0394	0.0055	0.139593909
CHUDEA1_3490	cgd1_3490	0.971	0.0595	0.0113	0.189915966
CHUDEA1_3500	cgd1_3500	0.961	0.0771	0.0148	0.191958495
CHUDEA1_350	cgd1_350	0.977	0.0522	0.01	0.191570881
CHUDEA1_3510	cgd1_3510	0.921	0.1079	0.0414	0.383688601
CHUDEA1_3520	cgd1_3520	0.984	0.0763	0.0066	0.086500655
CHUDEA1_3530	cgd1_3530	1	0.0078	0	0
CHUDEA1_3540	cgd1_3540	0.969	0.0544	0.0139	0.255514706
CHUDEA1_3550	cgd1_3550	0.966	0.1408	0.0173	0.122869318
CHUDEA1_3560	cgd1_3560	0.976	0.0266	0.0089	0.334586466
CHUDEA1_3570	cgd1_3570	0.989	0.1496	0.0028	0.018716578
CHUDEA1_3580	cgd1_3580	0.917	0.1153	0.0285	0.247181266
CHUDEA1_3590	cgd1_3590	0.955	0.0631	0.02	0.316957211
CHUDEA1_3600	cgd1_3600	0.972	0.0602	0.0119	0.197674419
CHUDEA1_360	cgd1_360	0.976	0.0469	0.0121	0.257995736
CHUDEA1_3610	cgd1_3610	0.956	0.0892	0.0181	0.202914798
CHUDEA1_3620	cgd1_3620	0.984	0.0639	0.0069	0.107981221
CHUDEA1_3630	cgd1_3630	0.96	0.0636	0.015	0.235849057
CHUDEA1_3640	cgd1_3640	0.968	0.0878	0.0124	0.141230068
CHUDEA1_3650	cgd1_3650	0.937	0.065	0.0303	0.466153846
CHUDEA1_3660	cgd1_3660	0.98	0.0811	0.0083	0.102342787
CHUDEA1_3670	cgd1_3670	0.961	0.0632	0.0103	0.162974684
CHUDEA1_3690	cgd1_3690	0.971	0.0769	0.0111	0.144343303
CHUDEA1_3700	cgd1_3700	0.998	0.066	0.001	0.015151515
CHUDEA1_370	cgd1_370	0.975	0.073	0.0115	0.157534247
CHUDEA1_3710	cgd1_3710	0.99	0.0724	0.0047	0.064917127
CHUDEA1_3720	cgd1_3720	0.988	0.0594	0.0053	0.089225589
CHUDEA1_3730	cgd1_3730	0.99	0.078	0.004	0.051282051
CHUDEA1_3740	cgd1_3740	0.984	0.0557	0.0073	0.131059246
CHUDEA1_3750	cgd1_3750	0.996	0.068	0.0017	0.025
CHUDEA1_3760	cgd1_3760	0.955	0.052	0.0181	0.348076923
CHUDEA1_3770	cgd1_3770	0.99	0.0381	0.0046	0.120734908
CHUDEA1_3780	cgd1_3780	0.93	0.0633	0.0329	0.519747235
CHUDEA1_3790	cgd1_3790	0.954	0.059	0.0191	0.323728814
CHUDEA1_3800	cgd1_3800	1	0.0636	0	0
CHUDEA1_380	cgd1_380	0.987	0.0555	0.0058	0.104504505
CHUDEA1_3810	cgd1_3810	0.919	0.1229	0.0388	0.315703824
CHUDEA1_3820	cgd1_3820	0.969	0.0561	0.0139	0.247771836

CHUDEA1_3830	cgd1_3830	0.903	0.0423	0.0484	1.144208038
CHUDEA1_3840	cgd1_3840	0.968	0.0789	0.0143	0.181242079
CHUDEA1_3850	cgd1_3850	0.786	0.1619	0.1109	0.684990735
CHUDEA1_3860	cgd1_3860	0.939	0.1269	0.0257	0.202521671
CHUDEA1_390	cgd1_390	1	0.043	0	0
CHUDEA1_400	cgd1_400	0.97	0.0792	0.0122	0.154040404
CHUDEA1_40	cgd1_40	0.988	0.0626	0.0054	0.086261981
CHUDEA1_410	cgd1_410	1	0.0655	0	0
CHUDEA1_420	cgd1_420	0.995	0.084	0.0021	0.025
CHUDEA1_430	cgd1_430	0.992	0.042	0.0032	0.076190476
CHUDEA1_440	cgd1_440	1	0.0481	0	0
CHUDEA1_450	cgd1_450	0.96	0.0422	0.013	0.308056872
CHUDEA1_460	cgd1_460	0.942	0.061	0.0191	0.313114754
CHUDEA1_470	cgd1_470	0.745	0.1762	0.1029	0.58399546
CHUDEA1_480	cgd1_480	0.945	0.0632	0.0265	0.419303797
CHUDEA1_490	cgd1_490	0.992	0.0884	0.0032	0.036199095
CHUDEA1_500	cgd1_500	0.984	0.0958	0.0067	0.06993737
CHUDEA1_50	cgd1_50	0.99	0.0801	0.0045	0.056179775
CHUDEA1_510	cgd1_510	0.981	0.0677	0.0077	0.113737075
CHUDEA1_520	cgd1_520	1	0.0554	0	0
CHUDEA1_530	cgd1_530	1	0.044	0	0
CHUDEA1_540	cgd1_540	0.994	0.0542	0.0024	0.044280443
CHUDEA1_550	cgd1_550	0.972	0.0602	0.012	0.199335548
CHUDEA1_560	cgd1_560	0.972	0.0792	0.0119	0.150252525
CHUDEA1_570	cgd1_570	0.983	0.0852	0.0073	0.085680751
CHUDEA1_580	cgd1_580	0.995	0.092	0.0021	0.022826087
CHUDEA1_600	cgd1_600	0.987	0.0596	0.0057	0.095637584
CHUDEA1_60	cgd1_60	0.987	0.0997	0.0041	0.04112337
CHUDEA1_610	cgd1_610	0.987	0.027	0.0073	0.27037037
CHUDEA1_620	cgd1_620	0.886	0.0736	0.0538	0.730978261
CHUDEA1_630	cgd1_630	0.997	0.0707	0.0014	0.01980198
CHUDEA1_640	cgd1_640	0.95	0.0721	0.023	0.319001387
CHUDEA1_650	cgd1_650	0.962	0.0687	0.0161	0.234352256
CHUDEA1_660	cgd1_660	0.985	0.0643	0.0064	0.099533437
CHUDEA1_670	cgd1_670	0.98	0.0766	0.0085	0.110966057
CHUDEA1_680	cgd1_680	0.836	0.083	0.0314	0.378313253
CHUDEA1_690	cgd1_690	0.97	0.0873	0.0081	0.092783505
CHUDEA1_700	cgd1_700	0.973	0.069	0.0102	0.147826087
CHUDEA1_70	cgd1_70	1	0.0473	0	0
CHUDEA1_710	cgd1_710	0.984	0.052	0.0071	0.136538462
CHUDEA1_720	cgd1_720	0.976	0.1181	0.0095	0.080440305
CHUDEA1_730	cgd1_730	0.983	0.0911	0.0068	0.074643249
CHUDEA1_740	cgd1_740	0.989	0.0661	0.0045	0.068078669
CHUDEA1_750	cgd1_750	0.989	0.0725	0.0033	0.045517241
CHUDEA1_760	cgd1_760	0.963	0.0698	0.0158	0.226361032

CHUDEA1_770	cgd1_770	0.984	0.0391	0.009	0.230179028
CHUDEA1_780	cgd1_780	0.981	0.041	0.0086	0.209756098
CHUDEA1_790	cgd1_790	0.973	0.0617	0.0115	0.186385737
CHUDEA1_800	cgd1_800	0.959	0.0406	0.015	0.369458128
CHUDEA1_80	cgd1_80	0.966	0.103	0.0147	0.142718447
CHUDEA1_810	cgd1_810	0.992	0.0581	0.0034	0.058519793
CHUDEA1_820	cgd1_820	0.992	0.0479	0.0035	0.073068894
CHUDEA1_830	cgd1_830	0.985	0.0656	0.0067	0.102134146
CHUDEA1_840	cgd1_840	1	0.0272	0	0
CHUDEA1_850	cgd1_850	0.993	0.0092	0.0029	0.315217391
CHUDEA1_860	cgd1_860	0.986	0.0778	0.0061	0.07840617
CHUDEA1_870	cgd1_870	0.986	0.0607	0.0061	0.100494234
CHUDEA1_880	cgd1_880	1	0.0644	0	0
CHUDEA1_890	cgd1_890	0.975	0.064	0.0109	0.1703125
CHUDEA1_90	cgd1_90	0.96	0.0386	0.0171	0.443005181
CHUDEA1_910	cgd1_910	0.99	0.0681	0.0045	0.066079295
CHUDEA1_920	cgd1_920	0.997	0.0627	0.001	0.015948963
CHUDEA1_930	cgd1_930	0.99	0.0652	0.0031	0.047546012
CHUDEA1_940	cgd1_940	0.994	0	0.0025	1000
CHUDEA1_950	cgd1_950	0.99	0.0608	0.0044	0.072368421
CHUDEA1_960	cgd1_960	0.941	0.088	0.0164	0.186363636
CHUDEA1_970	cgd1_970	0.942	0.067	0.0168	0.250746269
CHUDEA1_980	cgd1_980	0.998	0.0666	0.0007	0.010510511
CHUDEA1_990	cgd1_990	0.987	0.0506	0.0056	0.110671937
CHUDEA1_new_01	cgd1_new_01	0.985	0.0784	0.0068	0.086734694
CHUDEA1_new_02	cgd1_new_02	1	0.0784	0	0
CHUDEA1_new_03	cgd1_new_03	0.994	0.0574	0.0027	0.047038328
CHUDEA1_new_04	cgd1_new_04	1	0.0628	0	0
CHUDEA1_new_05	cgd1_new_05	0.994	0.0526	0.0025	0.047528517
CHUDEA1_new_06	cgd1_new_06	0.98	0.0848	0.0087	0.10259434
CHUDEA2_1000	cgd2_1000	0.986	0.0344	0.0038	0.110465116
CHUDEA2_100	cgd2_100	0.979	0.1136	0.0082	0.072183099
CHUDEA2_1010	cgd2_1010	0.995	0.0221	0.0023	0.104072398
CHUDEA2_1020	cgd2_1020	0.986	0.0495	0.0066	0.133333333
CHUDEA2_1030	cgd2_1030	0.994	0.0732	0.0027	0.036885246
CHUDEA2_1040	cgd2_1040	0.99	0.063	0.0046	0.073015873
CHUDEA2_1050	cgd2_1050	0.954	0.0326	0.022	0.674846626
CHUDEA2_1060	cgd2_1060	0.985	0.0449	0.0065	0.144766147
CHUDEA2_1070	cgd2_1070	1	0.0488	0	0
CHUDEA2_1080	cgd2_1080	0.957	0.0461	0.0199	0.431670282
CHUDEA2_1090	cgd2_1090	0.998	0.0381	0.0009	0.023622047
CHUDEA2_10	cgd2_470	0.936	0.0617	0.0185	0.299837925
CHUDEA2_1100	cgd2_1100	0.997	0.0561	0.0014	0.024955437
CHUDEA2_110	cgd2_110	0.991	0.0972	0.0036	0.037037037
CHUDEA2_1110	cgd2_1110	0.972	0.0637	0.0126	0.197802198

CHUDEA2_1120	cgd2_1120	0.99	0.0433	0.0044	0.101616628
CHUDEA2_1130	cgd2_1130	0.941	0.0466	0.0274	0.587982833
CHUDEA2_1140	cgd2_1140	0.994	0.0561	0.004	0.071301248
CHUDEA2_1150	cgd2_1150	0.997	0.0349	0.0013	0.037249284
CHUDEA2_1160	cgd2_1160	0.988	0.0596	0.0046	0.077181208
CHUDEA2_1170	cgd2_1170	0.998	0.0627	0.001	0.015948963
CHUDEA2_1190	cgd2_1190	0.955	0.0683	0.0196	0.286969253
CHUDEA2_1200	cgd2_1200	0.987	0.042	0.0057	0.135714286
CHUDEA2_120	cgd2_120	0.985	0.021	0.0132	0.628571429
CHUDEA2_1210	cgd2_1210	0.976	0.0492	0.0098	0.199186992
CHUDEA2_1220	cgd2_1220	0.981	0.0345	0.0086	0.249275362
CHUDEA2_1230	cgd2_1230	0.985	0.0627	0.0063	0.100478469
CHUDEA2_1240	cgd2_1240	0.974	0.031	0.0113	0.364516129
CHUDEA2_1250	cgd2_1250	0.985	0.0529	0.0066	0.124763705
CHUDEA2_1260	cgd2_1260	0.985	0.0361	0.0066	0.182825485
CHUDEA2_1270	cgd2_1270	0.984	0.0371	0.007	0.188679245
CHUDEA2_1280	cgd2_1280	0.985	0.0617	0.0067	0.108589951
CHUDEA2_1290	cgd2_1290	0.976	0.0403	0.0098	0.243176179
CHUDEA2_1300	cgd2_1300	0.982	0.0536	0.0083	0.154850746
CHUDEA2_130	cgd2_130	0.991	0.0222	0.0041	0.184684685
CHUDEA2_1310	cgd2_1310	0.977	0.0793	0.0109	0.137452711
CHUDEA2_1330	cgd2_1330	0.958	0.0323	0.0194	0.600619195
CHUDEA2_1340	cgd2_1340	0.993	0.0507	0.0033	0.065088757
CHUDEA2_1350	cgd2_1350	1	0.0613	0	0
CHUDEA2_1360	cgd2_1360	0.985	0.0953	0.0067	0.070304302
CHUDEA2_1370	cgd2_1370	0.983	0.0544	0.0077	0.141544118
CHUDEA2_1380	cgd2_1380	0.929	0.026	0.033	1.269230769
CHUDEA2_1390	cgd2_1390	0.985	0.0483	0.0066	0.136645963
CHUDEA2_1400	cgd2_1400	0.964	0.0444	0.016	0.36036036
CHUDEA2_140	cgd2_140	0.985	0.1059	0.0065	0.061378659
CHUDEA2_1410	cgd2_1410	0.93	0.0676	0.0355	0.525147929
CHUDEA2_1420	cgd2_1420	0.996	0.0511	0.0016	0.031311155
CHUDEA2_1430	cgd2_1430	0.959	0.0777	0.0178	0.229086229
CHUDEA2_1440	cgd2_1440	1	0.0495	0	0
CHUDEA2_1450	cgd2_1450	0.986	0.0486	0.0063	0.12962963
CHUDEA2_1460	cgd2_1460	0.976	0.0482	0.0102	0.211618257
CHUDEA2_1470	cgd2_1470	0.989	0.0659	0.0048	0.072837633
CHUDEA2_1480	cgd2_1480	0.982	0.0346	0.0077	0.222543353
CHUDEA2_1490	cgd2_1490	0.994	0.0685	0.0027	0.039416058
CHUDEA2_1500	cgd2_1500	0.994	0.089	0.0023	0.025842697
CHUDEA2_150	cgd2_150	0.954	0.0826	0.0176	0.213075061
CHUDEA2_1510	cgd2_1510	0.959	0.0357	0.0194	0.543417367
CHUDEA2_1520	cgd2_1520	0.997	0.0505	0.0015	0.02970297
CHUDEA2_1530	cgd2_1530	0.993	0.09	0.0034	0.037777778
CHUDEA2_1540	cgd2_1540	0.98	0.0552	0.0084	0.152173913

CHUDEA2_1550	cgd2_1550	0.986	0.0802	0.0064	0.079800499
CHUDEA2_1560	cgd2_1560	0.977	0.0443	0.0103	0.232505643
CHUDEA2_1570	cgd2_1570	1	0.0696	0	0
CHUDEA2_1580	cgd2_1580	0.975	0.062	0.0108	0.174193548
CHUDEA2_1590	cgd2_1590	0.98	0.0464	0.0088	0.189655172
CHUDEA2_1600	cgd2_1600	0.984	0.0745	0.0072	0.096644295
CHUDEA2_160	cgd2_160	0.986	0.081	0.0056	0.069135802
CHUDEA2_1610	cgd2_1610	0.994	0.0595	0.0025	0.042016807
CHUDEA2_1620	cgd2_1620	0.972	0.0583	0.0119	0.204116638
CHUDEA2_1630	cgd2_1630	0.939	0.0737	0.0268	0.363636364
CHUDEA2_1640	cgd2_1640	0.975	0.0665	0.0121	0.181954887
CHUDEA2_1650	cgd2_1650	0.979	0.065	0.0093	0.143076923
CHUDEA2_1660	cgd2_1660	0.988	0.0758	0.0051	0.067282322
CHUDEA2_1670	cgd2_1670	0.966	0.0336	0.0156	0.464285714
CHUDEA2_1680	cgd2_1680	0.949	0.0705	0.0244	0.346099291
CHUDEA2_1690	cgd2_1690	1	0.0488	0	0
CHUDEA2_1700	cgd2_1700	1	0.0374	0	0
CHUDEA2_170	cgd2_170	1	0.0196	0	0
CHUDEA2_1710	cgd2_1710	0.958	0.0544	0.0192	0.352941176
CHUDEA2_1720	cgd2_1720	0.973	0.0552	0.0124	0.224637681
CHUDEA2_1730	cgd2_1730	0.956	0.0453	0.0187	0.412803532
CHUDEA2_1740	cgd2_1740	0.964	0.0594	0.0144	0.242424242
CHUDEA2_1750	cgd2_1750	0.971	0.0609	0.0131	0.215106732
CHUDEA2_1760	cgd2_1760	0.976	0.0863	0.0096	0.111239861
CHUDEA2_1770	cgd2_1770	0.974	0.0528	0.0116	0.21969697
CHUDEA2_1780	cgd2_1780	0.984	0.0464	0.0084	0.181034483
CHUDEA2_1790	cgd2_1790	0.967	0.0464	0.0155	0.334051724
CHUDEA2_1800	cgd2_1800	1	0.0442	0	0
CHUDEA2_180	cgd2_180	0.935	0.0635	0.0305	0.480314961
CHUDEA2_1810	cgd2_1810	0.981	0.0549	0.008	0.14571949
CHUDEA2_1820	cgd2_1820	0.975	0.0428	0.0133	0.310747664
CHUDEA2_1830	cgd2_1830	0.984	0.0642	0.007	0.109034268
CHUDEA2_1840	cgd2_1840	0.963	0.0664	0.0166	0.25
CHUDEA2_1850	cgd2_1850	0.997	0.0651	0.0015	0.023041475
CHUDEA2_1860	cgd2_1860	0.966	0.0658	0.0153	0.232522796
CHUDEA2_1870	cgd2_1870	1	0.0462	0	0
CHUDEA2_1880	cgd2_1880	0.983	0.0985	0.0072	0.073096447
CHUDEA2_1890	cgd2_1890	0.953	0.1172	0.0202	0.172354949
CHUDEA2_1900	cgd2_1900	0.967	0.0857	0.0154	0.179696616
CHUDEA2_190	cgd2_190	1	0.0846	0	0
CHUDEA2_1910	cgd2_1910	0.976	0.0451	0.0111	0.246119734
CHUDEA2_1920	cgd2_1920	0.97	0.0392	0.0134	0.341836735
CHUDEA2_1930	cgd2_1930	0.982	0.052	0.0081	0.155769231
CHUDEA2_1940	cgd2_1940	0.995	0.072	0.0021	0.029166667
CHUDEA2_1950	cgd2_1950	0.968	0.0454	0.0147	0.323788546

CHUDEA2_1960	cgd2_1960	0.98	0.0781	0.0086	0.110115237
CHUDEA2_1970	cgd2_1970	0.94	0.0572	0.0223	0.38986014
CHUDEA2_1980	cgd2_1980	0.962	0.0582	0.0174	0.298969072
CHUDEA2_1990	cgd2_1990	0.97	0.056	0.0147	0.2625
CHUDEA2_2000	cgd2_2000	0.992	0.0803	0.0032	0.03985056
CHUDEA2_200	cgd2_200	0.972	0.0619	0.003	0.048465267
CHUDEA2_2010	cgd2_2010	0.993	0.0826	0.0033	0.039951574
CHUDEA2_2020	cgd2_2020	0.99	0.0787	0.0044	0.055908513
CHUDEA2_2030	cgd2_2030	0.978	0.0223	0.0097	0.434977578
CHUDEA2_2040	cgd2_2040	0.97	0.0939	0.0141	0.150159744
CHUDEA2_2050	cgd2_2050	0.977	0.0654	0.0098	0.149847095
CHUDEA2_2060	cgd2_2060	0.961	0.0487	0.0176	0.361396304
CHUDEA2_2070	cgd2_2070	0.985	0.0853	0.0069	0.080890973
CHUDEA2_2080	cgd2_2080	0.963	0.035	0.0161	0.46
CHUDEA2_2090	cgd2_2090	0.977	0.0532	0.0105	0.197368421
CHUDEA2_20	cgd2_20	0.993	0.0677	0.0007	0.010339734
CHUDEA2_2100	cgd2_2100	0.978	0.0385	0.0101	0.262337662
CHUDEA2_210	cgd2_210	0.993	0.0594	0.003	0.050505051
CHUDEA2_2110	cgd2_2110	0.984	0.0686	0.0069	0.10058309
CHUDEA2_2120	cgd2_2120	0.989	0	0.0048	1000
CHUDEA2_2130	cgd2_2130	0.992	0.083	0.0037	0.044578313
CHUDEA2_2140	cgd2_2140	0.985	0.0505	0.0068	0.134653465
CHUDEA2_2150	cgd2_2150	0.948	0.052	0.0233	0.448076923
CHUDEA2_2160	cgd2_2160	0.964	0.0539	0.0164	0.304267161
CHUDEA2_2170	cgd2_2170	0.975	0.0822	0.0096	0.116788321
CHUDEA2_2180	cgd2_2180	0.974	0.0536	0.0112	0.208955224
CHUDEA2_2190	cgd2_2190	0.98	0.0221	0.0076	0.343891403
CHUDEA2_2200	cgd2_2200	1	0.0989	0	0
CHUDEA2_220	cgd2_220	0.971	0.0603	0.0081	0.134328358
CHUDEA2_2210	cgd2_2210	0.98	0.0463	0.0093	0.200863931
CHUDEA2_2220	cgd2_2220	0.958	0.0468	0.0226	0.482905983
CHUDEA2_2230	cgd2_2230	0.978	0.0581	0.01	0.17211704
CHUDEA2_2240	cgd2_2240	0.98	0.0583	0.0097	0.166380789
CHUDEA2_2250	cgd2_2250	0.995	0.0819	0.0022	0.026862027
CHUDEA2_2260	cgd2_2260	0.951	0.0662	0.0198	0.299093656
CHUDEA2_2270	cgd2_2270	0.968	0.0572	0.0143	0.25
CHUDEA2_2280	cgd2_2280	0.971	0.0546	0.0133	0.243589744
CHUDEA2_2290	cgd2_2290	0.945	0.0388	0.0264	0.680412371
CHUDEA2_2300	cgd2_2300	0.988	0.0441	0.0053	0.120181406
CHUDEA2_230	cgd2_230	0.977	0.0899	0.0096	0.106785317
CHUDEA2_2310	cgd2_2310	0.971	0.0527	0.0125	0.237191651
CHUDEA2_2320	cgd2_2320	0.988	0.0616	0.0054	0.087662338
CHUDEA2_2330	cgd2_2330	0.952	0.0493	0.0203	0.411764706
CHUDEA2_2340	cgd2_2340	0.964	0.063	0.0161	0.255555556
CHUDEA2_2350	cgd2_2350	0.975	0.0507	0.0039	0.076923077

CHUDEA2_2360	cgd2_2360	0.967	0.058	0.0145	0.25
CHUDEA2_2370	cgd2_2370	0.918	0.0632	0.0352	0.556962025
CHUDEA2_2380	cgd2_2380	0.919	0.0297	0.04	1.346801347
CHUDEA2_2390	cgd2_2390	0.949	0.0546	0.0232	0.424908425
CHUDEA2_2400	cgd2_2400	0.981	0.0785	0.0085	0.108280255
CHUDEA2_240	cgd2_240	0.983	0.0892	0.0066	0.073991031
CHUDEA2_2410	cgd2_2410	0.987	0.0103	0.0055	0.533980583
CHUDEA2_2420	cgd2_2420	0.967	0.0553	0.0149	0.269439421
CHUDEA2_2430	cgd2_2430	0.962	0.0463	0.0173	0.373650108
CHUDEA2_2440	cgd2_2440	0.968	0.0443	0.0142	0.320541761
CHUDEA2_2450	cgd2_2450	0.97	0.0733	0.0131	0.178717599
CHUDEA2_2460	cgd2_2460	0.947	0.0488	0.0219	0.448770492
CHUDEA2_2470	cgd2_2470	0.936	0.0927	0.0291	0.313915858
CHUDEA2_2480	cgd2_2480	0.994	0.0549	0.0028	0.051001821
CHUDEA2_2490	cgd2_2490	0.946	0.0612	0.0257	0.419934641
CHUDEA2_2500	cgd2_2500	0.958	0.0713	0.0189	0.265077139
CHUDEA2_250	cgd2_250	0.992	0.0832	0.0035	0.042067308
CHUDEA2_2510	cgd2_2510	0.98	0.0563	0.0083	0.147424512
CHUDEA2_2520	cgd2_2520	0.942	0.0605	0.0249	0.411570248
CHUDEA2_2530	cgd2_2530	0.901	0.0881	0.0484	0.549375709
CHUDEA2_2540	cgd2_2540	1	0.066	0	0
CHUDEA2_2550	cgd2_2550	0.879	0.1126	0.0582	0.51687389
CHUDEA2_2570	cgd2_2570	0.883	0.1482	0.0617	0.416329285
CHUDEA2_2580	cgd2_2580	0.954	0.0707	0.0189	0.267326733
CHUDEA2_2590	cgd2_2590	0.945	0.0324	0.0249	0.768518519
CHUDEA2_2600	cgd2_2600	0.92	0.1203	0.0335	0.27847049
CHUDEA2_260	cgd2_260	0.968	0.0952	0.0145	0.152310924
CHUDEA2_2610	cgd2_2610	0.98	0.0577	0.009	0.155979203
CHUDEA2_2620	cgd2_2620	0.968	0.0629	0.0144	0.228934817
CHUDEA2_2630	cgd2_2630	0.967	0.0735	0.0152	0.206802721
CHUDEA2_2640	cgd2_2640	0.978	0.0986	0.0091	0.092292089
CHUDEA2_2650	cgd2_2650	0.932	0.0364	0.031	0.851648352
CHUDEA2_2660	cgd2_2660	0.979	0.0675	0.0093	0.137777778
CHUDEA2_2670	cgd2_2670	0.981	0.0518	0.009	0.173745174
CHUDEA2_2680	cgd2_2680	0.969	0.0537	0.0141	0.262569832
CHUDEA2_2690	cgd2_2690	0.985	0.073	0.0067	0.091780822
CHUDEA2_2700	cgd2_2700	0.998	0.0985	0.0009	0.009137056
CHUDEA2_270	cgd2_270	0.996	0.0832	0.0016	0.019230769
CHUDEA2_2710	cgd2_2710	0.971	0.0728	0.0149	0.20467033
CHUDEA2_2720	cgd2_2720	0.985	0.0421	0.0065	0.154394299
CHUDEA2_2730	cgd2_2730	0.972	0.0759	0.0133	0.175230567
CHUDEA2_2740	cgd2_2740	0.969	0.0436	0.0129	0.29587156
CHUDEA2_2750	cgd2_2750	0.974	0.0541	0.0121	0.223659889
CHUDEA2_2760	cgd2_2760	0.966	0.0639	0.015	0.234741784
CHUDEA2_2770	cgd2_2770	0.973	0.0592	0.0116	0.195945946

CHUDEA2_2780	cgd2_2780	0.968	0.0567	0.0156	0.275132275
CHUDEA2_2790	cgd2_2790	0.991	0.0687	0.0037	0.053857351
CHUDEA2_2800	cgd2_2800	0.961	0.0642	0.0189	0.294392523
CHUDEA2_280	cgd2_280	1	0.0376	0	0
CHUDEA2_2810	cgd2_2810	0.948	0.068	0.0225	0.330882353
CHUDEA2_2820	cgd2_2820	0.908	0.1099	0.0032	0.029117379
CHUDEA2_2830	cgd2_2830	0.954	0.0666	0.02	0.3003003
CHUDEA2_2840	cgd2_2840	0.976	0.0494	0.0103	0.208502024
CHUDEA2_2850	cgd2_2850	0.946	0.0259	0.0235	0.907335907
CHUDEA2_2860	cgd2_2860	0.968	0.0902	0.0117	0.129711752
CHUDEA2_2870	cgd2_2870	1	0.1346	0	0
CHUDEA2_2880	cgd2_2880	0.954	0.0739	0.0194	0.262516915
CHUDEA2_2890	cgd2_2890	0.987	0.0846	0.0054	0.063829787
CHUDEA2_2900	cgd2_2900	0.877	0.0601	0.0618	1.02828619
CHUDEA2_290	cgd2_290	0.956	0.0601	0.0182	0.302828619
CHUDEA2_2910	cgd2_2910	0.98	0.0623	0.0089	0.142857143
CHUDEA2_2920	cgd2_2920	1	0.0516	0	0
CHUDEA2_2930	cgd2_2930	0.978	0.0604	0.01	0.165562914
CHUDEA2_2940	cgd2_2940	0.979	0.054	0.0107	0.198148148
CHUDEA2_2950	cgd2_2950	0.967	0.0535	0.0153	0.285981308
CHUDEA2_2960	cgd2_2960	0.994	0.0604	0.0024	0.039735099
CHUDEA2_2970	cgd2_2970	1	0.071	0	0
CHUDEA2_2980	cgd2_2980	0.968	0.0643	0.0142	0.220839813
CHUDEA2_2990	cgd2_2990	0.99	0.0296	0.0045	0.152027027
CHUDEA2_3000	cgd2_3000	0.993	0.0177	0.0032	0.18079096
CHUDEA2_300	cgd2_300	0.975	0.0775	0.0106	0.136774194
CHUDEA2_3010	cgd2_3010	0.991	0.056	0.0042	0.075
CHUDEA2_3020	cgd2_3020	0.977	0.0776	0.0104	0.134020619
CHUDEA2_3030	cgd2_3030	0.946	0.0559	0.0253	0.452593918
CHUDEA2_3040	cgd2_3040	1	0.0882	0	0
CHUDEA2_3050	cgd2_3050	0.951	0.0598	0.021	0.351170569
CHUDEA2_3060	cgd2_3060	0.968	0.051	0.0128	0.250980392
CHUDEA2_3070	cgd2_3070	1	0.0543	0	0
CHUDEA2_3080	cgd2_3080	0.972	0.0637	0.0124	0.19466248
CHUDEA2_3090	cgd2_3090	0.923	0.0524	0.0387	0.738549618
CHUDEA2_30	cgd2_30	0.978	0.0935	0.0085	0.090909091
CHUDEA2_3100	cgd2_3100	0.982	0.0613	0.0098	0.159869494
CHUDEA2_310	cgd2_310	0.984	0.0534	0.0064	0.119850187
CHUDEA2_3110	cgd2_3110	0.931	0.0913	0.0295	0.323110624
CHUDEA2_3120	cgd2_3120	0.934	0.0815	0.028	0.343558282
CHUDEA2_3150	cgd2_3150	0.964	0.0363	0.0117	0.32231405
CHUDEA2_3160	cgd2_3160	0.971	0.0418	0.0117	0.279904306
CHUDEA2_3170	cgd2_3170	0.978	0.0637	0.0094	0.147566719
CHUDEA2_3180	cgd2_3180	1	0.0625	0	0
CHUDEA2_3190	cgd2_3190	0.994	0.0718	0.0024	0.033426184

CHUDEA2_3200	cgd2_3200	0.989	0.109	0.0045	0.041284404
CHUDEA2_320	cgd2_320	0.986	0.0599	0.0058	0.096828047
CHUDEA2_3210	cgd2_3210	0.969	0.0388	0.0164	0.422680412
CHUDEA2_3220	cgd2_3220	0.989	0.0661	0.0044	0.066565809
CHUDEA2_3230	cgd2_3230	0.988	0.0602	0.0028	0.046511628
CHUDEA2_3240	cgd2_3240	0.983	0.0552	0.0074	0.134057971
CHUDEA2_3250	cgd2_3250	0.978	0.0583	0.0096	0.164665523
CHUDEA2_3260	cgd2_3260	0.977	0.1311	0.01	0.076277651
CHUDEA2_3270	cgd2_3270	0.977	0.1417	0.01	0.07057163
CHUDEA2_3290	cgd2_3290	0.937	0.0819	0.0239	0.291819292
CHUDEA2_3300	cgd2_3300	0.947	0.0938	0.0214	0.228144989
CHUDEA2_330	cgd2_330	0.987	0.0556	0.0056	0.100719424
CHUDEA2_3310	cgd2_3310	0.959	0.0465	0.0171	0.367741935
CHUDEA2_3320	cgd2_3320	0.959	0.0659	0.0136	0.206373293
CHUDEA2_3330	cgd2_3330	0.981	0.0633	0.0084	0.132701422
CHUDEA2_3340	cgd2_3340	0.972	0.0682	0.0085	0.124633431
CHUDEA2_3350	cgd2_3350	0.997	0.1053	0.0014	0.013295347
CHUDEA2_3360	cgd2_3360	0.967	0.0523	0.015	0.286806883
CHUDEA2_3380	cgd2_3380	0.979	0.0464	0.0094	0.202586207
CHUDEA2_3390	cgd2_3390	0.976	0.0325	0.0101	0.310769231
CHUDEA2_3400	cgd2_3400	0.979	0.0691	0.0096	0.138929088
CHUDEA2_340	cgd2_340	0.961	0.0525	0.0177	0.337142857
CHUDEA2_3410	cgd2_3410	1	0.0436	0	0
CHUDEA2_3420	cgd2_3420	0.982	0.0513	0.0077	0.150097466
CHUDEA2_3430	cgd2_3430	0.977	0.0801	0.0095	0.118601748
CHUDEA2_3440	cgd2_3440	0.973	0.0402	0.0109	0.271144279
CHUDEA2_3450	cgd2_3450	0.992	0.0577	0.0034	0.058925477
CHUDEA2_3460	cgd2_3460	0.961	0.0722	0.0183	0.253462604
CHUDEA2_3470	cgd2_3470	0.963	0.0333	0.0162	0.486486486
CHUDEA2_3480	cgd2_3480	0.973	0.0405	0.0129	0.318518519
CHUDEA2_3490	cgd2_3490	0.956	0.0777	0.0136	0.175032175
CHUDEA2_3500	cgd2_3500	0.963	0.0573	0.0136	0.237347295
CHUDEA2_350	cgd2_350	1	0	0	0
CHUDEA2_3510	cgd2_3510	0.962	0.0815	0.0091	0.111656442
CHUDEA2_3520	cgd2_3520	0.832	0.1539	0.0779	0.50617284
CHUDEA2_3530	cgd2_3530	0.786	0.089	0.0499	0.560674157
CHUDEA2_3540	cgd2_3540	0.945	0.0973	0.014	0.143884892
CHUDEA2_3550	cgd2_3550	0.941	0.1104	0.0116	0.105072464
CHUDEA2_3560	cgd2_3560	0.951	0.0368	0.0147	0.399456522
CHUDEA2_3570	cgd2_3570	0.947	0.0677	0.013	0.192023634
CHUDEA2_3580	cgd2_3580	0.955	0.0731	0.0166	0.227086183
CHUDEA2_3590	cgd2_3590	0.969	0.0709	0.011	0.155148096
CHUDEA2_3600	cgd2_3600	0.972	0.0567	0.0114	0.201058201
CHUDEA2_360	cgd2_360	0.997	0.0498	0.0012	0.024096386
CHUDEA2_3610	cgd2_3610	0.972	0.0669	0.0112	0.167414051

CHUDEA2_3620	cgd2_3620	0.965	0.0868	0.0151	0.173963134
CHUDEA2_3630	cgd2_3630	0.939	0.0554	0.0284	0.512635379
CHUDEA2_3640	cgd2_3640	0.984	0.0524	0.0074	0.141221374
CHUDEA2_3650	cgd2_3650	0.966	0.055	0.0134	0.243636364
CHUDEA2_3660	cgd2_3660	0.988	0.0806	0.0025	0.03101737
CHUDEA2_3670	cgd2_3670	0.973	0.0436	0.0103	0.236238532
CHUDEA2_3680	cgd2_3680	0.987	0.0541	0.0055	0.101663586
CHUDEA2_3690	cgd2_3690	0.961	0.0613	0.0151	0.246329527
CHUDEA2_3700	cgd2_3700	0.972	0.065	0.0073	0.112307692
CHUDEA2_370	cgd2_370	0.996	0.0565	0.0015	0.026548673
CHUDEA2_3710	cgd2_3710	0.992	0.0566	0.0033	0.058303887
CHUDEA2_3720	cgd2_3720	0.97	0.1167	0.0143	0.122536418
CHUDEA2_3730	cgd2_3730	0.974	0.0532	0.012	0.22556391
CHUDEA2_3740	cgd2_3740	0.961	0.1184	0.0156	0.131756757
CHUDEA2_3750	cgd2_3750	0.94	0.1155	0.0253	0.219047619
CHUDEA2_3760	cgd2_3760	0.987	0.1022	0.0057	0.055772994
CHUDEA2_3770	cgd2_3770	0.973	0.0537	0.0116	0.216014898
CHUDEA2_3780	cgd2_3780	0.941	0.131	0.021	0.160305344
CHUDEA2_3790	cgd2_3790	0.942	0.1166	0.0306	0.262435678
CHUDEA2_3800	cgd2_3800	0.946	0.0818	0.0181	0.221271394
CHUDEA2_380	cgd2_380	1	0.1268	0	0
CHUDEA2_3810	cgd2_3810	0.966	0.0331	0.0124	0.374622356
CHUDEA2_3830	cgd2_3830	0.972	0.0667	0.0114	0.170914543
CHUDEA2_3840	cgd2_3840	0.95	0.0326	0.0139	0.426380368
CHUDEA2_3850	cgd2_3850	0.935	0.0661	0.0153	0.231467474
CHUDEA2_3860	cgd2_3860	0.99	0.0766	0.0038	0.049608355
CHUDEA2_3870	cgd2_3870	0.968	0.0675	0.0097	0.143703704
CHUDEA2_3880	cgd2_3880	0.954	0.0665	0.0072	0.108270677
CHUDEA2_3890	cgd2_3890	0.982	0.0435	0.0047	0.108045977
CHUDEA2_3900	cgd2_3900	0.945	0.075	0.0135	0.18
CHUDEA2_390	cgd2_390	0.813	0.0768	0.1003	1.305989583
CHUDEA2_3910	cgd2_3910	1	0.0553	0	0
CHUDEA2_3920	cgd2_3920	0.931	0.1247	0.0197	0.15797915
CHUDEA2_3930	cgd2_3930	0.992	0.1016	0.0034	0.033464567
CHUDEA2_3940	cgd2_3940	0.984	0.0312	0.0066	0.211538462
CHUDEA2_3950	cgd2_3950	0.988	0.03	0.0035	0.116666667
CHUDEA2_3960	cgd2_3960	0.998	0.0566	0.0008	0.014134276
CHUDEA2_3970	cgd2_3970	0.995	0.0642	0.002	0.031152648
CHUDEA2_3980	cgd2_3980	0.966	0.0666	0.0126	0.189189189
CHUDEA2_3990	cgd2_3990	0.984	0.0708	0.0068	0.096045198
CHUDEA2_4000	cgd2_4000	0.993	0.0403	0.0033	0.081885856
CHUDEA2_400	cgd2_400	0.825	0.1612	0.0917	0.568858561
CHUDEA2_4010	cgd2_4010	0.993	0.0745	0.0029	0.038926174
CHUDEA2_4020	cgd2_4020	0.898	0.0619	0.0408	0.659127625
CHUDEA2_4030	cgd2_4030	0.979	0.0697	0.0084	0.120516499

CHUDEA2_4040	cgd2_4040	0.963	0.0819	0.0109	0.133089133
CHUDEA2_4050	cgd2_4050	0.981	0.0449	0.0081	0.180400891
CHUDEA2_4060	cgd2_4060	0.969	0.0384	0.0134	0.348958333
CHUDEA2_4070	cgd2_4070	0.974	0.0953	0.011	0.115424974
CHUDEA2_4080	cgd2_4080	0.992	0.0292	0.0036	0.123287671
CHUDEA2_4090	cgd2_4090	0.983	0.0565	0.0058	0.102654867
CHUDEA2_40	cgd2_40	0.964	0.0593	0.0136	0.229342327
CHUDEA2_4100	cgd2_4100	1	0	0	0
CHUDEA2_410	cgd2_410	0.829	0.09	0.0878	0.975555556
CHUDEA2_4110	cgd2_4110	0.965	0.0513	0.0157	0.306042885
CHUDEA2_4120	cgd2_4120	1	0.0388	0	0
CHUDEA2_4130	cgd2_4130	0.965	0.0455	0.0153	0.336263736
CHUDEA2_4140	cgd2_4140	0.975	0.0378	0.0106	0.28042328
CHUDEA2_4150	cgd2_4150	1	0.0274	0	0
CHUDEA2_4160	cgd2_4160	0.987	0.0537	0.0053	0.098696462
CHUDEA2_4170	cgd2_4170	0.958	0.0354	0.0188	0.531073446
CHUDEA2_4180	cgd2_4180	1	0.0279	0	0
CHUDEA2_4190	cgd2_4190	0.972	0.0497	0.0124	0.249496982
CHUDEA2_4200	cgd2_4200	0.973	0.0564	0.0118	0.209219858
CHUDEA2_420	cgd2_420	0.572	0.1542	0.2372	1.538261997
CHUDEA2_4210	cgd2_4210	0.975	0.0874	0.008	0.091533181
CHUDEA2_4220	cgd2_4220	0.964	0.0543	0.0165	0.303867403
CHUDEA2_4230	cgd2_4230	0.966	0.0913	0.015	0.164293538
CHUDEA2_4240	cgd2_4240	0.986	0.05	0.0063	0.126
CHUDEA2_4250	cgd2_4250	0.969	0.062	0.0132	0.212903226
CHUDEA2_4260	cgd2_4260	1	0.0283	0	0
CHUDEA2_4270	cgd2_4270	0.976	0.0439	0.0109	0.248291572
CHUDEA2_4280	cgd2_4280	0.98	0.0638	0.0103	0.161442006
CHUDEA2_4290	cgd2_4290	0.971	0.0754	0.0123	0.163129973
CHUDEA2_4300	cgd2_4300	0.981	0.066	0.0082	0.124242424
CHUDEA2_430	cgd2_430	0.709	0.1693	0.1499	0.885410514
CHUDEA2_4310	cgd2_4310	0.936	0.0462	0.0298	0.645021645
CHUDEA2_4320	cgd2_4320	0.996	0.0778	0.0017	0.0218509
CHUDEA2_4330	cgd2_4330	1	0.0725	0	0
CHUDEA2_4340	cgd2_4340	0.984	0.0465	0.0053	0.113978495
CHUDEA2_4350	cgd2_4350	0.979	0.0934	0.0088	0.094218415
CHUDEA2_4360	cgd2_4360	0.979	0.0593	0.009	0.151770658
CHUDEA2_4370	cgd2_4370	0.78	0.0963	0.1171	1.215991693
CHUDEA2_440	cgd2_440	0.785	0.0311	0.1002	3.221864952
CHUDEA2_450	cgd2_450	0.79	0.1948	0.0982	0.504106776
CHUDEA2_480	cgd2_480	0.958	0.0549	0.009	0.163934426
CHUDEA2_490	cgd2_490	0.961	0.0496	0.0095	0.191532258
CHUDEA2_500	cgd2_500	0.981	0.0557	0.0085	0.152603232
CHUDEA2_50	cgd2_50	0.994	0.0567	0.0025	0.044091711
CHUDEA2_510	cgd2_510	0.974	0.0443	0.0098	0.221218962

CHUDEA2_520	cgd2_520	0.971	0.0513	0.0119	0.231968811
CHUDEA2_530	cgd2_530	0.995	0.0737	0.002	0.027137042
CHUDEA2_540	cgd2_540	0.941	0.0757	0.0181	0.239101717
CHUDEA2_550	cgd2_550	0.996	0.0582	0.0019	0.032646048
CHUDEA2_560	cgd2_560	0.972	0.0644	0.012	0.186335404
CHUDEA2_570	cgd2_570	0.974	0.0587	0.01	0.170357751
CHUDEA2_580	cgd2_580	0.97	0.0758	0.0116	0.153034301
CHUDEA2_590	cgd2_590	0.991	0.0532	0.004	0.07518797
CHUDEA2_600	cgd2_600	0.996	0.0592	0.0016	0.027027027
CHUDEA2_60	cgd2_60	0.981	0.0526	0.0066	0.125475285
CHUDEA2_610	cgd2_610	0.983	0.0394	0.0085	0.215736041
CHUDEA2_620	cgd2_620	0.989	0.0652	0.0046	0.070552147
CHUDEA2_630	cgd2_630	0.958	0.0511	0.0102	0.199608611
CHUDEA2_640	cgd2_640	0.988	0.0501	0.0051	0.101796407
CHUDEA2_650	cgd2_650	0.952	0.0501	0.0194	0.387225549
CHUDEA2_660	cgd2_660	0.984	0.0476	0.0069	0.144957983
CHUDEA2_670	cgd2_670	0.981	0.0466	0.0076	0.163090129
CHUDEA2_680	cgd2_680	0.957	0.0652	0.0133	0.20398773
CHUDEA2_690	cgd2_690	0.915	0.0901	0.0189	0.209766926
CHUDEA2_700	cgd2_700	0.94	0.0671	0.0136	0.202682563
CHUDEA2_70	cgd2_70	0.968	0.1075	0.0093	0.086511628
CHUDEA2_710	cgd2_710	0.989	0.0742	0.0045	0.0606469
CHUDEA2_720	cgd2_720	1	0.0924	0	0
CHUDEA2_730	cgd2_730	0.993	0.0466	0.0034	0.072961373
CHUDEA2_740	cgd2_740	1	0.0386	0	0
CHUDEA2_750	cgd2_750	0.974	0.0547	0.012	0.219378428
CHUDEA2_760	cgd2_760	0.988	0.0366	0.0055	0.150273224
CHUDEA2_770	cgd2_770	0.958	0.0529	0.0206	0.389413989
CHUDEA2_780	cgd2_780	0.995	0.0844	0.0022	0.026066351
CHUDEA2_790	cgd2_790	0.994	0.0493	0.0024	0.048681542
CHUDEA2_800	cgd2_800	0.977	0.0343	0.0109	0.317784257
CHUDEA2_80	cgd2_80	0.978	0.0664	0.0074	0.111445783
CHUDEA2_810	cgd2_810	1	0.0641	0	0
CHUDEA2_820	cgd2_820	1	0.0112	0	0
CHUDEA2_830	cgd2_830	0.991	0.0577	0.0036	0.062391681
CHUDEA2_840	cgd2_840	0.981	0.0752	0.0082	0.109042553
CHUDEA2_850	cgd2_850	0.997	0.0482	0.0014	0.029045643
CHUDEA2_860	cgd2_860	0.995	0.0479	0.0021	0.043841336
CHUDEA2_870	cgd2_870	0.996	0.0436	0.002	0.04587156
CHUDEA2_880	cgd2_880	0.954	0.0535	0.023	0.429906542
CHUDEA2_890	cgd2_890	0.966	0.0252	0.016	0.634920635
CHUDEA2_900	cgd2_900	0.991	0.0479	0.0039	0.081419624
CHUDEA2_90	cgd2_90	0.977	0.1127	0.0088	0.078083407
CHUDEA2_910	cgd2_910	0.976	0.0588	0.0103	0.175170068
CHUDEA2_920	cgd2_920	0.981	0.0575	0.008	0.139130435

CHUDEA2_930	cgd2_930	0.98	0.0725	0.0087	0.12
CHUDEA2_940	cgd2_940	0.934	0.0811	0.0286	0.352651048
CHUDEA2_950	cgd2_950	0.973	0.0574	0.0115	0.200348432
CHUDEA2_960	cgd2_960	0.97	0.0413	0.0116	0.280871671
CHUDEA2_970	cgd2_970	0.979	0.0852	0.0091	0.106807512
CHUDEA2_980	cgd2_980	1	0.0447	0	0
CHUDEA2_990	cgd2_990	0.976	0.0377	0.0108	0.286472149
CHUDEA2_newUdeA_01	cgd5_4570	0.266	3.3515	0.6792	0.202655527
CHUDEA2_new_01	cgd2_new_01	0.976	0.063	0.0114	0.180952381
CHUDEA2_new_02	cgd2_new_02	0.981	0.0402	0.0077	0.191542289
CHUDEA2_new_03	cgd2_new_03	0.969	0.0162	0.0134	0.827160494
CHUDEA2_new_04	cgd2_new_04	0.972	0.082	0.012	0.146341463
CHUDEA2_new_05	cgd2_new_05	0.991	0.1052	0.0039	0.037072243
CHUDEA2_new_06	cgd2_new_06	0.99	0.0498	0.0049	0.098393574
CHUDEA2_new_07	cgd2_new_07	0.988	0.0084	0.0054	0.642857143
CHUDEA3_1000	cgd3_1000	1	0.0595	0	0
CHUDEA3_100	cgd3_100	0.932	0.0378	0.0311	0.822751323
CHUDEA3_1010	cgd3_1010	0.94	0.0733	0.0288	0.392905866
CHUDEA3_1020	cgd3_1020	0.985	0.0605	0.0066	0.109090909
CHUDEA3_1030	cgd3_1030	0.94	0.0772	0.0113	0.146373057
CHUDEA3_1040	cgd3_1040	0.93	0.0403	0.03	0.744416873
CHUDEA3_1050	cgd3_1050	0.978	0.0529	0.0102	0.192816635
CHUDEA3_1060	cgd3_1060	0.97	0.0288	0.0112	0.388888889
CHUDEA3_1070	cgd3_1070	0.971	0.0407	0.013	0.319410319
CHUDEA3_1080	cgd3_1080	0.971	0.0366	0.0131	0.357923497
CHUDEA3_1090	cgd3_1090	0.982	0.046	0.0064	0.139130435
CHUDEA3_10	cgd3_10	0.833	0.1547	0.0805	0.520361991
CHUDEA3_1100	cgd3_1100	0.826	0.0508	0.0467	0.919291339
CHUDEA3_110	cgd3_110	0.976	0.0706	0.0109	0.154390935
CHUDEA3_1110	cgd3_1110	0.964	0.0684	0.0155	0.226608187
CHUDEA3_1120	cgd3_1120	0.989	0.067	0.0048	0.071641791
CHUDEA3_1130	cgd3_1130	0.978	0.1491	0.0068	0.045606975
CHUDEA3_1140	cgd3_1140	0.928	0.0707	0.0334	0.47241867
CHUDEA3_1150	cgd3_1150	0.702	0.1995	0.1655	0.829573935
CHUDEA3_1160	cgd3_1160	0.848	0.1068	0.0753	0.70505618
CHUDEA3_1180	cgd3_1180	0.962	0.0297	0.0094	0.316498316
CHUDEA3_1200	cgd3_1200	0.991	0.0913	0.0037	0.040525739
CHUDEA3_120	cgd3_120	0.987	0.0448	0.0058	0.129464286
CHUDEA3_1210	cgd3_1210	0.983	0.068	0.0075	0.110294118
CHUDEA3_1220	cgd3_1220	0.972	0.0406	0.0117	0.28817734
CHUDEA3_1230	cgd3_1230	0.981	0.0331	0.0074	0.223564955
CHUDEA3_1240	cgd3_1240	0.978	0.0368	0.0111	0.301630435
CHUDEA3_1250	cgd3_1250	0.992	0.0928	0.0033	0.035560345
CHUDEA3_1260	cgd3_1260	0.985	0.0402	0.0068	0.169154229
CHUDEA3_1270	cgd3_1270	0.995	0.0739	0.0021	0.028416779

CHUDEA3_1280	cgd3_1280	0.961	0.0425	0.0203	0.477647059
CHUDEA3_1290	cgd3_1290	0.996	0.0428	0.0018	0.042056075
CHUDEA3_1300	cgd3_1300	1	0.0236	0	0
CHUDEA3_130	cgd3_130	0.995	0.0582	0.0025	0.042955326
CHUDEA3_1310	cgd3_1310	0.971	0.0623	0.0131	0.210272873
CHUDEA3_1320	cgd3_1320	0.98	0.0713	0.0087	0.122019635
CHUDEA3_1340	cgd3_1340	0.99	0.0568	0.0044	0.077464789
CHUDEA3_1350	cgd3_1350	0.96	0.0644	0.0176	0.273291925
CHUDEA3_1360	cgd3_1360	0.993	0.0765	0.0028	0.036601307
CHUDEA3_1370	cgd3_1370	0.984	0.0915	0.0079	0.086338798
CHUDEA3_1380	cgd3_1380	0.98	0.0533	0.0089	0.166979362
CHUDEA3_1390	cgd3_1390	0.984	0.0541	0.0078	0.144177449
CHUDEA3_1400	cgd3_1400	0.974	0.0534	0.0116	0.217228464
CHUDEA3_140	cgd3_140	0.971	0.0558	0.0135	0.241935484
CHUDEA3_1410	cgd3_1410	1	0.0102	0	0
CHUDEA3_1420	cgd3_1420	1	0	0	0
CHUDEA3_1430	cgd3_1430	0.958	0.0602	0.0174	0.289036545
CHUDEA3_1440	cgd3_1440	0.973	0.0597	0.0121	0.202680067
CHUDEA3_1450	cgd3_1450	0.997	0.04	0.0013	0.0325
CHUDEA3_1460	cgd3_1460	0.989	0.0379	0.0043	0.113456464
CHUDEA3_1470	cgd3_1470	0.984	0.0958	0.0073	0.076200418
CHUDEA3_1480	cgd3_1480	0.984	0.0368	0.0075	0.203804348
CHUDEA3_1490	cgd3_1490	0.97	0.1139	0.0131	0.115013169
CHUDEA3_1500	cgd3_1500	0.982	0.0417	0.0079	0.189448441
CHUDEA3_150	cgd3_150	0.99	0.0531	0.0046	0.086629002
CHUDEA3_1510	cgd3_1510	0.997	0.0536	0.0014	0.026119403
CHUDEA3_1520	cgd3_1520	0.975	0.0336	0.0118	0.351190476
CHUDEA3_1530	cgd3_1530	0.98	0.0591	0.008	0.13536379
CHUDEA3_1540	cgd3_1540	0.983	0.0889	0.0068	0.076490439
CHUDEA3_1550	cgd3_1550	0.954	0.0877	0.0136	0.155074116
CHUDEA3_1560	cgd3_1560	1	0.0442	0	0
CHUDEA3_1570	cgd3_1570	0.981	0.1242	0.0084	0.06763285
CHUDEA3_1580	cgd3_1580	0.985	0.0566	0.0059	0.104240283
CHUDEA3_1590	cgd3_1590	0.978	0.0534	0.0101	0.189138577
CHUDEA3_1600	cgd3_1600	0.974	0.0859	0.0102	0.118742724
CHUDEA3_160	cgd3_160	0.988	0.0694	0.0053	0.076368876
CHUDEA3_1610	cgd3_1610	0.95	0.0615	0.0213	0.346341463
CHUDEA3_1620	cgd3_1620	0.988	0.0767	0.005	0.065189048
CHUDEA3_1630	cgd3_1630	0.994	0.0562	0.0027	0.048042705
CHUDEA3_1640	cgd3_1640	0.976	0.0434	0.011	0.253456221
CHUDEA3_1650	cgd3_1650	0.998	0.0474	0.001	0.021097046
CHUDEA3_1660	cgd3_1660	0.957	0.0528	0.0207	0.392045455
CHUDEA3_1680	cgd3_1680	0.904	0.1258	0.0485	0.385532591
CHUDEA3_1690	cgd3_1690	0.867	0.1263	0.0657	0.520190024
CHUDEA3_1700	cgd3_1700	0.925	0.0714	0.0354	0.495798319

CHUDEA3_170	cgd3_170	0.97	0.0339	0.0125	0.368731563
CHUDEA3_1710	cgd3_1710	0.857	0.0738	0.0706	0.956639566
CHUDEA3_1720	cgd3_1720	0.948	0.0604	0.0246	0.407284768
CHUDEA3_1730	cgd3_1730	0.879	0.0476	0.0567	1.191176471
CHUDEA3_1740	cgd3_1740	0.85	0.0656	0.0647	0.986280488
CHUDEA3_1750	cgd3_1750	0.887	0.0805	0.058	0.720496894
CHUDEA3_1760	cgd3_1760	0.806	0.1185	0.0977	0.824472574
CHUDEA3_1770	cgd3_1770	0.755	0.1596	0.1334	0.835839599
CHUDEA3_1780	cgd3_1780	0.829	0.0719	0.0777	1.080667594
CHUDEA3_1790	cgd3_1790	0.984	0.0687	0.0077	0.112081514
CHUDEA3_1800	cgd3_1800	0.984	0.037	0.0073	0.197297297
CHUDEA3_180	cgd3_180	0.992	0.0603	0.0037	0.061359867
CHUDEA3_1810	cgd3_1810	0.988	0.0731	0.0052	0.071135431
CHUDEA3_1820	cgd3_1820	0.997	0.0534	0.0012	0.02247191
CHUDEA3_1830	cgd3_1830	0.971	0.0517	0.0127	0.245647969
CHUDEA3_1840	cgd3_1840	0.968	0.057	0.015	0.263157895
CHUDEA3_1850	cgd3_1850	0.991	0.0409	0.0039	0.095354523
CHUDEA3_1860	cgd3_1860	0.977	0.0992	0.0099	0.099798387
CHUDEA3_1870	cgd3_1870	1	0.0746	0	0
CHUDEA3_1880	cgd3_1880	0.966	0.0482	0.0162	0.336099585
CHUDEA3_1890	cgd3_1890	0.984	0.0416	0.0078	0.1875
CHUDEA3_1900	cgd3_1900	0.993	0.0869	0.003	0.03452244
CHUDEA3_190	cgd3_190	0.779	0.2533	0.127	0.501381761
CHUDEA3_1910	cgd3_1910	0.965	0.0584	0.016	0.273972603
CHUDEA3_1920	cgd3_1920	0.969	0.0303	0.0139	0.458745875
CHUDEA3_1930	cgd3_1930	0.963	0.0414	0.0171	0.413043478
CHUDEA3_1940	cgd3_1940	0.994	0.0256	0.0028	0.109375
CHUDEA3_1950	cgd3_1950	0.979	0.089	0.0089	0.1
CHUDEA3_1960	cgd3_1960	0.979	0.0339	0.0099	0.292035398
CHUDEA3_1970	cgd3_1970	0.983	0.0627	0.0074	0.118022329
CHUDEA3_1980	cgd3_1980	0.98	0.0664	0.0093	0.140060241
CHUDEA3_1990	cgd3_1990	0.983	0.0606	0.0065	0.107260726
CHUDEA3_2000	cgd3_2000	0.962	0.0456	0.0178	0.390350877
CHUDEA3_200	cgd3_200	1	0.044	0	0
CHUDEA3_2010	cgd3_2010	0.988	0.0546	0.0051	0.093406593
CHUDEA3_2020	cgd3_2020	0.988	0.068	0.0054	0.079411765
CHUDEA3_2030	cgd3_2030	0.995	0.0476	0.0022	0.046218487
CHUDEA3_2040	cgd3_2040	0.987	0.0423	0.0059	0.139479905
CHUDEA3_2050	cgd3_2050	0.987	0.0777	0.007	0.09009009
CHUDEA3_2060	cgd3_2060	0.972	0.0404	0.0115	0.284653465
CHUDEA3_2070	cgd3_2070	0.962	0.0421	0.0171	0.406175772
CHUDEA3_2080	cgd3_2080	0.976	0.0565	0.0116	0.205309735
CHUDEA3_2090	cgd3_2090	1	0.0469	0	0
CHUDEA3_20	cgd3_20	0.963	0.0923	0.0166	0.179848321
CHUDEA3_2100	cgd3_2100	0.99	0.0574	0.0042	0.073170732

CHUDEA3_210	cgd3_210	0.975	0.0639	0.0111	0.17370892
CHUDEA3_2110	cgd3_2110	0.994	0.0785	0.0019	0.024203822
CHUDEA3_2120	cgd3_2120	0.989	0.0613	0.0044	0.07177814
CHUDEA3_2130	cgd3_2130	0.991	0.0342	0.0041	0.119883041
CHUDEA3_2140	cgd3_2140	0.964	0.0421	0.0153	0.363420428
CHUDEA3_2150	cgd3_2150	0.992	0.0495	0.0038	0.076767677
CHUDEA3_2160	cgd3_2160	0.952	0.0647	0.0231	0.357032457
CHUDEA3_2170	cgd3_2170	0.988	0.0512	0.0052	0.1015625
CHUDEA3_2190	cgd3_2190	0.982	0.042	0.0085	0.202380952
CHUDEA3_2200	cgd3_2200	0.984	0.0761	0.0072	0.094612352
CHUDEA3_220	cgd3_220	1	0.0485	0	0
CHUDEA3_2210	cgd3_2210	0.992	0.0941	0.0035	0.037194474
CHUDEA3_2220	cgd3_2220	0.993	0.0565	0.0056	0.099115044
CHUDEA3_2230	cgd3_2230	0.981	0.1214	0.0076	0.062602965
CHUDEA3_2240	cgd3_2240	0.975	0.0522	0.0114	0.218390805
CHUDEA3_2250	cgd3_2250	1	0.0407	0	0
CHUDEA3_2260	cgd3_2260	0.986	0.0356	0.0066	0.185393258
CHUDEA3_2270	cgd3_2270	0.965	0.0452	0.0153	0.338495575
CHUDEA3_2280	cgd3_2280	0.956	0.0466	0.0199	0.427038627
CHUDEA3_2300	cgd3_2300	0.943	0.0694	0.0284	0.409221902
CHUDEA3_230	cgd3_230	0.979	0.0629	0.0089	0.141494436
CHUDEA3_2310	cgd3_2310	0.977	0.2164	0.0096	0.044362292
CHUDEA3_2320	cgd3_2320	0.991	0.0462	0.004	0.086580087
CHUDEA3_2330	cgd3_2330	0.971	0.0561	0.0126	0.22459893
CHUDEA3_2340	cgd3_2340	0.989	0.0543	0.0049	0.090239411
CHUDEA3_2350	cgd3_2350	0.97	0.0395	0.0139	0.351898734
CHUDEA3_2360	cgd3_2360	0.97	0.0868	0.0134	0.15437788
CHUDEA3_2370	cgd3_2370	0.983	0.0551	0.0079	0.143375681
CHUDEA3_2380	cgd3_2380	0.967	0.0896	0.0168	0.1875
CHUDEA3_2390	cgd3_2390	0.992	0.0835	0.0034	0.040718563
CHUDEA3_2400	cgd3_2400	0.971	0.046	0.0128	0.27826087
CHUDEA3_240	cgd3_240	0.959	0.0602	0.0182	0.302325581
CHUDEA3_2410	cgd3_2410	0.981	0.064	0.0085	0.1328125
CHUDEA3_2420	cgd3_2420	0.99	0.0149	0.0041	0.275167785
CHUDEA3_2430	cgd3_2430	0.977	0.0189	0.0108	0.571428571
CHUDEA3_2440	cgd3_2440	1	0.0786	0	0
CHUDEA3_2450	cgd3_2450	0.951	0.0785	0.021	0.267515924
CHUDEA3_2460	cgd3_2460	0.958	0.1095	0.0193	0.176255708
CHUDEA3_2470	cgd3_2470	0.977	0.0355	0.0086	0.242253521
CHUDEA3_2480	cgd3_2480	0.971	0.0379	0.0129	0.340369393
CHUDEA3_2490	cgd3_2490	0.982	0.0498	0.0077	0.154618474
CHUDEA3_2500	cgd3_2500	0.959	0.0618	0.0179	0.289644013
CHUDEA3_250	cgd3_250	0.991	0.0655	0.0028	0.042748092
CHUDEA3_2510	cgd3_2510	0.972	0.0596	0.0124	0.208053691
CHUDEA3_2520	cgd3_2520	0.963	0.0868	0.0164	0.188940092

CHUDEA3_2530	cgd3_2530	0.989	0.0964	0.0046	0.047717842
CHUDEA3_2540	cgd3_2540	1	0.0091	0	0
CHUDEA3_2550	cgd3_2550	0.966	0.0402	0.0178	0.44278607
CHUDEA3_2560	cgd3_2560	0.984	0.0463	0.0074	0.159827214
CHUDEA3_2570	cgd3_2570	0.967	0.0829	0.0139	0.167671894
CHUDEA3_2580	cgd3_2580	0.987	0.0694	0.0054	0.077809798
CHUDEA3_2590	cgd3_2590	0.98	0.0455	0.0099	0.217582418
CHUDEA3_2600	cgd3_2600	0.958	0.0536	0.0195	0.36380597
CHUDEA3_260	cgd3_260	0.986	0.0848	0.0058	0.068396226
CHUDEA3_2610	cgd3_2610	0.974	0.0533	0.0115	0.21575985
CHUDEA3_2620	cgd3_2620	0.994	0.0996	0.0028	0.02811245
CHUDEA3_2630	cgd3_2630	0.968	0.0435	0.0144	0.331034483
CHUDEA3_2640	cgd3_2640	0.961	0.0567	0.0167	0.294532628
CHUDEA3_2650	cgd3_2650	0.953	0.0807	0.0217	0.26889715
CHUDEA3_2660	cgd3_2660	0.98	0.0515	0.009	0.174757282
CHUDEA3_2670	cgd3_2670	0.98	0.0519	0.0092	0.177263969
CHUDEA3_2680	cgd3_2680	0.983	0.0584	0.0077	0.131849315
CHUDEA3_2690	cgd3_2690	0.973	0.0232	0.0126	0.543103448
CHUDEA3_2700	cgd3_2700	0.995	0.0342	0.0021	0.061403509
CHUDEA3_270	cgd3_270	0.967	0.1221	0.012	0.098280098
CHUDEA3_2710	cgd3_2710	0.963	0.0476	0.0165	0.346638655
CHUDEA3_2720	cgd3_2720	0.964	0.0371	0.0158	0.425876011
CHUDEA3_2730	cgd3_2730	0.986	0.07	0.0058	0.082857143
CHUDEA3_2740	cgd3_2740	0.961	0.0717	0.0178	0.248256625
CHUDEA3_2750	cgd3_2750	0.942	0.0683	0.0261	0.382137628
CHUDEA3_2760	cgd3_2760	0.964	0.0421	0.0182	0.432304038
CHUDEA3_2770	cgd3_2770	0.976	0.0649	0.0103	0.158705701
CHUDEA3_2780	cgd3_2780	0.98	0.0733	0.0085	0.115961801
CHUDEA3_2790	cgd3_2790	0.967	0.0426	0.014	0.328638498
CHUDEA3_2800	cgd3_2800	0.954	0.0483	0.0209	0.432712215
CHUDEA3_280	cgd3_280	0.962	0.1032	0.0115	0.111434109
CHUDEA3_2810	cgd3_2810	0.951	0.0557	0.0217	0.389587074
CHUDEA3_2820	cgd3_2820	0.972	0.0503	0.0127	0.252485089
CHUDEA3_2830	cgd3_2830	0.963	0.091	0.0158	0.173626374
CHUDEA3_2840	cgd3_2840	0.93	0.0377	0.0302	0.801061008
CHUDEA3_2850	cgd3_2850	0.984	0.0934	0.007	0.074946467
CHUDEA3_2860	cgd3_2860	0.96	0.0859	0.0109	0.126891735
CHUDEA3_2870	cgd3_2870	0.945	0.0486	0.0255	0.524691358
CHUDEA3_2880	cgd3_2880	0.98	0.0604	0.0089	0.147350993
CHUDEA3_2890	cgd3_2890	0.993	0.0612	0.0029	0.047385621
CHUDEA3_2900	cgd3_2900	0.975	0.0467	0.011	0.235546039
CHUDEA3_2910	cgd3_2910	0.973	0.0779	0.0114	0.146341463
CHUDEA3_2920	cgd3_2920	0.974	0.06	0.0116	0.193333333
CHUDEA3_2930	cgd3_2930	0.967	0.0426	0.0145	0.340375587
CHUDEA3_2940	cgd3_2940	0.972	0.0466	0.0127	0.272532189

CHUDEA3_2950	cgd3_2950	0.983	0.0297	0.0094	0.316498316
CHUDEA3_2960	cgd3_2960	0.97	0.0606	0.0134	0.221122112
CHUDEA3_2970	cgd3_2970	0.97	0.0687	0.0124	0.180494905
CHUDEA3_2980	cgd3_2980	0.961	0.0513	0.0179	0.348927875
CHUDEA3_2990	cgd3_2990	0.974	0.0627	0.0113	0.180223285
CHUDEA3_3000	cgd3_3000	0.974	0.0684	0.0117	0.171052632
CHUDEA3_300	cgd3_300	0.969	0.0576	0.0127	0.220486111
CHUDEA3_3010	cgd3_3010	0.957	0.0542	0.019	0.350553506
CHUDEA3_3020	cgd3_3020	0.994	0.0609	0.0026	0.042692939
CHUDEA3_3030	cgd3_3030	0.991	0.0715	0.004	0.055944056
CHUDEA3_3040	cgd3_3040	0.995	0.0408	0.0022	0.053921569
CHUDEA3_3050	cgd3_3050	0.938	0.0405	0.0294	0.725925926
CHUDEA3_3060	cgd3_3060	0.985	0.0495	0.0068	0.137373737
CHUDEA3_3070	cgd3_3070	0.951	0.0678	0.0221	0.325958702
CHUDEA3_3080	cgd3_3080	0.964	0.0549	0.0153	0.278688525
CHUDEA3_3090	cgd3_3090	0.972	0.0358	0.0123	0.343575419
CHUDEA3_30	cgd3_30	0.952	0.0723	0.022	0.30428769
CHUDEA3_3100	cgd3_3100	0.992	0.0467	0.0034	0.072805139
CHUDEA3_310	cgd3_310	0.991	0.0546	0.0037	0.067765568
CHUDEA3_3110	cgd3_3110	0.981	0.0616	0.0085	0.137987013
CHUDEA3_3120	cgd3_3120	0.967	0.0585	0.0153	0.261538462
CHUDEA3_3130	cgd3_3130	0.991	0.027	0.0042	0.155555556
CHUDEA3_3140	cgd3_3140	0.991	0.0375	0.004	0.106666667
CHUDEA3_3150	cgd3_3150	0.991	0.027	0.004	0.148148148
CHUDEA3_3160	cgd3_3160	0.968	0.0748	0.0146	0.195187166
CHUDEA3_3170	cgd3_3170	0.987	0.0815	0.0057	0.06993865
CHUDEA3_3180	cgd3_3180	0.984	0.058	0.0074	0.127586207
CHUDEA3_3190	cgd3_3190	0.988	0.067	0.0052	0.07761194
CHUDEA3_3200	cgd3_3200	0.988	0.0923	0.0054	0.058504875
CHUDEA3_320	cgd3_320	0.983	0.0725	0.0073	0.100689655
CHUDEA3_3210	cgd3_3210	0.988	0.061	0.0057	0.093442623
CHUDEA3_3220	cgd3_3220	0.978	0.0858	0.009	0.104895105
CHUDEA3_3230	cgd3_3230	0.978	0.0482	0.0098	0.203319502
CHUDEA3_3240	cgd3_3240	0.986	0.0755	0.006	0.079470199
CHUDEA3_3260	cgd3_3250	0.982	0.091	0.0082	0.09010989
CHUDEA3_3270	cgd3_3270	0.994	0.0818	0.0025	0.030562347
CHUDEA3_3280	cgd3_3280	0.987	0.0869	0.0054	0.062140391
CHUDEA3_3290	cgd3_3290	0.988	0.0829	0.0049	0.059107358
CHUDEA3_3300	cgd3_3300	0.983	0.0391	0.0081	0.207161125
CHUDEA3_330	cgd3_330	0.993	0.0792	0.003	0.037878788
CHUDEA3_3310	cgd3_3310	0.93	0.052	0.0268	0.515384615
CHUDEA3_3320	cgd3_3320	0.986	0.054	0.0061	0.112962963
CHUDEA3_3330	cgd3_3330	0.981	0.0518	0.0083	0.16023166
CHUDEA3_3340	cgd3_3340	0.977	0.0708	0.0094	0.132768362
CHUDEA3_3350	cgd3_3350	0.964	0.049	0.0172	0.351020408

CHUDEA3_3360	cgd3_3360	0.967	0.0563	0.0146	0.259325044
CHUDEA3_3370	cgd3_3370	0.934	0.0884	0.0339	0.383484163
CHUDEA3_3380	cgd3_3380	0.979	0.0616	0.0102	0.165584416
CHUDEA3_3390	cgd3_3390	0.963	0.0489	0.0173	0.353783231
CHUDEA3_3400	cgd3_3400	0.991	0.1233	0.0041	0.03325223
CHUDEA3_340	cgd3_340	0.984	0.0466	0.0075	0.160944206
CHUDEA3_3410	cgd3_3410	0.965	0.0573	0.0157	0.27399651
CHUDEA3_3420	cgd3_3420	0.97	0.0354	0.0119	0.336158192
CHUDEA3_3430	cgd3_3430	0.989	0.0639	0.0046	0.07198748
CHUDEA3_3440	cgd3_3440	0.991	0.0464	0.0038	0.081896552
CHUDEA3_3450	cgd3_3450	0.961	0.0655	0.0217	0.33129771
CHUDEA3_3460	cgd3_3460	0.981	0.0682	0.0083	0.12170088
CHUDEA3_3470	cgd3_3470	0.992	0.0774	0.003	0.03875969
CHUDEA3_3480	cgd3_3480	0.976	0.0521	0.0102	0.195777351
CHUDEA3_3490	cgd3_3490	0.973	0.0534	0.0114	0.213483146
CHUDEA3_3500	cgd3_3500	0.971	0.0791	0.012	0.1517067
CHUDEA3_350	cgd3_350	0.993	0.0494	0.0031	0.062753036
CHUDEA3_3510	cgd3_3510	0.95	0.1019	0.0229	0.224730128
CHUDEA3_3520	cgd3_3520	0.983	0.0675	0.0069	0.102222222
CHUDEA3_3530	cgd3_3530	0.974	0.05	0.0104	0.208
CHUDEA3_3550	cgd3_3550	0.982	0.0672	0.0067	0.099702381
CHUDEA3_3560	cgd3_3560	0.988	0.0894	0.0046	0.051454139
CHUDEA3_3570	cgd3_3570	0.961	0.0588	0.0171	0.290816327
CHUDEA3_3580	cgd3_3580	1	0.0542	0	0
CHUDEA3_3590	cgd3_3590	0.939	0.0627	0.0273	0.435406699
CHUDEA3_3600	cgd3_3600	0.994	0.0486	0.0032	0.065843621
CHUDEA3_360	cgd3_360	0.93	0.0607	0.0395	0.650741351
CHUDEA3_3610	cgd3_3610	0.987	0.0872	0.0062	0.071100917
CHUDEA3_3630	cgd3_3630	0.942	0.1622	0.0243	0.149815043
CHUDEA3_3640	cgd3_3640	0.973	0.0724	0.0077	0.106353591
CHUDEA3_3650	cgd3_3650	0.962	0.0593	0.0164	0.276559865
CHUDEA3_3660	cgd3_3660	0.988	0.0523	0.0051	0.09751434
CHUDEA3_3670	cgd3_3670	0.996	0.1281	0.0016	0.012490242
CHUDEA3_3680	cgd3_3680	0.97	0.057	0.0095	0.166666667
CHUDEA3_3690	cgd3_3690	0.989	0.0721	0.0053	0.073509015
CHUDEA3_3700	cgd3_3700	0.964	0.0806	0.0126	0.156327543
CHUDEA3_3710	cgd3_3710	0.974	0.0604	0.0094	0.155629139
CHUDEA3_3720	cgd3_3720	0.981	0.1036	0.01	0.096525097
CHUDEA3_3730	cgd3_3730	0.975	0.0398	0.0106	0.266331658
CHUDEA3_3740	cgd3_3740	0.981	0.0573	0.0073	0.127399651
CHUDEA3_3750	cgd3_3750	0.993	0.0535	0.0028	0.052336449
CHUDEA3_3760	cgd3_3760	0.994	0.0443	0.0022	0.0496614
CHUDEA3_3770	cgd3_3770	1	0.0306	0	0
CHUDEA3_3780	cgd3_3780	0.972	0.0767	0.012	0.156453716
CHUDEA3_3790	cgd3_3790	0.994	0.0528	0.0026	0.049242424

CHUDEA3_3800	cgd3_3800	0.987	0.0992	0.0051	0.05141129
CHUDEA3_380	cgd3_380	0.943	0.0767	0.0245	0.319426336
CHUDEA3_3810	cgd3_3810	0.993	0.0691	0.003	0.04341534
CHUDEA3_3820	cgd3_3820	0.983	0.0802	0.0079	0.098503741
CHUDEA3_3830	cgd3_3830	0.986	0.06	0.0058	0.096666667
CHUDEA3_3840	cgd3_3840	0.994	0.0534	0.0024	0.04494382
CHUDEA3_3850	cgd3_3850	0.992	0.078	0.004	0.051282051
CHUDEA3_3860	cgd3_3860	0.99	0.061	0.0048	0.078688525
CHUDEA3_3870	cgd3_3870	1	0.0764	0	0
CHUDEA3_3880	cgd3_3880	0.983	0.0523	0.0072	0.137667304
CHUDEA3_3890	cgd3_3890	1	0.0127	0	0
CHUDEA3_3900	cgd3_3900	0.964	0.0675	0.0153	0.226666667
CHUDEA3_390	cgd3_390	0.984	0.0726	0.008	0.110192837
CHUDEA3_3910	cgd3_3910	0.957	0.0499	0.0152	0.304609218
CHUDEA3_3920	cgd3_3920	0.992	0.0914	0.0031	0.033916849
CHUDEA3_3930	cgd3_3930	0.987	0.0643	0.0057	0.088646967
CHUDEA3_3940	cgd3_3940	0.986	0.0942	0.0055	0.058386412
CHUDEA3_3950	cgd3_3950	0.99	0.0432	0.0046	0.106481481
CHUDEA3_3960	cgd3_3960	0.955	0.0656	0.0192	0.292682927
CHUDEA3_3970	cgd3_3970	1	0.0687	0	0
CHUDEA3_3980	cgd3_3980	0.912	0.087	0.0294	0.337931034
CHUDEA3_3990	cgd3_3990	0.961	0.06	0.0179	0.298333333
CHUDEA3_4000	cgd3_4000	0.95	0.0749	0.021	0.280373832
CHUDEA3_400	cgd3_400	0.963	0.0561	0.0179	0.319073084
CHUDEA3_4010	cgd3_4010	0.992	0.0607	0.0035	0.057660626
CHUDEA3_4020	cgd3_4020	0.987	0.085	0.0054	0.063529412
CHUDEA3_4030	cgd3_4030	0.968	0.0425	0.016	0.376470588
CHUDEA3_4040	cgd3_4040	0.962	0.0978	0.0072	0.073619632
CHUDEA3_4050	cgd3_4050	0.985	0.0516	0.0064	0.124031008
CHUDEA3_4060	cgd3_4060	0.971	0.0639	0.0118	0.184663537
CHUDEA3_4070	cgd3_4070	0.992	0.0607	0.0036	0.059308072
CHUDEA3_4080	cgd3_4080	0.97	0.0646	0.0131	0.202786378
CHUDEA3_4090	cgd3_4090	0.97	0.0513	0.0135	0.263157895
CHUDEA3_40	cgd3_40	0.988	0.0699	0.0054	0.077253219
CHUDEA3_4100	cgd3_4100	1	0.0549	0	0
CHUDEA3_410	cgd3_410	0.974	0.0578	0.0109	0.188581315
CHUDEA3_4110	cgd3_4110	0.976	0.0719	0.0121	0.168289291
CHUDEA3_4120	cgd3_4120	0.982	0.04	0.0079	0.1975
CHUDEA3_4130	cgd3_4130	0.974	0.0695	0.012	0.172661871
CHUDEA3_4140	cgd3_4140	0.962	0.0772	0.0184	0.238341969
CHUDEA3_4150	cgd3_4150	0.914	0.1638	0.0352	0.214896215
CHUDEA3_4160	cgd3_4160	0.942	0.0686	0.0084	0.12244898
CHUDEA3_4170	cgd3_4170	0.974	0.0669	0.0119	0.177877429
CHUDEA3_4180	cgd3_4180	0.928	0.0613	0.024	0.391517129
CHUDEA3_4190	cgd3_4190	0.913	0.1025	0.0408	0.39804878

CHUDEA3_4200	cgd3_4200	0.978	0.0728	0.0095	0.130494505
CHUDEA3_420	cgd3_420	0.975	0.0394	0.0112	0.284263959
CHUDEA3_4210	cgd3_4210	0.98	0.0512	0.0099	0.193359375
CHUDEA3_4220	cgd3_4220	0.97	0.058	0.0143	0.246551724
CHUDEA3_4230	cgd3_4230	0.946	0.0784	0.0262	0.334183673
CHUDEA3_4240	cgd3_4240	0.968	0.0807	0.0146	0.180916976
CHUDEA3_4250	cgd3_4250	0.926	0.0937	0.0301	0.321237994
CHUDEA3_4260	cgd3_4260	0.875	0.1875	0.0621	0.3312
CHUDEA3_4270	cgd3_4270	0.895	0.1716	0.0511	0.297785548
CHUDEA3_4280	cgd3_4280	0.947	0.0801	0.024	0.299625468
CHUDEA3_4290	cgd3_4290	0.97	0.0828	0.0135	0.163043478
CHUDEA3_4300	cgd3_4300	0.973	0.0466	0.0124	0.266094421
CHUDEA3_430	cgd3_430	0.973	0.0514	0.0113	0.219844358
CHUDEA3_4310	cgd3_4310	0.943	0.0774	0.0213	0.275193798
CHUDEA3_4320	cgd3_4320	0.983	0.0761	0.0074	0.097240473
CHUDEA3_4330	cgd3_4330	0.966	0.0873	0.0147	0.16838488
CHUDEA3_4340	cgd3_4340	0.941	0.0851	0.026	0.305522914
CHUDEA3_4350	cgd3_4350	0.97	0.0467	0.0131	0.280513919
CHUDEA3_4360	cgd3_4360	0.901	0.0978	0.046	0.470347648
CHUDEA3_440	cgd3_440	0.964	0.0923	0.0121	0.131094258
CHUDEA3_450	cgd3_450	0.995	0.0507	0.0033	0.065088757
CHUDEA3_460	cgd3_460	0.995	0.097	0.0021	0.021649485
CHUDEA3_470	cgd3_470	0.987	0.0722	0.0062	0.085872576
CHUDEA3_480	cgd3_480	0.973	0.0957	0.0117	0.122257053
CHUDEA3_490	cgd3_490	0.995	0.0412	0.0021	0.050970874
CHUDEA3_500	cgd3_500	0.992	0.0769	0.0035	0.045513654
CHUDEA3_50	cgd3_50	0.979	0.0716	0.0088	0.122905028
CHUDEA3_510	cgd3_510	0.997	0.0496	0.0012	0.024193548
CHUDEA3_520	cgd3_520	0.982	0.0897	0.0077	0.085841695
CHUDEA3_530	cgd3_530	0.962	0.0627	0.015	0.23923445
CHUDEA3_540	cgd3_540	0.996	0.0701	0.0017	0.02425107
CHUDEA3_550	cgd3_550	0.978	0.096	0.0108	0.1125
CHUDEA3_560	cgd3_560	0.985	0.0496	0.0064	0.129032258
CHUDEA3_570	cgd3_570	0.964	0.0173	0.0185	1.069364162
CHUDEA3_580	cgd3_580	0.967	0.052	0.0145	0.278846154
CHUDEA3_590	cgd3_590	0.984	0.0557	0.0073	0.131059246
CHUDEA3_600	cgd3_600	0.989	0.0676	0.0048	0.071005917
CHUDEA3_60	cgd3_60	0.96	0.0241	0.0189	0.784232365
CHUDEA3_610	cgd3_610	0.992	0.0929	0.0023	0.024757804
CHUDEA3_640	cgd3_640	0.991	0.0731	0.0034	0.046511628
CHUDEA3_650	cgd3_650	0.978	0.0481	0.0091	0.189189189
CHUDEA3_660	cgd3_660	0.964	0.0569	0.0167	0.293497364
CHUDEA3_670	cgd3_670	0.991	0.0454	0.0037	0.081497797
CHUDEA3_680	cgd3_680	0.965	0.0599	0.0145	0.242070117
CHUDEA3_690	cgd3_690	0.991	0.057	0.004	0.070175439

CHUDEA3_70	cgd3_70	0.977	0.0907	0.0101	0.111356119
CHUDEA3_710	cgd3_710	0.957	0.075	0.0186	0.248
CHUDEA3_730	cgd3_730	0.962	0.0778	0.0184	0.236503856
CHUDEA3_740	cgd3_740	0.989	0.0497	0.0049	0.098591549
CHUDEA3_750	cgd3_750	0.974	0.0574	0.0102	0.177700348
CHUDEA3_760	cgd3_760	1	0.0344	0	0
CHUDEA3_770	cgd3_770	0.976	0.05	0.011	0.22
CHUDEA3_780	cgd3_780	0.996	0.0516	0.0017	0.032945736
CHUDEA3_790	cgd3_790	0.978	0.0582	0.0105	0.180412371
CHUDEA3_800	cgd3_800	0.937	0.0514	0.0298	0.579766537
CHUDEA3_80	cgd3_80	0.992	0.0667	0.0034	0.050974513
CHUDEA3_810	cgd3_810	0.953	0.0646	0.0192	0.297213622
CHUDEA3_820	cgd3_820	0.887	0.0654	0.0517	0.790519878
CHUDEA3_830	cgd3_830	1	0.0269	0	0
CHUDEA3_840	cgd3_840	0.995	0.0535	0.0021	0.039252336
CHUDEA3_850	cgd3_850	0.99	0.0824	0.0046	0.055825243
CHUDEA3_860	cgd3_860	0.995	0.0392	0.0024	0.06122449
CHUDEA3_870	cgd3_870	0.995	0.0632	0.0023	0.036392405
CHUDEA3_880	cgd3_880	1	0.0665	0	0
CHUDEA3_890	cgd3_890	0.982	0.0732	0.0074	0.101092896
CHUDEA3_900	cgd3_900	0.972	0.0443	0.0121	0.273137698
CHUDEA3_90	cgd3_90	0.973	0.0451	0.0118	0.261640798
CHUDEA3_910	cgd3_910	0.996	0.0663	0.0016	0.02413273
CHUDEA3_920	cgd3_920	0.989	0.0516	0.0047	0.091085271
CHUDEA3_930	cgd3_930	0.97	0.0659	0.0127	0.192716237
CHUDEA3_940	cgd3_940	0.981	0.1095	0.0074	0.067579909
CHUDEA3_950	cgd3_950	0.966	0.061	0.0148	0.242622951
CHUDEA3_960	cgd3_960	0.957	0.0529	0.0185	0.349716446
CHUDEA3_970	cgd3_970	0.981	0.0625	0.0078	0.1248
CHUDEA3_980	cgd3_980	0.996	0.0786	0.0015	0.019083969
CHUDEA3_990	cgd3_990	0.957	0.0386	0.0209	0.541450777
CHUDEA3_new_01	cgd3_new_01	0.992	0.0592	0.0036	0.060810811
CHUDEA3_new_02	cgd3_new_02	0.919	0.0492	0.0367	0.745934959
CHUDEA3_new_03	cgd3_new_03	0.995	0.0565	0.0025	0.044247788
CHUDEA3_new_04	cgd3_new_04	0.951	0.0252	0.0222	0.880952381
CHUDEA3_new_05	cgd3_new_05	0.991	0.0904	0.0042	0.046460177
CHUDEA3_new_06	cgd3_new_06	0.974	0.0542	0.0114	0.210332103
CHUDEA3_new_07	cgd3_new_07	0.951	0.0237	0.0238	1.004219409
CHUDEA3_new_08	cgd3_new_08	0.954	0.0331	0.0212	0.640483384
CHUDEA3_new_09	cgd3_new_09	0.983	0.0748	0.0077	0.102941176
CHUDEA4_100	cgd4_100	0.969	0.0648	0.0137	0.211419753
CHUDEA4_1010	cgd4_1010	0.924	0.0635	0.0211	0.332283465
CHUDEA4_1030	cgd4_1030	0.989	0.085	0.0044	0.051764706
CHUDEA4_1040	cgd4_1040	0.986	0.0874	0.0054	0.061784897
CHUDEA4_1070	cgd4_1070	0.991	0.0495	0.0035	0.070707071

CHUDEA4_1080	cgd4_1080	0.984	0.0737	0.0064	0.086838535
CHUDEA4_1090	cgd4_1090	0.977	0.0678	0.0096	0.14159292
CHUDEA4_10	cgd4_10	0.869	0.0919	0.0592	0.644178455
CHUDEA4_1100	cgd4_1100	0.973	0.0546	0.011	0.201465201
CHUDEA4_110	cgd4_110	0.974	0.0741	0.0111	0.149797571
CHUDEA4_1110	cgd4_1110	0.957	0.0564	0.0152	0.269503546
CHUDEA4_1120	cgd4_1120	0.973	0.082	0.0127	0.154878049
CHUDEA4_1130	cgd4_1130	0.969	0.0573	0.0137	0.239092496
CHUDEA4_1140	cgd4_1140	0.953	0.0887	0.0197	0.222096956
CHUDEA4_1150	cgd4_1150	0.975	0.0317	0.0103	0.324921136
CHUDEA4_1160	cgd4_1160	0.985	0.0847	0.0059	0.069657615
CHUDEA4_1170	cgd4_1170	1	0.0879	0	0
CHUDEA4_1180	cgd4_1180	0.973	0.0137	0.0118	0.861313869
CHUDEA4_1190	cgd4_1190	0.985	0.1053	0.0058	0.055080722
CHUDEA4_1200	cgd4_1200	0.997	0.0834	0.0013	0.01558753
CHUDEA4_120	cgd4_120	0.976	0.0606	0.0103	0.169966997
CHUDEA4_1210	cgd4_1210	0.979	0.0634	0.0081	0.127760252
CHUDEA4_1220	cgd4_1220	0.977	0.086	0.0096	0.111627907
CHUDEA4_1230	cgd4_1230	0.976	0.0084	0.0116	1.380952381
CHUDEA4_1240	cgd4_1240	0.965	0.0654	0.0154	0.235474006
CHUDEA4_1250	cgd4_1250	0.98	0.0824	0.008	0.097087379
CHUDEA4_1260	cgd4_1260	0.981	0.0465	0.0078	0.167741935
CHUDEA4_1270	cgd4_1270	0.982	0.068	0.0076	0.111764706
CHUDEA4_1280	cgd4_1280	0.991	0.0312	0.0038	0.121794872
CHUDEA4_1300	cgd4_1300	0.681	0.2378	0.0878	0.36921783
CHUDEA4_130	cgd4_130	0.968	0.0441	0.0123	0.278911565
CHUDEA4_1310	cgd4_1310	0.97	0.0795	0.0118	0.148427673
CHUDEA4_1330	cgd4_1330	0.983	0.0793	0.0065	0.081967213
CHUDEA4_1340	cgd4_1340	0.938	0.09	0.0085	0.094444444
CHUDEA4_1350	cgd4_1350	0.919	0.0675	0.0177	0.262222222
CHUDEA4_1360	cgd4_1360	0.974	0.0695	0.0091	0.130935252
CHUDEA4_1370	cgd4_1370	0.979	0.0742	0.009	0.121293801
CHUDEA4_1380	cgd4_1380	0.976	0.0729	0.0108	0.148148148
CHUDEA4_1390	cgd4_1390	0.974	0.0499	0.0115	0.230460922
CHUDEA4_1400	cgd4_1400	0.992	0.1192	0.0031	0.026006711
CHUDEA4_140	cgd4_140	0.977	0.0674	0.0072	0.106824926
CHUDEA4_1410	cgd4_1410	0.962	0.0421	0.0122	0.289786223
CHUDEA4_1420	cgd4_1420	0.973	0.0452	0.0069	0.152654867
CHUDEA4_1430	cgd4_1430	0.976	0.078	0.0087	0.111538462
CHUDEA4_1440	cgd4_1440	0.982	0.0502	0.0085	0.169322709
CHUDEA4_1450	cgd4_1450	0.951	0.0775	0.0106	0.136774194
CHUDEA4_1460	cgd4_1460	0.986	0.0623	0.0059	0.09470305
CHUDEA4_1470	cgd4_1470	0.997	0.0317	0.001	0.031545741
CHUDEA4_1480	cgd4_1480	0.978	0.0535	0.0056	0.104672897
CHUDEA4_1490	cgd4_1490	0.997	0.0424	0.001	0.023584906

CHUDEA4_1500	cgd4_1500	0.968	0.0417	0.0134	0.321342926
CHUDEA4_150	cgd4_150	0.986	0.0756	0.0053	0.07010582
CHUDEA4_1510	cgd4_1510	0.98	0.0512	0.0077	0.150390625
CHUDEA4_1520	cgd4_1520	0.972	0.0501	0.0102	0.203592814
CHUDEA4_1530	cgd4_1530	0.972	0.0693	0.0092	0.132756133
CHUDEA4_1540	cgd4_1540	0.96	0.0816	0.0166	0.203431373
CHUDEA4_1550	cgd4_1550	0.993	0.1125	0	0
CHUDEA4_1560	cgd4_1560	0.953	0.0523	0.0157	0.300191205
CHUDEA4_1570	cgd4_1570	0.957	0.0327	0.0186	0.568807339
CHUDEA4_1580	cgd4_1580	0.986	0.0529	0.0055	0.103969754
CHUDEA4_1590	cgd4_1590	0.975	0.0671	0.0105	0.156482861
CHUDEA4_1600	cgd4_1600	0.971	0.0758	0.0109	0.143799472
CHUDEA4_160	cgd4_160	0.949	0.0932	0.0249	0.267167382
CHUDEA4_1610	cgd4_1610	0.965	0.0628	0.0121	0.192675159
CHUDEA4_1620	cgd4_1620	0.98	0.0295	0.0091	0.308474576
CHUDEA4_1630	cgd4_1630	0.967	0.0695	0.0142	0.204316547
CHUDEA4_1640	cgd4_1640	0.939	0.063	0.014	0.222222222
CHUDEA4_1650	cgd4_1650	0.981	0.0985	0.0089	0.09035533
CHUDEA4_1660	cgd4_1660	0.978	0.0472	0.0096	0.203389831
CHUDEA4_1670	cgd4_1670	0.935	0.1457	0.0277	0.190116678
CHUDEA4_1680	cgd4_1680	0.976	0.0827	0.0087	0.105199516
CHUDEA4_1690	cgd4_1690	0.914	0.079	0.0105	0.132911392
CHUDEA4_1700	cgd4_1700	0.999	0.0807	0.0006	0.007434944
CHUDEA4_170	cgd4_170	0.981	0.0219	0.0073	0.333333333
CHUDEA4_1710	cgd4_1710	0.974	0.055	0.0112	0.203636364
CHUDEA4_1720	cgd4_1720	0.983	0.0576	0.0068	0.118055556
CHUDEA4_1730	cgd4_1730	0.973	0.0601	0.0109	0.181364393
CHUDEA4_1740	cgd4_1740	0.947	0.0512	0.0237	0.462890625
CHUDEA4_1760	cgd4_1760	0.963	0.0537	0.013	0.242085661
CHUDEA4_1770	cgd4_1770	0.996	0.0715	0.0017	0.023776224
CHUDEA4_1780	cgd4_1780	0.962	0.0964	0.0161	0.167012448
CHUDEA4_1790	cgd4_1790	0.975	0.0304	0.0071	0.233552632
CHUDEA4_1800	cgd4_1800	0.986	0.053	0.0059	0.111320755
CHUDEA4_180	cgd4_180	0.973	0.0576	0.0121	0.210069444
CHUDEA4_1810	cgd4_1810	0.974	0.0263	0.0111	0.422053232
CHUDEA4_1820	cgd4_1820	0.989	0.0687	0.0043	0.062590975
CHUDEA4_1830	cgd4_1830	1	0.0954	0	0
CHUDEA4_1840	cgd4_1840	0.99	0.064	0.004	0.0625
CHUDEA4_1850	cgd4_1850	0.98	0.0664	0.0089	0.134036145
CHUDEA4_1860	cgd4_1860	0.979	0.0539	0.0093	0.172541744
CHUDEA4_1870	cgd4_1870	0.973	0.0366	0.0115	0.31420765
CHUDEA4_1880	cgd4_1880	0.981	0.059	0.008	0.13559322
CHUDEA4_1890	cgd4_1890	0.985	0.0439	0.0077	0.175398633
CHUDEA4_1900	cgd4_1900	0.951	0.0189	0.0221	1.169312169
CHUDEA4_190	cgd4_190	0.98	0.0656	0.0098	0.149390244

CHUDEA4_1910	cgd4_1910	0.99	0.0515	0.0051	0.099029126
CHUDEA4_1920	cgd4_1920	0.992	0.0371	0.0035	0.094339623
CHUDEA4_1930	cgd4_1930	0.986	0.0578	0.0067	0.115916955
CHUDEA4_1940	cgd4_1940	0.993	0.0424	0.003	0.070754717
CHUDEA4_1950	cgd4_1950	0.967	0.0824	0.0148	0.17961165
CHUDEA4_1960	cgd4_1960	0.969	0.0819	0.0135	0.164835165
CHUDEA4_1970	cgd4_1970	0.952	0.1143	0.0037	0.032370954
CHUDEA4_1980	cgd4_1980	0.986	0.068	0.0057	0.083823529
CHUDEA4_1990	cgd4_1990	0.972	0.0299	0.0142	0.474916388
CHUDEA4_2000	cgd4_2000	0.957	0.0242	0.0198	0.818181818
CHUDEA4_200	cgd4_200	0.927	0.1243	0.0195	0.15687852
CHUDEA4_2010	cgd4_2010	0.975	0.0853	0.0111	0.130128957
CHUDEA4_2020	cgd4_2020	0.99	0.0675	0.0037	0.054814815
CHUDEA4_2030	cgd4_2030	1	0.0628	0	0
CHUDEA4_2040	cgd4_2040	0.969	0.0704	0.013	0.184659091
CHUDEA4_2050	cgd4_2050	0.985	0.1103	0.0062	0.056210335
CHUDEA4_2060	cgd4_2060	0.98	0.0984	0.0086	0.087398374
CHUDEA4_2070	cgd4_2070	0.97	0.0735	0.013	0.176870748
CHUDEA4_2080	cgd4_2080	0.985	0.0524	0.0063	0.120229008
CHUDEA4_2090	cgd4_2090	0.979	0.0516	0.0062	0.120155039
CHUDEA4_20	cgd4_20	0.976	0.0596	0.0099	0.166107383
CHUDEA4_2100	cgd4_2100	0.989	0.0761	0.0051	0.067017083
CHUDEA4_210	cgd4_210	0.99	0.0539	0.0044	0.081632653
CHUDEA4_2110	cgd4_2110	0.983	0.0487	0.0077	0.158110883
CHUDEA4_2120	cgd4_2120	0.978	0.0559	0.0096	0.171735242
CHUDEA4_2130	cgd4_2130	0.991	0.0485	0.004	0.082474227
CHUDEA4_2140	cgd4_2140	0.99	0.0751	0.0045	0.059920107
CHUDEA4_2150	cgd4_2150	0.961	0.0441	0.0174	0.394557823
CHUDEA4_2160	cgd4_2160	0.979	0.0612	0.0083	0.135620915
CHUDEA4_2170	cgd4_2170	0.96	0.051	0.0189	0.370588235
CHUDEA4_2180	cgd4_2180	0.967	0.0393	0.0152	0.386768448
CHUDEA4_2190	cgd4_2190	0.945	0.0336	0.0268	0.797619048
CHUDEA4_2200	cgd4_2200	0.977	0.0623	0.0102	0.163723917
CHUDEA4_220	cgd4_220	0.974	0.0487	0.0117	0.240246407
CHUDEA4_2210	cgd4_2210	0.97	0.0609	0.0132	0.216748768
CHUDEA4_2220	cgd4_2220	0.977	0.0734	0.01	0.136239782
CHUDEA4_2230	cgd4_2230	0.984	0.0812	0.0071	0.087438424
CHUDEA4_2240	cgd4_2240	0.964	0.0481	0.0162	0.336798337
CHUDEA4_2250	cgd4_2250	0.974	0.068	0.0117	0.172058824
CHUDEA4_2260	cgd4_2260	0.994	0.0491	0.0031	0.063136456
CHUDEA4_2270	cgd4_2270	0.966	0.0671	0.0163	0.242921013
CHUDEA4_2280	cgd4_2280	0.984	0.0641	0.0068	0.106084243
CHUDEA4_2290	cgd4_2290	0.993	0.0394	0.003	0.076142132
CHUDEA4_2300	cgd4_2300	0.99	0.0803	0.0051	0.063511831
CHUDEA4_230	cgd4_230	0.995	0.0453	0.003	0.066225166

CHUDEA4_2310	cgd4_2310	0.943	0.0345	0.0247	0.715942029
CHUDEA4_2320	cgd4_2320	0.972	0.0784	0.0115	0.146683673
CHUDEA4_2330	cgd4_2330	0.971	0.1163	0.0127	0.109200344
CHUDEA4_2340	cgd4_2340	0.973	0.0465	0.012	0.258064516
CHUDEA4_2350	cgd4_2350	0.959	0.0854	0	0
CHUDEA4_2360	cgd4_2360	0.963	0.097	0.0149	0.153608247
CHUDEA4_2370	cgd4_2370	0.994	0.051	0.0025	0.049019608
CHUDEA4_2380	cgd4_2380	0.972	0.0488	0.0148	0.303278689
CHUDEA4_2390	cgd4_2390	0.993	0.0461	0.0035	0.075921909
CHUDEA4_2400	cgd4_2400	1	0.0516	0	0
CHUDEA4_240	cgd4_240	1	0.0415	0	0
CHUDEA4_2410	cgd4_2410	0.958	0.0668	0.0176	0.263473054
CHUDEA4_2420	cgd4_2420	0.978	0.062	0.0093	0.15
CHUDEA4_2430	cgd4_2430	0.964	0.0666	0.0148	0.222222222
CHUDEA4_2440	cgd4_2440	0.985	0.0667	0.0085	0.127436282
CHUDEA4_2450	cgd4_2450	0.959	0.0429	0.0224	0.522144522
CHUDEA4_2460	cgd4_2460	0.977	0.0836	0.0106	0.126794258
CHUDEA4_2470	cgd4_2470	0.957	0.0744	0.0189	0.254032258
CHUDEA4_2480	cgd4_2480	0.97	0.1362	0.0135	0.099118943
CHUDEA4_2490	cgd4_2490	0.983	0.0798	0.0079	0.098997494
CHUDEA4_2500	cgd4_2500	0.964	0.092	0.016	0.173913043
CHUDEA4_250	cgd4_250	0.992	0.0342	0.0032	0.093567251
CHUDEA4_2510	cgd4_2510	0.895	0.0612	0.0504	0.823529412
CHUDEA4_2520	cgd4_2520	0.934	0.0503	0.0295	0.586481113
CHUDEA4_2530	cgd4_2530	0.952	0.0965	0.0204	0.211398964
CHUDEA4_2540	cgd4_2540	0.99	0.0487	0.0043	0.088295688
CHUDEA4_2550	cgd4_2550	0.964	0.0841	0.0184	0.218787158
CHUDEA4_2560	cgd4_2560	0.985	0.0488	0.0061	0.125
CHUDEA4_2570	cgd4_2570	0.964	0.0634	0.0159	0.250788644
CHUDEA4_2580	cgd4_2580	0.983	0.0477	0.0078	0.163522013
CHUDEA4_2590	cgd4_2590	0.974	0.0561	0.0123	0.219251337
CHUDEA4_2600	cgd4_2600	0.994	0.0686	0.0025	0.036443149
CHUDEA4_260	cgd4_260	0.987	0.0589	0.0053	0.089983022
CHUDEA4_2610	cgd4_2610	0.991	0.0479	0.0038	0.079331942
CHUDEA4_2620	cgd4_2620	0.944	0.0535	0.0249	0.465420561
CHUDEA4_2630	cgd4_2630	0.957	0.0576	0.0186	0.322916667
CHUDEA4_2640	cgd4_2640	0.952	0.0707	0.0229	0.323903819
CHUDEA4_2650	cgd4_2650	0.987	0.0622	0.0053	0.085209003
CHUDEA4_2660	cgd4_2660	0.964	0.0643	0.0152	0.236391913
CHUDEA4_2670	cgd4_2670	0.973	0.0551	0.0121	0.219600726
CHUDEA4_2680	cgd4_2680	0.976	0.0267	0.0109	0.4082397
CHUDEA4_2690	cgd4_2690	0.97	0.064	0.0128	0.2
CHUDEA4_2700	cgd4_2700	0.972	0.0524	0.0125	0.238549618
CHUDEA4_270	cgd4_270	0.979	0.086	0.0097	0.112790698
CHUDEA4_2710	cgd4_2710	1	0.1208	0	0

CHUDEA4_2720	cgd4_2720	0.986	0.071	0.0062	0.087323944
CHUDEA4_2730	cgd4_2730	0.971	0.0604	0.0132	0.218543046
CHUDEA4_2740	cgd4_2740	0.965	0.045	0.0155	0.344444444
CHUDEA4_2750	cgd4_2750	0.974	0.0821	0.0111	0.135200974
CHUDEA4_2770	cgd4_2770	0.98	0.07	0.0099	0.141428571
CHUDEA4_2780	cgd4_2780	0.986	0.0699	0.0062	0.08869814
CHUDEA4_2790	cgd4_2790	0.967	0.0546	0.0159	0.291208791
CHUDEA4_2800	cgd4_2800	0.987	0.0593	0.0059	0.099494098
CHUDEA4_280	cgd4_280	0.978	0.0741	0.0092	0.124156545
CHUDEA4_2810	cgd4_2810	0.98	0.0391	0.0088	0.225063939
CHUDEA4_2820	cgd4_2820	0.945	0.0515	0.0249	0.483495146
CHUDEA4_2830	cgd4_2830	0.967	0.0385	0.0152	0.394805195
CHUDEA4_2840	cgd4_2840	0.988	0.0382	0.0055	0.143979058
CHUDEA4_2850	cgd4_2850	0.969	0.0382	0.0147	0.384816754
CHUDEA4_2860	cgd4_2860	1	0.0743	0	0
CHUDEA4_2870	cgd4_2870	0.975	0.033	0.0116	0.351515152
CHUDEA4_2880	cgd4_2880	0.979	0.0543	0.0094	0.173112339
CHUDEA4_2890	cgd4_2890	0.969	0.0421	0.0145	0.344418052
CHUDEA4_2900	cgd4_2900	0.968	0.0701	0.0137	0.195435093
CHUDEA4_290	cgd4_290	0.98	0.0742	0.0084	0.113207547
CHUDEA4_2910	cgd4_2910	0.981	0.0564	0.0085	0.15070922
CHUDEA4_2920	cgd4_2920	0.965	0.0498	0.0136	0.273092369
CHUDEA4_2930	cgd4_2930	0.95	0.0497	0.0235	0.472837022
CHUDEA4_2940	cgd4_2940	0.991	0.0431	0.0045	0.104408353
CHUDEA4_2950	cgd4_2950	0.986	0.0547	0.006	0.109689214
CHUDEA4_2960	cgd4_2960	0.984	0.0531	0.0059	0.111111111
CHUDEA4_2970	cgd4_2970	0.97	0.0258	0.0128	0.496124031
CHUDEA4_2980	cgd4_2980	0.983	0.0568	0.0037	0.065140845
CHUDEA4_2990	cgd4_2990	0.969	0.0495	0.0155	0.313131313
CHUDEA4_3000	cgd4_3000	0.99	0.0681	0.0044	0.064610866
CHUDEA4_300	cgd4_300	0.994	0.0654	0.0012	0.018348624
CHUDEA4_3010	cgd4_3010	0.967	0.0453	0.0144	0.317880795
CHUDEA4_3020	cgd4_3020	0.966	0.0382	0.0172	0.45026178
CHUDEA4_3030	cgd4_3030	0.97	0.0742	0.0134	0.180592992
CHUDEA4_3040	cgd4_3040	0.998	0.0922	0.001	0.010845987
CHUDEA4_3050	cgd4_3050	0.959	0.1022	0.0196	0.191780822
CHUDEA4_3070	cgd4_3070	0.972	0.0391	0.0138	0.352941176
CHUDEA4_3080	cgd4_3080	0.974	0.0553	0.012	0.216998192
CHUDEA4_3090	cgd4_3090	0.994	0.0887	0.0025	0.028184893
CHUDEA4_30	cgd4_30	0.98	0.101	0.0085	0.084158416
CHUDEA4_3100	cgd4_3100	0.991	0.0669	0.0036	0.053811659
CHUDEA4_310	cgd4_310	0.95	0.0487	0.02	0.410677618
CHUDEA4_3110	cgd4_3110	0.974	0.0659	0.0113	0.171471927
CHUDEA4_3120	cgd4_3120	0.97	0.0355	0.0137	0.385915493
CHUDEA4_3130	cgd4_3130	0.996	0.0856	0.0017	0.019859813

CHUDEA4_3140	cgd4_3140	0.974	0.0537	0.0123	0.229050279
CHUDEA4_3150	cgd4_3150	0.971	0.0472	0.0143	0.302966102
CHUDEA4_3160	cgd4_3160	0.992	0.061	0.0034	0.055737705
CHUDEA4_3170	cgd4_3170	0.974	0.0712	0.0106	0.148876404
CHUDEA4_3180	cgd4_3180	0.986	0.0446	0.0063	0.141255605
CHUDEA4_3190	cgd4_3190	1	0.0863	0	0
CHUDEA4_31	cgd4_31	0.849	0.0222	0.0746	3.36036036
CHUDEA4_3200	cgd4_3200	0.98	0.0658	0.0088	0.133738602
CHUDEA4_320	cgd4_320	1	0.0692	0	0
CHUDEA4_3210	cgd4_3210	0.978	0.0658	0.0101	0.153495441
CHUDEA4_3220	cgd4_3220	1	0.0197	0	0
CHUDEA4_3230	cgd4_3230	0.99	0.0465	0.0045	0.096774194
CHUDEA4_3240	cgd4_3240	0.98	0.057	0.0091	0.159649123
CHUDEA4_3250	cgd4_3250	0.981	0.0726	0.008	0.110192837
CHUDEA4_3260	cgd4_3260	1	0.0214	0	0
CHUDEA4_3270	cgd4_3270	0.996	0.0485	0.0017	0.035051546
CHUDEA4_3280	cgd4_3280	0.963	0.0677	0.0154	0.227474151
CHUDEA4_3290	cgd4_3290	1	0.0349	0	0
CHUDEA4_32	cgd4_32	0.899	0.0799	0.0422	0.5281602
CHUDEA4_3300	cgd4_3300	1	0.0444	0	0
CHUDEA4_330	cgd4_330	0.98	0.0372	0.0087	0.233870968
CHUDEA4_3310	cgd4_3310	0.969	0.0569	0.0139	0.244288225
CHUDEA4_3320	cgd4_3320	0.977	0.0642	0.0102	0.158878505
CHUDEA4_3330	cgd4_3330	0.995	0.0575	0.0022	0.03826087
CHUDEA4_3340	cgd4_3340	0.984	0.0718	0.0068	0.094707521
CHUDEA4_3350	cgd4_3350	0.997	0.0381	0.0012	0.031496063
CHUDEA4_3360	cgd4_3360	0.984	0.0742	0.0068	0.091644205
CHUDEA4_3370	cgd4_3370	0.97	0.0417	0.0135	0.323741007
CHUDEA4_3380	cgd4_3380	0.984	0.0653	0.0071	0.108728943
CHUDEA4_3390	cgd4_3390	0.981	0.0641	0.0087	0.135725429
CHUDEA4_3400	cgd4_3400	0.987	0.0476	0.006	0.12605042
CHUDEA4_340	cgd4_340	0.975	0.0448	0.0112	0.25
CHUDEA4_3410	cgd4_3410	0.971	0.0507	0.0125	0.246548323
CHUDEA4_3420	cgd4_3420	0.977	0.0342	0.0103	0.301169591
CHUDEA4_3430	cgd4_3430	0.976	0.0652	0.0098	0.150306748
CHUDEA4_3440	cgd4_3440	0.979	0.039	0.0091	0.233333333
CHUDEA4_3450	cgd4_3450	0.97	0.0606	0.0115	0.189768977
CHUDEA4_3460	cgd4_3460	0.924	0.0832	0.0329	0.395432692
CHUDEA4_3470	cgd4_3470	0.955	0.0512	0.0195	0.380859375
CHUDEA4_3480	cgd4_3480	0.965	0.0615	0.0141	0.229268293
CHUDEA4_3490	cgd4_3490	0.974	0.0769	0.0104	0.135240572
CHUDEA4_3500	cgd4_3500	0.906	0.0263	0.0433	1.646387833
CHUDEA4_350	cgd4_350	0.989	0.046	0.0049	0.106521739
CHUDEA4_3510	cgd4_3510	0.981	0.0904	0.0096	0.10619469
CHUDEA4_3520	cgd4_3520	0.889	0.1146	0.0321	0.280104712

CHUDEA4_3530	cgd4_3530	0.965	0.0933	0.0163	0.174705252
CHUDEA4_3540	cgd4_3540	0.971	0.0537	0.0125	0.232774674
CHUDEA4_3550	cgd4_3550	0.881	0.1094	0.0283	0.258683729
CHUDEA4_3560	cgd4_3560	0.959	0.0573	0.0165	0.287958115
CHUDEA4_3570	cgd4_3570	0.965	0.047	0.0132	0.280851064
CHUDEA4_3580	cgd4_3580	0.973	0.0864	0.0106	0.122685185
CHUDEA4_3590	cgd4_3590	0.958	0.0757	0.0197	0.260237781
CHUDEA4_3600	cgd4_3600	0.981	0.0509	0.008	0.157170923
CHUDEA4_360	cgd4_360	0.988	0.0565	0.0056	0.099115044
CHUDEA4_3610	cgd4_3610	0.96	0.0568	0.0185	0.325704225
CHUDEA4_3620	cgd4_3620	0.973	0.0389	0.0133	0.341902314
CHUDEA4_3630	cgd4_3630	0.68	0.1887	0.1082	0.573396926
CHUDEA4_3640	cgd4_3640	0.88	0.1604	0.0535	0.333541147
CHUDEA4_3650	cgd4_3650	0.83	0.0684	0.0928	1.356725146
CHUDEA4_3670	cgd4_3670	0.736	0.2431	0.1197	0.492389963
CHUDEA4_3680	cgd4_3680	0.878	0.113	0.0574	0.507964602
CHUDEA4_3690	cgd4_3690	0.717	0.1464	0.0635	0.433743169
CHUDEA4_3700	cgd4_3700	0.953	0.0364	0.0205	0.563186813
CHUDEA4_370	cgd4_370	0.978	0.0844	0.0102	0.120853081
CHUDEA4_3710	cgd4_3710	0.989	0.0671	0.0043	0.064083458
CHUDEA4_3720	cgd4_3720	0.985	0.0629	0.0061	0.096979332
CHUDEA4_3730	cgd4_3730	0.99	0.0223	0.0045	0.201793722
CHUDEA4_3740	cgd4_3740	0.976	0.0752	0.0104	0.138297872
CHUDEA4_3750	cgd4_3750	0.933	0.043	0.0301	0.7
CHUDEA4_3760	cgd4_3760	1	0.0959	0	0
CHUDEA4_3770	cgd4_3770	0.987	0.0753	0.0051	0.067729084
CHUDEA4_3780	cgd4_3780	0.975	0.0374	0.0107	0.286096257
CHUDEA4_3790	cgd4_3790	0.994	0.0615	0.0025	0.040650407
CHUDEA4_3800	cgd4_3800	0.951	0.0439	0.0211	0.480637813
CHUDEA4_380	cgd4_380	0.976	0.0525	0.0097	0.184761905
CHUDEA4_3810	cgd4_3810	0.974	0.0659	0.0103	0.15629742
CHUDEA4_3820	cgd4_3820	0.971	0.0668	0.0116	0.173652695
CHUDEA4_3830	cgd4_3830	0.976	0.0556	0.0108	0.194244604
CHUDEA4_3840	cgd4_3840	0.974	0.0591	0.0108	0.182741117
CHUDEA4_3850	cgd4_3850	0.998	0.0471	0.0008	0.016985138
CHUDEA4_3860	cgd4_3860	0.996	0.0582	0.0016	0.027491409
CHUDEA4_3870	cgd4_3870	0.967	0.0562	0.0134	0.238434164
CHUDEA4_3880	cgd4_3880	0.978	0.052	0.0059	0.113461538
CHUDEA4_3890	cgd4_3890	0.979	0.0745	0.0066	0.088590604
CHUDEA4_3900	cgd4_3900	0.988	0.0549	0.0047	0.0856102
CHUDEA4_390	cgd4_390	0.964	0.0534	0.0161	0.301498127
CHUDEA4_3910	cgd4_3910	0.961	0.1026	0.0151	0.147173489
CHUDEA4_3930	cgd4_3930	0.992	0.1718	0.0041	0.023864959
CHUDEA4_3940	cgd4_3940	0.914	0.137	0.0194	0.141605839
CHUDEA4_3950	cgd4_3950	0.98	0.055	0.008	0.145454545

CHUDEA4_3960	cgd4_3960	0.987	0.0453	0.0056	0.123620309
CHUDEA4_3970	cgd4_3970	0.85	0.2075	0.0529	0.254939759
CHUDEA4_3980	cgd4_3980	0.975	0.0537	0.0098	0.182495345
CHUDEA4_3990	cgd4_3990	0.983	0.0551	0.007	0.127041742
CHUDEA4_4000	cgd4_4000	0.974	0.0106	0.0112	1.056603774
CHUDEA4_400	cgd4_400	0.988	0.0639	0.005	0.078247261
CHUDEA4_4010	cgd4_4010	0.989	0.0463	0.0042	0.090712743
CHUDEA4_4020	cgd4_4020	0.996	0.0492	0.0016	0.032520325
CHUDEA4_4030	cgd4_4030	0.991	0.0753	0.0034	0.045152722
CHUDEA4_4040	cgd4_4040	0.983	0.0649	0.0078	0.1201849
CHUDEA4_4050	cgd4_4050	0.993	0.0507	0.0027	0.053254438
CHUDEA4_4060	cgd4_4060	0.989	0.077	0.0041	0.053246753
CHUDEA4_4080	cgd4_4080	1	0.0962	0	0
CHUDEA4_4090	cgd4_4090	0.967	0.1081	0.011	0.101757632
CHUDEA4_40	cgd4_40	0.969	0.0884	0.0133	0.150452489
CHUDEA4_4100	cgd4_4100	0.987	0.0999	0.0053	0.053053053
CHUDEA4_410	cgd4_410	0.99	0.0443	0.0045	0.101580135
CHUDEA4_4110	cgd4_4110	0.997	0.0898	0.0013	0.014476615
CHUDEA4_4120	cgd4_4120	0.983	0.0865	0.0068	0.078612717
CHUDEA4_4130	cgd4_4130	0.936	0.0615	0.0117	0.190243902
CHUDEA4_4140	cgd4_4140	0.966	0.0556	0.0125	0.224820144
CHUDEA4_4150	cgd4_4150	0.961	0.096	0.0095	0.098958333
CHUDEA4_4200	cgd4_4200	0.953	0.0508	0.0124	0.244094488
CHUDEA4_420	cgd4_420	0.989	0.052	0.0048	0.092307692
CHUDEA4_4210	cgd4_4210	0.969	0.0735	0.0129	0.175510204
CHUDEA4_4220	cgd4_4220	0.955	0.0575	0.0195	0.339130435
CHUDEA4_4230	cgd4_4230	0.978	0.0708	0.008	0.11299435
CHUDEA4_4240	cgd4_4240	0.963	0.0579	0.0161	0.27806563
CHUDEA4_4260	cgd4_4260	0.979	0.0506	0.0096	0.18972332
CHUDEA4_4270	cgd4_4270	0.94	0.0679	0.0276	0.406480118
CHUDEA4_4280	cgd4_4280	0.971	0.0662	0.0125	0.188821752
CHUDEA4_4290	cgd4_4290	0.97	0.0584	0.0144	0.246575342
CHUDEA4_4300	cgd4_4300	0.992	0.0769	0.0033	0.042912874
CHUDEA4_430	cgd4_430	0.98	0.0302	0.0079	0.261589404
CHUDEA4_4310	cgd4_4310	0.965	0.1006	0.0117	0.116302187
CHUDEA4_4320	cgd4_4320	0.984	0.0673	0.0062	0.092124814
CHUDEA4_4330	cgd4_4330	0.983	0.0426	0.007	0.164319249
CHUDEA4_4340	cgd4_4340	1	0.054	0	0
CHUDEA4_4350	cgd4_4350	0.979	0.0501	0.0076	0.151696607
CHUDEA4_4360	cgd4_4360	0.948	0.0739	0.0227	0.307171854
CHUDEA4_4370	cgd4_4370	0.984	0.0719	0.0068	0.0945758
CHUDEA4_4380	cgd4_4380	0.987	0.068	0.0057	0.083823529
CHUDEA4_4390	cgd4_4390	0.99	0.0763	0.0043	0.056356488
CHUDEA4_4400	cgd4_4400	0.997	0.0702	0.0014	0.01994302
CHUDEA4_440	cgd4_440	0.951	0.0854	0.0158	0.18501171

CHUDEA4_4410	cgd4_4410	0.964	0.0592	0.0157	0.265202703
CHUDEA4_4420	cgd4_4420	0.978	0.0807	0.0098	0.121437423
CHUDEA4_4440	cgd4_4440	0.958	0.0526	0.0194	0.368821293
CHUDEA4_4450	cgd4_4450	0.979	0.0845	0.0091	0.107692308
CHUDEA4_4460	cgd4_4460	0.979	0.0809	0.0092	0.113720643
CHUDEA4_4470	cgd4_4470	0.884	0.1219	0.0412	0.337981952
CHUDEA4_4480	cgd4_4480	0.89	0.1029	0.0523	0.508260447
CHUDEA4_4490	cgd4_4490	0.909	0.0853	0.0289	0.33880422
CHUDEA4_4500	cgd4_4500	0.769	0.1511	0.1151	0.761747187
CHUDEA4_450	cgd4_450	0.971	0.0516	0.0104	0.201550388
CHUDEA4_460	cgd4_460	0.986	0.0758	0.0049	0.064643799
CHUDEA4_470	cgd4_470	0.989	0.0336	0.0049	0.145833333
CHUDEA4_480	cgd4_480	0.97	0.0605	0.0148	0.244628099
CHUDEA4_490	cgd4_490	0.981	0.0709	0.0073	0.102961918
CHUDEA4_500	cgd4_500	0.988	0.0777	0.0048	0.061776062
CHUDEA4_50	cgd4_50	0.989	0.1552	0.0043	0.027706186
CHUDEA4_510	cgd4_510	0.952	0.0959	0.0154	0.160583942
CHUDEA4_520	cgd4_520	0.974	0.0455	0.0098	0.215384615
CHUDEA4_530	cgd4_530	0.987	0.0525	0.0056	0.106666667
CHUDEA4_540	cgd4_540	0.993	0.0502	0.0031	0.061752988
CHUDEA4_550	cgd4_550	0.97	0.0555	0.0132	0.237837838
CHUDEA4_560	cgd4_560	0.958	0.0639	0.0174	0.272300469
CHUDEA4_570	cgd4_570	1	0.0408	0	0
CHUDEA4_580	cgd4_580	0.977	0.0664	0.0106	0.159638554
CHUDEA4_590	cgd4_590	1	0.0549	0	0
CHUDEA4_600	cgd4_600	0.962	0.0499	0.0072	0.144288577
CHUDEA4_60	cgd4_60	0.981	0.0655	0.008	0.122137405
CHUDEA4_610	cgd4_610	0.969	0.0566	0.0131	0.231448763
CHUDEA4_620	cgd4_620	0.965	0.0584	0.0072	0.123287671
CHUDEA4_630	cgd4_630	0.99	0.1294	0.0042	0.032457496
CHUDEA4_640	cgd4_640	0.962	0.0827	0.0164	0.198307134
CHUDEA4_650	cgd4_650	0.976	0.0672	0.0106	0.157738095
CHUDEA4_660	cgd4_660	1	0.0867	0	0
CHUDEA4_670	cgd4_670	0.983	0.0694	0.0072	0.103746398
CHUDEA4_680	cgd4_680	0.982	0.0604	0.0075	0.124172185
CHUDEA4_690	cgd4_690	0.994	0.061	0.0028	0.045901639
CHUDEA4_700	cgd4_700	0.984	0.047	0.0069	0.146808511
CHUDEA4_70	cgd4_70	0.968	0.0549	0.014	0.255009107
CHUDEA4_710	cgd4_710	0.982	0.0897	0.0073	0.081382386
CHUDEA4_720	cgd4_720	0.979	0.071	0.0091	0.128169014
CHUDEA4_730	cgd4_730	0.95	0.0562	0.0216	0.384341637
CHUDEA4_740	cgd4_740	1	0.0342	0	0
CHUDEA4_750	cgd4_750	0.98	0.0565	0.008	0.14159292
CHUDEA4_760	cgd4_760	0.996	0.0633	0.0016	0.025276461
CHUDEA4_770	cgd4_770	0.874	0.297	0.0369	0.124242424

CHUDEA4_780	cgd4_780	0.973	0.0704	0.007	0.099431818
CHUDEA4_790	cgd4_790	1	0	0	0
CHUDEA4_800	cgd4_800	0.967	0.0705	0.0129	0.182978723
CHUDEA4_80	cgd4_80	0.976	0.0647	0.0111	0.171561051
CHUDEA4_810	cgd4_810	0.959	0.1128	0.0113	0.100177305
CHUDEA4_820	cgd4_820	0.986	0.0722	0.0052	0.072022161
CHUDEA4_830	cgd4_830	0.975	0.057	0.0102	0.178947368
CHUDEA4_840	cgd4_840	1	0.029	0	0
CHUDEA4_850	cgd4_850	0.971	0.0679	0.0125	0.184094256
CHUDEA4_860	cgd4_860	0.957	0.0532	0.0184	0.345864662
CHUDEA4_870	cgd4_870	0.977	0.0592	0.0097	0.163851351
CHUDEA4_880	cgd4_880	0.99	0.0778	0.0038	0.048843188
CHUDEA4_890	cgd4_890	0.976	0.0466	0.0098	0.210300429
CHUDEA4_900	cgd4_900	0.975	0.0851	0.0094	0.110458284
CHUDEA4_90	cgd4_90	0.974	0.0538	0.0122	0.226765799
CHUDEA4_910	cgd4_910	0.98	0.1115	0.0079	0.070852018
CHUDEA4_920	cgd4_920	0.747	0.6456	0.1387	0.21483891
CHUDEA4_930	cgd4_930	0.987	0.1251	0.0032	0.025579536
CHUDEA4_940	cgd4_940	0.983	0.0335	0.0072	0.214925373
CHUDEA4_950	cgd4_950	0.978	0.0667	0.0093	0.139430285
CHUDEA4_960	cgd4_960	0.996	0.077	0.0017	0.022077922
CHUDEA4_970	cgd4_970	0.986	0.0625	0.0059	0.0944
CHUDEA4_980	cgd4_980	0.98	0.0544	0.0089	0.163602941
CHUDEA4_990	cgd4_990	0.892	0.2127	0.0582	0.273624824
CHUDEA4_newUdeA_01	cgd4_3920	0.978	0.0525	0.0098	0.186666667
CHUDEA4_new_01	cgd4_new_01	1	0.0291	0	0
CHUDEA4_new_02	cgd4_new_02	1	0.0222	0	0
CHUDEA4_new_03	cgd4_new_03	0.958	0.0792	0.0165	0.208333333
CHUDEA4_new_04	cgd4_new_04	0.979	0.0421	0.0096	0.228028504
CHUDEA4_new_05	cgd4_new_05	1	0.0425	0	0
CHUDEA4_new_06	cgd4_new_06	1	0.0447	0	0
CHUDEA4_new_07	cgd4_new_07	0.972	0.0362	0.0094	0.259668508
CHUDEA5_1000	cgd5_1000	0.982	0.0623	0.0077	0.123595506
CHUDEA5_100	cgd5_100	0.973	0.0624	0.0119	0.190705128
CHUDEA5_1010	cgd5_1010	0.982	0.0735	0.008	0.108843537
CHUDEA5_1020	cgd5_1020	0.988	0.059	0.0052	0.088135593
CHUDEA5_1030	cgd5_1030	0.985	0.0365	0.0066	0.180821918
CHUDEA5_1040	cgd5_1040	0.977	0.059	0.01	0.169491525
CHUDEA5_1050	cgd5_1050	0.985	0.0564	0.0065	0.115248227
CHUDEA5_1060	cgd5_1060	0.986	0.0504	0.0064	0.126984127
CHUDEA5_1070	cgd5_1070	0.957	0.0711	0.0193	0.271448664
CHUDEA5_1080	cgd5_1080	0.984	0.0744	0.0069	0.092741935
CHUDEA5_1090	cgd5_1090	0.901	0.0651	0.0443	0.680491551
CHUDEA5_10	cgd5_10	0.87	0.0831	0.0557	0.670276775
CHUDEA5_1100	cgd5_1100	0.978	0.0782	0.0093	0.118925831

CHUDEA5_110	cgd5_110	0.986	0.0657	0.0058	0.088280061
CHUDEA5_1110	cgd5_1110	0.992	0.1059	0.0032	0.030217186
CHUDEA5_1120	cgd5_1120	0.976	0.0808	0.0109	0.13490099
CHUDEA5_1130	cgd5_1130	0.977	0.0613	0.0114	0.185970636
CHUDEA5_1140	cgd5_1140	0.975	0.0785	0.0107	0.136305732
CHUDEA5_1150	cgd5_1150	0.974	0.0595	0.0113	0.189915966
CHUDEA5_1160	cgd5_1160	0.977	0.0455	0.0112	0.246153846
CHUDEA5_1170	cgd5_1170	0.977	0.0755	0.0093	0.123178808
CHUDEA5_1180	cgd5_1180	0.967	0.0747	0.0141	0.18875502
CHUDEA5_1190	cgd5_1190	0.961	0.0302	0.0173	0.572847682
CHUDEA5_1200	cgd5_1200	0.988	0.0639	0.0053	0.082942097
CHUDEA5_120	cgd5_120	0.983	0.0875	0.0074	0.084571429
CHUDEA5_1230	cgd5_1230	0.932	0.0367	0.0333	0.907356948
CHUDEA5_1240	cgd5_1240	0.973	0.0841	0.0117	0.139120095
CHUDEA5_1250	cgd5_1250	0.962	0.0406	0.017	0.418719212
CHUDEA5_1260	cgd5_1260	0.96	0.0127	0.0186	1.464566929
CHUDEA5_1270	cgd5_1270	0.997	0.0604	0.0012	0.01986755
CHUDEA5_1280	cgd5_1280	0.98	0.0624	0.0088	0.141025641
CHUDEA5_1300	cgd5_1290	0.971	0.047	0.0128	0.272340426
CHUDEA5_130	cgd5_130	0.984	0.0673	0.0047	0.069836553
CHUDEA5_1310	cgd5_1310	0.978	0.0286	0.0095	0.332167832
CHUDEA5_1320	cgd5_1320	0.968	0.0565	0.016	0.283185841
CHUDEA5_1330	cgd5_1330	0.992	0.0797	0.0033	0.04140527
CHUDEA5_1340	cgd5_1340	0.952	0.0528	0.022	0.416666667
CHUDEA5_1360	cgd5_1360	0.966	0.0577	0.015	0.259965338
CHUDEA5_1370	cgd5_1370	0.944	0.0217	0.0262	1.207373272
CHUDEA5_1380	cgd5_1380	0.972	0.0735	0.0123	0.167346939
CHUDEA5_1390	cgd5_1390	0.952	0.0311	0.0235	0.75562701
CHUDEA5_1400	cgd5_1400	0.944	0.0552	0.0235	0.425724638
CHUDEA5_140	cgd5_140	0.99	0.0915	0.0045	0.049180328
CHUDEA5_1410	cgd5_1410	0.959	0.0792	0.0173	0.218434343
CHUDEA5_1420	cgd5_1420	0.993	0.0668	0.0035	0.05239521
CHUDEA5_1430	cgd5_1430	0.977	0.0968	0.0099	0.102272727
CHUDEA5_1440	cgd5_1440	0.97	0.0587	0.0128	0.218057922
CHUDEA5_1450	cgd5_1450	0.963	0.0523	0.0171	0.326959847
CHUDEA5_1460	cgd5_1460	0.971	0.0588	0.0122	0.207482993
CHUDEA5_1470	cgd5_1470	0.955	0.0481	0.0202	0.41995842
CHUDEA5_1480	cgd5_1480	0.971	0.0655	0.0128	0.195419847
CHUDEA5_1490	cgd5_1490	0.98	0.0592	0.0095	0.160472973
CHUDEA5_1500	cgd5_1500	0.97	0.0605	0.0147	0.242975207
CHUDEA5_150	cgd5_150	0.987	0.0706	0.0053	0.075070822
CHUDEA5_1510	cgd5_1510	0.949	0.0466	0.0205	0.439914163
CHUDEA5_1520	cgd5_1520	0.909	0.0468	0.0411	0.878205128
CHUDEA5_1530	cgd5_1530	0.971	0.1061	0.0125	0.117813384
CHUDEA5_1540	cgd5_1540	0.985	0.0564	0.0054	0.095744681

CHUDEA5_1550	cgd5_1550	1	0.0214	0	0
CHUDEA5_1560	cgd5_1560	0.947	0.0508	0.0257	0.505905512
CHUDEA5_1570	cgd5_1570	0.99	0.0459	0.0043	0.093681917
CHUDEA5_1580	cgd5_1580	0.995	0.0548	0.0024	0.04379562
CHUDEA5_1590	cgd5_1590	0.96	0.0469	0.0176	0.375266525
CHUDEA5_1600	cgd5_1600	0.994	0.0526	0.0026	0.049429658
CHUDEA5_160	cgd5_160	0.988	0.0912	0.0025	0.027412281
CHUDEA5_1610	cgd5_1610	0.972	0.0517	0.0127	0.245647969
CHUDEA5_1620	cgd5_1620	0.95	0.0758	0.0235	0.310026385
CHUDEA5_1630	cgd5_1630	0.974	0.0645	0.013	0.201550388
CHUDEA5_1640	cgd5_1640	0.997	0.0802	0.0014	0.017456359
CHUDEA5_1650	cgd5_1650	0.978	0.0745	0.0094	0.126174497
CHUDEA5_1660	cgd5_1660	0.902	0.0745	0.0434	0.582550336
CHUDEA5_1670	cgd5_1670	1	0.0655	0	0
CHUDEA5_1680	cgd5_1680	0.96	0.0396	0.0188	0.474747475
CHUDEA5_1690	cgd5_1690	0.982	0.06	0.0091	0.151666667
CHUDEA5_1700	cgd5_1700	0.953	0.0762	0.0198	0.25984252
CHUDEA5_170	cgd5_170	0.969	0.0864	0.0115	0.133101852
CHUDEA5_1710	cgd5_1710	0.994	0.0641	0.0025	0.03900156
CHUDEA5_1720	cgd5_1720	0.928	0.1111	0.0308	0.277227723
CHUDEA5_1730	cgd5_1730	0.965	0.1058	0.0157	0.148393195
CHUDEA5_1740	cgd5_1740	0.976	0.0467	0.0099	0.211991435
CHUDEA5_1750	cgd5_1750	0.99	0.1142	0.0041	0.035901926
CHUDEA5_1760	cgd5_1760	0.978	0.0766	0.0096	0.125326371
CHUDEA5_1770	cgd5_1770	0.995	0.0549	0.0024	0.043715847
CHUDEA5_1780	cgd5_1780	0.978	0.0567	0.0097	0.171075838
CHUDEA5_1790	cgd5_1790	0.964	0.0299	0.0167	0.558528428
CHUDEA5_1800	cgd5_1800	0.961	0.0713	0.018	0.252454418
CHUDEA5_180	cgd5_180	0.995	0.0843	0.0019	0.022538553
CHUDEA5_1810	cgd5_1810	0.966	0.044	0.015	0.340909091
CHUDEA5_1820	cgd5_1820	0.992	0.0496	0.0035	0.070564516
CHUDEA5_1830	cgd5_1830	0.977	0.049	0.0104	0.212244898
CHUDEA5_1840	cgd5_1840	0.97	0.0506	0.0146	0.288537549
CHUDEA5_1850	cgd5_1850	0.976	0.043	0.0104	0.241860465
CHUDEA5_1860	cgd5_1860	0.954	0.0505	0.019	0.376237624
CHUDEA5_1870	cgd5_1870	0.97	0.0841	0.0134	0.159334126
CHUDEA5_1880	cgd5_1880	0.994	0.0351	0.0025	0.071225071
CHUDEA5_1890	cgd5_1890	0.981	0.0824	0.0083	0.100728155
CHUDEA5_1900	cgd5_1900	0.978	0.0537	0.0101	0.188081937
CHUDEA5_190	cgd5_190	0.983	0.114	0.0052	0.045614035
CHUDEA5_1910	cgd5_1910	0.983	0.0648	0.0074	0.114197531
CHUDEA5_1920	cgd5_1920	1	0.0667	0	0
CHUDEA5_1930	cgd5_1930	0.981	0.032	0.0095	0.296875
CHUDEA5_1940	cgd5_1940	0.872	0.0649	0.0276	0.425269646
CHUDEA5_1950	cgd5_1950	0.979	0.0727	0.009	0.123796424

CHUDEA5_1960	cgd5_1960	0.998	0.0592	0.0011	0.018581081
CHUDEA5_1970	cgd5_1970	1	0.0956	0	0
CHUDEA5_1980	cgd5_1980	0.965	0.0597	0.0161	0.269681742
CHUDEA5_1990	cgd5_1990	0.954	0.0573	0.021	0.366492147
CHUDEA5_2000	cgd5_2000	0.975	0.073	0.0113	0.154794521
CHUDEA5_200	cgd5_200	0.982	0.0445	0.0069	0.15505618
CHUDEA5_2010	cgd5_2010	0.988	0.0852	0.0049	0.057511737
CHUDEA5_2020	cgd5_2020	0.988	0.0707	0.0052	0.073550212
CHUDEA5_2030	cgd5_2030	0.975	0.0469	0.0112	0.23880597
CHUDEA5_2040	cgd5_2040	0.988	0.0956	0.0052	0.054393305
CHUDEA5_2050	cgd5_2050	0.966	0.0515	0.0157	0.304854369
CHUDEA5_2060	cgd5_2060	0.968	0.0733	0.0144	0.196452933
CHUDEA5_2070	cgd5_2070	0.987	0.0634	0.0059	0.093059937
CHUDEA5_2080	cgd5_2080	0.973	0.0532	0.0118	0.221804511
CHUDEA5_2090	cgd5_2090	0.981	0.0516	0.0091	0.176356589
CHUDEA5_20	cgd5_20	0.889	0.1072	0.0554	0.516791045
CHUDEA5_2100	cgd5_2100	0.98	0.0554	0.0086	0.155234657
CHUDEA5_210	cgd5_210	0.939	0.0677	0.0122	0.180206795
CHUDEA5_2110	cgd5_2110	0.992	0.0564	0.0033	0.058510638
CHUDEA5_2120	cgd5_2120	0.966	0.0611	0.0063	0.103109656
CHUDEA5_2130	cgd5_2130	0.965	0.0708	0.0166	0.234463277
CHUDEA5_2140	cgd5_2140	0.974	0.0642	0.0112	0.174454829
CHUDEA5_2150	cgd5_2150	0.975	0.0757	0.0109	0.143989432
CHUDEA5_2160	cgd5_2160	0.973	0.0906	0.0122	0.134657837
CHUDEA5_2170	cgd5_2170	0.976	0.0586	0.0101	0.172354949
CHUDEA5_2180	cgd5_2180	0.679	0.2115	0.0363	0.171631206
CHUDEA5_2190	cgd5_2190	0.989	0.0556	0.0046	0.082733813
CHUDEA5_2200	cgd5_2200	0.973	0.0698	0.0118	0.169054441
CHUDEA5_220	cgd5_220	0.935	0.1014	0.0212	0.209072978
CHUDEA5_2210	cgd5_2210	1	0.0092	0	0
CHUDEA5_2220	cgd5_2220	0.968	0.1102	0.0123	0.111615245
CHUDEA5_2230	cgd5_2230	0.968	0.1285	0.0048	0.037354086
CHUDEA5_2240	cgd5_2240	0.991	0.0407	0.0041	0.100737101
CHUDEA5_2250	cgd5_2250	0.952	0.077	0.0183	0.237662338
CHUDEA5_2260	cgd5_2260	0.935	0.0271	0.0298	1.099630996
CHUDEA5_2270	cgd5_2270	0.982	0.0594	0.0079	0.132996633
CHUDEA5_2290	cgd5_2290	0.978	0.0682	0.0098	0.143695015
CHUDEA5_2300	cgd5_2300	0.977	0.0608	0.0097	0.159539474
CHUDEA5_230	cgd5_230	0.963	0.097	0.0091	0.093814433
CHUDEA5_2310	cgd5_2310	0.982	0.0594	0.008	0.134680135
CHUDEA5_2320	cgd5_2320	0.989	0.0807	0.0047	0.058240397
CHUDEA5_2330	cgd5_2330	0.997	0.0677	0.0011	0.016248154
CHUDEA5_2340	cgd5_2340	0.971	0.0556	0.0129	0.232014388
CHUDEA5_2350	cgd5_2350	0.986	0.058	0.0046	0.079310345
CHUDEA5_2360	cgd5_2360	0.966	0.0651	0.0148	0.22734255

CHUDEA5_2370	cgd5_2370	0.984	0.0274	0	0
CHUDEA5_2380	cgd5_2380	0.981	0.0581	0.0081	0.139414802
CHUDEA5_2390	cgd5_2390	0.978	0.0692	0.0101	0.145953757
CHUDEA5_2400	cgd5_2400	0.966	0.0531	0.0144	0.271186441
CHUDEA5_240	cgd5_240	0.944	0.0899	0.0239	0.265850945
CHUDEA5_2410	cgd5_2410	0.956	0.0762	0.0175	0.229658793
CHUDEA5_2420	cgd5_2420	0.94	0.0893	0.0173	0.193729003
CHUDEA5_2430	cgd5_2430	0.994	0.0496	0.0025	0.050403226
CHUDEA5_2440	cgd5_2440	0.972	0.0628	0.0121	0.192675159
CHUDEA5_2450	cgd5_2450	0.969	0.0459	0.014	0.305010893
CHUDEA5_2460	cgd5_2460	0.964	0.1075	0.0102	0.094883721
CHUDEA5_2470	cgd5_2470	0.99	0.0668	0.0042	0.062874251
CHUDEA5_2480	cgd5_2480	0.98	0.0544	0.0075	0.137867647
CHUDEA5_2490	cgd5_2490	0.981	0.0755	0.0076	0.100662252
CHUDEA5_2500	cgd5_2500	0.952	0.0712	0.0168	0.235955056
CHUDEA5_250	cgd5_250	0.969	0.0997	0.0092	0.09227683
CHUDEA5_2510	cgd5_2510	1	0.0148	0	0
CHUDEA5_2520	cgd5_2520	0.991	0.0471	0.0041	0.087048832
CHUDEA5_2530	cgd5_2530	0.99	0.0496	0.0047	0.094758065
CHUDEA5_2540	cgd5_2540	0.963	0.0774	0.0157	0.202842377
CHUDEA5_2550	cgd5_2550	1	0.0559	0	0
CHUDEA5_2560	cgd5_2560	0.968	0.0454	0.0151	0.332599119
CHUDEA5_2570	cgd5_2570	0.981	0.0528	0.0085	0.160984848
CHUDEA5_2580	cgd5_2580	0.961	0.0856	0.0178	0.207943925
CHUDEA5_2590	cgd5_2590	0.975	0.0592	0.0111	0.1875
CHUDEA5_2600	cgd5_2600	0.987	0.0791	0.0058	0.073324905
CHUDEA5_260	cgd5_260	0.958	0.0338	0.0104	0.307692308
CHUDEA5_2610	cgd5_2610	0.992	0.0373	0.0022	0.058981233
CHUDEA5_2620	cgd5_2620	0.986	0.0452	0.0069	0.152654867
CHUDEA5_2630	cgd5_2630	0.981	0.0873	0.007	0.080183276
CHUDEA5_2640	cgd5_2640	0.956	0.0452	0.016	0.353982301
CHUDEA5_2650	cgd5_2650	0.971	0.0799	0.0135	0.168961202
CHUDEA5_2660	cgd5_2660	0.982	0.0426	0.0077	0.180751174
CHUDEA5_2670	cgd5_2670	0.986	0.0538	0.0058	0.107806691
CHUDEA5_2680	cgd5_2680	0.977	0.0605	0.0098	0.161983471
CHUDEA5_2690	cgd5_2690	0.978	0.0643	0.0087	0.135303266
CHUDEA5_2700	cgd5_2700	0.943	0.0568	0.0299	0.526408451
CHUDEA5_2710	cgd5_2710	0.983	0.0463	0.0089	0.192224622
CHUDEA5_2720	cgd5_2720	0.969	0.0745	0.0122	0.163758389
CHUDEA5_2730	cgd5_2730	0.948	0.0531	0.0205	0.38606403
CHUDEA5_2740	cgd5_2740	0.982	0.0535	0.0071	0.13271028
CHUDEA5_2750	cgd5_2750	0.981	0.0596	0.0084	0.140939597
CHUDEA5_2760	cgd5_2760	0.97	0.0434	0.0145	0.334101382
CHUDEA5_2770	cgd5_2770	0.989	0.0492	0.0049	0.099593496
CHUDEA5_2780	cgd5_2780	1	0.0232	0	0

CHUDEA5_2790	cgd5_2790	0.989	0.0514	0.0047	0.091439689
CHUDEA5_2800	cgd5_2800	0.993	0.0454	0.0035	0.077092511
CHUDEA5_2810	cgd5_2810	0.98	0.0495	0.0096	0.193939394
CHUDEA5_2820	cgd5_2820	0.962	0.0274	0.0148	0.540145985
CHUDEA5_2830	cgd5_2840	0.974	0.0657	0.0081	0.123287671
CHUDEA5_2850	cgd5_2850	0.907	0.1046	0.0228	0.217973231
CHUDEA5_2860	cgd5_2860	0.917	0.057	0.0115	0.201754386
CHUDEA5_2870	cgd5_2870	0.972	0.0815	0.0114	0.139877301
CHUDEA5_2880	cgd5_2880	0.967	0.078	0.0098	0.125641026
CHUDEA5_2890	cgd5_2890	0.942	0.0625	0.0266	0.4256
CHUDEA5_2900	cgd5_2900	0.993	0.0558	0.0028	0.050179211
CHUDEA5_2910	cgd5_2910	0.986	0.0839	0.0057	0.067938021
CHUDEA5_2920	cgd5_2920	0.976	0.0434	0.0107	0.246543779
CHUDEA5_2930	cgd5_2930	0.977	0.0557	0.0101	0.181328546
CHUDEA5_2940	cgd5_2940	0.97	0.0342	0.0111	0.324561404
CHUDEA5_2950	cgd5_2950	0.966	0.0505	0.0137	0.271287129
CHUDEA5_2960	cgd5_2960	0.98	0.0621	0.0088	0.141706924
CHUDEA5_2990	cgd5_2990	0.982	0.0484	0.0077	0.159090909
CHUDEA5_3000	cgd5_3000	0.82	0.3177	0.1028	0.3235757
CHUDEA5_300	cgd5_300	0.975	0.058	0.0101	0.174137931
CHUDEA5_3010	cgd5_3010	0.975	0.0863	0.0093	0.107763615
CHUDEA5_3020	cgd5_3020	0.962	0.0684	0.0174	0.254385965
CHUDEA5_3030	cgd5_3030	0.938	0.0479	0.0208	0.434237996
CHUDEA5_3040	cgd5_3040	1	0.0347	0	0
CHUDEA5_3050	cgd5_3050	0.976	0.0951	0.01	0.105152471
CHUDEA5_3060	cgd5_3060	0.968	0.1201	0.0115	0.095753539
CHUDEA5_3070	cgd5_3070	0.991	0.0658	0.0035	0.053191489
CHUDEA5_3080	cgd5_3080	0.959	0.0725	0.0132	0.182068966
CHUDEA5_3090	cgd5_3090	0.981	0.0781	0.0081	0.103713188
CHUDEA5_30	cgd5_40	0.723	0.1175	0.1006	0.856170213
CHUDEA5_3100	cgd5_3100	0.98	0.0583	0.0069	0.118353345
CHUDEA5_310	cgd5_310	0.979	0.0972	0.0066	0.067901235
CHUDEA5_3120	cgd5_3120	0.975	0.0859	0.0086	0.100116414
CHUDEA5_3130	cgd5_3130	0.979	0.068	0.008	0.117647059
CHUDEA5_3140	cgd5_3140	0.983	0.0459	0.008	0.174291939
CHUDEA5_3160	cgd5_3160	0.995	0.0509	0.0023	0.04518664
CHUDEA5_3170	cgd5_3170	1	0.0194	0	0
CHUDEA5_3180	cgd5_3180	0.997	0.0593	0.0012	0.020236088
CHUDEA5_3190	cgd5_3190	0.983	0.0464	0.0074	0.159482759
CHUDEA5_3200	cgd5_3200	0.972	0.0606	0.0121	0.199669967
CHUDEA5_320	cgd5_320	0.96	0.0817	0.0165	0.201958384
CHUDEA5_3210	cgd5_3210	0.966	0.0749	0.0143	0.190921228
CHUDEA5_3220	cgd5_3220	0.994	0.0564	0.0027	0.04787234
CHUDEA5_3230	cgd5_3230	0.991	0.0975	0.0058	0.059487179
CHUDEA5_3240	cgd5_3240	0.957	0.1401	0.0157	0.112062812

CHUDEA5_3250	cgd5_3250	0.975	0.0996	0.0131	0.131526104
CHUDEA5_3260	cgd5_3260	0.982	0.1001	0.0075	0.074925075
CHUDEA5_3270	cgd5_3270	0.985	0.1165	0.0062	0.053218884
CHUDEA5_3280	cgd5_3280	0.963	0.0484	0.0157	0.324380165
CHUDEA5_3290	cgd5_3290	0.996	0.0763	0.0019	0.024901704
CHUDEA5_3300	cgd5_3300	0.964	0.0705	0.013	0.184397163
CHUDEA5_330	cgd5_330	0.973	0.0451	0.0115	0.254988914
CHUDEA5_3310	cgd5_3310	0.954	0.1056	0.0193	0.182765152
CHUDEA5_3320	cgd5_3320	0.991	0.0423	0.004	0.094562648
CHUDEA5_3330	cgd5_3330	1	0.0423	0	0
CHUDEA5_3340	cgd5_3340	0.997	0.0346	0.0011	0.031791908
CHUDEA5_3350	cgd5_3350	0.985	0.0904	0.0064	0.07079646
CHUDEA5_3360	cgd5_3360	0.995	0.123	0.0018	0.014634146
CHUDEA5_3370	cgd5_3370	0.964	0.0713	0.0175	0.245441795
CHUDEA5_3380	cgd5_3380	0.909	0.0748	0.0355	0.47459893
CHUDEA5_3390	cgd5_3390	0.971	0.0564	0.0128	0.226950355
CHUDEA5_3400	cgd5_3400	0.987	0.0243	0.0053	0.218106996
CHUDEA5_3410	cgd5_3410	0.974	0.076	0.0123	0.161842105
CHUDEA5_3420	cgd5_3420	0.979	0.0859	0.0089	0.103608847
CHUDEA5_3430	cgd5_3430	0.991	0.0769	0.0039	0.050715215
CHUDEA5_3440	cgd5_3440	0.947	0.0381	0.0261	0.68503937
CHUDEA5_3450	cgd5_3450	0.985	0.0545	0.0047	0.086238532
CHUDEA5_3460	cgd5_3460	0.993	0.0768	0.0035	0.045572917
CHUDEA5_3470	cgd5_3470	0.975	0.0406	0.0112	0.275862069
CHUDEA5_3480	cgd5_3480	0.981	0.1038	0.0083	0.079961464
CHUDEA5_3490	cgd5_3490	0.899	0.1066	0.0487	0.45684803
CHUDEA5_3500	cgd5_3500	0.985	0.0801	0.0056	0.069912609
CHUDEA5_3510	cgd5_3510	0.996	0.0752	0.0019	0.025265957
CHUDEA5_3520	cgd5_3520	0.991	0.0507	0.0039	0.076923077
CHUDEA5_3530	cgd5_3530	0.994	0.0628	0.0024	0.038216561
CHUDEA5_3540	cgd5_3540	0.986	0.0445	0.0068	0.152808989
CHUDEA5_3550	cgd5_3550	0.943	0.0963	0.0259	0.268951194
CHUDEA5_3560	cgd5_3560	0.985	0.0795	0.0072	0.090566038
CHUDEA5_3570	cgd5_3570	0.983	0.0889	0.0079	0.088863892
CHUDEA5_3580	cgd5_3580	0.988	0.1139	0.0051	0.044776119
CHUDEA5_3590	cgd5_3590	0.976	0.0655	0.0112	0.170992366
CHUDEA5_3600	cgd5_3600	0.95	0.0775	0.0229	0.295483871
CHUDEA5_360	cgd5_360	0.965	0.1398	0.0124	0.08869814
CHUDEA5_3610	cgd5_3610	0.975	0.1367	0.01	0.07315289
CHUDEA5_3620	cgd5_3620	0.975	0.0715	0.011	0.153846154
CHUDEA5_3630	cgd5_3630	0.982	0.063	0.0051	0.080952381
CHUDEA5_3640	cgd5_3640	0.981	0.0376	0.0109	0.289893617
CHUDEA5_3650	cgd5_3650	0.984	0.0615	0.0057	0.092682927
CHUDEA5_3660	cgd5_3660	0.956	0.0223	0.0178	0.798206278
CHUDEA5_3670	cgd5_3670	0.985	0.0657	0.0063	0.095890411

CHUDEA5_3680	cgd5_3680	0.977	0.0711	0.0106	0.149085795
CHUDEA5_3690	cgd5_3690	0.985	0.0596	0.0068	0.11409396
CHUDEA5_3700	cgd5_3700	0.994	0.0503	0.0025	0.049701789
CHUDEA5_370	cgd5_370	0.977	0.0761	0.0073	0.095926413
CHUDEA5_3710	cgd5_3710	0.963	0.0593	0.0072	0.121416526
CHUDEA5_3720	cgd5_3720	1	0.0117	0	0
CHUDEA5_3730	cgd5_3730	0.979	0.0407	0.0078	0.191646192
CHUDEA5_3740	cgd5_3740	1	0.0663	0	0
CHUDEA5_3750	cgd5_3750	0.943	0.0786	0.0145	0.184478372
CHUDEA5_3760	cgd5_3760	0.993	0.0912	0.0029	0.031798246
CHUDEA5_3770	cgd5_3770	0.976	0.106	0.0098	0.09245283
CHUDEA5_3780	cgd5_3780	0.965	0.0615	0.0153	0.248780488
CHUDEA5_3790	cgd5_3790	0.995	0.0682	0.002	0.029325513
CHUDEA5_3800	cgd5_3800	0.959	0.0744	0.0183	0.245967742
CHUDEA5_380	cgd5_380	0.952	0.0658	0.0095	0.1443769
CHUDEA5_3810	cgd5_3810	0.986	0.0427	0.0056	0.131147541
CHUDEA5_3820	cgd5_3820	0.98	0.0458	0.006	0.131004367
CHUDEA5_3830	cgd5_3830	0.907	0.0526	0.0254	0.482889734
CHUDEA5_3840	cgd5_3840	0.984	0.0864	0.0064	0.074074074
CHUDEA5_3850	cgd5_3850	0.977	0.069	0.0096	0.139130435
CHUDEA5_3860	cgd5_3860	0.992	0.2153	0.0031	0.014398514
CHUDEA5_3870	cgd5_3870	0.981	0.0679	0.0085	0.125184094
CHUDEA5_3880	cgd5_3880	0.957	0.0343	0.0185	0.539358601
CHUDEA5_3890	cgd5_3890	0.966	0.1114	0.011	0.098743268
CHUDEA5_3900	cgd5_3900	0.976	0.0584	0.01	0.171232877
CHUDEA5_390	cgd5_390	0.992	0.0868	0.0028	0.032258065
CHUDEA5_3910	cgd5_3910	0.977	0.0615	0.0096	0.156097561
CHUDEA5_3920	cgd5_3920	0.991	0.0178	0.0037	0.207865169
CHUDEA5_3930	cgd5_3930	0.983	0.0949	0.0072	0.075869336
CHUDEA5_3940	cgd5_3940	0.971	0.076	0.0139	0.182894737
CHUDEA5_3950	cgd5_3950	0.982	0.0769	0.008	0.104031209
CHUDEA5_3960	cgd5_3960	0.948	0.0748	0.0226	0.302139037
CHUDEA5_3980	cgd5_3980	0.969	0.0873	0.0128	0.146620848
CHUDEA5_3990	cgd5_3990	0.971	0.0476	0.0115	0.241596639
CHUDEA5_4000	cgd5_4000	0.967	0.057	0.013	0.228070175
CHUDEA5_400	cgd5_400	0.969	0.0926	0.0131	0.141468683
CHUDEA5_4010	cgd5_4010	0.976	0.0706	0.0113	0.160056657
CHUDEA5_4020	cgd5_4020	0.951	0.0309	0.022	0.71197411
CHUDEA5_4030	cgd5_4030	0.961	0.0397	0.0179	0.450881612
CHUDEA5_4040	cgd5_4040	0.975	0.063	0.01	0.158730159
CHUDEA5_4050	cgd5_4050	0.962	0.063	0.0157	0.249206349
CHUDEA5_4060	cgd5_4060	0.97	0.1412	0.0098	0.069405099
CHUDEA5_4070	cgd5_4070	0.994	0.0524	0.0023	0.04389313
CHUDEA5_4080	cgd5_4080	0.993	0.0768	0.0026	0.033854167
CHUDEA5_4090	cgd5_4090	0.953	0.0728	0.0121	0.166208791

CHUDEA5_40	cgd5_40	0.723	0.1175	0.1006	0.856170213
CHUDEA5_4100	cgd5_4100	0.989	0.0868	0.0042	0.048387097
CHUDEA5_410	cgd5_410	0.997	0.0826	0.0012	0.014527845
CHUDEA5_4110	cgd5_4110	1	0.0373	0	0
CHUDEA5_4120	cgd5_4120	0.944	0.0933	0.028	0.300107181
CHUDEA5_4130	cgd5_4130	0.984	0.1088	0.0063	0.057904412
CHUDEA5_4140	cgd5_4140	0.985	0.0778	0.0045	0.057840617
CHUDEA5_4150	cgd5_4150	0.986	0.0555	0.0059	0.106306306
CHUDEA5_4160	cgd5_4160	0.978	0.0591	0.0078	0.131979695
CHUDEA5_4170	cgd5_4170	0.993	0.0848	0.0027	0.031839623
CHUDEA5_4180	cgd5_4180	0.998	0.0639	0.0009	0.014084507
CHUDEA5_4190	cgd5_4190	0.961	0.0568	0.0144	0.253521127
CHUDEA5_4200	cgd5_4200	1	0.0653	0	0
CHUDEA5_420	cgd5_420	0.959	0.0933	0.0126	0.135048232
CHUDEA5_4210	cgd5_4210	0.993	0.0833	0.0027	0.032412965
CHUDEA5_4220	cgd5_4220	0.946	0.1043	0.0081	0.077660594
CHUDEA5_4230	cgd5_4230	0.962	0.0619	0.0125	0.201938611
CHUDEA5_4240	cgd5_4240	0.931	0.0846	0.0259	0.306146572
CHUDEA5_4250	cgd5_4250	0.971	0.0607	0.0131	0.215815486
CHUDEA5_4260	cgd5_4260	0.992	0.0727	0.0033	0.045392022
CHUDEA5_4270	cgd5_4270	0.96	0.0412	0.0189	0.458737864
CHUDEA5_4280	cgd5_4280	0.956	0.0596	0.0177	0.296979866
CHUDEA5_4290	cgd5_4290	0.974	0.0622	0.0114	0.183279743
CHUDEA5_4300	cgd5_4300	0.961	0.0611	0.0152	0.248772504
CHUDEA5_4310	cgd5_4310	0.977	0.0344	0.0096	0.279069767
CHUDEA5_4320	cgd5_4320	0.977	0.1165	0.0092	0.078969957
CHUDEA5_4330	cgd5_4330	0.996	0.0448	0.0018	0.040178571
CHUDEA5_4340	cgd5_4340	0.983	0.0821	0.0073	0.088915956
CHUDEA5_4350	cgd5_4350	0.985	0.0744	0.0062	0.083333333
CHUDEA5_4360	cgd5_4360	0.972	0.0755	0.0121	0.160264901
CHUDEA5_4370	cgd5_4370	0.977	0.0472	0.0106	0.224576271
CHUDEA5_4380	cgd5_4380	0.975	0.0478	0.0107	0.223849372
CHUDEA5_4390	cgd5_4390	0.984	0.0554	0.0061	0.110108303
CHUDEA5_4400	cgd5_4400	0.976	0.0667	0.01	0.149925037
CHUDEA5_440	cgd5_440	0.983	0.0482	0.007	0.145228216
CHUDEA5_4410	cgd5_4410	0.95	0.0547	0.018	0.329067642
CHUDEA5_4420	cgd5_4420	0.976	0.0908	0.0077	0.084801762
CHUDEA5_4430	cgd5_4430	0.976	0.0535	0.0083	0.155140187
CHUDEA5_4440	cgd5_4440	0.979	0.0725	0.0086	0.11862069
CHUDEA5_4460	cgd5_4460	0.959	0.0475	0.0103	0.216842105
CHUDEA5_4470	cgd5_4470	0.957	0.0746	0.0178	0.238605898
CHUDEA5_450	cgd5_450	0.89	0.0901	0.0112	0.124306326
CHUDEA5_460	cgd5_460	0.981	0.052	0.0076	0.146153846
CHUDEA5_480	cgd5_480	0.979	0.0591	0.0082	0.138747885
CHUDEA5_490	cgd5_490	0.992	0.0738	0.0034	0.046070461

CHUDEA5_500	cgd5_500	0.98	0.0576	0.0083	0.144097222
CHUDEA5_50	cgd5_50	0.82	0.0921	0.0953	1.034744843
CHUDEA5_510	cgd5_510	0.962	0.0499	0.015	0.300601202
CHUDEA5_520	cgd5_520	0.983	0.0501	0.0072	0.143712575
CHUDEA5_530	cgd5_530	0.988	0.0885	0.0052	0.058757062
CHUDEA5_540	cgd5_540	0.913	0.107	0.0138	0.128971963
CHUDEA5_550	cgd5_550	0.959	0.0677	0.0091	0.134416544
CHUDEA5_560	cgd5_560	0.98	0.0772	0.0075	0.097150259
CHUDEA5_570	cgd5_580	0.975	0.0776	0.0107	0.137886598
CHUDEA5_590	cgd5_590	0.952	0.049	0.023	0.469387755
CHUDEA5_600	cgd5_600	0.978	0.0432	0.0051	0.118055556
CHUDEA5_60	cgd5_60	1	0.0429	0	0
CHUDEA5_610	cgd5_610	0.996	0.0594	0.0014	0.023569024
CHUDEA5_620	cgd5_620	0.983	0.0274	0.0071	0.259124088
CHUDEA5_630	cgd5_630	0.993	0.0649	0.003	0.046224961
CHUDEA5_640	cgd5_640	0.987	0.0701	0.0054	0.07703281
CHUDEA5_650	cgd5_650	0.972	0.0525	0.0129	0.245714286
CHUDEA5_660	cgd5_660	0.975	0.0408	0.0119	0.291666667
CHUDEA5_670	cgd5_670	0.992	0.0715	0.0043	0.06013986
CHUDEA5_680	cgd5_680	1	0.0762	0	0
CHUDEA5_690	cgd5_690	0.981	0.0691	0.0095	0.13748191
CHUDEA5_700	cgd5_700	1	0.0447	0	0
CHUDEA5_70	cgd5_70	0.989	0.0684	0.005	0.073099415
CHUDEA5_710	cgd5_710	0.988	0.0397	0.0055	0.138539043
CHUDEA5_720	cgd5_720	0.98	0.0735	0.008	0.108843537
CHUDEA5_730	cgd5_730	0.978	0.0529	0.0093	0.175803403
CHUDEA5_740	cgd5_740	0.985	0.0735	0.0063	0.085714286
CHUDEA5_750	cgd5_750	0.986	0.0627	0.0064	0.102073365
CHUDEA5_760	cgd5_760	0.984	0.0403	0.0066	0.163771712
CHUDEA5_770	cgd5_770	0.972	0.0393	0.012	0.305343511
CHUDEA5_780	cgd5_780	0.984	0.0585	0.0068	0.116239316
CHUDEA5_790	cgd5_790	0.953	0.0496	0.0213	0.429435484
CHUDEA5_800	cgd5_800	0.987	0.0699	0.0054	0.077253219
CHUDEA5_80	cgd5_80	0.937	0.0324	0.0283	0.87345679
CHUDEA5_810	cgd5_810	0.986	0.0611	0.006	0.098199673
CHUDEA5_820	cgd5_820	0.998	0.0405	0.0009	0.022222222
CHUDEA5_830	cgd5_830	0.962	0.0595	0.0175	0.294117647
CHUDEA5_840	cgd5_840	0.986	0.0712	0.0062	0.087078652
CHUDEA5_850	cgd5_850	0.984	0.0694	0.0071	0.102305476
CHUDEA5_860	cgd5_860	0.99	0.0692	0.0042	0.060693642
CHUDEA5_870	cgd5_870	0.958	0.085	0.0172	0.202352941
CHUDEA5_880	cgd5_880	0.981	0.0878	0.008	0.091116173
CHUDEA5_890	cgd5_890	0.981	0.0546	0.0077	0.141025641
CHUDEA5_900	cgd5_900	0.977	0.0573	0.0101	0.176265271
CHUDEA5_90	cgd5_90	0.968	0.0895	0.0158	0.176536313

CHUDEA5_910	cgd5_910	0.975	0.0785	0.0105	0.133757962
CHUDEA5_920	cgd5_920	0.972	0.0604	0.0124	0.205298013
CHUDEA5_930	cgd5_930	0.983	0.0603	0.0069	0.114427861
CHUDEA5_940	cgd5_940	1	0.0196	0	0
CHUDEA5_950	cgd5_950	0.993	0.0586	0.0028	0.04778157
CHUDEA5_960	cgd5_960	0.992	0.0469	0.0036	0.076759062
CHUDEA5_970	cgd5_970	0.995	0.0626	0.0023	0.036741214
CHUDEA5_980	cgd5_980	0.929	0.0818	0.0327	0.399755501
CHUDEA5_990	cgd5_990	0.963	0.0596	0.0157	0.263422819
CHUDEA5_newUdeA_01	cgd6_5470	0.78	0.1606	0.1035	0.644458281
CHUDEA5_newUdeA_02	cgd6_5460	0.907	0.0738	0.0479	0.649051491
CHUDEA5_new_01	cgd5_new_01	0.833	0.1032	0.0495	0.479651163
CHUDEA5_new_02	cgd5_new_02	0.973	0.0578	0.0093	0.160899654
CHUDEA5_new_03	cgd5_new_03	0.967	0.1719	0.0141	0.082024433
CHUDEA5_new_04	cgd5_new_04	0.949	0.0837	0.0218	0.260454002
CHUDEA5_new_05	cgd5_new_05	0.971	0.0266	0.0129	0.484962406
CHUDEA5_new_06	cgd5_new_06	0.995	0.0575	0.0021	0.036521739
CHUDEA6_1000	cgd6_1000	0.907	0.1134	0.0241	0.212522046
CHUDEA6_100	cgd6_100	0.984	0.0642	0.0065	0.101246106
CHUDEA6_1010	cgd6_1010	0.978	0.0708	0.0096	0.13559322
CHUDEA6_1020	cgd6_1020	0.996	0.0919	0.0017	0.018498368
CHUDEA6_1030	cgd6_1030	0.933	0.0431	0.0313	0.726218097
CHUDEA6_1040	cgd6_1040	0.964	0.0929	0.0157	0.168998924
CHUDEA6_1050	cgd6_1050	1	0.0627	0	0
CHUDEA6_1060	cgd6_1060	0.961	0.0661	0.019	0.287443268
CHUDEA6_1070	cgd6_1070	0.961	0.0772	0.0185	0.239637306
CHUDEA6_1080	cgd6_1080	0.669	0.3458	0.201	0.581260844
CHUDEA6_1090	cgd6_1090	0.984	0.0497	0.0078	0.15694165
CHUDEA6_10	cgd6_10	0.471	0.5254	0.3345	0.636657785
CHUDEA6_1100	cgd6_1100	0.954	0.0794	0.017	0.214105793
CHUDEA6_110	cgd6_110	0.99	0.1525	0.0042	0.027540984
CHUDEA6_1110	cgd6_1110	0.969	0.0459	0.0126	0.274509804
CHUDEA6_1120	cgd6_1120	0.952	0.0765	0.0216	0.282352941
CHUDEA6_1130	cgd6_1130	0.944	0.0357	0.0248	0.694677871
CHUDEA6_1140	cgd6_1140	0.98	0.0372	0.0094	0.252688172
CHUDEA6_1150	cgd6_1150	0.959	0.0255	0.0195	0.764705882
CHUDEA6_1160	cgd6_1160	0.979	0.0545	0.0104	0.190825688
CHUDEA6_1170	cgd6_1170	0.904	0.0498	0.0348	0.698795181
CHUDEA6_1180	cgd6_1180	0.966	0.0625	0.0158	0.2528
CHUDEA6_1190	cgd6_1190	0.992	0.0954	0.0053	0.055555556
CHUDEA6_1200	cgd6_1200	0.993	0.0529	0.0032	0.060491493
CHUDEA6_120	cgd6_120	0.994	0.0699	0.0029	0.04148784
CHUDEA6_1210	cgd6_1210	0.991	0.0702	0.0035	0.04985755
CHUDEA6_1220	cgd6_1220	0.977	0.1055	0.0104	0.098578199
CHUDEA6_1230	cgd6_1230	0.96	0.0541	0.0187	0.345656192

CHUDEA6_1240	cgd6_1240	0.987	0.0634	0.0055	0.086750789
CHUDEA6_1250	cgd6_1260	0.974	0.0698	0.0107	0.153295129
CHUDEA6_1270	cgd6_1270	0.993	0.0795	0.0029	0.036477987
CHUDEA6_1280	cgd6_1280	0.968	0.0464	0.0145	0.3125
CHUDEA6_1290	cgd6_1290	0.981	0.0337	0.0064	0.189910979
CHUDEA6_1300	cgd6_1300	0.986	0.0758	0.0062	0.081794195
CHUDEA6_130	cgd6_130	0.994	0.0578	0.0025	0.043252595
CHUDEA6_1310	cgd6_1310	0.981	0.0541	0.0064	0.118299445
CHUDEA6_1320	cgd6_1320	0.984	0.0333	0.0067	0.201201201
CHUDEA6_1330	cgd6_1330	0.979	0.0402	0.009	0.223880597
CHUDEA6_1340	cgd6_1340	0.994	0.0577	0.0025	0.043327556
CHUDEA6_1350	cgd6_1350	0.967	0.0676	0.0162	0.23964497
CHUDEA6_1360	cgd6_1360	0.95	0.0276	0.0224	0.811594203
CHUDEA6_1370	cgd6_1370	0.985	0.0406	0.0062	0.15270936
CHUDEA6_1380	cgd6_1380	1	0.0651	0	0
CHUDEA6_1390	cgd6_1390	1	0.0191	0	0
CHUDEA6_1400	cgd6_1400	0.98	0.0251	0.0126	0.501992032
CHUDEA6_140	cgd6_140	0.991	0.0649	0.0037	0.057010786
CHUDEA6_1410	cgd6_1410	0.986	0.0672	0.0062	0.092261905
CHUDEA6_1420	cgd6_1420	0.941	0.0712	0.0138	0.193820225
CHUDEA6_1430	cgd6_1430	0.989	0.0694	0.0033	0.047550432
CHUDEA6_1440	cgd6_1440	0.953	0.0495	0.0198	0.4
CHUDEA6_1450	cgd6_1450	0.984	0.0402	0.0071	0.176616915
CHUDEA6_1460	cgd6_1460	0.997	0.0806	0.0013	0.016129032
CHUDEA6_1470	cgd6_1470	0.969	0.0473	0.0134	0.283298097
CHUDEA6_1480	cgd6_1480	0.978	0.0608	0.009	0.148026316
CHUDEA6_1490	cgd6_1490	0.99	0.0195	0.0047	0.241025641
CHUDEA6_1500	cgd6_1500	0.997	0.0607	0.0016	0.026359143
CHUDEA6_150	cgd6_150	0.976	0.0553	0.0105	0.189873418
CHUDEA6_1510	cgd6_1510	0.991	0.0519	0.0037	0.071290944
CHUDEA6_1520	cgd6_1520	0.98	0.0459	0.0093	0.202614379
CHUDEA6_1530	cgd6_1530	0.963	0.0617	0.0178	0.288492707
CHUDEA6_1540	cgd6_1540	0.976	0.0523	0.0104	0.198852772
CHUDEA6_1550	cgd6_1550	0.984	0.0937	0.0063	0.067235859
CHUDEA6_1560	cgd6_1560	0.959	0.0713	0.0192	0.269284712
CHUDEA6_1570	cgd6_1570	0.977	0.0274	0.0112	0.408759124
CHUDEA6_1580	cgd6_1580	0.979	0.0477	0.0098	0.205450734
CHUDEA6_1590	cgd6_1590	0.94	0.0535	0.0131	0.244859813
CHUDEA6_1600	cgd6_1600	0.912	0.0909	0.0415	0.456545655
CHUDEA6_160	cgd6_160	0.952	0.0732	0.0186	0.254098361
CHUDEA6_1610	cgd6_1610	0.982	0.0655	0.0079	0.120610687
CHUDEA6_1620	cgd6_1620	0.92	0.0725	0.0359	0.495172414
CHUDEA6_1630	cgd6_1630	0.933	0.0905	0.0024	0.026519337
CHUDEA6_1640	cgd6_1640	0.965	0.0666	0.0165	0.247747748
CHUDEA6_1650	cgd6_1650	0.974	0.0211	0.0118	0.559241706

CHUDEA6_1660	cgd6_1660	0.955	0.0659	0.0213	0.323216995
CHUDEA6_1670	cgd6_1670	0.986	0.0398	0.006	0.150753769
CHUDEA6_1680	cgd6_1680	0.939	0.0649	0.0267	0.411402157
CHUDEA6_1690	cgd6_1690	0.985	0.058	0.0064	0.110344828
CHUDEA6_1700	cgd6_1700	0.962	0.0633	0.016	0.252764613
CHUDEA6_170	cgd6_170	0.972	0.0716	0.008	0.111731844
CHUDEA6_1710	cgd6_1710	0.954	0.073	0.0205	0.280821918
CHUDEA6_1720	cgd6_1720	0.986	0.0574	0.0064	0.111498258
CHUDEA6_1730	cgd6_1730	0.994	0.091	0.0025	0.027472527
CHUDEA6_1740	cgd6_1740	0.98	0.0603	0.009	0.149253731
CHUDEA6_1750	cgd6_1750	0.973	0.0397	0.0125	0.314861461
CHUDEA6_1760	cgd6_1760	0.976	0.0571	0.0111	0.194395797
CHUDEA6_1770	cgd6_1770	0.969	0.0688	0.0136	0.197674419
CHUDEA6_1780	cgd6_1780	0.96	0.0548	0.0181	0.330291971
CHUDEA6_1790	cgd6_1790	0.973	0.0568	0.0118	0.207746479
CHUDEA6_1800	cgd6_1800	0.984	0.0478	0.0068	0.142259414
CHUDEA6_180	cgd6_180	0.972	0.0626	0.0096	0.153354633
CHUDEA6_1810	cgd6_1810	1	0.0393	0	0
CHUDEA6_1820	cgd6_1820	0.984	0.0641	0.0066	0.102964119
CHUDEA6_1830	cgd6_1830	0.953	0.0972	0.0207	0.212962963
CHUDEA6_1840	cgd6_1840	0.993	0.0486	0.0031	0.063786008
CHUDEA6_1850	cgd6_1850	0.985	0.1083	0.0064	0.059095106
CHUDEA6_1860	cgd6_1860	0.965	0.0621	0.0119	0.191626409
CHUDEA6_1870	cgd6_1870	0.965	0.0766	0.0146	0.190600522
CHUDEA6_1880	cgd6_1880	0.979	0.0418	0.0092	0.220095694
CHUDEA6_1890	cgd6_1890	0.949	0.0598	0.0217	0.362876254
CHUDEA6_1900	cgd6_1900	0.934	0.0933	0.0297	0.318327974
CHUDEA6_190	cgd6_190	0.972	0.0738	0.0063	0.085365854
CHUDEA6_1910	cgd6_1910	0.927	0.0648	0.0346	0.533950617
CHUDEA6_1920	cgd6_1920	0.952	0.0754	0.0173	0.229442971
CHUDEA6_1930	cgd6_1930	0.974	0.0379	0.0122	0.321899736
CHUDEA6_1940	cgd6_1940	0.941	0.0717	0.027	0.376569038
CHUDEA6_1950	cgd6_1950	0.993	0.0722	0.0033	0.045706371
CHUDEA6_1960	cgd6_1960	0.984	0.0472	0.0073	0.154661017
CHUDEA6_1970	cgd6_1970	0.963	0.0458	0.0173	0.377729258
CHUDEA6_1980	cgd6_1980	0.951	0.0377	0.0247	0.655172414
CHUDEA6_1990	cgd6_1990	0.972	0.0666	0.0116	0.174174174
CHUDEA6_2000	cgd6_2000	0.956	0.0473	0.0198	0.418604651
CHUDEA6_200	cgd6_200	0.991	0.0791	0.0035	0.044247788
CHUDEA6_2010	cgd6_2010	0.977	0.0556	0.0106	0.190647482
CHUDEA6_2020	cgd6_2020	0.986	0.0465	0.0063	0.135483871
CHUDEA6_2030	cgd6_2030	0.979	0.0963	0.0097	0.100726895
CHUDEA6_2040	cgd6_2040	0.992	0.0682	0.0036	0.052785924
CHUDEA6_2050	cgd6_2050	0.986	0.0428	0.0059	0.137850467
CHUDEA6_2060	cgd6_2060	0.982	0.0745	0.0078	0.104697987

CHUDEA6_2070	cgd6_2070	0.977	0.0538	0.0099	0.18401487
CHUDEA6_2080	cgd6_2080	0.969	0.0728	0.0134	0.184065934
CHUDEA6_2090	cgd6_2090	0.992	0.0591	0.0025	0.042301184
CHUDEA6_20	cgd6_20	0.98	0.0296	0.0081	0.273648649
CHUDEA6_2100	cgd6_2100	0.95	0.0691	0.0218	0.315484805
CHUDEA6_210	cgd6_210	0.948	0.0723	0.0214	0.295988935
CHUDEA6_2120	cgd6_2120	0.944	0.1104	0.0254	0.230072464
CHUDEA6_2130	cgd6_2130	0.966	0.0654	0.0161	0.24617737
CHUDEA6_2140	cgd6_2140	0.978	0.0603	0.0087	0.144278607
CHUDEA6_2150	cgd6_2150	0.931	0.0593	0.0306	0.516020236
CHUDEA6_2160	cgd6_2160	0.977	0.0693	0.0098	0.141414141
CHUDEA6_2170	cgd6_2170	0.988	0.0495	0.0052	0.105050505
CHUDEA6_2180	cgd6_2180	0.982	0.079	0.0091	0.115189873
CHUDEA6_2190	cgd6_2190	0.966	0.0525	0.0153	0.291428571
CHUDEA6_2200	cgd6_2200	0.971	0.0656	0.0136	0.207317073
CHUDEA6_220	cgd6_220	0.966	0.0494	0.0143	0.289473684
CHUDEA6_2210	cgd6_2210	0.993	0.0503	0.0032	0.06361829
CHUDEA6_2220	cgd6_2220	0.994	0.0624	0.0027	0.043269231
CHUDEA6_2230	cgd6_2230	0.978	0.0705	0.0097	0.137588652
CHUDEA6_2240	cgd6_2240	0.963	0.0593	0.0161	0.271500843
CHUDEA6_2250	cgd6_2250	0.98	0.0481	0.009	0.187110187
CHUDEA6_2260	cgd6_2260	0.977	0.0795	0.0099	0.124528302
CHUDEA6_2270	cgd6_2270	0.99	0.0678	0.0047	0.069321534
CHUDEA6_2280	cgd6_2280	0.963	0.0697	0.0173	0.2482066
CHUDEA6_2290	cgd6_2290	0.994	0.0499	0.0027	0.054108216
CHUDEA6_2300	cgd6_2300	0.981	0.0493	0.0087	0.176470588
CHUDEA6_230	cgd6_230	0.959	0.0573	0.0173	0.301919721
CHUDEA6_2310	cgd6_2310	0.986	0.0627	0.0063	0.100478469
CHUDEA6_2320	cgd6_2320	0.975	0.0577	0.0107	0.185441941
CHUDEA6_2330	cgd6_2330	1	0.034	0	0
CHUDEA6_2340	cgd6_2340	0.988	0.0378	0.0055	0.145502646
CHUDEA6_2350	cgd6_2350	0.936	0.0465	0.0294	0.632258065
CHUDEA6_2360	cgd6_2360	0.982	0.0203	0.0077	0.379310345
CHUDEA6_2370	cgd6_2370	0.991	0.047	0.0045	0.095744681
CHUDEA6_2380	cgd6_2380	0.989	0.041	0.0049	0.119512195
CHUDEA6_2390	cgd6_2390	0.978	0.0462	0.0093	0.201298701
CHUDEA6_2400	cgd6_2400	1	0.047	0	0
CHUDEA6_240	cgd6_240	0.958	0.0723	0.0059	0.081604426
CHUDEA6_2410	cgd6_2410	0.955	0.0658	0.0198	0.300911854
CHUDEA6_2420	cgd6_2420	0.967	0.0718	0.0138	0.192200557
CHUDEA6_2430	cgd6_2430	0.989	0.0706	0.0048	0.067988669
CHUDEA6_2440	cgd6_2440	0.996	0.0708	0.0017	0.024011299
CHUDEA6_2450	cgd6_2450	0.997	0.0458	0.0016	0.034934498
CHUDEA6_2460	cgd6_2460	0.994	0.0662	0.0029	0.043806647
CHUDEA6_2470	cgd6_2470	0.985	0.0742	0.0067	0.090296496

CHUDEA6_2480	cgd6_2480	0.985	0.0377	0.0068	0.180371353
CHUDEA6_2490	cgd6_2490	0.991	0.0592	0.0039	0.065878378
CHUDEA6_2500	cgd6_2500	0.979	0.0626	0.0098	0.156549521
CHUDEA6_250	cgd6_250	0.974	0.0439	0.0102	0.232346241
CHUDEA6_2510	cgd6_2510	0.988	0.0754	0.0054	0.071618037
CHUDEA6_2520	cgd6_2520	0.981	0.07	0.011	0.157142857
CHUDEA6_2530	cgd6_2530	0.968	0.0715	0.0141	0.197202797
CHUDEA6_2540	cgd6_2540	0.979	0.0504	0.009	0.178571429
CHUDEA6_2550	cgd6_2550	0.987	0.0271	0.0058	0.21402214
CHUDEA6_2560	cgd6_2560	0.949	0.0615	0.0232	0.377235772
CHUDEA6_2570	cgd6_2570	0.997	0.0351	0.0012	0.034188034
CHUDEA6_2580	cgd6_2580	0.959	0.0389	0.0188	0.483290488
CHUDEA6_2590	cgd6_2590	0.986	0.0526	0.0066	0.125475285
CHUDEA6_2600	cgd6_2600	0.967	0.0601	0.0142	0.236272879
CHUDEA6_2610	cgd6_2610	0.982	0.0458	0.0095	0.207423581
CHUDEA6_2620	cgd6_2620	0.984	0.0429	0.0076	0.177156177
CHUDEA6_2630	cgd6_2630	0.968	0.0553	0.0144	0.26039783
CHUDEA6_2640	cgd6_2640	0.97	0.0491	0.0134	0.272912424
CHUDEA6_2650	cgd6_2650	0.993	0.067	0.0031	0.046268657
CHUDEA6_2660	cgd6_2660	0.989	0.0425	0.0052	0.122352941
CHUDEA6_2670	cgd6_2670	0.982	0.0494	0.0082	0.165991903
CHUDEA6_2680	cgd6_2680	0.978	0.0499	0.0095	0.190380762
CHUDEA6_2690	cgd6_2690	0.988	0.1194	0.0055	0.046063652
CHUDEA6_2700	cgd6_2700	0.991	0.0359	0.0042	0.116991643
CHUDEA6_2710	cgd6_2710	0.984	0.0552	0.0073	0.132246377
CHUDEA6_2720	cgd6_2720	0.933	0.0661	0.0306	0.462934947
CHUDEA6_2730	cgd6_2730	0.981	0.0436	0.0087	0.199541284
CHUDEA6_2740	cgd6_2740	0.985	0.0336	0.0067	0.199404762
CHUDEA6_2750	cgd6_2750	0.96	0.0549	0.0195	0.355191257
CHUDEA6_2760	cgd6_2760	0.958	0.0629	0.0176	0.279809221
CHUDEA6_2770	cgd6_2770	0.99	0.1021	0.0045	0.044074437
CHUDEA6_2780	cgd6_2780	0.973	0.0558	0.0121	0.216845878
CHUDEA6_2790	cgd6_2790	0.996	0.0657	0.0017	0.02587519
CHUDEA6_2800	cgd6_2800	0.988	0.0562	0.0062	0.110320285
CHUDEA6_2810	cgd6_2810	0.972	0.038	0.0132	0.347368421
CHUDEA6_2820	cgd6_2820	0.977	0.0672	0.0101	0.150297619
CHUDEA6_2830	cgd6_2830	0.991	0.1096	0.0058	0.052919708
CHUDEA6_2840	cgd6_2840	0.988	0.0537	0.0054	0.100558659
CHUDEA6_2850	cgd6_2850	0.988	0.045	0.0049	0.108888889
CHUDEA6_2860	cgd6_2860	0.973	0.0445	0.0138	0.31011236
CHUDEA6_2870	cgd6_2870	0.98	0.0261	0.0091	0.348659004
CHUDEA6_2880	cgd6_2880	0.971	0.0612	0.0146	0.238562092
CHUDEA6_2890	cgd6_2890	0.983	0.0709	0.0072	0.101551481
CHUDEA6_2900	cgd6_2900	0.991	0.0757	0.0039	0.051519155
CHUDEA6_290	cgd6_290	0.991	0.0249	0.0049	0.196787149

CHUDEA6_2910	cgd6_2910	0.965	0.047	0.0158	0.336170213
CHUDEA6_2920	cgd6_2920	0.99	0.0518	0.0042	0.081081081
CHUDEA6_2930	cgd6_2930	0.976	0.0629	0.0109	0.173290938
CHUDEA6_2940	cgd6_2940	0.966	0.0636	0.0151	0.237421384
CHUDEA6_2950	cgd6_2950	0.966	0.0795	0.0149	0.187421384
CHUDEA6_2960	cgd6_2960	0.992	0.0553	0.0036	0.065099458
CHUDEA6_2970	cgd6_2970	0.992	0.0881	0.0032	0.036322361
CHUDEA6_2980	cgd6_2980	0.983	0.0422	0.0079	0.187203791
CHUDEA6_2990	cgd6_2990	0.996	0.0326	0.0017	0.052147239
CHUDEA6_3000	cgd6_3000	0.988	0.0563	0.0054	0.095914742
CHUDEA6_300	cgd6_300	0.964	0.0784	0.0083	0.105867347
CHUDEA6_3010	cgd6_3010	0.991	0.075	0.0042	0.056
CHUDEA6_3020	cgd6_3020	0.97	0.0622	0.0135	0.217041801
CHUDEA6_3030	cgd6_3030	0.976	0.0743	0.0099	0.133243607
CHUDEA6_3040	cgd6_3040	0.956	0.0441	0.0209	0.473922902
CHUDEA6_3050	cgd6_3050	0.854	0.0943	0.0684	0.725344645
CHUDEA6_3060	cgd6_3060	0.979	0.0117	0.009	0.769230769
CHUDEA6_3070	cgd6_3070	0.96	0.0643	0.0187	0.290824261
CHUDEA6_3080	cgd6_3080	0.969	0.0548	0.0127	0.231751825
CHUDEA6_3090	cgd6_3090	0.965	0.0564	0.0148	0.262411348
CHUDEA6_30	cgd6_30	0.971	0.0462	0.0131	0.283549784
CHUDEA6_3100	cgd6_3100	0.989	0.0776	0.0043	0.055412371
CHUDEA6_310	cgd6_310	0.981	0.0643	0.0101	0.157076205
CHUDEA6_3110	cgd6_3110	0.909	0.1677	0.0206	0.122838402
CHUDEA6_3120	cgd6_3120	0.956	0.0387	0.0135	0.348837209
CHUDEA6_3130	cgd6_3130	0.98	0.077	0.0075	0.097402597
CHUDEA6_3140	cgd6_3140	0.973	0.0583	0.0123	0.210977702
CHUDEA6_3150	cgd6_3150	0.984	0.0457	0.0083	0.181619256
CHUDEA6_3160	cgd6_3160	0.964	0.052	0.015	0.288461538
CHUDEA6_3170	cgd6_3170	0.975	0.0598	0.0114	0.190635452
CHUDEA6_3180	cgd6_3180	1	0.039	0	0
CHUDEA6_3190	cgd6_3190	0.949	0.0503	0.0048	0.095427435
CHUDEA6_3200	cgd6_3200	0.974	0.0293	0.0115	0.392491468
CHUDEA6_320	cgd6_320	0.964	0.0427	0.0135	0.316159251
CHUDEA6_3210	cgd6_3210	0.983	0.059	0.0079	0.133898305
CHUDEA6_3220	cgd6_3220	1	0.0636	0	0
CHUDEA6_3230	cgd6_3230	0.987	0.059	0.0054	0.091525424
CHUDEA6_3240	cgd6_3240	0.974	0.0382	0.0118	0.308900524
CHUDEA6_3250	cgd6_3250	0.942	0.0522	0.0267	0.511494253
CHUDEA6_3260	cgd6_3260	0.973	0.0642	0.0123	0.191588785
CHUDEA6_3270	cgd6_3270	0.99	0.0639	0.004	0.062597809
CHUDEA6_3280	cgd6_3280	0.997	0.0529	0.0013	0.024574669
CHUDEA6_3290	cgd6_3290	0.981	0.0528	0.0077	0.145833333
CHUDEA6_3300	cgd6_3300	0.983	0.0711	0.0072	0.101265823
CHUDEA6_3310	cgd6_3310	0.983	0.0419	0.0073	0.174224344

CHUDEA6_3330	cgd6_3330	0.985	0.0505	0.0069	0.136633663
CHUDEA6_3340	cgd6_3340	1	0.0364	0	0
CHUDEA6_3350	cgd6_3350	0.982	0.0601	0.0076	0.126455907
CHUDEA6_3360	cgd6_3360	0.988	0.0517	0.0038	0.073500967
CHUDEA6_3370	cgd6_3370	0.991	0.0702	0.0036	0.051282051
CHUDEA6_3380	cgd6_3380	1	0.034	0	0
CHUDEA6_3390	cgd6_3390	0.977	0.0387	0.0102	0.263565891
CHUDEA6_3400	cgd6_3400	0.983	0.0624	0.0054	0.086538462
CHUDEA6_340	cgd6_340	0.977	0.06	0.0043	0.071666667
CHUDEA6_3410	cgd6_3410	0.987	0.0409	0.0056	0.136919315
CHUDEA6_3420	cgd6_3420	0.984	0.0535	0.0071	0.13271028
CHUDEA6_3430	cgd6_3430	0.969	0.0568	0.0139	0.24471831
CHUDEA6_3440	cgd6_3440	0.979	0.0646	0.0089	0.137770898
CHUDEA6_3450	cgd6_3450	0.992	0.0371	0.0037	0.099730458
CHUDEA6_3460	cgd6_3460	0.907	0.0641	0.0267	0.416536661
CHUDEA6_3470	cgd6_3470	0.996	0.0858	0.0016	0.018648019
CHUDEA6_3480	cgd6_3480	0.996	0.0661	0.0016	0.024205749
CHUDEA6_3490	cgd6_3490	0.971	0.0623	0.0129	0.2070626
CHUDEA6_3500	cgd6_3500	0.957	0.1256	0.0206	0.164012739
CHUDEA6_3510	cgd6_3510	0.98	0.0354	0.0089	0.251412429
CHUDEA6_3530	cgd6_3530	0.984	0.0534	0.0063	0.117977528
CHUDEA6_3540	cgd6_3540	0.99	0.0487	0.004	0.082135524
CHUDEA6_3550	cgd6_3550	0.986	0.0801	0.0071	0.088639201
CHUDEA6_3560	cgd6_3560	0.965	0.0651	0.0151	0.231950845
CHUDEA6_3570	cgd6_3570	0.982	0.0606	0.0079	0.130363036
CHUDEA6_3580	cgd6_3580	0.996	0.035	0.0016	0.045714286
CHUDEA6_3590	cgd6_3590	0.991	0.0552	0.005	0.09057971
CHUDEA6_3600	cgd6_3600	0.98	0.037	0.0091	0.245945946
CHUDEA6_3610	cgd6_3610	0.993	0.0665	0.0028	0.042105263
CHUDEA6_3620	cgd6_3620	0.982	0.0582	0.0082	0.140893471
CHUDEA6_3630	cgd6_3630	0.975	0.0434	0.0107	0.246543779
CHUDEA6_3640	cgd6_3640	0.96	0.0447	0.0191	0.427293065
CHUDEA6_3650	cgd6_3650	0.968	0.0598	0.0136	0.227424749
CHUDEA6_3660	cgd6_3660	0.975	0.0579	0.0115	0.198618307
CHUDEA6_3670	cgd6_3670	0.976	0.0588	0.0103	0.175170068
CHUDEA6_3680	cgd6_3680	0.997	0.0799	0.0015	0.018773467
CHUDEA6_3690	cgd6_3690	0.988	0.0732	0.0044	0.06010929
CHUDEA6_3710	cgd6_3710	1	0.07	0	0
CHUDEA6_3720	cgd6_3720	0.982	0.0951	0.0075	0.078864353
CHUDEA6_3730	cgd6_3730	0.966	0.0765	0.012	0.156862745
CHUDEA6_3740	cgd6_3740	1	0.0919	0	0
CHUDEA6_3750	cgd6_3750	0.972	0.077	0.0127	0.164935065
CHUDEA6_3760	cgd6_3760	0.96	0.0614	0.0157	0.255700326
CHUDEA6_3770	cgd6_3770	0.991	0.0645	0.0041	0.063565891
CHUDEA6_3780	cgd6_3780	0.967	0.0748	0.0158	0.211229947

CHUDEA6_3790	cgd6_3790	1	0.0532	0	0
CHUDEA6_3800	cgd6_3800	0.977	0.0704	0.0097	0.137784091
CHUDEA6_380	cgd6_380	0.979	0.0571	0.0121	0.211908932
CHUDEA6_3810	cgd6_3810	0.978	0.056	0.0091	0.1625
CHUDEA6_3820	cgd6_3820	0.987	0.0483	0.0047	0.097308489
CHUDEA6_3830	cgd6_3830	0.995	0.0735	0	0
CHUDEA6_3840	cgd6_3840	0.979	0.0454	0.0089	0.196035242
CHUDEA6_3850	cgd6_3850	1	0	0	0
CHUDEA6_3860	cgd6_3860	0.992	0.0647	0.0035	0.054095827
CHUDEA6_3870	cgd6_3870	0.972	0.0701	0.0136	0.194008559
CHUDEA6_3880	cgd6_3880	0.972	0.0514	0.0116	0.225680934
CHUDEA6_3890	cgd6_3890	1	0.0242	0	0
CHUDEA6_3900	cgd6_3900	0.971	0.0625	0.0106	0.1696
CHUDEA6_390	cgd6_390	0.955	0.1118	0.0157	0.140429338
CHUDEA6_3910	cgd6_3910	0.983	0.0299	0.0075	0.25083612
CHUDEA6_3920	cgd6_3920	0.934	0.1072	0.0266	0.248134328
CHUDEA6_3930	cgd6_3930	0.819	0.1369	0.0833	0.608473338
CHUDEA6_3950	cgd6_3950	0.974	0.0671	0.0107	0.159463487
CHUDEA6_3960	cgd6_3960	0.984	0.0732	0.007	0.095628415
CHUDEA6_3970	cgd6_3970	0.995	0.1137	0.002	0.01759015
CHUDEA6_3980	cgd6_3980	0.949	0.0651	0.0217	0.333333333
CHUDEA6_3990	cgd6_3990	0.998	0.0279	0.0011	0.039426523
CHUDEA6_4000	cgd6_4000	0.967	0.0663	0.0148	0.223227753
CHUDEA6_400	cgd6_400	0.988	0.0822	0.0052	0.063260341
CHUDEA6_4010	cgd6_4010	0.925	0.0636	0.0325	0.511006289
CHUDEA6_4020	cgd6_4020	0.968	0.0669	0.0135	0.201793722
CHUDEA6_4030	cgd6_4030	0.964	0.0598	0.0124	0.20735786
CHUDEA6_4040	cgd6_4040	0.969	0.0485	0.014	0.288659794
CHUDEA6_4050	cgd6_4050	0.983	0.0581	0.0075	0.12908778
CHUDEA6_4060	cgd6_4060	0.993	0.0195	0.0031	0.158974359
CHUDEA6_4070	cgd6_4070	0.973	0.0431	0.012	0.278422274
CHUDEA6_4080	cgd6_4080	0.97	0.0928	0.0104	0.112068966
CHUDEA6_4090	cgd6_4090	0.942	0.0709	0.0259	0.365303244
CHUDEA6_40	cgd6_40	0.716	0.2841	0.2103	0.740232313
CHUDEA6_410	cgd6_410	0.981	0.083	0.0059	0.071084337
CHUDEA6_4110	cgd6_4110	0.944	0.0706	0.0139	0.196883853
CHUDEA6_4120	cgd6_4120	0.981	0.0297	0.0082	0.276094276
CHUDEA6_4130	cgd6_4130	0.98	0.0604	0.0084	0.139072848
CHUDEA6_4140	cgd6_4140	0.994	0.0599	0.0025	0.041736227
CHUDEA6_4150	cgd6_4150	0.972	0.0609	0.0093	0.15270936
CHUDEA6_4160	cgd6_4160	0.981	0.0663	0.008	0.12066365
CHUDEA6_4170	cgd6_4170	0.96	0.0523	0.0157	0.300191205
CHUDEA6_4180	cgd6_4180	0.987	0.0184	0.0064	0.347826087
CHUDEA6_4190	cgd6_4190	1	0.0253	0	0
CHUDEA6_4200	cgd6_4200	0.996	0.0718	0.0016	0.022284123

CHUDEA6_420	cgd6_420	0.984	0.0529	0.0066	0.124763705
CHUDEA6_4210	cgd6_4210	0.98	0.0508	0.009	0.177165354
CHUDEA6_4220	cgd6_4220	0.997	0.0261	0.0012	0.045977011
CHUDEA6_4230	cgd6_4230	0.89	0.107	0.0566	0.528971963
CHUDEA6_4240	cgd6_4240	0.969	0.056	0.0134	0.239285714
CHUDEA6_4250	cgd6_4250	0.97	0.0667	0.0117	0.175412294
CHUDEA6_4260	cgd6_4260	0.971	0.0911	0.012	0.131723381
CHUDEA6_4270	cgd6_4270	1	0.0848	0	0
CHUDEA6_4280	cgd6_4280	0.934	0.0813	0.0273	0.335793358
CHUDEA6_4290	cgd6_4290	0.958	0.082	0.0127	0.154878049
CHUDEA6_4300	cgd6_4300	0.978	0.0475	0.0085	0.178947368
CHUDEA6_430	cgd6_430	0.955	0.022	0.0177	0.804545455
CHUDEA6_4310	cgd6_4310	0.972	0.078	0.0114	0.146153846
CHUDEA6_4320	cgd6_4320	1	0.0442	0	0
CHUDEA6_4330	cgd6_4330	0.978	0.0554	0.0095	0.171480144
CHUDEA6_4340	cgd6_4340	0.991	0.0431	0.0042	0.097447796
CHUDEA6_4350	cgd6_4350	1	0.0589	0	0
CHUDEA6_4360	cgd6_4360	0.991	0.0396	0.0038	0.095959596
CHUDEA6_4370	cgd6_4370	0.977	0.0728	0.0102	0.14010989
CHUDEA6_4380	cgd6_4380	0.97	0.042	0.0132	0.314285714
CHUDEA6_4390	cgd6_4390	0.986	0.0663	0.0067	0.101055807
CHUDEA6_4400	cgd6_4400	0.991	0.0644	0.0038	0.059006211
CHUDEA6_440	cgd6_440	0.976	0.0603	0.0093	0.154228856
CHUDEA6_4410	cgd6_4410	0.99	0.0521	0.0043	0.082533589
CHUDEA6_4420	cgd6_4420	0.98	0.0561	0.0094	0.167557932
CHUDEA6_4430	cgd6_4430	0.973	0.0427	0.0116	0.271662763
CHUDEA6_4440	cgd6_4440	0.986	0.0727	0.0067	0.09215956
CHUDEA6_4450	cgd6_4450	0.979	0.0921	0.0101	0.109663409
CHUDEA6_4460	cgd6_4460	0.992	0.0748	0.0033	0.044117647
CHUDEA6_4470	cgd6_4470	0.981	0.0546	0.0095	0.173992674
CHUDEA6_4480	cgd6_4480	0.978	0.0589	0.0095	0.161290323
CHUDEA6_4490	cgd6_4490	1	0.0427	0	0
CHUDEA6_4500	cgd6_4500	0.975	0.0336	0.011	0.327380952
CHUDEA6_4510	cgd6_4510	0.983	0.0444	0.0068	0.153153153
CHUDEA6_4520	cgd6_4520	0.974	0.0566	0.0116	0.204946996
CHUDEA6_4530	cgd6_4530	0.927	0.0621	0.0336	0.541062802
CHUDEA6_4540	cgd6_4540	0.997	0.0269	0.0013	0.048327138
CHUDEA6_4550	cgd6_4550	0.976	0.0785	0.0089	0.113375796
CHUDEA6_4560	cgd6_4560	0.984	0.0371	0.0077	0.20754717
CHUDEA6_4570	cgd6_4570	0.994	0.069	0.0027	0.039130435
CHUDEA6_4580	cgd6_4580	0.987	0.0972	0.006	0.061728395
CHUDEA6_4590	cgd6_4590	0.991	0.0318	0.0054	0.169811321
CHUDEA6_4600	cgd6_4600	0.984	0.0608	0.0077	0.126644737
CHUDEA6_460	cgd6_450	0.954	0.0815	0.0096	0.117791411
CHUDEA6_4610	cgd6_4610	1	0.0453	0	0

CHUDEA6_4620	cgd6_4620	1	0.0434	0	0
CHUDEA6_4630	cgd6_4630	0.99	0.0414	0.004	0.096618357
CHUDEA6_4640	cgd6_4640	0.968	0.054	0.0136	0.251851852
CHUDEA6_4650	cgd6_4650	0.989	0.0505	0.0042	0.083168317
CHUDEA6_4660	cgd6_4660	0.989	0.0559	0.0049	0.08765653
CHUDEA6_4670	cgd6_4670	0.979	0.0473	0.0096	0.202959831
CHUDEA6_4680	cgd6_4680	0.977	0.0533	0.0115	0.21575985
CHUDEA6_4690	cgd6_4690	0.974	0.0459	0.0119	0.259259259
CHUDEA6_4700	cgd6_4700	0.979	0.0453	0.0095	0.209713024
CHUDEA6_470	cgd6_470	0.958	0.0793	0.0173	0.21815889
CHUDEA6_4710	cgd6_4710	0.964	0.0614	0.0144	0.234527687
CHUDEA6_4720	cgd6_4720	0.985	0.038	0.0065	0.171052632
CHUDEA6_4730	cgd6_4730	0.971	0.0552	0.0135	0.244565217
CHUDEA6_4740	cgd6_4740	0.841	0.1706	0.0842	0.493552169
CHUDEA6_4750	cgd6_4750	0.983	0.0599	0.0085	0.141903172
CHUDEA6_4760	cgd6_4760	1	0.0642	0	0
CHUDEA6_4770	cgd6_4770	0.988	0.0529	0.0065	0.122873346
CHUDEA6_4780	cgd6_4780	0.978	0.061	0.01	0.163934426
CHUDEA6_4790	cgd6_4790	0.967	0.0623	0.0135	0.216693419
CHUDEA6_4800	cgd6_4800	0.977	0.0857	0.0097	0.113185531
CHUDEA6_480	cgd6_480	0.975	0.0594	0.0106	0.178451178
CHUDEA6_4810	cgd6_4810	1	0.1066	0	0
CHUDEA6_4820	cgd6_4820	1	0.1063	0	0
CHUDEA6_4830	cgd6_4830	0.991	0.054	0.0039	0.072222222
CHUDEA6_4840	cgd6_4840	0.985	0.0599	0.0051	0.085141903
CHUDEA6_4850	cgd6_4850	0.966	0.0706	0.016	0.226628895
CHUDEA6_4860	cgd6_4860	0.984	0.0748	0.0006	0.00802139
CHUDEA6_4870	cgd6_4870	0.984	0.0366	0.0046	0.12568306
CHUDEA6_4880	cgd6_4880	0.988	0.0551	0.0066	0.119782214
CHUDEA6_4890	cgd6_4890	0.984	0.0371	0.0074	0.199460916
CHUDEA6_4900	cgd6_4900	0.989	0.0583	0.0048	0.082332762
CHUDEA6_490	cgd6_490	0.977	0.1375	0.0071	0.051636364
CHUDEA6_4910	cgd6_4910	0.986	0.0415	0.0057	0.137349398
CHUDEA6_4920	cgd6_4920	0.99	0.0573	0.0046	0.080279232
CHUDEA6_4930	cgd6_4930	0.989	0.0588	0.0048	0.081632653
CHUDEA6_4940	cgd6_4940	0.993	0.0613	0.0028	0.045676998
CHUDEA6_4950	cgd6_4950	0.985	0.0452	0.0069	0.152654867
CHUDEA6_4960	cgd6_4960	0.971	0.0507	0.0089	0.175542406
CHUDEA6_4970	cgd6_4970	0.995	0.0575	0.0021	0.036521739
CHUDEA6_4980	cgd6_4980	0.881	0	0.0673	1000
CHUDEA6_4990	cgd6_4990	0.971	0.0507	0.0103	0.203155819
CHUDEA6_5000	cgd6_5000	0.974	0.0819	0.0118	0.144078144
CHUDEA6_500	cgd6_500	0.927	0.082	0.0133	0.162195122
CHUDEA6_5010	cgd6_5010	0.971	0.0481	0.0137	0.284823285
CHUDEA6_5030	cgd6_5030	0.983	0.0216	0.007	0.324074074

CHUDEA6_5040	cgd6_5040	0.979	0.0765	0.0091	0.118954248
CHUDEA6_5050	cgd6_5050	0.981	0.0944	0.008	0.084745763
CHUDEA6_5060	cgd6_5060	0.994	0.0555	0.0026	0.046846847
CHUDEA6_5070	cgd6_5070	0.99	0.0293	0.0047	0.160409556
CHUDEA6_5080	cgd6_5080	0.991	0.0373	0.004	0.107238606
CHUDEA6_5090	cgd6_5090	0.969	0.041	0.0133	0.324390244
CHUDEA6_5100	cgd6_5100	0.989	0.0675	0.0047	0.06962963
CHUDEA6_510	cgd6_510	0.982	0.1147	0.0048	0.0418483
CHUDEA6_5110	cgd6_5110	0.936	0.074	0.0223	0.301351351
CHUDEA6_5120	cgd6_5120	0.977	0.0521	0.0086	0.165067179
CHUDEA6_5130	cgd6_5130	0.985	0.0756	0.0064	0.084656085
CHUDEA6_5140	cgd6_5140	0.996	0.074	0.0018	0.024324324
CHUDEA6_5150	cgd6_5150	0.989	0.0704	0.0045	0.063920455
CHUDEA6_5160	cgd6_5160	0.974	0.0466	0.0122	0.261802575
CHUDEA6_5170	cgd6_5170	0.978	0.036	0.01	0.277777778
CHUDEA6_5180	cgd6_5180	0.992	0.0789	0.0034	0.043092522
CHUDEA6_520	cgd6_520	0.956	0.074	0.0072	0.097297297
CHUDEA6_5220	cgd6_5220	0.98	0.0278	0.0042	0.151079137
CHUDEA6_5230	cgd6_5230	0.955	0.0372	0.0058	0.155913978
CHUDEA6_5240	cgd6_5240	0.98	0.0458	0.007	0.152838428
CHUDEA6_5250	cgd6_5250	0.982	0.0602	0.0067	0.111295681
CHUDEA6_5260	cgd6_5260	0.984	0.0903	0.007	0.07751938
CHUDEA6_5270	cgd6_5270	0.887	0.1465	0.0595	0.406143345
CHUDEA6_5280	cgd6_5280	0.97	0.0683	0.0135	0.197657394
CHUDEA6_5290	cgd6_5290	0.981	0.0593	0.0082	0.138279933
CHUDEA6_5300	cgd6_5300	0.964	0.0533	0.0156	0.292682927
CHUDEA6_5310	cgd6_5310	0.976	0.0431	0.0112	0.259860789
CHUDEA6_5320	cgd6_5320	0.972	0.0671	0.0117	0.174366617
CHUDEA6_5330	cgd6_5330	0.98	0.0574	0.009	0.156794425
CHUDEA6_5340	cgd6_5340	0.979	0.0601	0.0091	0.151414309
CHUDEA6_5350	cgd6_5350	0.989	0.0812	0.005	0.061576355
CHUDEA6_5360	cgd6_5360	0.988	0.0628	0.0051	0.081210191
CHUDEA6_5370	cgd6_5370	0.942	0.0598	0.0266	0.444816054
CHUDEA6_5380	cgd6_5380	0.977	0.0675	0.0103	0.152592593
CHUDEA6_5390	cgd6_5390	0.991	0.0474	0.0039	0.082278481
CHUDEA6_5400	cgd6_5400	0.765	0.1742	0.037	0.212399541
CHUDEA6_540	cgd6_540	0.978	0.0742	0.0086	0.115902965
CHUDEA6_5410	cgd6_5410	0.856	0.1384	0.0508	0.367052023
CHUDEA6_5420	cgd6_5420	0.978	0.0918	0.0094	0.102396514
CHUDEA6_5430	cgd6_5430	0.794	0.078	0.0499	0.63974359
CHUDEA6_5440	cgd6_5440	0.973	0.0538	0.0096	0.178438662
CHUDEA6_5450	cgd6_5450	0.984	0.0599	0.0068	0.113522538
CHUDEA6_5470	cgd6_5470	0.78	0.1606	0.1035	0.644458281
CHUDEA6_5490	cgd5_4600	0.767	0.158	0.0782	0.494936709
CHUDEA6_5490	cgd5_4610	0.695	0.2663	0.1345	0.505069471

CHUDEA6_5500	cgd6_5500	0.9	2.0778	0.7929	0.381605544
CHUDEA6_550	cgd6_550	0.984	0.0676	0.0067	0.099112426
CHUDEA6_560	cgd6_560	0.985	0.083	0.0061	0.073493976
CHUDEA6_570	cgd6_570	1	0.0258	0	0
CHUDEA6_580	cgd6_580	0.968	0.0666	0.008	0.12012012
CHUDEA6_590	cgd6_590	0.952	0.0597	0.0097	0.162479062
CHUDEA6_600	cgd6_600	0.953	0.0956	0.0121	0.126569038
CHUDEA6_60	cgd6_60	0.881	0.1613	0.0533	0.330440174
CHUDEA6_610	cgd6_610	0.969	0.0323	0.0071	0.219814241
CHUDEA6_620	cgd6_620	0.994	0.0562	0.0023	0.040925267
CHUDEA6_630	cgd6_630	0.983	0.0281	0.0071	0.252669039
CHUDEA6_640	cgd6_640	0.965	0.051	0.0127	0.249019608
CHUDEA6_650	cgd6_650	0.971	0.0767	0.0077	0.100391134
CHUDEA6_660	cgd6_660	0.978	0.1021	0.0047	0.046033301
CHUDEA6_670	cgd6_670	0.968	0.0822	0.0126	0.153284672
CHUDEA6_680	cgd6_680	0.969	0.1002	0.0109	0.108782435
CHUDEA6_690	cgd6_690	0.991	0.0854	0.0035	0.040983607
CHUDEA6_700	cgd6_700	0.976	0.0874	0.0097	0.110983982
CHUDEA6_70	cgd6_70	0.977	0.053	0.0105	0.198113208
CHUDEA6_710	cgd6_710	0.978	0.1709	0.0042	0.024575775
CHUDEA6_720	cgd6_720	0.97	0.0723	0.0074	0.102351314
CHUDEA6_730	cgd6_730	0.97	0.0632	0.0094	0.148734177
CHUDEA6_740	cgd6_740	0.982	0.0536	0.0065	0.121268657
CHUDEA6_750	cgd6_750	0.973	0.036	0.0111	0.308333333
CHUDEA6_760	cgd6_760	0.981	0.0806	0.0052	0.064516129
CHUDEA6_770	cgd6_770	0.996	0.0768	0.0017	0.022135417
CHUDEA6_780	cgd6_780	0.978	0.0522	0.0092	0.176245211
CHUDEA6_800	cgd6_800	0.985	0.0439	0.0061	0.138952164
CHUDEA6_80	cgd6_80	0.993	0.064	0.0028	0.04375
CHUDEA6_810	cgd6_810	0.976	0.1043	0.009	0.086289549
CHUDEA6_820	cgd6_820	0.997	0.0569	0.0013	0.0228471
CHUDEA6_830	cgd6_830	0.932	0.1087	0.0167	0.153633855
CHUDEA6_840	cgd6_840	0.98	0.0532	0.0089	0.167293233
CHUDEA6_850	cgd6_850	0.985	0.0461	0.0076	0.164859002
CHUDEA6_860	cgd6_860	0.973	0.0681	0.0117	0.171806167
CHUDEA6_870	cgd6_870	0.973	0.0723	0.0113	0.156293223
CHUDEA6_880	cgd6_880	0.98	0.0569	0.0088	0.154657293
CHUDEA6_890	cgd6_890	0.982	0.0575	0.0075	0.130434783
CHUDEA6_900	cgd6_900	0.979	0.0474	0.0073	0.154008439
CHUDEA6_90	cgd6_90	0.986	0.0641	0.0067	0.104524181
CHUDEA6_910	cgd6_910	0.978	0.0926	0.0094	0.101511879
CHUDEA6_920	cgd6_920	0.995	0.0697	0.0021	0.030129125
CHUDEA6_930	cgd6_930	0.977	0.0533	0.0089	0.166979362
CHUDEA6_940	cgd6_940	0.989	0.0976	0.0044	0.045081967
CHUDEA6_950	cgd6_950	0.993	0.0421	0.0029	0.06888361

CHUDEA6_960	cgd6_960	0.986	0.0707	0.006	0.084865629
CHUDEA6_970	cgd6_970	0.961	0.0739	0.016	0.216508796
CHUDEA6_980	cgd6_980	0.985	0.049	0.0065	0.132653061
CHUDEA6_990	cgd6_990	0.979	0.0544	0.009	0.165441176
CHUDEA6_newUdeA_03	cgd2_3560	0.951	0.0368	0.0147	0.399456522
CHUDEA6_new_01	cgd6_new_01	0.973	0.0411	0.014	0.340632603
CHUDEA6_new_02	cgd6_new_02	0.944	0.1054	0.0174	0.165085389
CHUDEA6_new_03	cgd6_new_03	0.974	0.0221	0.0107	0.484162896
CHUDEA6_new_04	cgd6_new_04	0.993	0.0539	0.0031	0.057513915
CHUDEA6_new_05	cgd6_new_05	0.978	0.0865	0.0093	0.107514451
CHUDEA6_new_06	cgd6_new_06	0.957	0.0507	0.0179	0.353057199
CHUDEA6_new_07	cgd6_new_07	1	0.1041	0	0
CHUDEA7_1000	cgd7_1000	0.971	0.066	0.0137	0.207575758
CHUDEA7_100	cgd7_100	0.988	0.062	0.0055	0.088709677
CHUDEA7_1010	cgd7_1010	0.958	0.0733	0.0106	0.144611187
CHUDEA7_1020	cgd7_1020	0.993	0.0315	0.0027	0.085714286
CHUDEA7_1030	cgd7_1030	0.996	0.0222	0.0015	0.067567568
CHUDEA7_1040	cgd7_1040	1	0.064	0	0
CHUDEA7_1050	cgd7_1050	0.973	0.0806	0.0078	0.096774194
CHUDEA7_1060	cgd7_1060	0.98	0.049	0.0077	0.157142857
CHUDEA7_1070	cgd7_1070	1	0.0596	0	0
CHUDEA7_1080	cgd7_1080	0.991	0.0625	0.0033	0.0528
CHUDEA7_1090	cgd7_1090	0.987	0.0791	0.0052	0.06573957
CHUDEA7_10	cgd7_10	0.845	0.1239	0.0647	0.522195319
CHUDEA7_1100	cgd7_1100	0.969	0.0539	0.0128	0.237476809
CHUDEA7_110	cgd7_110	0.99	0.0669	0.0044	0.065769806
CHUDEA7_1110	cgd7_1110	0.946	0.0217	0.0259	1.193548387
CHUDEA7_1120	cgd7_1120	0.983	0.0429	0.006	0.13986014
CHUDEA7_1130	cgd7_1130	0.953	0.0817	0.0159	0.194614443
CHUDEA7_1140	cgd7_1140	0.91	0.1072	0.0537	0.500932836
CHUDEA7_1150	cgd7_1150	0.981	0.064	0.0077	0.1203125
CHUDEA7_1160	cgd7_1160	0.986	0.0649	0.0063	0.097072419
CHUDEA7_1170	cgd7_1170	0.982	0.0494	0.0083	0.168016194
CHUDEA7_1180	cgd7_1180	0.96	0.0451	0.0198	0.43902439
CHUDEA7_1190	cgd7_1190	0.969	0.0712	0.009	0.126404494
CHUDEA7_1200	cgd7_1200	0.962	0.0724	0.0169	0.233425414
CHUDEA7_120	cgd7_120	0.997	0.0564	0.0013	0.023049645
CHUDEA7_1210	cgd7_1210	0.888	0.0394	0.0568	1.441624365
CHUDEA7_1220	cgd7_1220	0.95	0.0521	0.0201	0.385796545
CHUDEA7_1230	cgd7_1230	0.974	0.133	0.0111	0.083458647
CHUDEA7_1240	cgd7_1240	0.976	0.0721	0.0111	0.153952843
CHUDEA7_1250	cgd7_1250	0.99	0	0.0042	1000
CHUDEA7_1260	cgd7_1260	0.991	0.05	0.0037	0.074
CHUDEA7_1270	cgd7_1270	0.931	0.0688	0.033	0.479651163
CHUDEA7_1280	cgd7_1280	0.767	0.151	0.1111	0.735761589

CHUDEA7_1290	cgd7_1290	0.942	0.0836	0.0199	0.238038278
CHUDEA7_1300	cgd7_1300	0.969	0.0731	0.0098	0.134062927
CHUDEA7_130	cgd7_130	1	0.051	0	0
CHUDEA7_1310	cgd7_1310	0.975	0.0499	0.0108	0.216432866
CHUDEA7_1320	cgd7_1320	0.977	0.0351	0.0085	0.242165242
CHUDEA7_1330	cgd7_1330	0.935	0.0642	0.0281	0.437694704
CHUDEA7_1340	cgd7_1340	0.983	0.0545	0.0066	0.121100917
CHUDEA7_1350	cgd7_1350	0.968	0.075	0.0143	0.190666667
CHUDEA7_1360	cgd7_1360	0.954	0.0637	0.0217	0.340659341
CHUDEA7_1370	cgd7_1370	0.891	0.0891	0.0545	0.611672278
CHUDEA7_1380	cgd7_1380	0.985	0.0813	0.0065	0.0799508
CHUDEA7_1390	cgd7_1390	0.985	0.0676	0.007	0.103550296
CHUDEA7_1400	cgd7_1400	0.963	0.0838	0.0172	0.205250597
CHUDEA7_140	cgd7_140	0.973	0.0456	0.0117	0.256578947
CHUDEA7_1410	cgd7_1410	0.976	0.0513	0.0106	0.20662768
CHUDEA7_1420	cgd7_1420	0.976	0.0708	0.0119	0.168079096
CHUDEA7_1430	cgd7_1430	0.974	0.0573	0.0116	0.202443281
CHUDEA7_1440	cgd7_1440	0.991	0.0356	0.0043	0.120786517
CHUDEA7_1450	cgd7_1450	0.97	0.0499	0.014	0.280561122
CHUDEA7_1460	cgd7_1460	1	0.0524	0	0
CHUDEA7_1470	cgd7_1470	0.962	0.056	0.0172	0.307142857
CHUDEA7_1480	cgd7_1480	0.976	0.048	0.0103	0.214583333
CHUDEA7_1490	cgd7_1490	0.993	0.0563	0.0031	0.055062167
CHUDEA7_1500	cgd7_1500	0.982	0.0654	0.0079	0.120795107
CHUDEA7_150	cgd7_150	0.991	0.057	0.0039	0.068421053
CHUDEA7_1510	cgd7_1510	0.966	0.0631	0.0157	0.24881141
CHUDEA7_1520	cgd7_1520	0.966	0.0426	0.0194	0.455399061
CHUDEA7_1530	cgd7_1530	0.973	0.052	0.0107	0.205769231
CHUDEA7_1540	cgd7_1540	0.987	0.0634	0.006	0.094637224
CHUDEA7_1550	cgd7_1550	0.988	0.048	0.005	0.104166667
CHUDEA7_1560	cgd7_1560	0.977	0.0377	0.0108	0.286472149
CHUDEA7_1570	cgd7_1570	0.984	0.0121	0.0067	0.553719008
CHUDEA7_1580	cgd7_1580	0.985	0.061	0.0068	0.11147541
CHUDEA7_1590	cgd7_1590	0.97	0.0431	0.0117	0.271461717
CHUDEA7_1600	cgd7_1600	0.995	0.0353	0.0019	0.053824363
CHUDEA7_160	cgd7_160	0.967	0.0531	0.0138	0.259887006
CHUDEA7_1610	cgd7_1610	0.991	0.0376	0.004	0.106382979
CHUDEA7_1620	cgd7_1620	0.973	0.0379	0.012	0.316622691
CHUDEA7_1630	cgd7_1630	0.962	0.0671	0.0163	0.242921013
CHUDEA7_1640	cgd7_1640	0.998	0.0778	0.0008	0.010282776
CHUDEA7_1650	cgd7_1650	0.994	0.1008	0.0024	0.023809524
CHUDEA7_1660	cgd7_1660	0.996	0.0845	0.0019	0.022485207
CHUDEA7_1670	cgd7_1670	0.997	0.0454	0.0013	0.028634361
CHUDEA7_1680	cgd7_1680	1	0.0587	0	0
CHUDEA7_1690	cgd7_1690	0.997	0.0444	0.0012	0.027027027

CHUDEA7_1700	cgd7_1700	1	0.0296	0	0
CHUDEA7_170	cgd7_170	0.991	0.0313	0.0037	0.118210863
CHUDEA7_1710	cgd7_1710	0.993	0.0527	0.0028	0.05313093
CHUDEA7_1720	cgd7_1720	0.994	0.064	0.0025	0.0390625
CHUDEA7_1730	cgd7_1730	0.985	0.0695	0.0062	0.089208633
CHUDEA7_1740	cgd7_1740	0.984	0.0959	0.0055	0.057351408
CHUDEA7_1750	cgd7_1750	0.983	0.0542	0.0081	0.149446494
CHUDEA7_1760	cgd7_1760	0.981	0.0666	0.0083	0.124624625
CHUDEA7_1770	cgd7_1770	0.991	0.106	0.0041	0.038679245
CHUDEA7_1780	cgd7_1780	0.983	0.0625	0.0076	0.1216
CHUDEA7_1790	cgd7_1790	0.968	0.042	0.0147	0.35
CHUDEA7_1800	cgd7_1800	0.986	0.0736	0.0058	0.078804348
CHUDEA7_180	cgd7_180	0.993	0.0485	0.0028	0.057731959
CHUDEA7_1810	cgd7_1810	0.956	0.0708	0.0215	0.303672316
CHUDEA7_1820	cgd7_1820	1	0.0709	0	0
CHUDEA7_1830	cgd7_1830	0.988	0.0669	0.0058	0.086696562
CHUDEA7_1840	cgd7_1840	0.988	0.061	0.0044	0.072131148
CHUDEA7_1850	cgd7_1850	0.979	0.0413	0.0096	0.232445521
CHUDEA7_1860	cgd7_1860	0.98	0.0323	0.0089	0.275541796
CHUDEA7_1870	cgd7_1870	0.872	0.0272	0.0028	0.102941176
CHUDEA7_1890	cgd7_1890	0.991	0.0629	0.0042	0.066772655
CHUDEA7_1900	cgd7_1900	0.981	0.0545	0.0092	0.168807339
CHUDEA7_190	cgd7_190	0.913	0.1132	0.049	0.432862191
CHUDEA7_1910	cgd7_1910	0.986	0.0613	0.0063	0.102773246
CHUDEA7_1920	cgd7_1920	1	0.0783	0	0
CHUDEA7_1930	cgd7_1930	0.994	0.0793	0.0029	0.036569987
CHUDEA7_1940	cgd7_1940	0.968	0.0415	0.0141	0.339759036
CHUDEA7_1950	cgd7_1950	0.937	0.0518	0.0288	0.555984556
CHUDEA7_1960	cgd7_1960	0.952	0.0623	0.0077	0.123595506
CHUDEA7_1970	cgd7_1970	0.982	0.0609	0.0083	0.136288998
CHUDEA7_1980	cgd7_1980	0.998	0.0693	0.0009	0.012987013
CHUDEA7_1990	cgd7_1990	0.974	0.0513	0.0106	0.20662768
CHUDEA7_2000	cgd7_2000	0.965	0.054	0.0175	0.324074074
CHUDEA7_200	cgd7_200	0.963	0.0474	0.0166	0.35021097
CHUDEA7_2010	cgd7_2010	0.972	0.0494	0.013	0.263157895
CHUDEA7_2020	cgd7_2020	0.985	0.0364	0.0069	0.18956044
CHUDEA7_2030	cgd7_2030	0.987	0.0485	0.0061	0.125773196
CHUDEA7_2040	cgd7_2040	1	0.0344	0	0
CHUDEA7_2050	cgd7_2050	0.977	0.0869	0.0107	0.123130035
CHUDEA7_2060	cgd7_2060	1	0.0684	0	0
CHUDEA7_2070	cgd7_2070	0.972	0.0561	0.0134	0.23885918
CHUDEA7_2080	cgd7_2080	0.98	0.0525	0.0092	0.175238095
CHUDEA7_2090	cgd7_2090	1	0.0654	0	0
CHUDEA7_20	cgd7_20	0.972	0.0759	0.0139	0.183135705
CHUDEA7_2100	cgd7_2100	0.981	0.0824	0.0075	0.091019417

CHUDEA7_210	cgd7_210	0.984	0.0575	0.0068	0.11826087
CHUDEA7_2110	cgd7_2110	0.996	0.0558	0.0016	0.028673835
CHUDEA7_2120	cgd7_2120	0.885	0.2923	0.0749	0.256243585
CHUDEA7_2130	cgd7_2130	0.954	0.0283	0.022	0.777385159
CHUDEA7_2140	cgd7_2140	0.971	0.0746	0.0121	0.162198391
CHUDEA7_2150	cgd7_2150	0.963	0.0617	0.0169	0.273905997
CHUDEA7_2160	cgd7_2160	0.987	0.0506	0.0059	0.116600791
CHUDEA7_2170	cgd7_2170	0.985	0.0638	0.007	0.109717868
CHUDEA7_2180	cgd7_2180	0.965	0.0477	0.0159	0.333333333
CHUDEA7_2190	cgd7_2190	0.959	0.0637	0.0182	0.285714286
CHUDEA7_2200	cgd7_2200	0.919	0.0984	0.0337	0.342479675
CHUDEA7_220	cgd7_220	1	0.0722	0	0
CHUDEA7_2210	cgd7_2210	0.952	0.0643	0.0225	0.34992224
CHUDEA7_2220	cgd7_2220	0.92	0.0421	0.0331	0.786223278
CHUDEA7_2230	cgd7_2230	0.958	0.069	0.0207	0.3
CHUDEA7_2240	cgd7_2240	0.961	0.0737	0.0193	0.261872456
CHUDEA7_2250	cgd7_2250	0.995	0.0307	0.002	0.06514658
CHUDEA7_2260	cgd7_2260	0.973	0.0196	0.0096	0.489795918
CHUDEA7_2270	cgd7_2270	0.949	0.0726	0.0223	0.307162534
CHUDEA7_2280	cgd7_2280	1	0.0408	0	0
CHUDEA7_2290	cgd7_2290	0.99	0.0401	0.0046	0.114713217
CHUDEA7_2300	cgd7_2300	0.994	0.0687	0.0029	0.042212518
CHUDEA7_230	cgd7_230	0.976	0.0498	0.011	0.220883534
CHUDEA7_2310	cgd7_2310	0.966	0.0413	0.0146	0.353510896
CHUDEA7_2320	cgd7_2320	0.973	0.1086	0.0116	0.106813996
CHUDEA7_2330	cgd7_2330	0.995	0.0573	0.0021	0.036649215
CHUDEA7_2340	cgd7_2340	0.948	0.0749	0.0225	0.300400534
CHUDEA7_2350	cgd7_2350	0.955	0.1662	0.0186	0.111913357
CHUDEA7_2360	cgd7_2360	0.989	0.0643	0.0047	0.073094868
CHUDEA7_2370	cgd7_2370	0.965	0.0927	0.0148	0.1596548
CHUDEA7_2380	cgd7_2380	0.975	0.0526	0.0107	0.203422053
CHUDEA7_2390	cgd7_2390	0.963	0.0525	0.0178	0.339047619
CHUDEA7_2400	cgd7_2400	0.975	0.0558	0.0105	0.188172043
CHUDEA7_240	cgd7_240	0.977	0.0482	0.0102	0.211618257
CHUDEA7_2410	cgd7_2410	0.949	0.0549	0.0246	0.448087432
CHUDEA7_2420	cgd7_2420	0.983	0.0567	0.0079	0.139329806
CHUDEA7_2430	cgd7_2430	0.993	0.0819	0.0029	0.035409035
CHUDEA7_2440	cgd7_2440	0.971	0.0475	0.0128	0.269473684
CHUDEA7_2450	cgd7_2450	0.969	0.0252	0.0146	0.579365079
CHUDEA7_2460	cgd7_2460	0.966	0.0559	0.0154	0.27549195
CHUDEA7_2470	cgd7_2470	0.983	0.084	0.0076	0.09047619
CHUDEA7_2480	cgd7_2480	0.988	0.0769	0.0056	0.072821847
CHUDEA7_2490	cgd7_2490	0.979	0.0239	0.0085	0.355648536
CHUDEA7_2500	cgd7_2500	1	0.1013	0	0
CHUDEA7_250	cgd7_250	0.973	0.06	0.0125	0.208333333

CHUDEA7_2510	cgd7_2510	0.984	0.0515	0.0073	0.141747573
CHUDEA7_2520	cgd7_2520	0.958	0.0505	0.02	0.396039604
CHUDEA7_2530	cgd7_2530	0.954	0.0577	0.02	0.346620451
CHUDEA7_2540	cgd7_2540	1	0.0404	0	0
CHUDEA7_2550	cgd7_2550	0.983	0.08	0.0095	0.11875
CHUDEA7_2560	cgd7_2560	0.967	0.0564	0.0144	0.255319149
CHUDEA7_2570	cgd7_2570	0.975	0.0563	0.0114	0.202486679
CHUDEA7_2580	cgd7_2580	0.972	0.0368	0.0121	0.328804348
CHUDEA7_2590	cgd7_2590	0.976	0.0639	0.0122	0.190923318
CHUDEA7_2600	cgd7_2600	0.946	0.0768	0.0244	0.317708333
CHUDEA7_260	cgd7_260	0.99	0.0943	0.0044	0.046659597
CHUDEA7_2610	cgd7_2610	0.983	0.078	0.0074	0.094871795
CHUDEA7_2620	cgd7_2620	0.981	0.0566	0.0083	0.14664311
CHUDEA7_2630	cgd7_2630	0.984	0.0921	0.0071	0.077090119
CHUDEA7_2640	cgd7_2640	0.981	0.0482	0.0087	0.180497925
CHUDEA7_2650	cgd7_2650	0.99	0.0811	0.0042	0.051787916
CHUDEA7_2660	cgd7_2660	0.971	0.0462	0.0138	0.298701299
CHUDEA7_2670	cgd7_2670	0.997	0.0511	0.0013	0.025440313
CHUDEA7_2680	cgd7_2680	0.996	0.0516	0.0019	0.036821705
CHUDEA7_2690	cgd7_2690	0.953	0.0889	0.0214	0.24071991
CHUDEA7_2700	cgd7_2700	0.973	0.0437	0.0119	0.272311213
CHUDEA7_270	cgd7_270	0.986	0.0627	0.0062	0.098883573
CHUDEA7_2710	cgd7_2710	0.986	0.0597	0.0061	0.102177554
CHUDEA7_2720	cgd7_2720	0.978	0.0539	0.0095	0.176252319
CHUDEA7_2730	cgd7_2730	0.98	0.0564	0.0087	0.154255319
CHUDEA7_2740	cgd7_2740	0.994	0.0649	0.0042	0.064714946
CHUDEA7_2750	cgd7_2750	0.965	0.0379	0.0163	0.430079156
CHUDEA7_2760	cgd7_2760	0.967	0.0523	0.0152	0.290630975
CHUDEA7_2770	cgd7_2770	0.975	0.0683	0.0112	0.16398243
CHUDEA7_2780	cgd7_2780	0.973	0.0374	0.0117	0.312834225
CHUDEA7_2790	cgd7_2790	0.962	0.0931	0.0154	0.165413534
CHUDEA7_2800	cgd7_2800	0.994	0.0493	0.0028	0.056795132
CHUDEA7_280	cgd7_280	1	0.0421	0	0
CHUDEA7_2810	cgd7_2810	0.97	0.0778	0.0097	0.124678663
CHUDEA7_2820	cgd7_2820	0.967	0.0766	0.0141	0.184073107
CHUDEA7_2830	cgd7_2830	0.967	0.0421	0.0146	0.346793349
CHUDEA7_2840	cgd7_2840	1	0.0577	0	0
CHUDEA7_2850	cgd7_2850	0.96	0.0563	0.0185	0.328596803
CHUDEA7_2860	cgd7_2860	0.947	0.0451	0.0239	0.529933481
CHUDEA7_2880	cgd7_2880	0.993	0.0294	0.003	0.102040816
CHUDEA7_2890	cgd7_2890	0.943	0.0546	0.0283	0.518315018
CHUDEA7_2900	cgd7_2900	0.978	0.0564	0.0099	0.175531915
CHUDEA7_290	cgd7_290	0.971	0.0479	0.0128	0.267223382
CHUDEA7_2910	cgd7_2910	0.979	0.0835	0.0086	0.102994012
CHUDEA7_2920	cgd7_2920	0.989	0.0702	0.0051	0.072649573

CHUDEA7_2930	cgd7_2930	0.923	0.1075	0.0346	0.321860465
CHUDEA7_2940	cgd7_2940	0.972	0.0897	0.014	0.156075808
CHUDEA7_2950	cgd7_2950	0.978	0.0335	0.0098	0.292537313
CHUDEA7_2960	cgd7_2960	0.925	0.0481	0.034	0.706860707
CHUDEA7_2970	cgd7_2970	0.958	0.0327	0.0191	0.584097859
CHUDEA7_2980	cgd7_2980	0.955	0.0579	0.0209	0.360967185
CHUDEA7_2990	cgd7_2990	0.986	0.0692	0.0059	0.085260116
CHUDEA7_3000	cgd7_3000	0.991	0.0501	0.0041	0.081836327
CHUDEA7_300	cgd7_300	0.985	0.0566	0.0062	0.109540636
CHUDEA7_3010	cgd7_3010	0.985	0.0602	0.0066	0.109634551
CHUDEA7_3020	cgd7_3020	0.972	0.0699	0.0126	0.180257511
CHUDEA7_3030	cgd7_3030	0.993	0.0577	0.0031	0.05372617
CHUDEA7_3040	cgd7_3040	0.978	0.0606	0.0097	0.160066007
CHUDEA7_3050	cgd7_3050	0.962	0.056	0.0169	0.301785714
CHUDEA7_3060	cgd7_3060	0.957	0.0609	0.02	0.328407225
CHUDEA7_3070	cgd7_3070	0.985	0.0546	0.0071	0.13003663
CHUDEA7_3080	cgd7_3080	0.979	0.0669	0.0104	0.155455904
CHUDEA7_3090	cgd7_3090	0.971	0.054	0.0134	0.248148148
CHUDEA7_30	cgd7_30	0.983	0.0744	0.0078	0.10483871
CHUDEA7_3100	cgd7_3100	0.944	0.1024	0.024	0.234375
CHUDEA7_310	cgd7_310	0.993	0.0485	0.0029	0.059793814
CHUDEA7_3110	cgd7_3110	0.99	0.0495	0.0054	0.109090909
CHUDEA7_3120	cgd7_3120	0.986	0.0759	0.0059	0.07773386
CHUDEA7_3130	cgd7_3130	0.987	0.0766	0.0053	0.069190601
CHUDEA7_3140	cgd7_3140	0.973	0.0765	0.0105	0.137254902
CHUDEA7_3150	cgd7_3150	0.964	0.0361	0.0174	0.48199446
CHUDEA7_3160	cgd7_3160	0.979	0.0917	0.009	0.098146129
CHUDEA7_3170	cgd7_3170	0.983	0.0476	0.0077	0.161764706
CHUDEA7_3180	cgd7_3180	0.989	0.0733	0.0051	0.06957708
CHUDEA7_3190	cgd7_3190	0.981	0.0656	0.0087	0.132621951
CHUDEA7_3200	cgd7_3200	0.966	0.0431	0.0162	0.37587007
CHUDEA7_320	cgd7_320	1	0.0418	0	0
CHUDEA7_3210	cgd7_3210	0.978	0.0461	0.0096	0.20824295
CHUDEA7_3220	cgd7_3220	0.953	0.0491	0.0222	0.452138493
CHUDEA7_3230	cgd7_3230	0.97	0.066	0.0118	0.178787879
CHUDEA7_3240	cgd7_3240	0.986	0.0206	0.0061	0.296116505
CHUDEA7_3250	cgd7_3250	0.989	0.0573	0.0051	0.089005236
CHUDEA7_3260	cgd7_3260	0.97	0.0932	0.0143	0.153433476
CHUDEA7_3270	cgd7_3270	0.996	0.0825	0.0019	0.023030303
CHUDEA7_3280	cgd7_3280	0.958	0.0748	0.0196	0.262032086
CHUDEA7_3290	cgd7_3290	0.979	0.0569	0.0099	0.173989455
CHUDEA7_3300	cgd7_3300	0.975	0.0598	0.0134	0.224080268
CHUDEA7_330	cgd7_330	0.987	0.0495	0.0061	0.123232323
CHUDEA7_3310	cgd7_3310	0.979	0.0691	0.0094	0.136034732
CHUDEA7_3320	cgd7_3320	0.981	0.058	0.0088	0.151724138

CHUDEA7_3330	cgd7_3330	0.992	0.0545	0.0033	0.060550459
CHUDEA7_3340	cgd7_3340	0.961	0.0394	0.0184	0.467005076
CHUDEA7_3350	cgd7_3350	0.991	0.0527	0.0048	0.091081594
CHUDEA7_3360	cgd7_3360	0.97	0.0471	0.0137	0.290870488
CHUDEA7_3370	cgd7_3370	0.948	0.052	0.0249	0.478846154
CHUDEA7_3380	cgd7_3380	0.981	0.0539	0.0083	0.153988868
CHUDEA7_3390	cgd7_3390	0.992	0.0502	0.0033	0.065737052
CHUDEA7_3400	cgd7_3400	0.945	0.0529	0.0243	0.459357278
CHUDEA7_340	cgd7_340	0.944	0.0772	0.0253	0.327720207
CHUDEA7_3410	cgd7_3410	0.951	0.063	0.0223	0.353968254
CHUDEA7_3430	cgd7_3430	0.972	0.0625	0.0129	0.2064
CHUDEA7_3440	cgd7_3440	0.957	0.0806	0.0142	0.17617866
CHUDEA7_3450	cgd7_3450	0.981	0.0599	0.0084	0.140233723
CHUDEA7_3460	cgd7_3460	0.945	0.0563	0.0261	0.463587922
CHUDEA7_3470	cgd7_3470	0.967	0.067	0.0152	0.226865672
CHUDEA7_3480	cgd7_3480	0.978	0.0593	0.0094	0.15851602
CHUDEA7_3490	cgd7_3490	0.992	0.0475	0.0038	0.08
CHUDEA7_3500	cgd7_3500	0.97	0.0694	0.0128	0.18443804
CHUDEA7_350	cgd7_350	0.987	0.0466	0.0055	0.118025751
CHUDEA7_3510	cgd7_3510	0.969	0.0394	0.0145	0.368020305
CHUDEA7_3520	cgd7_3520	1	0.1154	0	0
CHUDEA7_3530	cgd7_3530	0.974	0.052	0.0113	0.217307692
CHUDEA7_3540	cgd7_3540	0.994	0.0553	0.0026	0.047016275
CHUDEA7_3550	cgd7_3550	0.936	0.0541	0.0284	0.524953789
CHUDEA7_3560	cgd7_3560	0.965	0.085	0.0175	0.205882353
CHUDEA7_3570	cgd7_3570	0.973	0.0749	0.0126	0.168224299
CHUDEA7_3580	cgd7_3580	0.971	0.0354	0.0131	0.370056497
CHUDEA7_3590	cgd7_3590	0.964	0.0859	0.0151	0.175785797
CHUDEA7_3600	cgd7_3600	0.974	0.0733	0.0117	0.159618008
CHUDEA7_360	cgd7_360	0.998	0.053	0.0007	0.013207547
CHUDEA7_3610	cgd7_3610	0.977	0.0573	0.011	0.191972077
CHUDEA7_3620	cgd7_3620	0.987	0.0603	0.006	0.099502488
CHUDEA7_3630	cgd7_3630	0.992	0.0574	0.0037	0.06445993
CHUDEA7_3640	cgd7_3640	0.967	0.0875	0.0144	0.164571429
CHUDEA7_3650	cgd7_3650	0.989	0.0806	0.0045	0.055831266
CHUDEA7_3660	cgd7_3660	0.996	0.0444	0.0019	0.042792793
CHUDEA7_3670	cgd7_3670	0.989	0.0571	0.0054	0.094570928
CHUDEA7_3680	cgd7_3680	0.991	0.091	0.0039	0.042857143
CHUDEA7_3690	cgd7_3690	0.974	0.0637	0.0118	0.185243328
CHUDEA7_3700	cgd7_3700	0.954	0.0826	0.0221	0.267554479
CHUDEA7_370	cgd7_370	0.979	0.0931	0.0087	0.093447905
CHUDEA7_3710	cgd7_3710	0.981	0.049	0.0089	0.181632653
CHUDEA7_3720	cgd7_3720	0.998	0.0694	0.0008	0.011527378
CHUDEA7_3730	cgd7_3730	0.968	0.0361	0.0148	0.409972299
CHUDEA7_3740	cgd7_3740	0.991	0.0733	0.0037	0.05047749

CHUDEA7_3750	cgd7_3750	0.989	0.0482	0.0055	0.114107884
CHUDEA7_3760	cgd7_3760	0.983	0.0627	0.0079	0.12599681
CHUDEA7_3770	cgd7_3770	0.973	0.0578	0.0123	0.212802768
CHUDEA7_3780	cgd7_3780	0.941	0.0843	0.0244	0.289442467
CHUDEA7_3790	cgd7_3790	0.989	0.0547	0.005	0.091407678
CHUDEA7_3800	cgd7_3800	0.975	0.0639	0.0112	0.175273865
CHUDEA7_380	cgd7_380	0.979	0.0337	0.0092	0.272997033
CHUDEA7_3810	cgd7_3810	0.976	0.055	0.0108	0.196363636
CHUDEA7_3820	cgd7_3820	0.977	0.088	0.0098	0.111363636
CHUDEA7_3830	cgd7_3830	0.98	0.0676	0.0094	0.139053254
CHUDEA7_3840	cgd7_3840	0.975	0.0607	0.0117	0.192751236
CHUDEA7_3850	cgd7_3850	0.975	0.0399	0.0109	0.273182957
CHUDEA7_3860	cgd7_3860	0.978	0.0543	0.0099	0.182320442
CHUDEA7_3870	cgd7_3870	0.968	0.0613	0.014	0.228384992
CHUDEA7_3880	cgd7_3880	0.997	0.0344	0.0018	0.052325581
CHUDEA7_3890	cgd7_3890	0.994	0.0322	0.0024	0.074534161
CHUDEA7_3900	cgd7_3900	0.976	0.0372	0.011	0.295698925
CHUDEA7_390	cgd7_390	0.977	0.0424	0.0102	0.240566038
CHUDEA7_3910	cgd7_3910	0.959	0.0485	0.0197	0.406185567
CHUDEA7_3920	cgd7_3920	0.988	0.0768	0.005	0.065104167
CHUDEA7_3930	cgd7_3930	0.977	0.0428	0.0119	0.278037383
CHUDEA7_3940	cgd7_3940	0.992	0.057	0.0032	0.056140351
CHUDEA7_3950	cgd7_3950	0.979	0.0477	0.0104	0.21802935
CHUDEA7_3960	cgd7_3960	0.969	0.0511	0.0132	0.258317025
CHUDEA7_3970	cgd7_3970	0.988	0.0531	0.0055	0.103578154
CHUDEA7_3980	cgd7_3980	0.985	0.0614	0.0066	0.107491857
CHUDEA7_3990	cgd7_3990	1	0.0244	0	0
CHUDEA7_4000	cgd7_4000	0.968	0.043	0.0143	0.33255814
CHUDEA7_400	cgd7_400	0.988	0.0635	0.0048	0.075590551
CHUDEA7_4010	cgd7_4010	0.971	0.0543	0.013	0.239410681
CHUDEA7_4020	cgd7_4020	0.81	0.1826	0.0099	0.054216867
CHUDEA7_4030	cgd7_4030	0.973	0.0607	0.013	0.21416804
CHUDEA7_4040	cgd7_4040	0.981	0.0295	0.0081	0.274576271
CHUDEA7_4050	cgd7_4050	1	0.0744	0	0
CHUDEA7_4060	cgd7_4060	0.979	0.0711	0.0098	0.137834037
CHUDEA7_4070	cgd7_4070	0.989	0.0946	0.005	0.052854123
CHUDEA7_4080	cgd7_4080	0.984	0.0531	0.0074	0.139359699
CHUDEA7_4090	cgd7_4090	0.983	0.0453	0.0078	0.17218543
CHUDEA7_40	cgd7_40	0.982	0.0619	0.0081	0.13085622
CHUDEA7_4100	cgd7_4100	0.967	0.052	0.0152	0.292307692
CHUDEA7_410	cgd7_410	0.961	0.0274	0.0143	0.52189781
CHUDEA7_4110	cgd7_4110	0.974	0.0702	0.011	0.156695157
CHUDEA7_4120	cgd7_4120	0.976	0.0495	0.0107	0.216161616
CHUDEA7_4130	cgd7_4130	0.99	0.0351	0.0044	0.125356125
CHUDEA7_4140	cgd7_4140	1	0.0826	0	0

CHUDEA7_4150	cgd7_4150	0.976	0.0696	0.0102	0.146551724
CHUDEA7_4160	cgd7_4160	0.992	0.0541	0.0036	0.066543438
CHUDEA7_4170	cgd7_4170	0.984	0.041	0.0071	0.173170732
CHUDEA7_4180	cgd7_4180	0.977	0.0706	0.0107	0.151558074
CHUDEA7_4190	cgd7_4190	0.995	0.0549	0.0024	0.043715847
CHUDEA7_4200	cgd7_4200	0.978	0.0352	0.0106	0.301136364
CHUDEA7_420	cgd7_420	0.916	0.1068	0.0228	0.213483146
CHUDEA7_4210	cgd7_4210	0.988	0.0564	0.0053	0.093971631
CHUDEA7_4220	cgd7_4220	0.996	0.0499	0.0017	0.034068136
CHUDEA7_4230	cgd7_4230	0.962	0.0587	0.0172	0.293015332
CHUDEA7_4240	cgd7_4240	0.993	0.045	0.0034	0.075555556
CHUDEA7_4250	cgd7_4250	0.993	0.0696	0.0028	0.040229885
CHUDEA7_4260	cgd7_4260	0.975	0.0589	0.0113	0.191850594
CHUDEA7_4270	cgd7_4270	0.992	0.0818	0.0036	0.04400978
CHUDEA7_4280	cgd7_4280	0.963	0.0739	0.0116	0.156968877
CHUDEA7_4290	cgd7_4290	0.975	0.061	0.0113	0.185245902
CHUDEA7_4300	cgd7_4300	0.974	0.0614	0.0126	0.205211726
CHUDEA7_430	cgd7_430	0.994	0.0336	0.0023	0.068452381
CHUDEA7_4310	cgd7_4310	0.988	0.0665	0.0054	0.081203008
CHUDEA7_4320	cgd7_4320	0.974	0.0611	0.0124	0.20294599
CHUDEA7_4330	cgd7_4330	0.978	0.0872	0.0124	0.142201835
CHUDEA7_4340	cgd7_4340	0.971	0.0718	0.0131	0.182451253
CHUDEA7_4350	cgd7_4350	0.988	0.066	0.0057	0.086363636
CHUDEA7_4360	cgd7_4360	0.996	0.0584	0	0
CHUDEA7_4370	cgd7_4370	0.992	0.0585	0.0034	0.058119658
CHUDEA7_4380	cgd7_4380	0.995	0.0312	0.0022	0.070512821
CHUDEA7_4390	cgd7_4390	0.987	0.045	0.0056	0.124444444
CHUDEA7_4400	cgd7_4400	0.998	0.0483	0.0009	0.01863354
CHUDEA7_440	cgd7_440	0.963	0.0878	0.0136	0.154897494
CHUDEA7_4410	cgd7_4410	0.978	0.0979	0.0094	0.096016343
CHUDEA7_4420	cgd7_4420	0.979	0.0554	0.0099	0.178700361
CHUDEA7_4430	cgd7_4430	0.833	0.1341	0.0853	0.636092468
CHUDEA7_4440	cgd7_4440	0.993	0.1212	0.0045	0.037128713
CHUDEA7_4450	cgd7_4450	0.979	0.0486	0.0103	0.211934156
CHUDEA7_4460	cgd7_4460	0.995	0.045	0.0026	0.057777778
CHUDEA7_4470	cgd7_4470	1	0.0438	0	0
CHUDEA7_4480	cgd7_4480	0.995	0.038	0.0023	0.060526316
CHUDEA7_4490	cgd7_4490	0.98	0.0791	0.0083	0.104930468
CHUDEA7_4500	cgd7_4500	0.823	0.1324	0.0755	0.570241692
CHUDEA7_450	cgd7_450	0.987	0.0566	0.0052	0.091872792
CHUDEA7_4510	cgd7_4510	0.991	0.0529	0.0037	0.069943289
CHUDEA7_4520	cgd7_4520	0.974	0.0602	0.0112	0.186046512
CHUDEA7_4530	cgd7_4530	0.988	0.0527	0.0054	0.102466793
CHUDEA7_4540	cgd7_4540	1	0.0302	0	0
CHUDEA7_4550	cgd7_4550	1	0.0378	0	0

CHUDEA7_4560	cgd7_4560	0.985	0.0723	0.0061	0.084370678
CHUDEA7_4570	cgd7_4570	0.991	0.0173	0.004	0.231213873
CHUDEA7_4580	cgd7_4580	0.974	0.0487	0.0116	0.238193018
CHUDEA7_4590	cgd7_4590	0.975	0.0424	0.011	0.259433962
CHUDEA7_4600	cgd7_4600	0.993	0.0714	0.0029	0.040616246
CHUDEA7_460	cgd7_460	0.971	0.0744	0.0125	0.168010753
CHUDEA7_4610	cgd7_4610	0.994	0.0516	0.0024	0.046511628
CHUDEA7_4620	cgd7_4620	0.991	0.0646	0.0026	0.040247678
CHUDEA7_4630	cgd7_4630	0.969	0.0631	0.0106	0.167987322
CHUDEA7_4640	cgd7_4640	0.977	0.0327	0.0103	0.314984709
CHUDEA7_4650	cgd7_4650	0.988	0.0617	0.0053	0.085899514
CHUDEA7_4660	cgd7_4660	0.918	0.0874	0.0327	0.374141876
CHUDEA7_4670	cgd7_4670	0.96	0.0469	0.0178	0.379530917
CHUDEA7_4680	cgd7_4680	0.93	0.0789	0.0314	0.397972117
CHUDEA7_4690	cgd7_4690	0.975	0.0679	0.0104	0.153166421
CHUDEA7_4700	cgd7_4700	0.977	0.06	0.0096	0.16
CHUDEA7_470	cgd7_470	0.987	0.0675	0.0052	0.077037037
CHUDEA7_4710	cgd7_4710	0.985	0.0555	0.0069	0.124324324
CHUDEA7_4720	cgd7_4720	0.983	0.0566	0.0076	0.134275618
CHUDEA7_4730	cgd7_4730	0.985	0.0639	0.0066	0.103286385
CHUDEA7_4740	cgd7_4740	0.98	0.0509	0.0091	0.178781925
CHUDEA7_4750	cgd7_4750	0.972	0.0724	0.0119	0.164364641
CHUDEA7_4760	cgd7_4760	1	0.025	0	0
CHUDEA7_4770	cgd7_4770	1	0.0716	0	0
CHUDEA7_4780	cgd7_4780	0.988	0.0321	0.0057	0.177570093
CHUDEA7_4790	cgd7_4790	0.985	0.055	0.0067	0.121818182
CHUDEA7_4800	cgd7_4800	0.98	0.0502	0.0101	0.201195219
CHUDEA7_480	cgd7_480	0.997	0.0953	0.0013	0.013641133
CHUDEA7_4810	cgd7_4810	0.992	0.0484	0.0039	0.080578512
CHUDEA7_4820	cgd7_4820	0.965	0.0837	0.0131	0.15651135
CHUDEA7_4830	cgd7_4830	0.982	0.0533	0.0086	0.161350844
CHUDEA7_4840	cgd7_4840	0.981	0.0417	0.0083	0.199040767
CHUDEA7_4850	cgd7_4850	0.976	0.0358	0.0129	0.360335196
CHUDEA7_4860	cgd7_4860	0.971	0.0651	0.0132	0.202764977
CHUDEA7_4870	cgd7_4870	1	0.0458	0	0
CHUDEA7_4880	cgd7_4880	0.954	0.0551	0.0187	0.33938294
CHUDEA7_4890	cgd7_4890	0.932	0.0503	0.0297	0.590457256
CHUDEA7_4900	cgd7_4900	0.949	0.0389	0.0218	0.560411311
CHUDEA7_490	cgd7_490	0.986	0.0543	0.0059	0.108655617
CHUDEA7_4910	cgd7_4910	0.997	0.0408	0.0013	0.031862745
CHUDEA7_4920	cgd7_4920	0.97	0.037	0.0139	0.375675676
CHUDEA7_4930	cgd7_4930	0.991	0.0288	0.004	0.138888889
CHUDEA7_4940	cgd7_4940	0.993	0.0572	0.0029	0.050699301
CHUDEA7_4950	cgd7_4950	0.986	0.0378	0.0064	0.169312169
CHUDEA7_4960	cgd7_4960	0.968	0.0546	0.0148	0.271062271

CHUDEA7_4970	cgd7_4970	0.983	0.0725	0.007	0.096551724
CHUDEA7_4980	cgd7_4980	0.972	0.0587	0.0103	0.175468484
CHUDEA7_4990	cgd7_4990	0.971	0.0578	0.012	0.207612457
CHUDEA7_5000	cgd7_5000	0.998	0.029	0.0007	0.024137931
CHUDEA7_500	cgd7_500	0.938	0.0753	0.0144	0.19123506
CHUDEA7_5010	cgd7_5010	0.975	0.0858	0.0076	0.088578089
CHUDEA7_5020	cgd7_5020	0.985	0.0633	0.0054	0.085308057
CHUDEA7_5030	cgd7_5030	0.981	0.0665	0.0081	0.121804511
CHUDEA7_5040	cgd7_5040	0.967	0.0643	0.015	0.233281493
CHUDEA7_5050	cgd7_5050	0.967	0.0659	0.0148	0.224582701
CHUDEA7_505	cgd7_505	1	0.0142	0	0
CHUDEA7_5060	cgd7_5060	1	0.0512	0	0
CHUDEA7_5070	cgd7_5070	0.983	0.0742	0.0078	0.105121294
CHUDEA7_5080	cgd7_5080	0.979	0.0424	0.0099	0.233490566
CHUDEA7_5090	cgd7_5090	0.983	0.0538	0.0067	0.124535316
CHUDEA7_50	cgd7_50	0.968	0.0199	0.0147	0.738693467
CHUDEA7_5100	cgd7_5100	0.984	0.0432	0.0072	0.166666667
CHUDEA7_5110	cgd7_5110	0.969	0.0449	0.0142	0.316258352
CHUDEA7_5120	cgd7_5120	0.986	0.0296	0.006	0.202702703
CHUDEA7_5130	cgd7_5130	0.939	0.098	0.0219	0.223469388
CHUDEA7_5140	cgd7_5140	0.984	0.0691	0.0103	0.149059334
CHUDEA7_5150	cgd7_5150	0.986	0.0848	0.0054	0.063679245
CHUDEA7_5160	cgd7_5160	0.983	0.0442	0.0074	0.167420814
CHUDEA7_5170	cgd7_5170	0.983	0.0571	0.0076	0.133099825
CHUDEA7_5180	cgd7_5180	0.998	0.0428	0.001	0.023364486
CHUDEA7_5190	cgd7_5190	0.978	0.039	0.0102	0.261538462
CHUDEA7_5200	cgd7_5200	0.972	0.0636	0.0112	0.176100629
CHUDEA7_520	cgd7_520	0.969	0.0905	0.0091	0.100552486
CHUDEA7_5210	cgd7_5210	0.981	0.0568	0.0085	0.149647887
CHUDEA7_5220	cgd7_5220	0.97	0.0474	0.014	0.29535865
CHUDEA7_5230	cgd7_5230	0.97	0.0186	0.013	0.698924731
CHUDEA7_5240	cgd7_5240	0.98	0.0739	0.0077	0.104194858
CHUDEA7_5250	cgd7_5250	0.972	0.0709	0.0123	0.17348378
CHUDEA7_5260	cgd7_5260	0.975	0.0343	0.0108	0.314868805
CHUDEA7_5270	cgd7_5270	1	0.0543	0	0
CHUDEA7_5280	cgd7_5280	0.99	0.0548	0.004	0.072992701
CHUDEA7_5290	cgd7_5290	0.976	0.0562	0.0105	0.18683274
CHUDEA7_5300	cgd7_5300	0.975	0.0291	0.0122	0.419243986
CHUDEA7_530	cgd7_530	0.991	0.0376	0.0038	0.10106383
CHUDEA7_5310	cgd7_5310	0.975	0.0554	0.0114	0.205776173
CHUDEA7_5320	cgd7_5320	0.973	0.0751	0.014	0.186418109
CHUDEA7_5330	cgd7_5330	0.971	0.0587	0.0139	0.236797274
CHUDEA7_5340	cgd7_5340	0.982	0.0731	0.0078	0.106703146
CHUDEA7_5350	cgd7_5350	0.969	0.0409	0.0139	0.339853301
CHUDEA7_5360	cgd7_5360	0.979	0.0489	0.0098	0.200408998

CHUDEA7_5370	cgd7_5370	0.957	0.0691	0.0204	0.295224313
CHUDEA7_5380	cgd7_5380	0.981	0.0831	0.008	0.096269555
CHUDEA7_5390	cgd7_5390	0.963	0.0369	0.0174	0.471544715
CHUDEA7_5400	cgd7_5400	0.977	0.1004	0.0096	0.09561753
CHUDEA7_540	cgd7_540	0.983	0.0409	0.0081	0.19804401
CHUDEA7_5410	cgd7_5410	0.975	0.0504	0.0111	0.220238095
CHUDEA7_5420	cgd7_5420	0.956	0.0388	0.0213	0.548969072
CHUDEA7_5430	cgd7_5430	0.966	0.0715	0.0159	0.222377622
CHUDEA7_5440	cgd7_5440	0.979	0.0605	0.0097	0.160330579
CHUDEA7_5450	cgd7_5450	0.982	0.094	0.007	0.074468085
CHUDEA7_5460	cgd7_5460	0.987	0.0506	0.0056	0.110671937
CHUDEA7_5470	cgd7_5470	0.965	0.0874	0.0158	0.180778032
CHUDEA7_5480	cgd7_5480	0.966	0.0576	0.016	0.277777778
CHUDEA7_5490	cgd7_5490	0.98	0.0376	0.0105	0.279255319
CHUDEA7_5500	cgd7_5500	0.989	0.0677	0.0049	0.072378139
CHUDEA7_550	cgd7_550	1	0.0496	0	0
CHUDEA7_5510	cgd7_5510	0.891	0.062	0.0614	0.990322581
CHUDEA7_5530	cgd7_5530	0.958	0.0429	0.0191	0.445221445
CHUDEA7_560	cgd7_560	0.979	0.0486	0.0089	0.183127572
CHUDEA7_570	cgd7_570	0.992	0.0704	0.0033	0.046875
CHUDEA7_580	cgd7_580	0.979	0.0633	0.0093	0.146919431
CHUDEA7_590	cgd7_590	0.996	0.0315	0.0019	0.06031746
CHUDEA7_600	cgd7_600	0.976	0.0507	0.0139	0.274161736
CHUDEA7_60	cgd7_60	0.979	0.0512	0.0092	0.1796875
CHUDEA7_610	cgd7_610	0.942	0.0654	0.0173	0.264525994
CHUDEA7_620	cgd7_620	0.98	0.0708	0.0086	0.121468927
CHUDEA7_630	cgd7_630	0.971	0.0912	0.0105	0.115131579
CHUDEA7_640	cgd7_640	0.965	0.056	0.0093	0.166071429
CHUDEA7_650	cgd7_650	0.982	0.0552	0.0064	0.115942029
CHUDEA7_660	cgd7_660	0.982	0.0623	0.0094	0.150882825
CHUDEA7_670	cgd7_670	0.984	0.0424	0.0066	0.155660377
CHUDEA7_680	cgd7_680	0.985	0.0552	0.0058	0.105072464
CHUDEA7_690	cgd7_690	0.99	0.0252	0.0048	0.19047619
CHUDEA7_700	cgd7_700	0.983	0.0857	0.0071	0.082847141
CHUDEA7_70	cgd7_70	0.988	0.0731	0.0051	0.069767442
CHUDEA7_710	cgd7_710	0.973	0.0406	0.0129	0.31773399
CHUDEA7_720	cgd7_720	0.981	0.0584	0.008	0.136986301
CHUDEA7_730	cgd7_730	0.986	0.11	0.0058	0.052727273
CHUDEA7_740	cgd7_740	0.958	0.0603	0.0178	0.295190713
CHUDEA7_750	cgd7_750	0.958	0.0586	0.0195	0.332764505
CHUDEA7_760	cgd7_760	0.984	0.0972	0.0067	0.068930041
CHUDEA7_770	cgd7_770	0.971	0.05	0.0135	0.27
CHUDEA7_780	cgd7_780	0.98	0.0559	0.009	0.161001789
CHUDEA7_790	cgd7_790	0.982	0.0318	0.005	0.157232704
CHUDEA7_800	cgd7_800	0.953	0.0577	0.0227	0.393414211

CHUDEA7_80	cgd7_80	0.984	0.0564	0.0072	0.127659574
CHUDEA7_810	cgd7_810	1	0.0412	0	0
CHUDEA7_820	cgd7_820	0.994	0.044	0.0026	0.059090909
CHUDEA7_830	cgd7_830	0.993	0.0864	0.0028	0.032407407
CHUDEA7_840	cgd7_840	0.985	0.067	0.0065	0.097014925
CHUDEA7_850	cgd7_850	0.987	0.0665	0.0057	0.085714286
CHUDEA7_860	cgd7_860	0.968	0.0544	0.0136	0.25
CHUDEA7_870	cgd7_870	0.973	0.0627	0.0113	0.180223285
CHUDEA7_880	cgd7_880	0.995	0.0504	0.0022	0.043650794
CHUDEA7_890	cgd7_890	0.95	0.07	0.0232	0.331428571
CHUDEA7_900	cgd7_900	0.958	0.031	0.0178	0.574193548
CHUDEA7_90	cgd7_90	0.978	0.0787	0.0111	0.141041931
CHUDEA7_910	cgd7_910	0.987	0.0888	0.0054	0.060810811
CHUDEA7_920	cgd7_920	0.975	0.0444	0.0106	0.238738739
CHUDEA7_930	cgd7_930	0.978	0.0403	0.0119	0.29528536
CHUDEA7_940	cgd7_940	1	0.0467	0	0
CHUDEA7_950	cgd7_950	0.998	0.0699	0.001	0.014306152
CHUDEA7_960	cgd7_960	0.981	0.0608	0.0082	0.134868421
CHUDEA7_970	cgd7_970	0.978	0.0594	0.0092	0.154882155
CHUDEA7_980	cgd7_980	0.97	0.0611	0.0125	0.204582651
CHUDEA7_990	cgd7_990	0.987	0.0484	0.0056	0.115702479
CHUDEA7_new_01	cgd7_new_01	0.965	0.0439	0.0174	0.396355353
CHUDEA7_new_02	cgd7_new_02	0.98	0.0514	0.0084	0.163424125
CHUDEA7_new_03	cgd7_new_03	0.987	0.0578	0.0059	0.102076125
CHUDEA7_new_04	cgd7_new_04	0.915	0.0451	0.0332	0.736141907
CHUDEA7_new_05	cgd7_new_05	0.964	0.0755	0.0155	0.205298013
CHUDEA7_new_06	cgd7_new_06	0.976	0.0498	0.0108	0.21686747
CHUDEA7_new_07	cgd7_new_07	0.987	0.0628	0.0057	0.090764331
CHUDEA7_new_08	cgd7_new_08	1	0.0781	0	0
CHUDEA7_new_09	cgd7_new_09	0.962	0.0843	0.0229	0.271648873
CHUDEA7_new_10	cgd7_new_10	0.995	0.0652	0.0026	0.039877301
CHUDEA7_new_11	cgd7_new_11	0.941	0.0473	0.0321	0.678646934
CHUDEA7_new_12	cgd7_new_12	0.942	0.0815	0.0268	0.328834356
CHUDEA7_new_13	cgd7_new_13	0.944	0.0598	0.0254	0.424749164
CHUDEA7_new_14	cgd7_new_14	0.996	0.0826	0.0019	0.023002421
CHUDEA7_new_15	cgd7_new_15	0.972	0.0597	0.0123	0.206030151
CHUDEA7_new_17	cgd7_new_17	1	0.0366	0	0
CHUDEA8_1000	cgd8_1000	0.967	0.0678	0.0145	0.213864307
CHUDEA8_100	cgd8_100	0.968	0.0438	0.0143	0.326484018
CHUDEA8_1010	cgd8_1010	0.966	0.0675	0.0165	0.244444444
CHUDEA8_1020	cgd8_1020	0.961	0.0863	0.016	0.185399768
CHUDEA8_1030	cgd8_1030	0.995	0.0657	0.0018	0.02739726
CHUDEA8_1040	cgd8_1040	1	0.0461	0	0
CHUDEA8_1050	cgd8_1050	0.951	0.0514	0.0184	0.357976654
CHUDEA8_1060	cgd8_1060	0.958	0.0772	0.0178	0.230569948

CHUDEA8_1070	cgd8_1070	0.99	0.0805	0.0042	0.052173913
CHUDEA8_1080	cgd8_1080	0.982	0.0599	0.0086	0.143572621
CHUDEA8_1090	cgd8_1090	0.948	0.0766	0.0222	0.289817232
CHUDEA8_1100	cgd8_1100	0.981	0.0831	0.007	0.08423586
CHUDEA8_110	cgd8_110	0.993	0.0109	0.0032	0.293577982
CHUDEA8_1110	cgd8_1110	0.995	0.0151	0.0019	0.125827815
CHUDEA8_1120	cgd8_1120	0.989	0.0596	0.0045	0.075503356
CHUDEA8_1130	cgd8_1130	0.984	0.0775	0.0064	0.082580645
CHUDEA8_1140	cgd8_1140	0.99	0.0812	0.0039	0.048029557
CHUDEA8_1150	cgd8_1150	0.991	0.0493	0.0036	0.073022312
CHUDEA8_1160	cgd8_1160	0.973	0.1451	0.0094	0.064782908
CHUDEA8_1170	cgd8_1170	0.985	0.0688	0.0064	0.093023256
CHUDEA8_1180	cgd8_1180	0.985	0.0684	0.0057	0.083333333
CHUDEA8_1190	cgd8_1190	0.955	0.0146	0.0173	1.184931507
CHUDEA8_1200	cgd8_1200	0.973	0.0534	0.0091	0.170411985
CHUDEA8_120	cgd8_120	0.959	0.0338	0.0184	0.544378698
CHUDEA8_1210	cgd8_1210	0.994	0.0706	0.0024	0.033994334
CHUDEA8_1220	cgd8_1220	0.819	0.1257	0.0178	0.141607001
CHUDEA8_1230	cgd8_1230	0.988	0.0701	0.0016	0.022824536
CHUDEA8_1240	cgd8_1240	0.944	0.0842	0.0111	0.131828979
CHUDEA8_1250	cgd8_1250	0.978	0.0849	0.0056	0.065959953
CHUDEA8_1260	cgd8_1260	0.968	0.0824	0.0137	0.166262136
CHUDEA8_1270	cgd8_1270	0.989	0.0757	0.0047	0.062087186
CHUDEA8_1280	cgd8_1280	1	0.0451	0	0
CHUDEA8_1290	cgd8_1290	0.978	0.0562	0.0094	0.167259786
CHUDEA8_1300	cgd8_1300	0.982	0.0682	0.0076	0.11143695
CHUDEA8_130	cgd8_130	0.976	0.0322	0.0105	0.326086957
CHUDEA8_1310	cgd8_1310	0.981	0.0329	0.0062	0.188449848
CHUDEA8_1320	cgd8_1320	0.966	0.1081	0.014	0.129509713
CHUDEA8_1330	cgd8_1330	0.901	0.0724	0.0473	0.653314917
CHUDEA8_1340	cgd8_1340	0.944	0.085	0.0173	0.203529412
CHUDEA8_1350	cgd8_1350	0.989	0.1014	0.0048	0.047337278
CHUDEA8_1360	cgd8_1360	0.979	0.0282	0.0086	0.304964539
CHUDEA8_1370	cgd8_1370	0.963	0.0847	0.017	0.200708383
CHUDEA8_1380	cgd8_1380	0.929	0.0712	0.0205	0.287921348
CHUDEA8_1390	cgd8_1390	0.983	0.0617	0.007	0.113452188
CHUDEA8_1400	cgd8_1400	0.97	0.0889	0.0131	0.14735658
CHUDEA8_140	cgd8_140	0.959	0.0512	0.019	0.37109375
CHUDEA8_1410	cgd8_1410	0.99	0.115	0.0037	0.032173913
CHUDEA8_1420	cgd8_1420	0.967	0.0762	0.0125	0.164041995
CHUDEA8_1430	cgd8_1430	0.98	0.1022	0.0077	0.075342466
CHUDEA8_1440	cgd8_1440	0.988	0.0526	0.0054	0.102661597
CHUDEA8_1450	cgd8_1450	0.985	0.0524	0.006	0.114503817
CHUDEA8_1460	cgd8_1460	0.978	0.0608	0.0099	0.162828947
CHUDEA8_1470	cgd8_1470	0.955	0.0671	0.0183	0.272727273

CHUDEA8_1480	cgd8_1480	0.985	0.0663	0.0066	0.099547511
CHUDEA8_1490	cgd8_1490	0.974	0.0442	0.0117	0.264705882
CHUDEA8_1500	cgd8_1500	0.974	0.0515	0.0129	0.250485437
CHUDEA8_150	cgd8_150	0.98	0.0434	0.0096	0.221198157
CHUDEA8_1510	cgd8_1510	0.943	0.0417	0.0239	0.573141487
CHUDEA8_1520	cgd8_1520	0.968	0.0481	0.015	0.311850312
CHUDEA8_1530	cgd8_1530	0.981	0.0635	0.0059	0.092913386
CHUDEA8_1540	cgd8_1540	0.966	0.041	0.0169	0.412195122
CHUDEA8_1550	cgd8_1550	0.966	0.0367	0.0106	0.288828338
CHUDEA8_1560	cgd8_1560	0.989	0.0792	0.0041	0.051767677
CHUDEA8_1570	cgd8_1570	0.949	0.058	0.0131	0.225862069
CHUDEA8_1580	cgd8_1580	0.913	0.0947	0.0219	0.2312566
CHUDEA8_1590	cgd8_1590	0.926	0.0488	0.0369	0.756147541
CHUDEA8_1600	cgd8_1600	0.976	0.0516	0.0092	0.178294574
CHUDEA8_160	cgd8_160	0.98	0.0635	0.009	0.141732283
CHUDEA8_1610	cgd8_1610	0.977	0.0622	0.0101	0.162379421
CHUDEA8_1620	cgd8_1620	0.981	0.0659	0.0075	0.113808801
CHUDEA8_1630	cgd8_1630	0.99	0.0606	0.0048	0.079207921
CHUDEA8_1640	cgd8_1640	0.987	0.0264	0.0057	0.215909091
CHUDEA8_1650	cgd8_1650	0.995	0.0328	0	0
CHUDEA8_1660	cgd8_1660	0.95	0.06	0.0223	0.371666667
CHUDEA8_1670	cgd8_1670	0.998	0.0452	0.0008	0.017699115
CHUDEA8_1680	cgd8_1680	0.986	0.0482	0.0061	0.126556017
CHUDEA8_1690	cgd8_1690	1	0.0357	0	0
CHUDEA8_1700	cgd8_1700	0.97	0.0748	0.0132	0.176470588
CHUDEA8_170	cgd8_170	0.986	0.081	0.0055	0.067901235
CHUDEA8_1710	cgd8_1710	0.974	0.0476	0.0111	0.233193277
CHUDEA8_1720	cgd8_1720	1	0.0697	0	0
CHUDEA8_1730	cgd8_1730	0.994	0.0465	0.0029	0.062365591
CHUDEA8_1740	cgd8_1740	0.989	0.0529	0.0046	0.086956522
CHUDEA8_1750	cgd8_1750	0.923	0.1442	0.0299	0.207350902
CHUDEA8_1760	cgd8_1760	0.981	0.0798	0.0084	0.105263158
CHUDEA8_1770	cgd8_1770	0.902	0.0992	0.0229	0.230846774
CHUDEA8_1780	cgd8_1780	0.98	0.0543	0.0089	0.163904236
CHUDEA8_1790	cgd8_1790	0.963	0.0495	0.0165	0.333333333
CHUDEA8_1800	cgd8_1800	0.962	0.0596	0.0175	0.293624161
CHUDEA8_180	cgd8_180	0.965	0.06	0.0164	0.273333333
CHUDEA8_1810	cgd8_1810	0.973	0.0593	0.0126	0.212478921
CHUDEA8_1820	cgd8_1820	1	0.0367	0	0
CHUDEA8_1830	cgd8_1830	0.989	0.0663	0.0047	0.070889894
CHUDEA8_1840	cgd8_1840	1	0.0194	0	0
CHUDEA8_1850	cgd8_1850	1	0.0443	0	0
CHUDEA8_1860	cgd8_1860	0.977	0.0374	0.01	0.267379679
CHUDEA8_1870	cgd8_1870	0.993	0.0584	0.0028	0.047945205
CHUDEA8_1880	cgd8_1880	0.991	0.1282	0.0035	0.027301092

CHUDEA8_1890	cgd8_1890	0.988	0.0399	0.0048	0.120300752
CHUDEA8_1900	cgd8_1900	0.969	0.0465	0.0125	0.268817204
CHUDEA8_190	cgd8_190	0.974	0.0695	0.012	0.172661871
CHUDEA8_1910	cgd8_1910	0.937	0.0415	0.0198	0.477108434
CHUDEA8_1920	cgd8_1920	0.96	0.0867	0.016	0.184544406
CHUDEA8_1930	cgd8_1930	0.979	0.053	0.0092	0.173584906
CHUDEA8_1940	cgd8_1940	0.989	0.0551	0.005	0.090744102
CHUDEA8_1950	cgd8_1950	0.972	0.0792	0.011	0.138888889
CHUDEA8_1960	cgd8_1960	0.941	0.0614	0.0232	0.377850163
CHUDEA8_1970	cgd8_1970	0.984	0.0927	0.0058	0.062567422
CHUDEA8_1980	cgd8_1980	0.977	0.0291	0.0107	0.367697595
CHUDEA8_1990	cgd8_1990	0.979	0.0466	0.0092	0.197424893
CHUDEA8_2000	cgd8_2000	0.979	0.0746	0.0087	0.116621984
CHUDEA8_200	cgd8_200	0.958	0.0633	0.0182	0.287519747
CHUDEA8_2010	cgd8_2010	0.99	0.0462	0.0044	0.095238095
CHUDEA8_2020	cgd8_2020	0.985	0.0436	0.0065	0.149082569
CHUDEA8_2030	cgd8_2030	0.992	0.042	0.0035	0.083333333
CHUDEA8_2040	cgd8_2040	0.958	0.0567	0.0037	0.065255732
CHUDEA8_2050	cgd8_2050	0.958	0.0705	0.0186	0.263829787
CHUDEA8_2060	cgd8_2060	0.971	0.0658	0.0126	0.191489362
CHUDEA8_2070	cgd8_2070	0.977	0.0716	0.0097	0.13547486
CHUDEA8_2080	cgd8_2080	0.941	0.0573	0.0249	0.434554974
CHUDEA8_2090	cgd8_2090	0.984	0.0659	0.0076	0.115326252
CHUDEA8_20	cgd8_20	0.874	0.1183	0.0507	0.428571429
CHUDEA8_2100	cgd8_2100	0.956	0.0417	0.0194	0.465227818
CHUDEA8_210	cgd8_210	0.992	0.0416	0.0034	0.081730769
CHUDEA8_2110	cgd8_2110	0.991	0.0566	0.0037	0.065371025
CHUDEA8_2120	cgd8_2120	0.992	0.0643	0.0035	0.054432348
CHUDEA8_2130	cgd8_2130	0.986	0.1205	0.0043	0.035684647
CHUDEA8_2140	cgd8_2140	0.982	0.1727	0	0
CHUDEA8_2150	cgd8_2150	0.992	0.087	0.0033	0.037931034
CHUDEA8_2160	cgd8_2160	0.845	0.1161	0.0768	0.661498708
CHUDEA8_2170	cgd8_2170	1	0.0483	0	0
CHUDEA8_2180	cgd8_2180	0.976	0.0311	0.0098	0.31511254
CHUDEA8_2190	cgd8_2190	0.965	0.056	0.0144	0.257142857
CHUDEA8_2200	cgd8_2200	0.988	0.0807	0.0051	0.063197026
CHUDEA8_220	cgd8_220	1	0.0636	0	0
CHUDEA8_2210	cgd8_2210	0.978	0.06	0.0088	0.146666667
CHUDEA8_2220	cgd8_2220	0.974	0.0638	0.0106	0.166144201
CHUDEA8_2230	cgd8_2230	0.993	0.0729	0.003	0.041152263
CHUDEA8_2240	cgd8_2240	0.949	0	0.0233	1000
CHUDEA8_2250	cgd8_2250	0.936	0.1061	0.0141	0.132893497
CHUDEA8_2260	cgd8_2260	0.945	0.0649	0.0246	0.379044684
CHUDEA8_2270	cgd8_2270	0.959	0.0637	0.0151	0.237048666
CHUDEA8_2280	cgd8_2280	0.983	0.1006	0.0071	0.070576541

CHUDEA8_2290	cgd8_2290	0.954	0.0609	0.022	0.361247947
CHUDEA8_2300	cgd8_2300	0.975	0.0861	0.0098	0.113821138
CHUDEA8_230	cgd8_230	0.982	0.0633	0.0096	0.151658768
CHUDEA8_2310	cgd8_2310	0.995	0.0555	0.0023	0.041441441
CHUDEA8_2320	cgd8_2320	0.957	0.0589	0.0195	0.33106961
CHUDEA8_2330	cgd8_2330	0.993	0.0516	0.003	0.058139535
CHUDEA8_2340	cgd8_2340	1	0.057	0	0
CHUDEA8_2350	cgd8_2350	0.982	0.049	0.0074	0.151020408
CHUDEA8_2360	cgd8_2360	0.97	0.0652	0.0129	0.197852761
CHUDEA8_2370	cgd8_2370	0.997	0.072	0.0011	0.015277778
CHUDEA8_2380	cgd8_2380	0.987	0.0485	0.0056	0.115463918
CHUDEA8_2390	cgd8_2390	0.977	0.0432	0.0096	0.222222222
CHUDEA8_2400	cgd8_2400	0.964	0.0227	0.0195	0.859030837
CHUDEA8_240	cgd8_240	0.988	0.0407	0.0052	0.127764128
CHUDEA8_2410	cgd8_2410	0.987	0.0595	0.0062	0.104201681
CHUDEA8_2420	cgd8_2420	0.973	0.0358	0.0116	0.324022346
CHUDEA8_2430	cgd8_2430	0.988	0.0493	0.0046	0.093306288
CHUDEA8_2440	cgd8_2440	0.979	0.0481	0.0092	0.191268191
CHUDEA8_2450	cgd8_2450	0.982	0.0451	0.0076	0.168514412
CHUDEA8_2460	cgd8_2460	0.968	0.0738	0.015	0.203252033
CHUDEA8_2470	cgd8_2470	0.988	0.0628	0.0052	0.082802548
CHUDEA8_2480	cgd8_2480	0.962	0.0477	0.0166	0.348008386
CHUDEA8_2490	cgd8_2490	0.977	0.0759	0.0056	0.073781291
CHUDEA8_2500	cgd8_2500	0.959	0.1052	0.02	0.190114068
CHUDEA8_250	cgd8_250	0.993	0.086	0.0045	0.052325581
CHUDEA8_2510	cgd8_2510	0.966	0.1039	0.015	0.144369586
CHUDEA8_2520	cgd8_2520	0.988	0.0657	0.0052	0.079147641
CHUDEA8_2530	cgd8_2530	0.919	0.0885	0.0206	0.232768362
CHUDEA8_2540	cgd8_2540	1	0.0578	0	0
CHUDEA8_2550	cgd8_2550	0.967	0.0593	0.0142	0.239460371
CHUDEA8_2560	cgd8_2560	0.981	0.0457	0.0083	0.181619256
CHUDEA8_2570	cgd8_2570	0.978	0.0727	0.011	0.15130674
CHUDEA8_2580	cgd8_2580	0.982	0.0772	0.0098	0.126943005
CHUDEA8_2590	cgd8_2590	0.976	0.1127	0.0094	0.083407276
CHUDEA8_2600	cgd8_2600	0.964	0.05	0.0161	0.322
CHUDEA8_260	cgd8_260	0.978	0.04	0.0098	0.245
CHUDEA8_2610	cgd8_2610	0.993	0.0581	0.0032	0.055077453
CHUDEA8_2620	cgd8_2620	0.967	0.0681	0.015	0.220264317
CHUDEA8_2630	cgd8_2630	0.979	0.0266	0.0094	0.353383459
CHUDEA8_2640	cgd8_2640	0.975	0.0491	0.0114	0.232179226
CHUDEA8_2650	cgd8_2650	0.979	0.0575	0.0095	0.165217391
CHUDEA8_2660	cgd8_2660	0.97	0.0705	0.0137	0.194326241
CHUDEA8_2670	cgd8_2670	0.993	0.0649	0.0029	0.044684129
CHUDEA8_2680	cgd8_2680	0.94	0.0504	0.026	0.515873016
CHUDEA8_2690	cgd8_2690	0.938	0.0842	0.0252	0.299287411

CHUDEA8_2700	cgd8_2700	0.983	0.0469	0.0069	0.147121535
CHUDEA8_270	cgd8_270	0.954	0.0453	0.0212	0.46799117
CHUDEA8_2710	cgd8_2710	0.974	0.0462	0.0111	0.24025974
CHUDEA8_2720	cgd8_2720	0.972	0.0611	0.0127	0.207855974
CHUDEA8_2730	cgd8_2730	0.96	0.0534	0.0181	0.338951311
CHUDEA8_2740	cgd8_2740	0.962	0.0335	0.0184	0.549253731
CHUDEA8_2750	cgd8_2750	0.952	0.0754	0.0209	0.277188329
CHUDEA8_2760	cgd8_2760	0.975	0.0455	0.0113	0.248351648
CHUDEA8_2770	cgd8_2770	0.981	0.0669	0.0086	0.128550075
CHUDEA8_2780	cgd8_2780	0.991	0.0834	0.0038	0.045563549
CHUDEA8_2790	cgd8_2790	0.993	0.0665	0.0031	0.046616541
CHUDEA8_2800	cgd8_2800	0.946	0.0991	0.0249	0.251261352
CHUDEA8_280	cgd8_280	0.952	0.0718	0.0208	0.289693593
CHUDEA8_2810	cgd8_2810	0.991	0.0901	0.0037	0.041065483
CHUDEA8_2820	cgd8_2820	0.984	0.0701	0.0071	0.10128388
CHUDEA8_2830	cgd8_2830	0.967	0.0405	0.0157	0.387654321
CHUDEA8_2840	cgd8_2840	0.964	0.0726	0.0151	0.207988981
CHUDEA8_2850	cgd8_2850	0.943	0.0597	0.0261	0.43718593
CHUDEA8_2860	cgd8_2860	0.966	0.0427	0.0156	0.365339578
CHUDEA8_2870	cgd8_2870	1	0.0337	0	0
CHUDEA8_2880	cgd8_2880	0.975	0.0334	0.0115	0.344311377
CHUDEA8_2890	cgd8_2890	0.977	0.0763	0.0101	0.132372215
CHUDEA8_2900	cgd8_2900	0.985	0.0332	0.0066	0.198795181
CHUDEA8_290	cgd8_290	0.984	0.0724	0.0071	0.098066298
CHUDEA8_2910	cgd8_2910	0.99	0.0559	0.0037	0.066189624
CHUDEA8_2920	cgd8_2920	0.958	0.0536	0.0194	0.361940299
CHUDEA8_2930	cgd8_2930	0.999	0.0715	0.0006	0.008391608
CHUDEA8_2940	cgd8_2940	0.991	0.0812	0.004	0.049261084
CHUDEA8_2950	cgd8_2950	0.95	0.0399	0.0252	0.631578947
CHUDEA8_2960	cgd8_2960	0.982	0.0623	0.0078	0.125200642
CHUDEA8_2970	cgd8_2970	0.972	0.078	0.0112	0.143589744
CHUDEA8_2980	cgd8_2980	1	0.063	0	0
CHUDEA8_2990	cgd8_2990	0.983	0.0836	0	0
CHUDEA8_3000	cgd8_3000	0.982	0.0682	0.009	0.131964809
CHUDEA8_300	cgd8_300	0.997	0.0374	0.0012	0.032085561
CHUDEA8_3010	cgd8_3010	0.967	0.0639	0.0149	0.233176839
CHUDEA8_3020	cgd8_3020	0.983	0.0498	0.0073	0.146586345
CHUDEA8_3030	cgd8_3030	0.949	0.0642	0.0185	0.288161994
CHUDEA8_3040	cgd8_3040	0.984	0.0714	0.0073	0.102240896
CHUDEA8_3050	cgd8_3050	0.988	0.0453	0.0045	0.099337748
CHUDEA8_3060	cgd8_3060	0.957	0.0574	0.0168	0.292682927
CHUDEA8_3070	cgd8_3070	0.975	0.095	0.0111	0.116842105
CHUDEA8_3080	cgd8_3080	0.966	0.044	0.0153	0.347727273
CHUDEA8_3090	cgd8_3090	0.983	0.0722	0.0076	0.105263158
CHUDEA8_30	cgd8_30	0.885	0.0945	0.0445	0.470899471

CHUDEA8_3100	cgd8_3100	0.988	0.0599	0.0051	0.085141903
CHUDEA8_310	cgd8_310	0.974	0.0529	0.0092	0.173913043
CHUDEA8_3110	cgd8_3110	0.962	0.0655	0.0167	0.254961832
CHUDEA8_3120	cgd8_3120	0.886	0.0602	0.0543	0.901993355
CHUDEA8_3130	cgd8_3130	0.956	0.0631	0.0197	0.312202853
CHUDEA8_3140	cgd8_3140	0.98	0.0668	0.0085	0.127245509
CHUDEA8_3150	cgd8_3150	0.981	0.1016	0.0081	0.079724409
CHUDEA8_3160	cgd8_3160	0.943	0.0496	0.0253	0.510080645
CHUDEA8_3170	cgd8_3170	1	0.0779	0	0
CHUDEA8_3180	cgd8_3180	0.969	0.0652	0.014	0.214723926
CHUDEA8_3190	cgd8_3190	0.982	0.0582	0.0079	0.135738832
CHUDEA8_3200	cgd8_3200	0.974	0.063	0.0125	0.198412698
CHUDEA8_320	cgd8_320	0.976	0.0669	0.011	0.164424514
CHUDEA8_3210	cgd8_3210	0.975	0.0774	0.0104	0.134366925
CHUDEA8_3220	cgd8_3220	0.984	0.0721	0.0068	0.094313454
CHUDEA8_3230	cgd8_3230	0.976	0.0615	0.0101	0.164227642
CHUDEA8_3240	cgd8_3240	0.983	0.0625	0.0077	0.1232
CHUDEA8_3250	cgd8_3250	0.961	0.0434	0.0171	0.394009217
CHUDEA8_3260	cgd8_3260	0.996	0.0695	0.0016	0.023021583
CHUDEA8_3270	cgd8_3270	0.982	0.0673	0.0078	0.11589896
CHUDEA8_3280	cgd8_3280	0.983	0.0597	0.0078	0.130653266
CHUDEA8_3290	cgd8_3290	0.996	0.0675	0.0018	0.026666667
CHUDEA8_3300	cgd8_3300	0.987	0.0676	0.0062	0.091715976
CHUDEA8_330	cgd8_330	0.97	0.0514	0.0127	0.247081712
CHUDEA8_3310	cgd8_3310	0.976	0.06	0.0109	0.181666667
CHUDEA8_3320	cgd8_3320	0.975	0.0478	0.0117	0.244769874
CHUDEA8_3330	cgd8_3330	1	0.0795	0	0
CHUDEA8_3340	cgd8_3340	0.949	0.09	0.0192	0.213333333
CHUDEA8_3350	cgd8_3350	0.968	0.0708	0.0144	0.203389831
CHUDEA8_3360	cgd8_3360	0.956	0.0397	0.0186	0.468513854
CHUDEA8_3370	cgd8_3370	1	0.0667	0	0
CHUDEA8_3380	cgd8_3380	0.97	0.0791	0.0135	0.170670038
CHUDEA8_3390	cgd8_3390	0.978	0.0772	0.0099	0.128238342
CHUDEA8_3400	cgd8_3400	0.958	0.045	0.0203	0.451111111
CHUDEA8_340	cgd8_340	0.986	0.0746	0.0061	0.081769437
CHUDEA8_3410	cgd8_3410	0.981	0.0394	0.0077	0.195431472
CHUDEA8_3420	cgd8_3420	0.981	0.0541	0.0084	0.155268022
CHUDEA8_3430	cgd8_3430	0.991	0.068	0.0039	0.057352941
CHUDEA8_3440	cgd8_3440	0.989	0.0811	0.0046	0.056720099
CHUDEA8_3450	cgd8_3450	0.995	0.0713	0.0024	0.033660589
CHUDEA8_3460	cgd8_3460	0.986	0.0564	0.0065	0.115248227
CHUDEA8_3470	cgd8_3470	0.973	0.0446	0.0133	0.298206278
CHUDEA8_3480	cgd8_3480	0.993	0.1153	0.0034	0.029488291
CHUDEA8_3490	cgd8_3490	0.953	0.0823	0.0238	0.289185905
CHUDEA8_3500	cgd8_3500	0.962	0.0427	0.017	0.398126464

CHUDEA8_350	cgd8_350	0.986	0.0724	0.0061	0.084254144
CHUDEA8_3510	cgd8_3510	0.993	0.0887	0.0033	0.037204059
CHUDEA8_3520	cgd8_3520	0.928	0.1403	0.0283	0.20171062
CHUDEA8_3530	cgd8_3530	0.971	0.0766	0.0131	0.171018277
CHUDEA8_3540	cgd8_3540	0.915	0.0753	0.0381	0.505976096
CHUDEA8_3550	cgd8_3550	0.761	0.0766	0.1057	1.379895561
CHUDEA8_3560	cgd8_3560	0.944	0.0565	0.0248	0.438938053
CHUDEA8_3570	cgd8_3570	0.95	0.0344	0.0231	0.671511628
CHUDEA8_3580	cgd8_3580	0.982	0.0416	0.0081	0.194711538
CHUDEA8_3590	cgd8_3590	0.959	0.0646	0.0194	0.300309598
CHUDEA8_3600	cgd8_3600	0.963	0.1013	0.0168	0.165844028
CHUDEA8_360	cgd8_360	0.996	0.0474	0.0021	0.044303797
CHUDEA8_3610	cgd8_3610	0.928	0.0401	0.0327	0.815461347
CHUDEA8_3620	cgd8_3620	0.964	0.0447	0.0169	0.378076063
CHUDEA8_3630	cgd8_3630	0.981	0.0382	0.0084	0.219895288
CHUDEA8_3640	cgd8_3640	0.976	0.0629	0.0108	0.171701113
CHUDEA8_3650	cgd8_3650	0.97	0.0469	0.0123	0.262260128
CHUDEA8_3660	cgd8_3660	0.984	0.0616	0.0069	0.112012987
CHUDEA8_3670	cgd8_3670	0.985	0.0513	0.0066	0.128654971
CHUDEA8_3680	cgd8_3680	0.994	0.0697	0.0027	0.038737446
CHUDEA8_3690	cgd8_3690	1	0.0182	0	0
CHUDEA8_3700	cgd8_3700	0.982	0.068	0.0074	0.108823529
CHUDEA8_370	cgd8_370	0.983	0.0509	0.0077	0.151277014
CHUDEA8_3710	cgd8_3710	0.98	0.0601	0.0087	0.144758735
CHUDEA8_3720	cgd8_3720	0.983	0.0685	0.0065	0.094890511
CHUDEA8_3730	cgd8_3730	0.956	0.054	0.0222	0.411111111
CHUDEA8_3740	cgd8_3740	0.998	0.0607	0.001	0.016474465
CHUDEA8_3750	cgd8_3750	0.977	0.0635	0.0093	0.146456693
CHUDEA8_3760	cgd8_3760	0.991	0.0591	0.0042	0.07106599
CHUDEA8_3770	cgd8_3770	0.995	0.0503	0.0022	0.043737575
CHUDEA8_3780	cgd8_3780	0.986	0.0708	0.0057	0.080508475
CHUDEA8_3790	cgd8_3790	0.997	0.0613	0.0013	0.021207178
CHUDEA8_380	cgd8_380	0.971	0.0636	0.0125	0.196540881
CHUDEA8_3810	cgd8_3810	0.977	0.0725	0.0105	0.144827586
CHUDEA8_3820	cgd8_3820	0.967	0.0531	0.0154	0.290018832
CHUDEA8_3830	cgd8_3830	0.987	0.0359	0.0059	0.164345404
CHUDEA8_3840	cgd8_3840	0.966	0.0666	0.0155	0.232732733
CHUDEA8_3850	cgd8_3850	0.973	0.066	0.0115	0.174242424
CHUDEA8_3860	cgd8_3860	0.974	0.0437	0.012	0.274599542
CHUDEA8_3870	cgd8_3870	0.969	0.0631	0.0139	0.220285261
CHUDEA8_3880	cgd8_3880	0.974	0.0428	0.0124	0.289719626
CHUDEA8_3890	cgd8_3890	0.979	0.0471	0.0095	0.201698514
CHUDEA8_3900	cgd8_3900	1	0.0516	0	0
CHUDEA8_390	cgd8_390	0.962	0.0692	0.0121	0.174855491
CHUDEA8_3910	cgd8_3910	0.982	0.0687	0.0082	0.119359534

CHUDEA8_3920	cgd8_3920	0.988	0.0215	0.0055	0.255813953
CHUDEA8_3930	cgd8_3930	0.977	0.0478	0.0104	0.217573222
CHUDEA8_3940	cgd8_3940	0.99	0.0536	0.0044	0.082089552
CHUDEA8_3950	cgd8_3950	0.983	0.0671	0.0074	0.110283159
CHUDEA8_3960	cgd8_3960	0.994	0.0506	0.0027	0.053359684
CHUDEA8_3970	cgd8_3970	0.989	0.0303	0.0023	0.075907591
CHUDEA8_3980	cgd8_3980	0.989	0.0327	0.0047	0.143730887
CHUDEA8_3990	cgd8_3990	0.967	0.0334	0.0128	0.383233533
CHUDEA8_4000	cgd8_4000	0.965	0.0718	0.0082	0.114206128
CHUDEA8_400	cgd8_400	1	0.0341	0	0
CHUDEA8_4010	cgd8_4010	0.971	0.0619	0.0124	0.200323102
CHUDEA8_4020	cgd8_4020	0.957	0.0833	0.0162	0.194477791
CHUDEA8_4030	cgd8_4030	0.975	0.0967	0.0075	0.077559462
CHUDEA8_4040	cgd8_4040	1	0	0	0
CHUDEA8_4050	cgd8_4050	1	0	0	0
CHUDEA8_4060	cgd8_4060	0.993	0.0725	0.0032	0.044137931
CHUDEA8_4070	cgd8_4070	0.973	0.0813	0.011	0.135301353
CHUDEA8_4080	cgd8_4080	0.962	0.0947	0.0132	0.13938754
CHUDEA8_4090	cgd8_4090	0.993	0.0891	0.0029	0.032547699
CHUDEA8_40	cgd8_40	0.791	0.1031	0.1081	1.048496605
CHUDEA8_4100	cgd8_4100	0.99	0.0681	0.0038	0.055800294
CHUDEA8_410	cgd8_410	0.958	0.0392	0.0113	0.288265306
CHUDEA8_4110	cgd8_4110	0.936	0.092	0.0228	0.247826087
CHUDEA8_4120	cgd8_4120	0.996	0.1218	0.0018	0.014778325
CHUDEA8_4130	cgd8_4130	0.98	0.0376	0.0081	0.215425532
CHUDEA8_4140	cgd8_4140	0.907	0.0774	0.0294	0.379844961
CHUDEA8_4150	cgd8_4150	0.968	0.0799	0.0093	0.116395494
CHUDEA8_4160	cgd8_4160	0.978	0.0959	0.0085	0.088633994
CHUDEA8_4170	cgd8_4170	0.959	0.088	0.0099	0.1125
CHUDEA8_4190	cgd8_4180	0.71	0.2067	0.1156	0.559264635
CHUDEA8_4200	cgd8_4200	0.973	0.0524	0.012	0.229007634
CHUDEA8_420	cgd8_420	0.976	0.0571	0.0107	0.187390543
CHUDEA8_4220	cgd8_4220	0.991	0.0396	0.0036	0.090909091
CHUDEA8_4230	cgd8_4230	0.973	0.0658	0.0114	0.17325228
CHUDEA8_4240	cgd8_4240	0.992	0.074	0.0031	0.041891892
CHUDEA8_4250	cgd8_4250	0.983	0.0646	0.0068	0.105263158
CHUDEA8_4260	cgd8_4260	1	0.0549	0	0
CHUDEA8_4270	cgd8_4270	0.978	0.1386	0.0036	0.025974026
CHUDEA8_4280	cgd8_4280	0.987	0.0548	0.0056	0.102189781
CHUDEA8_4290	cgd8_4290	0.97	0.0491	0.007	0.142566191
CHUDEA8_4300	cgd8_4300	1	0.0393	0	0
CHUDEA8_430	cgd8_430	1	0.0745	0	0
CHUDEA8_4310	cgd8_4310	0.947	0.0318	0.0158	0.496855346
CHUDEA8_4320	cgd8_4320	0.971	0.0605	0.0121	0.2
CHUDEA8_4330	cgd8_4330	0.997	0.0319	0.0013	0.040752351

CHUDEA8_4340	cgd8_4340	0.96	0.0645	0.0101	0.156589147
CHUDEA8_4350	cgd8_4350	0.992	0.0376	0.0034	0.090425532
CHUDEA8_4360	cgd8_4360	1	0.0669	0	0
CHUDEA8_4370	cgd8_4370	0.955	0.0637	0.0112	0.175824176
CHUDEA8_4380	cgd8_4380	0.988	0.0387	0.0056	0.144702842
CHUDEA8_4390	cgd8_4390	0.989	0.0288	0.0047	0.163194444
CHUDEA8_4400	cgd8_4400	0.985	0.0883	0.0062	0.070215176
CHUDEA8_440	cgd8_440	1	0.0359	0	0
CHUDEA8_4410	cgd8_4410	0.98	0.0864	0.0082	0.094907407
CHUDEA8_4420	cgd8_4420	0.964	0.0579	0.0152	0.262521589
CHUDEA8_4430	cgd8_4430	1	0.0415	0	0
CHUDEA8_4440	cgd8_4440	0.986	0.0452	0.0053	0.117256637
CHUDEA8_4450	cgd8_4450	0.963	0.0513	0.017	0.331384016
CHUDEA8_4460	cgd8_4460	0.953	0.0826	0.0077	0.093220339
CHUDEA8_4470	cgd8_4470	0.994	0.0536	0.0027	0.050373134
CHUDEA8_4480	cgd8_4480	0.923	0.1834	0.0202	0.110141767
CHUDEA8_4490	cgd8_4490	0.948	0.0975	0.0178	0.182564103
CHUDEA8_4500	cgd8_4500	0.987	0.0547	0.0051	0.093235832
CHUDEA8_450	cgd8_450	0.986	0.1051	0.0057	0.054234063
CHUDEA8_4510	cgd8_4510	0.966	0.0866	0.014	0.161662818
CHUDEA8_4520	cgd8_4520	0.974	0.0569	0.0108	0.189806678
CHUDEA8_4530	cgd8_4530	0.978	0.08	0	0
CHUDEA8_4540	cgd8_4540	0.989	0.0556	0.0048	0.086330935
CHUDEA8_4550	cgd8_4550	0.977	0.0289	0.0099	0.342560554
CHUDEA8_4560	cgd8_4560	0.987	0.0601	0.0054	0.08985025
CHUDEA8_4570	cgd8_4570	0.977	0.0791	0.0086	0.108723135
CHUDEA8_4580	cgd8_4580	0.96	0.0751	0.0152	0.202396804
CHUDEA8_4590	cgd8_4590	0.973	0.0201	0.0106	0.527363184
CHUDEA8_4600	cgd8_4600	0.941	0.1182	0.0149	0.12605753
CHUDEA8_460	cgd8_460	0.986	0.0664	0.0048	0.072289157
CHUDEA8_4610	cgd8_4610	0.947	0.0319	0.0227	0.711598746
CHUDEA8_4620	cgd8_4620	0.986	0.0736	0.0052	0.070652174
CHUDEA8_4630	cgd8_4630	0.988	0.0454	0.0056	0.123348018
CHUDEA8_4640	cgd8_4640	0.988	0.0747	0.005	0.066934404
CHUDEA8_4650	cgd8_4650	0.961	0.1264	0.0156	0.123417722
CHUDEA8_4660	cgd8_4660	0.99	0.0793	0.0041	0.051702396
CHUDEA8_4670	cgd8_4670	0.948	0.0377	0.0219	0.580901857
CHUDEA8_4680	cgd8_4680	0.997	0.0734	0.0012	0.016348774
CHUDEA8_4690	cgd8_4690	0.971	0.0487	0.0127	0.260780287
CHUDEA8_4700	cgd8_4700	0.957	0.1043	0.017	0.162991371
CHUDEA8_470	cgd8_470	0.985	0.1044	0.0067	0.064176245
CHUDEA8_4710	cgd8_4710	0.949	0.105	0.0173	0.164761905
CHUDEA8_4720	cgd8_4720	0.998	0.079	0.001	0.012658228
CHUDEA8_4730	cgd8_4730	0.984	0.0448	0.0074	0.165178571
CHUDEA8_4740	cgd8_4740	0.957	0.0662	0.0185	0.279456193

CHUDEA8_4750	cgd8_4750	0.99	0.0394	0.0039	0.098984772
CHUDEA8_4760	cgd8_4760	0.997	0.0482	0.0012	0.024896266
CHUDEA8_4770	cgd8_4770	0.981	0.0763	0.0079	0.103538663
CHUDEA8_4780	cgd8_4780	0.967	0.0467	0.0113	0.241970021
CHUDEA8_4790	cgd8_4790	0.994	0.148	0.0022	0.014864865
CHUDEA8_4800	cgd8_4800	0.967	0.0549	0.0101	0.183970856
CHUDEA8_480	cgd8_480	0.985	0.0747	0.0052	0.06961178
CHUDEA8_4810	cgd8_4810	0.983	0.0747	0.0049	0.065595716
CHUDEA8_4830	cgd8_4830	0.941	0.0968	0.0081	0.083677686
CHUDEA8_4840	cgd8_4840	0.922	0.1262	0.0206	0.163232964
CHUDEA8_4850	cgd8_4850	0.974	0.0702	0.0097	0.138176638
CHUDEA8_4860	cgd8_4860	0.936	0.056	0.0207	0.369642857
CHUDEA8_4870	cgd8_4870	0.97	0.0416	0.0124	0.298076923
CHUDEA8_4880	cgd8_4880	0.982	0.0506	0.0064	0.126482213
CHUDEA8_4900	cgd8_4900	1	0.0461	0	0
CHUDEA8_490	cgd8_490	0.979	0.0593	0.0092	0.155143339
CHUDEA8_4910	cgd8_4910	0.977	0.0899	0.0091	0.101223582
CHUDEA8_4920	cgd8_4920	0.978	0.079	0.0088	0.111392405
CHUDEA8_4930	cgd8_4930	0.966	0.0395	0.0137	0.346835443
CHUDEA8_4940	cgd8_4940	0.947	0.0769	0.0123	0.159947984
CHUDEA8_4950	cgd8_4950	0.973	0.0425	0.0113	0.265882353
CHUDEA8_4960	cgd8_4960	0.923	0.0514	0.0341	0.663424125
CHUDEA8_4970	cgd8_4970	0.988	0.0802	0.0047	0.058603491
CHUDEA8_4980	cgd8_4980	0.944	0.0969	0.0241	0.24871001
CHUDEA8_4990	cgd8_4990	0.979	0.0337	0.0097	0.287833828
CHUDEA8_5000	cgd8_5000	0.963	0.0627	0.0125	0.199362041
CHUDEA8_500	cgd8_500	1	0.0478	0	0
CHUDEA8_5010	cgd8_5010	0.994	0.0337	0.0026	0.077151335
CHUDEA8_5020	cgd8_5020	0.994	0.045	0.0056	0.124444444
CHUDEA8_5030	cgd8_5030	0.994	0.0421	0.0013	0.03087886
CHUDEA8_5040	cgd8_5040	0.972	0.074	0.0122	0.164864865
CHUDEA8_5050	cgd8_5050	0.968	0.0428	0.0172	0.401869159
CHUDEA8_5060	cgd8_5060	0.993	0.043	0.0028	0.065116279
CHUDEA8_5070	cgd8_5070	1	0.0301	0	0
CHUDEA8_5080	cgd8_5080	0.955	0.099	0.0196	0.197979798
CHUDEA8_5090	cgd8_5090	0.99	0.0365	0.004	0.109589041
CHUDEA8_50	cgd8_50	0.9	0.1171	0.0464	0.396242528
CHUDEA8_5100	cgd8_5100	0.977	0.0419	0.0101	0.241050119
CHUDEA8_510	cgd8_510	0.968	0.037	0.0135	0.364864865
CHUDEA8_5110	cgd8_5110	0.951	0.0275	0.024	0.872727273
CHUDEA8_5120	cgd8_5120	0.987	0.0737	0.0056	0.075983718
CHUDEA8_5130	cgd8_5130	0.984	0.0299	0.0068	0.227424749
CHUDEA8_5140	cgd8_5140	0.98	0.0735	0.0098	0.133333333
CHUDEA8_5150	cgd8_5150	0.979	0.043	0.0102	0.237209302
CHUDEA8_5160	cgd8_5160	0.989	0.0591	0.0049	0.082910321

CHUDEA8_5170	cgd8_5170	0.986	0.0341	0.0055	0.161290323
CHUDEA8_5180	cgd8_5180	0.978	0.0673	0.0098	0.145616642
CHUDEA8_5190	cgd8_5190	0.924	0.0893	0.0352	0.394176932
CHUDEA8_5200	cgd8_5200	0.997	0.0706	0.0014	0.019830028
CHUDEA8_520	cgd8_520	0.82	0.094	0.071	0.755319149
CHUDEA8_5210	cgd8_5210	0.967	0.0431	0.0152	0.352668213
CHUDEA8_5220	cgd8_5220	0.985	0.0497	0.0067	0.134808853
CHUDEA8_5230	cgd8_5230	1	0.0253	0	0
CHUDEA8_5240	cgd8_5240	0.988	0.1142	0.0067	0.058669002
CHUDEA8_5250	cgd8_5250	0.985	0.0525	0.0071	0.135238095
CHUDEA8_5260	cgd8_5260	0.982	0.0593	0.008	0.134907251
CHUDEA8_5270	cgd8_5270	0.98	0.0787	0.009	0.114358323
CHUDEA8_5280	cgd8_5280	0.984	0.0519	0.0074	0.142581888
CHUDEA8_5290	cgd8_5290	0.894	0.0746	0.0315	0.422252011
CHUDEA8_5300	cgd8_5300	0.908	0.0596	0.0453	0.760067114
CHUDEA8_530	cgd8_530	0.992	0.0737	0.0034	0.046132972
CHUDEA8_5310	cgd8_5310	0.934	0.0648	0.0297	0.458333333
CHUDEA8_5330	cgd8_5330	0.979	0.0677	0.0099	0.146233383
CHUDEA8_5340	cgd8_5340	0.97	0.07	0.0143	0.204285714
CHUDEA8_5350	cgd8_5350	0.972	0.0939	0.0134	0.142705005
CHUDEA8_5370	cgd8_5370	0.646	0.1178	0.2218	1.882852292
CHUDEA8_5380	cgd8_5380	0.754	0.1155	0.1068	0.924675325
CHUDEA8_5390	cgd8_5390	0.884	0.0533	0.0566	1.061913696
CHUDEA8_5400	cgd8_5400	0.953	0.0511	0.0225	0.440313112
CHUDEA8_540	cgd8_540	0.975	0.0759	0.0069	0.090909091
CHUDEA8_5410	cgd8_5410	0.967	0.0948	0.015	0.158227848
CHUDEA8_550	cgd8_550	0.977	0.0497	0.0086	0.173038229
CHUDEA8_560	cgd8_560	0.987	0.0533	0.0056	0.105065666
CHUDEA8_570	cgd8_570	0.985	0.0495	0.0062	0.125252525
CHUDEA8_580	cgd8_580	1	0.0785	0	0
CHUDEA8_590	cgd8_590	0.978	0.0939	0.0111	0.118210863
CHUDEA8_600	cgd8_600	0.964	0.0992	0.0142	0.143145161
CHUDEA8_60	cgd8_60	0.791	0.1442	0.1077	0.746879334
CHUDEA8_610	cgd8_610	0.981	0.062	0.0077	0.124193548
CHUDEA8_620	cgd8_620	0.931	0.0776	0.0279	0.359536082
CHUDEA8_630	cgd8_630	0.984	0.056	0.0082	0.146428571
CHUDEA8_640	cgd8_640	0.973	0.0782	0.009	0.115089514
CHUDEA8_650	cgd8_650	1	0.0752	0	0
CHUDEA8_660	cgd8_660	0.856	0.0647	0.0496	0.766615147
CHUDEA8_700	cgd8_700	0.869	0.0951	0.0367	0.385909569
CHUDEA8_70	cgd8_70	0.981	0.0539	0.0084	0.155844156
CHUDEA8_710	cgd8_710	0.972	0.0542	0.0101	0.186346863
CHUDEA8_720	cgd8_720	0.956	0.0454	0.0241	0.530837004
CHUDEA8_730	cgd8_730	1	0.073	0	0
CHUDEA8_740	cgd8_740	0.956	0.0579	0.0186	0.321243523

CHUDEA8_750	cgd8_750	0.989	0.0572	0.0054	0.094405594
CHUDEA8_760	cgd8_760	0.976	0.059	0.01	0.169491525
CHUDEA8_770	cgd8_770	0.973	0.0415	0.0123	0.296385542
CHUDEA8_780	cgd8_780	0.973	0.0767	0.0085	0.110821382
CHUDEA8_790	cgd8_790	0.996	0.0741	0.0015	0.020242915
CHUDEA8_800	cgd8_800	0.98	0.0726	0.0059	0.081267218
CHUDEA8_80	cgd8_80	0.998	0.1015	0.0018	0.01773399
CHUDEA8_810	cgd8_810	0.956	0.0921	0.0177	0.19218241
CHUDEA8_820	cgd8_820	0.946	0.0625	0.026	0.416
CHUDEA8_830	cgd8_830	0.94	0.0583	0.0273	0.468267581
CHUDEA8_840	cgd8_840	1	0.0692	0	0
CHUDEA8_850	cgd8_850	0.971	0.0499	0.0145	0.290581162
CHUDEA8_860	cgd8_860	0.991	0.0787	0.0047	0.059720457
CHUDEA8_870	cgd8_870	0.987	0.0677	0.0051	0.075332349
CHUDEA8_880	cgd8_880	0.967	0.0815	0.0045	0.055214724
CHUDEA8_890	cgd8_890	0.961	0.0968	0.0162	0.167355372
CHUDEA8_900	cgd8_900	0.967	0.0489	0.0145	0.296523517
CHUDEA8_90	cgd8_90	0.964	0.0569	0.0173	0.304042179
CHUDEA8_910	cgd8_910	0.982	0.0502	0.008	0.15936255
CHUDEA8_920	cgd8_920	0.969	0.0752	0.0129	0.171542553
CHUDEA8_930	cgd8_930	0.992	0.071	0.0034	0.047887324
CHUDEA8_940	cgd8_940	0.988	0.089	0.0049	0.05505618
CHUDEA8_950	cgd8_950	0.99	0.0508	0.0043	0.084645669
CHUDEA8_960	cgd8_960	0.979	0.0575	0.0086	0.149565217
CHUDEA8_970	cgd8_970	0.965	0.0695	0.0125	0.179856115
CHUDEA8_980	cgd8_980	0.973	0.0601	0.0116	0.193011647
CHUDEA8_990	cgd8_990	0.973	0.0546	0.0116	0.212454212
CHUDEA8_new_01	cgd8_new_01	0.973	0.0795	0.0133	0.167295597
CHUDEA8_new_02	cgd8_new_02	0.983	0.0907	0.0077	0.084895259
CHUDEA8_new_03	cgd8_new_03	0.942	0.0778	0.0266	0.341902314
CHUDEA8_new_04	cgd8_new_04	0.951	0.099	0.0208	0.21010101
CHUDEA8_new_05	cgd8_new_05	0.987	0.026	0.0052	0.2
CHUDEA8_new_06	cgd8_new_06	0.968	0.0589	0.0134	0.227504244
CHUDEA8_new_07	cgd8_new_07	0.996	0.1208	0.0016	0.013245033
CHUDEA8_new_08	cgd8_new_08	0.958	0.049	0.0185	0.37755102
CHUDEA8_new_09	cgd8_new_09	0.986	0	0.0063	1000
CHUDEA8_new_10	cgd8_new_10	0.958	0.0646	0.0194	0.300309598
CHUDEA8_new_12	cgd8_new_12	0.97	0.0737	0.0139	0.188602442
CHUDEA8_new_13	cgd8_new_13	0.982	0.0336	0.0081	0.241071429
CHUDEA8_new_14	cgd8_new_14	0.976	0.0493	0.0108	0.219066937
